# Rh-catalysed single-carbon insertion to 1,3-dienes†

**DOI:** 10.1039/d5sc03161c

**Published:** 2025-06-19

**Authors:** Pau Sarró, Norman Díaz, Josep Esteve Guasch, Wei Jie Teo, Marcos G. Suero

**Affiliations:** a Institute of Chemical Research of Catalonia (ICIQ-CERCA), The Barcelona Institute of Science and Technology Av. Països Catalans 16 43007 Tarragona Spain mgsuero@iciq.es; b ICREA Pg. Lluis Companys 23 08010 Barcelona Spain; c Departament de Química Analítica i Química Orgànica, Universitat Rovira i Virgili Calle Marcel·lí Domingo, 1 Tarragona 43007 Spain

## Abstract

Herein, we report the first catalytic single-carbon insertion to 1,3-dienes with Rh(ii)-carbynoids. The skeletal editing process is based on the catalytic generation of a Rh-carbynoid that promotes the insertion of a cationic monovalent carbon unit (:^+^C–R) into the C(sp^*2*^)–C(sp^*2*^) bond of the 1,3-diene, leading to a pentadienyl cation. Regioselective attack on the latter species leads to the formation of multi-substituted 1,3-dienes or 1,4-dienes with a broad range of carbon and heteroatomic nucleophiles.

## Abstract 2

A novel skeletal editing of 1,3-dienes mediated by Rh-carbynoids enabled the selective synthesis of 1,3- and 1,4-dienes.

## Graphical abstract

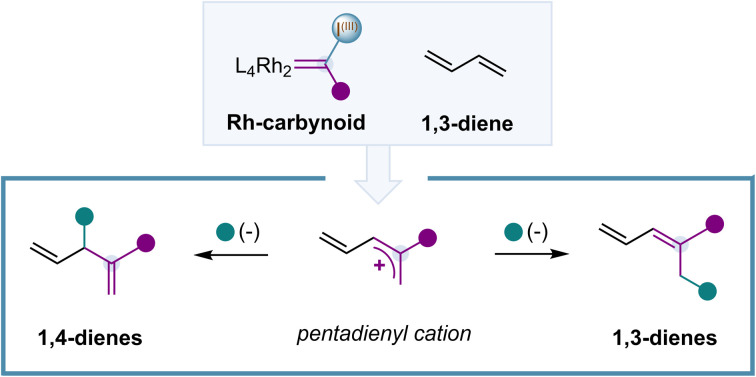

Since the discovery of the Diels–Alder reaction over a century ago,^1^ 1,3-dienes have become one of the most important building blocks in the synthesis of complex natural products, drug molecules and polymers.^2^ Transition-metal and photoredox catalysis have played a central role in the discovery and development of both efficient stereoselective syntheses and chemical transformations of 1,3-dienes based on 1,2- and 1,4-difunctionalizations (Scheme 1A).^3^ Such processes rely on π-bond activations of the C(sp^2^)–C(sp^2^) double bonds that can occur with excellent diastereo-, regio- and enantiocontrol.^4^ However, catalytic processes that can functionalize 1,3-dienes through σ- and π-bond activations of the 1,3-diene C(sp^2^)–C(sp^2^) bonds are unexplored and remain limited to cross metathesis of 1,3-dienes and alkenes (Scheme 1A).^5^

**Scheme 1:**
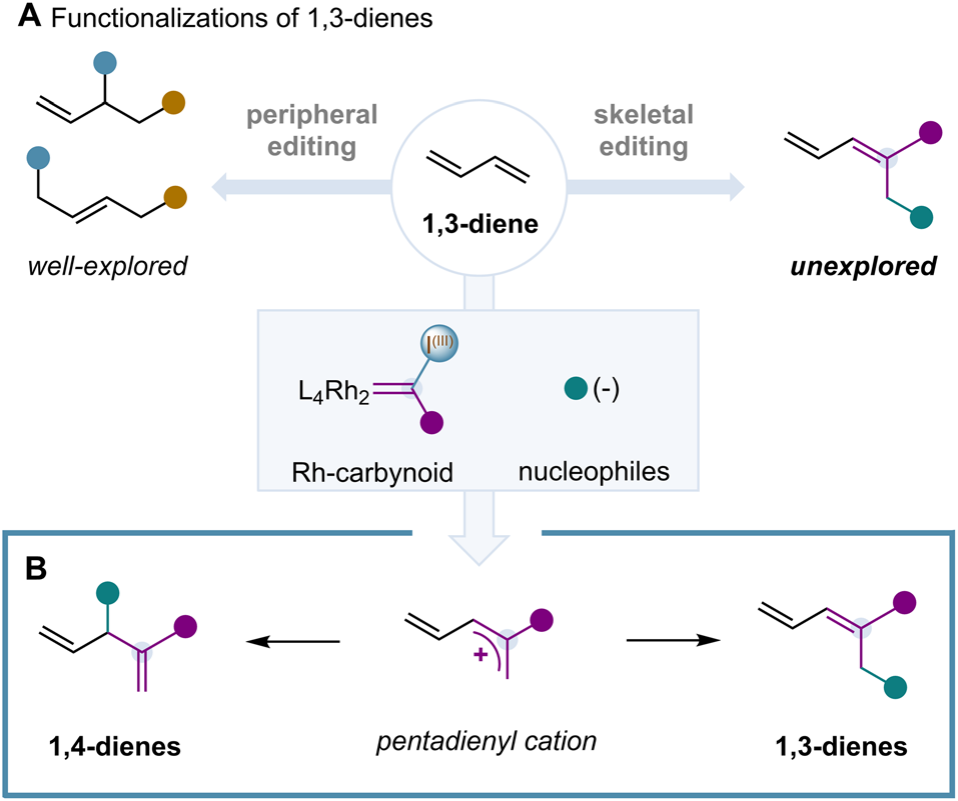
Peripheral and skeletal editing of 1,3-dienes.

Over the recent years, the discovery and development of single-carbon insertion reactions in unsaturated^6^ and saturated^7^ systems have received enormous attention. These types of skeletal manipulations involving the insertion of a single-carbon atom are of high interest since they provide new retrosynthetic logic and disconnection approaches.^8^ Given the interest of our group in developing a general catalytic carbyne transfer platform, we recently questioned whether a novel catalytic cationic monovalent carbon insertion (:^+^C–R) of 1,3-dienes could be within reach. If successful, this previously unknown transformation would complement cross metathesis as a skeletal editing process for 1,3-dienes, while providing a novel single-carbon insertion logic.

Herein, we disclose the first single-carbon insertion of 1,3-dienes induced by a catalytically generated Rh-carbynoid (Scheme 1B). These species were responsible for the generation of a transient pentadienyl cation that underwent regioselective attack by a wide range of nucleophiles, leading to 1,3- or 1,4-dienes.

Our group is interested in developing a general catalytic carbyne transfer platform using the (photo)catalytic activation of a novel class of diazomethyl-substituted hypervalent iodine reagents.^9^ In 2019, we reported the first catalytic generation of Rh(ii)-carbynoid species Rh=C–I^(III)^(*E*) [I^(III)^ = I^(III)^(Ar)(X); E = ester]^10^ using dirhodium carboxylate complexes.^11^ We disclosed that Rh(ii)-carbynoids promoted the skeletal manipulation of alkenes by inserting a cationic monovalent carbon unit (:^+^C–R) into the alkene C(sp^2^)–C(sp^2^) bond, resulting in the generation of allylic cations that were trapped by a broad range of nucleophiles in an inter- and intramolecular fashion. Moreover, we recently showed that such single-carbon insertion could occur with excellent regio- and enantiocontrol using chiral dirhodium catalysts.^12^ Experimental evidence supported the transient generation of a chiral intimate allyl cation–nucleophile pair.^13^

Recently, we hypothesized that single-carbon insertion into 1,3-dienes mediated by Rh-carbynoids could be of interest, considering that a pentadienyl cation may be generated. While these types of cations are known to undergo Nazarov 4π-conrotatory electrocyclizations that ultimately lead to the formation of 2-cyclopentenones,^14^ studies on their behavior towards nucleophilic attack remain largely unexplored.^15^ We envisioned that a cyclopropanation reaction between a 1,3-diene and Rh-carbynoid *int-1* would generate a cyclopropyl–I^(III)^*int-3,* placing the vinyl group and hypervalent iodine moiety in a *syn* disposition.^10^ Analogous to previous results from our group, this diastereoselectivity may be explained based on *int-2*, where the non-reactive double bond prevents steric clashes with the ester group, as seen in int-2* (purple ball). Then, a disrotatory ring-opening would lead to a pentadienyl cation *int-4* with three available electrophilic positions (α, γ, δ) (Scheme 2) that could lead to three different types of 1,3 and 1,4-dienes 3.

**Scheme 2:**
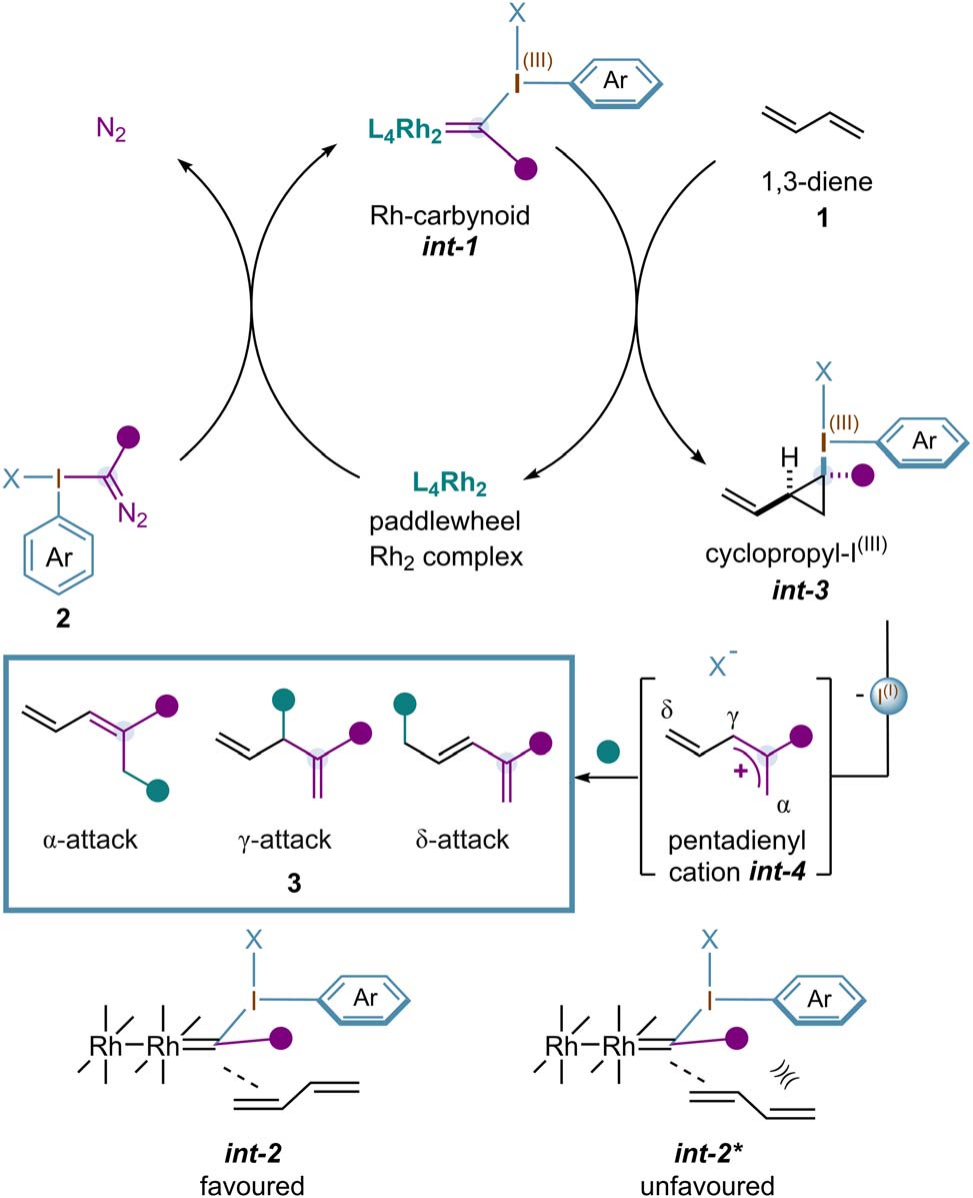
Mechanistic proposal.

Initial experiments were carried out with 1,3-butadiene (1a, 5.0 eq.) – a feedstock chemical produced on a >10 million ton scale per year^16^ – hypervalent iodine reagent^17^2a, Rh_2_(OAc)_4_ or Rh_2_(HFIB)_4_ (1.0 mol%) as catalysts, Bu_4_NBr (1.1 eq.) as the nucleophile and CH_2_Cl_2_ as the solvent (Table 1, entries 1,2). Unfortunately, we did not observe the formation of compound 3a. However, sterically demanding dirhodium catalysts such as Rh_2_(TPA)_4_, Rh_2_(Adc)_4_ or Rh_2_esp_2_ (Du Bois catalyst)^18^ provided 3a in good yields (entries 3–5, 73–80% yield) and with a 3 : 1 *Z* : *E* ratio. With the aim of improving the diastereoselectivity of the reaction, we explored a range of ester substituents on the hypervalent iodine reagent (2b–d; R = i-Pr, Bn, CH_2_CCl_3_) and found a superior *Z* : *E* ratio with a trichloroethyl substituent (entries 6–8). Finally, we observed that while an excess of 1,3-butadiene was necessary for the efficiency of the reaction (entry 9), a higher amount of Bu_4_NBr provides higher diastereoselectivity at the cost of yield (entry 10).

**Table 1:** Optimization studies^a^

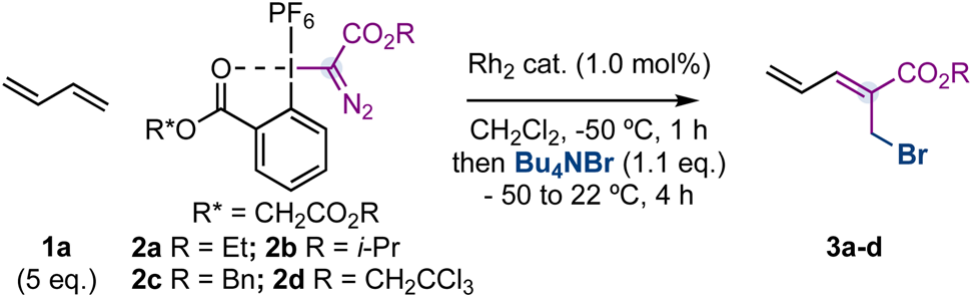				
Entry	2	Catalyst	Yield 3a–d^b^ [%]	Ratio *Z* : *E*^c^
1	2a	Rh_2_(OAc)_4_	0	—
2	2a	Rh_2_(HFIB)_4_	0	—
3	2a	Rh_2_(TPA)_4_	73	3 : 1
4	2a	Rh_2_(Adc)_4_	74	3 : 1
5	2a	Rh_2_esp_2_	80	3 : 1
6	2b	Rh_2_esp_2_	75	3 : 1
7	2c	Rh_2_esp_2_	73	3 : 1
8	2d	Rh_2_esp_2_	76(82)^d^	5 : 1
9	2d	Rh_2_esp_2_	48	5 : 1^e^
10	2d	Rh_2_esp_2_	52	8 : 1^f^

a Reactions were carried out with 1,3-butadiene (0.5 mmol), Rh catalyst (1.0 mol%) and reagent 2 (0.1 mmol) in CH_2_Cl_2_ (1.5 mL) at −50 °C for 60 min. Bu_4_NBr was added neat, and the tube was kept in the cooling bath and slowly warmed to rt over 4 h.

b Yield reported on the basis of ^1^H-NMR analysis of the crude reaction mixture using CH_2_Br_2_ as the internal standard.

c Ratio of diastereoisomers was determined using ^1^H-NMR analysis of the crude reaction mixture.

d Isolated yield.

e Using 0.1 mmol of 1,3-butadiene.

f Using 0.2 mmol of Bu_4_NBr. esp = α, α, α′, α′-tetramethyl-1,3-benzenedipropanoate. HFIB = heptafluorobutyrate. TPA = triphenylacetate. Adc = 1-adamantylcarboxylate.

We next turned our attention to evaluate a range of heteroatomic nucleophiles under the optimized reaction conditions (Table 2A). We were delighted to observe that alternative halide sources (3b,c), alcohols (3d,e), or phosphates (3f,g) were well tolerated. We noticed that while diastereoselectivities were maintained (*E* : *Z* ratios), regioselectivities (linear : branched ratios) were superior for sterically demanding nucleophiles (see 3a*vs.*3c, 3d*vs.*3e, 3f*vs.*3g). Unfortunately, amines such as morpholine, *p*-anisidine and dibenzylamine did not work under the optimised reaction conditions. Electron-rich (hetero)aromatic rings, such as 1,3,5-trimethoxybenzene (3h), furan (3i), thiophene (3j) and *N*-Boc-protected pyrrole (3k), led to the corresponding products in moderate yields with excellent regioselectivity. In contrast with such results, benzene provided 3l in poor yield. Then, we tested a range of phenyl derivatives and found that while organoboron compounds (PhBPin or PhB(OH)_2_), organosilicon (PhTMS) and organotin (PhSnBu_3_) provided poor efficiency (≤20% yield), the Molander potassium phenyltrifluoroborate^19^ provided 3l in 41% yield. The addition of tetrabutylammonium bisulfate (TBAHSO_4_) as a phase transfer agent to the reaction mixture increased the efficiency of the process (3l, 56%).^20^

**Table 2:** Scope of the Rh-catalysed single-carbon insertion in 1,3-butadiene^a^

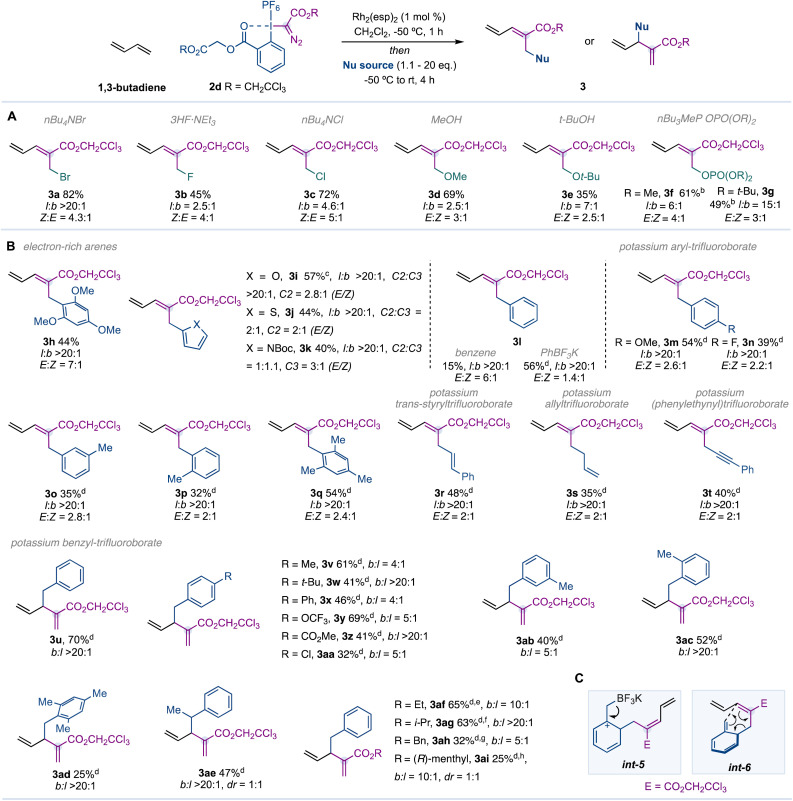

a Reactions were carried out with 1,3-butadiene (1.0 mmol), Rh_2_(esp)_2_ (1.0 mol%) and reagent 2d (0.2 mmol) in CH_2_Cl_2_ (3.0 mL) for 1 h at −50 °C. Nucleophile (1.1–20 mmol) was added neat, and the tube was kept in the cooling bath and slowly warmed to rt over 4 h. Yields are reported on the basis of the isolated pure product using flash column chromatography.

b Nucleophile was added in CH_2_Cl_2_ (2.0 mL) dropwise over 10 min at −50 °C.

c NaHCO_3_ (0.4 mmol) was added from the beginning.

d nBu_4_NHSO_4_ (1.0 equiv.) was added together with the nucleophile at −50 °C.

e Reagent 2a (0.2 mmol) was used.

f Reagent 2b (0.2 mmol) was used.

g Reagent 2c (0.2 mmol) was used.

h Reagent 2e (0.2 mmol) was used.

We then observed that alternative *para*-, *meta*- and *ortho*-substituted aryltrifluoroborates were well tolerated (3m–q). Vinylic, allylic and alkynyl Molander salts were also effective and provided the corresponding 1,3-dienes with excellent regioselectivity (3r–t).

In contrast with these observations, benzyl trifluoroborate salts provided 1,4-dienes from a presumable attack on the γ-position (3u–ai). It is interesting to see that substitutions on the phenyl ring (3v–ad), benzylic position (3ae) or the use of alternative reagents (3af–ai) did not prevent the attack on the γ-position (Table 2B). A reaction mechanism that could explain the preferred formation of the 1,4-diene may involve an *ortho*-selective electrophilic aromatic substitution of the Molander salt with the pentadienyl cation *int-4* at the alpha position. Elimination of BF_3_ in *int-5* would lead to *int-6*, which may undergo a 3,3-sigmatropic rearrangement, leading to the corresponding 1,4-diene (Table 2C).^21^ However, we cannot rule out the possibility of a direct attack of the benzyl nucleophile on the γ-position.

We next turned our attention to exploit our Rh-catalysed single carbon-insertion with substituted 1,3-dienes and tributylmethylphosphonium dimethylphosphate as the nucleophile (Scheme 3A). Under the optimized reaction conditions, isoprene led to a mixture of allylic phosphates 4a. We noticed that the major isomers found come from a preferred insertion into the more substituted double bond, consistent with prior observations reported for other metallocarbenes.^22^ In contrast, reactions carried out with 1,3-dienes substituted at C1 provided 1,3-dienes 4b,c with excellent levels of diastereo- and regioselectivity (*l* : *b* > 20 : 1; *E* : *Z* > 20 : 1) and cyclic dienes such as 1,3-cyclohexadiene provided 4d with good regioselectivity.

**Scheme 3:**
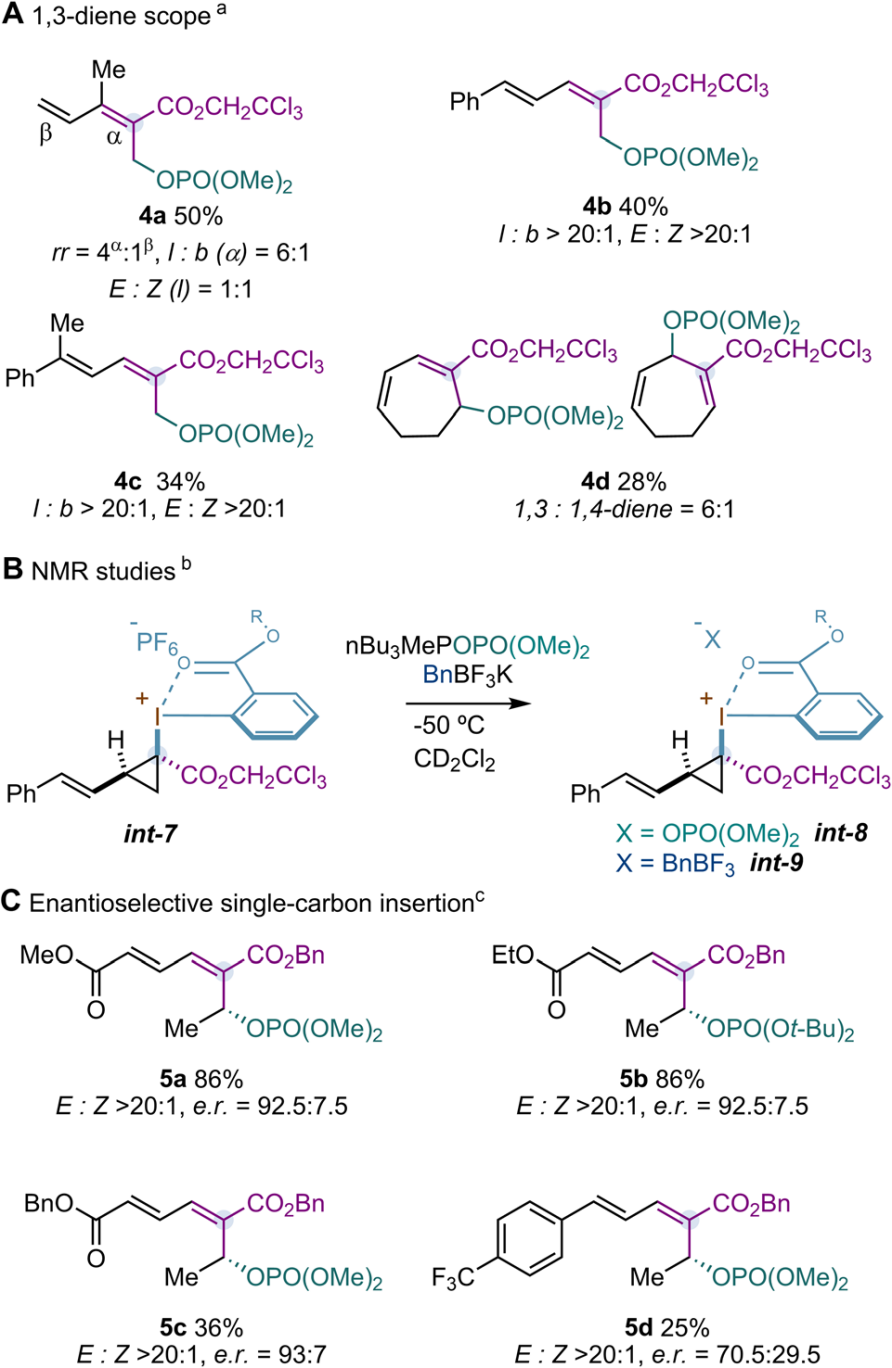
1,3-Diene scope, NMR studies and enantioselective single-carbon insertion. ^a^ Reactions carried out with diene 1 (1.0 mmol), Rh_2_(esp)_2_ (1.0 mol%) and reagent 2d (0.2 mmol) in CH_2_Cl_2_ (3.0 mL) for 1 h at −50 °C. Then, nBu_3_MePOPO(OMe)_2_ (0.6 mmol) in CH_2_Cl_2_ was dropwise added over 10 min at −50 °C and the tube was kept in the cooling bath and slowly warmed to rt over 4 h. Yields are reported on the basis of the isolated pure product using flash column chromatography. ^b^ Reaction carried out with 1,3-diene 1c (0.2 mmol), Rh_2_(esp)_2_ (1.0 mol%) and reagent 2d (0.1 mmol) in CD_2_Cl_2_ (1.5 mL) for 1 h at −50 °C. Then, *int-7* was observed by ^1^H NMR. After this, a solution of the nucleophile (0.3 mmol) in CD_2_Cl_2_ (1.0 mL) was added dropwise over 10 min at −50 °C. Then, *int-8* and *int-9* were observed by ^1^H NMR. ^c^ Reactions carried out with 1,3-dienes 1 (0.2 mmol), Rh_2_(*S*-NTTL)_4_(AcOEt)_2_ (5.0 mol%) and reagent 2c (0.24 mmol) in CH_2_Cl_2_ : PhCl (3.0 mL, 1 : 1) for 1.5 hours at −60 °C. Then, nucleophile (0.6 mmol) was added in CH_2_Cl_2_ dropwise over 10 min at −60 °C and the tube was allowed to warm to rt over 1 h. Enantiomeric ratios (e.r.) were determined by supercritical fluid chromatography mass spectrometry (SFC-MS) analysis on a chiral stationary phase of the isolated pure product by using flash column chromatography. The absolute configuration of 1,3-diene products 5 was assigned by analogy to that confirmed for styrenes.^12^

A reaction carried out between 1-phenyl-1,3-butadiene and reagent 2d with Rh_2_(esp)_2_ at −50 °C in CD_2_Cl_2_ allowed us to detect and characterize the cyclopropyl–I^(III)^–PF_6_*int-7* (Scheme 3B). The relative configuration assigned using NOESY experiments showed that the styryl and I^(III)^ moieties were in a relative *cis* disposition. Addition of 3.0 equiv. of dimethylphosphate and benzyl Molander salt at −50 °C promoted the formation of cyclopropyl–I^(III)^–OPO(OMe)_2_*int-8* and cyclopropyl–I^(III)^–F_3_BBn *int-9,* as observed by ^1^H NMR. As previously observed, a downfield chemical shift of the proton *o*-H to Ar–I^(III)^ was observed. This was diagnostic to invoke the formation of *int-8*. With this information, we wondered whether analogue *int-8* could evolve through an S_N_1-like S_N_i mechanism as previously observed for cyclopropyl–I^(III)^–OPO(OMe)_2_ intermediates derived from styrenes.^12^

Under the optimized reaction conditions previously developed using [Rh_2_(*S*-NTTL)_4_](AcOEt)_2_ (*S*-NTTL = *N*-naphthaloyl-(*S*)-*tert*-leucinate), benzylester reagent 2c, and CH_2_Cl_2_ : PhCl (1 : 1) as the solvent, we found that substituted sorbic acid esters could provide the desired 1,3-dienes 5a–c with high asymmetric induction (Scheme 3C). It is worth highlighting that the single-carbon insertion occurred with excellent site-selectivity towards the remote double bond to the ester group. Analogous to alkenes, the excellent enantiocontrol could be explained based on the enantioselective formation of cyclopropyl–I^(III)^–PF_6_ that, upon anion exchange, evolves into cyclopropyl–I^(III)^–dialkylphosphate and subsequently to the final products 5a–c through an S_N_1-like S_N_i mechanism. Unfortunately, such excellent levels of enantiocontrol were not observed in other substrates (5d).

## Conclusions

In conclusion, we have developed a catalytic single-carbon insertion to 1,3-dienes with Rh(ii)-carbynoids. We have demonstrated that this process can transform simple 1,3-dienes into complex 1,3- and 1,4-substituted dienes *via* σ- and π-bond activation. The value of the constructive scission of this kind of C(sp^2^)–C(sp^2^) bonds is exemplified in its versatile nucleophilic substitution, as well as in the enantiomeric control achieved for some examples. This reaction adds to the new methodologies concerning skeletal editing processes that involve single-carbon insertion into C(sp^2^)–C(sp^2^) bonds.

## Author contributions

P. S., N. D., J. E. G., W. J. T. & M. G. S. planned the experiments. P. S., N. D., J. E. G. & W. J. T. performed the experiments. All authors contributed to the analysis and interpretation of the data. M. G. S. directed the project and wrote the manuscript with contributions from all authors.

## Conflicts of interest

There are no conflicts to declare.

## Supplementary Material

SC-OLF-D5SC03161C-s001

## Data Availability

The data supporting this article have been included as part of the ESI.†
