# Supplementary material for: Rh-catalysed single-carbon insertion to 1,3-dienes
Source: Chem Sci. 2025 Jun 19;16(28):13042–7. doi: 10.1039/d5sc03161c (PMC12177889; doi:10.1039/d5sc03161c)
Supplement: SC-016-D5SC03161C-s001 [file SC-016-D5SC03161C-s001.pdf]

Supplementary Information (SI) for Chemical Science.
This journal is © The Royal Society of Chemistry 2025

Rh-Catalysed Single-Carbon Insertion to 1,3-Dienes

Pau Sarró[a,c,‡], Norman Díaz[a,c,‡], Josep Esteve Guasch[a,c], Wei Jie Teo[a] and Marcos G. Suero*[a,b]

[a] Institute of Chemical Research of Catalonia (ICIQ-CERCA), The Barcelona Institute of Science and Technology, Països Catalans 16, 43007 Tarragona, Spain. [b] ICREA, Pg. Lluis Companys 23, 08010 Barcelona, Spain. [c] Departament de Química Analítica i Química Orgánica, Universitat Rovira i Virgili, Calle Marcel·lí Domingo, 1, Tarragona, 43007, Spain.

*Correspondence to: mgsuero@iciq.es

‡ These authors contributed equally to this work.

Supplementary Information

Table of Contents

1. General Information *1*

2. Starting Materials *2*

2.1 Synthesis of 1,3-dienes 1. .... **2**

2.2 Synthesis of hypervalent iodine reagents 2. .... **2**

2.3 Synthesis of potassium trifluoroborate salts. **4**

3. Rh-catalysed single-carbon insertion to 1,3-dienes: reaction optimization and scope ***4***

4. Low temperature ^{1}H NMR studies and detection of cyclopropyl-I(III) intermediates ***39***

5. References ***43***

6. NMR spectra ***44***

1. General Information

All reagents were used as purchased and employed with no further purification. Rhodium(II) acetate dimer $Rh_2(OAc)_4$, rhodium(II) heptafluorobutyrate dimer $Rh_2(HFIB)_4$, bis[rhodium(α,α,α′,α′-tetramethyl-1,3-benzenedipropionic acid)] $Rh_2(esp)_2$ and rhodium(II) triphenylacetate dimer $Rh_2(TPA)_4$ were purchased from Sigma-Aldrich. Rhodium bis(1-adamantate) dimer $Rh_2(Adc)_4$ and Rh_2(*S*-NTTL)$_4$(AcOEt)$_2$ was prepared according to reported procedures.[1,2] Ethyl diazoacetate (contains ≥13 wt. % dichloromethane, Ref. E22201), 1,3-butadiene solution (contains 15 wt. % butadiene in hexane, Ref. 695904), isoprene, (*E*)-buta-1,3-dien-1-ylbenzene, 1,3-cyclohexadiene and tetrabutylammonium hydrogensulfate (97 %, Ref. 155837) were purchased from Sigma-Aldrich and used without further purification. Anhydrous solvents were dried by passing through an activated alumina column on a PureSolv™ solvent purification system (Innovative Technologies, Inc., MA). Analytical thin layer chromatography (TLC) was carried out using aluminum sheets with 0.2 mm of silica gel (Merck GF234). Visualization of the developed chromatogram was performed by irradiation with UV light or treatment with a solution of potassium permanganate stain followed by heating. Flash column chromatography was performed on silica gel (Aldrich, 230-400 mesh) or neutral silica gel (Material Harvest Ltd., 230-400 mesh). Organic solutions were concentrated under reduced pressure on a Büchi rotatory evaporator. Unless otherwise stated, reactions were carried out under argon atmosphere. Yields refer to purified compounds unless otherwise noted. NMR spectra were recorded at 298 K (unless otherwise stated) on Bruker Avance 300, Bruker Avance 400 Ultrashield and Bruker Avance 500 Ultrashield apparatuses. Chemical shifts (δ) are quoted in ppm relative to residual solvent signals, $CDCl_3$ referenced at δ 7.26 and 77.2 ppm, CD_2Cl_2 referenced at δ 5.32 and 53.5 ppm, CD_3CN referenced at δ 1.94 and 1.3, 118.3 ppm respectively. Coupling constants (*J*) are quoted in hertz (Hz). Multiplicity is reported with the following abbreviations: s = singlet, brs = broad singlet, d = doublet, t = triplet, q = quartet, p = quintet, dt = doublet of triplets, td = triplet of doublets, tt = triplet of triplets, sp = septet, m = multiplet, app = apparent. Mass spectra were recorded on a Waters LCT Premier spectrometer and Agilent 1260 Infinity - 6130 Quadrupole. Gas chromatographymass spectrometry (GC-MS) analyses were carried out in Agilent 7890B - 5977A MSD. The enantiomeric ratios were determined by SFC-MS analysis on a chiral stationary phase performed on Agilent 1260 Infinity II SFC system on Daicel chiral columns unless otherwise stated.

2. Starting Materials.

2.1 Synthesis of 1,3-dienes 1.

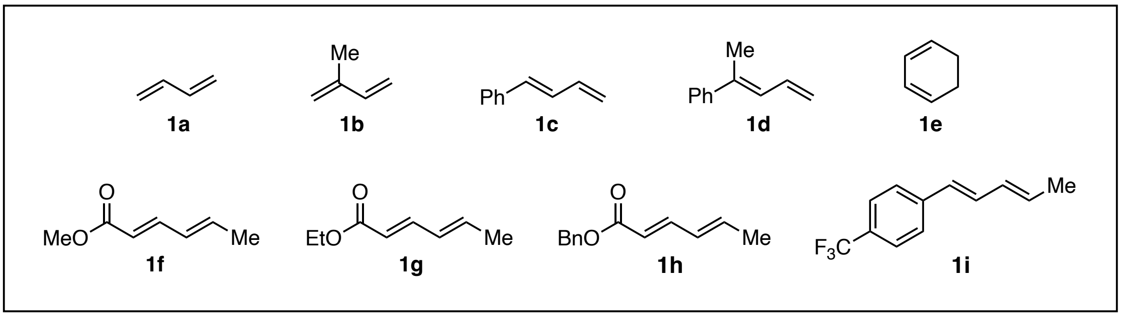

Substrates **1a**, **1b**, **1c** and **1e** were purchased and used without further purification. Substrates **1d**[3], **1f**[4], **1g**[4], **1h**[5] and **1i**[6] are known compounds and were synthesised following the corresponding reported protocols.

2.2 Synthesis of hypervalent iodine reagents 2.

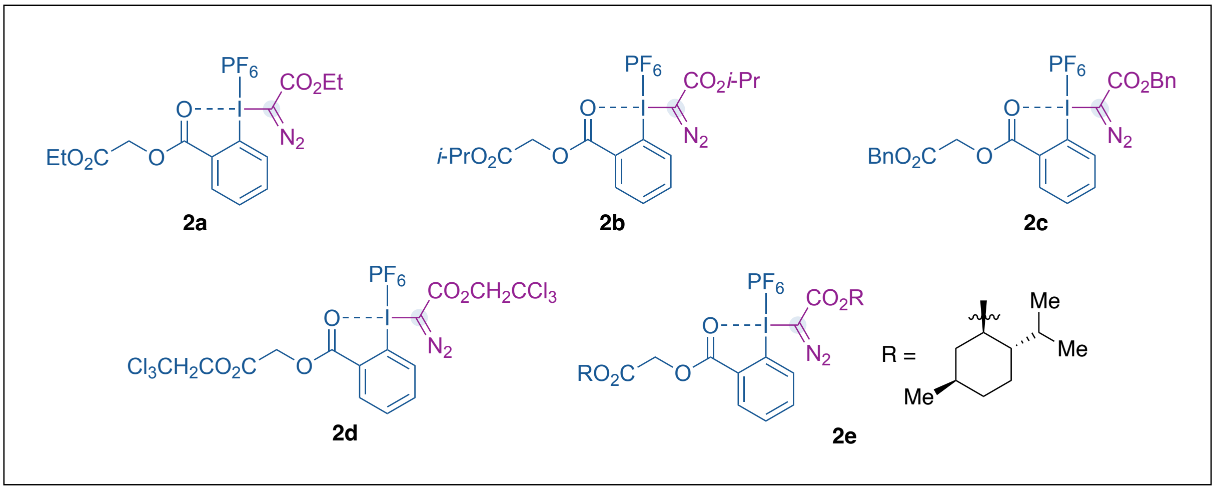

Hypervalent iodine reagents **2a**, **2b**, **2c** and **2e** are known compounds and were prepared following the reported literature protocols.[7] Reagent **2d** is new and was synthesised according to the following procedure:

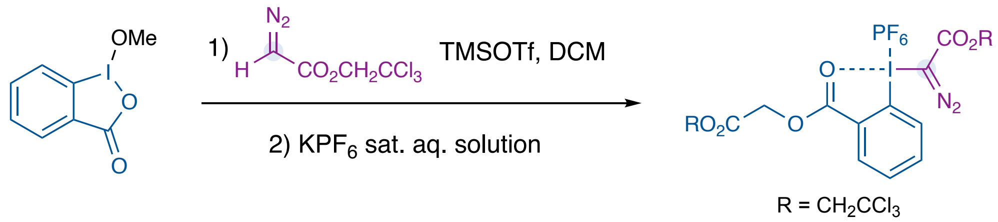

A solution of 1-methoxy-1,2-benziodoxol-3(*1H*)-one (1.7 g, 6.0 mmol, 1.0 equiv.) in dichloromethane (12 mL) was treated with trimethylsilyl trifluoromethanesulfonate (1.3 g, 6.0 mmol, 1.0 equiv.) at room temperature. After 30 min, a cloudy suspension was observed and 2,2,2-trichloroethyl diazoacetate[8] (3.0 g, 13.8 mmol, 2.3 equiv.) was added dropwise during 10 minutes. Nitrogen evolution was observed, and the resulting reaction mixture was stirred at room temperature until a clear yellow solution was observed (1 h). Solvent was removed under reduced pressure, redissolved in dichloromethane (30 mL) and washed with a saturated aqueous solution of KPF_6 in a separation funnel. The combined organic layers were dried over Na_2SO_4 and solvent was removed under reduced pressure. The crude mixture was sonicated in the mixture of Et_2O/CH_2Cl_2 (10:1) and decanted from the yellow solid residue. The yellow solid was dried under reduced pressure to obtain the product **2d** as a yellow solid (3.4 g, 70 % yield for two steps). (*Note: if the product contains impurities, a recrystallization process using CH_2Cl_2/Et_2O may be done at -30 ºC. Store the product under argon at -30 ºC*)

(1-diazo-2,2,2-trichloroethyl)(2-(2,2,2-trifluoroethyl-2-oxoethoxyl)carbonylphenyl)iodonium hexafluorophosphate (2d)

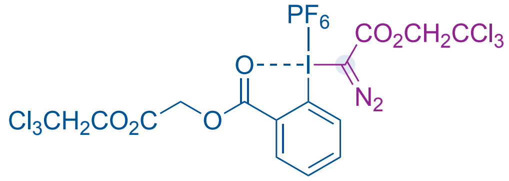

^{1}H NMR (400 MHz, Acetone-*d$_6$*) δ 8.55 (dd, *J* = 7.5, 1.7 Hz, 1H), 8.38 (d, *J* = 8.4 Hz, 1H), 8.15 (ddd, *J* = 8.4, 7.5, 1.7 Hz, 1H), 8.04 (td, *J* = 7.5, 0.9 Hz, 1H), 5.45 (s, 2H), 5.07 (s, 2H), 5.01 (s, 2H).
^{13}C NMR (126 MHz, $CDCl_3$) δ 169.7, 164.7, 160.1, 139.0, 133.9, 132.5, 128.9, 124.9, 115.3, 94.2, 94.1, 75.6, 74.7, 63.6. (the resonance resulting from the carbonyl carbon in the 2,2,2-trichloroethyl ester was not detected)
^{19}F NMR (376 MHz, Acetone-*d$_6$*) δ -71.8 (d, *J* = 708.7 Hz).
^{31}P NMR (162 MHz, Acetone-*d$_6$*) δ -141.17 (hept, *J* = 708.7 Hz).
HRMS (MALDI): calculated for $C_{15}H_{10}Cl_6IN_2O_6$ $[M-PF_6]^+$ m/z: 650.7685, found: 650.7670.

2.3 Synthesis of potassium trifluoroborate salts.

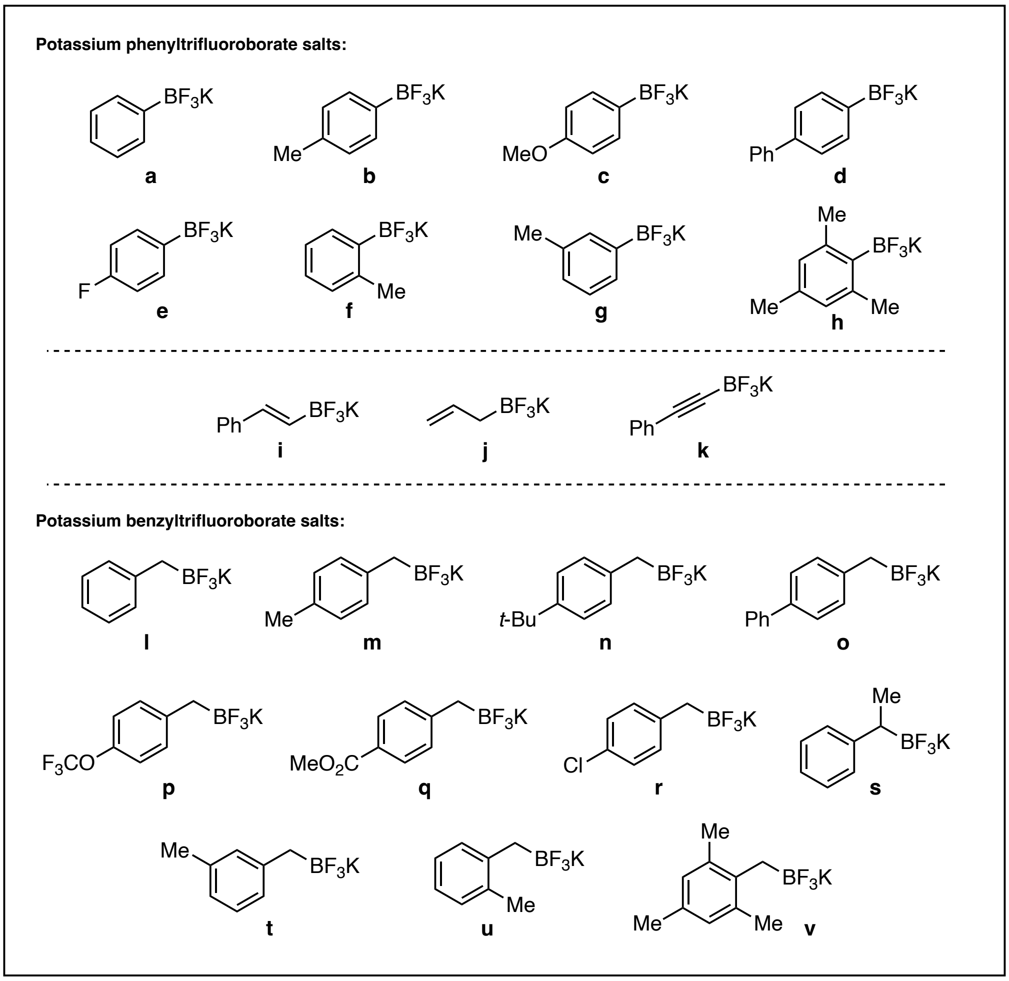

Substrates **a**, **b**, **c**, **i**, **j**, **k** and **l** were purchased and used without further purification. Substrates **d**[9], **e**[10], **f**[10], **g**[11], **h**[12], **m**[13], **n**[14], **o**[15], **p**[16], **q**[14], **r**[13], **s**[17], **t**[16], **u**[17] and **v**[13] are known compounds and were synthesised following the corresponding reported protocols.

3. Rh-catalysed single-carbon insertion to 1,3-dienes: reaction optimization and scope

General procedure A:

To a 10 mL oven-dried reaction tube equipped with a stirring bar was added the corresponding dirhodium catalyst (0.001 mmol, 1.0 mol%). The tube was sealed before being evacuated and backfilled with argon three times. 1,3-Butadiene (15% v/v in hexane) and degassed dichloromethane (0.5 mL) were added and the resulting mixture was cooled at -50 ºC. Then, a solution of the

corresponding reagent **2** (0.1 mmol, 1.0 equiv.) in degassed dichloromethane (1.0 mL) was added dropwise during 1 h using a syringe pump. Then, the desired nucleophile and additive (when indicated) were added to the reaction mixture. The tube was kept in the cooling bath and slowly warmed to room temperature during 4 h. After that, the reaction mixture was filtered through a short plug of silica gel and washed with dichloromethane. Solvent was removed under reduced pressure and the crude residue was analyzed by GC-MS and ^{1}H-NMR using dibromomethane as internal standard.

Table S1. Optimization table

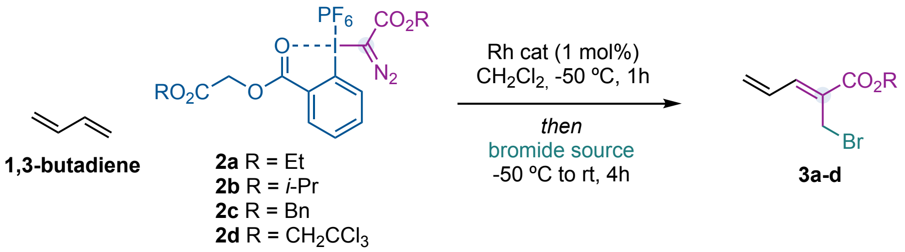

Entry	Reagent **2**	Rh catalyst	Ratio **1**:**2**	bromide source (eq.)	Yield **3** (%)[a]	*Z*:*E*[b]
1	**2a**	Rh_2esp_2	5 : 1	nBu_4NBr (1.1)	80	3:1
2	**2a**	$Rh_2(OAc)_4$	5 : 1	nBu_4NBr (1.1)	n.d.	-
3	**2a**	$Rh_2(HFIB)_4$	5 : 1	nBu_4NBr (1.1)	n.d.	-
4	**2a**	$Rh_2(TPA)_4$	5 : 1	nBu_4NBr (1.1)	73	3:1
5	**2a**	$Rh_2(Adc)_4$	5 : 1	nBu_4NBr (1.1)	74	3:1
6	**2b**	Rh_2esp_2	5 : 1	nBu_4NBr (1.1)	75	3:1
7	**2c**	Rh_2esp_2	5 : 1	nBu_4NBr (1.1)	73	3:1
8	**2d**	Rh_2esp_2	5 : 1	nBu_4NBr (1.1)	76 (80)[c]	5:1
9	**2d**	Rh_2esp_2	10 : 1	nBu_4NBr (1.1)	74	5:1
10	**2d**	Rh_2esp_2	1 : 1	nBu_4NBr (1.1)	48	5:1
11	**2d**	Rh_2esp_2	1 : 2	nBu_4NBr (1.1)	n.d.	-
12[d]	**2d**	Rh_2esp_2	5 : 1	nBu_4NBr (1.1)	70	6:1
13[e]	**2d**	Rh_2esp_2	5 : 1	nBu_4NBr (1.1)	n.d.	-
14	**2d**	Rh_2esp_2	5 : 1	nBu_4NBr (2.0)	52	8:1
15	**2d**	Rh_2esp_2	5 : 1	nBu_4NBr (5.0)	45	4:1
16	**2d**	Rh_2esp_2	5 : 1	Bu_4PBr (1.1)	72	7:1
17	**2d**	Rh_2esp_2	5 : 1	Me_3SiBr (1.1)	n.d.	-

[a] Yields reported on the basis of ^{1}H-NMR analysis using dibromomethane as internal standard. [b] The ratio of diastereoisomers were reported on the basis of ^{1}H-NMR analysis of the crude. [c] Isolated yield. [d] Reaction run at -40 ºC. [e] Reaction run at -78 ºC. esp = α, α, α', α'-tetramethyl-1,3-benzenedipropanoate. HFIB = heptafluorobutyrate. TPA = triphenylacetate. Adc = adamantylcarboxylate

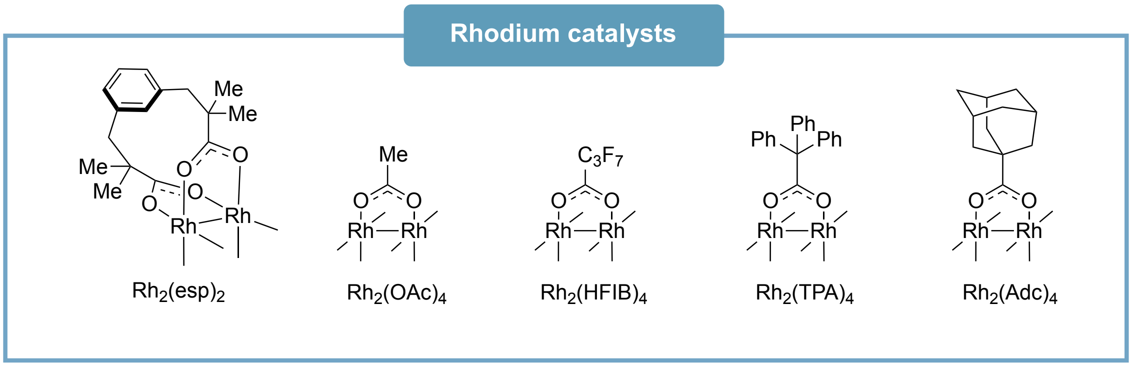

Table S2. Optimization table for the use of benzyl as nucleophile

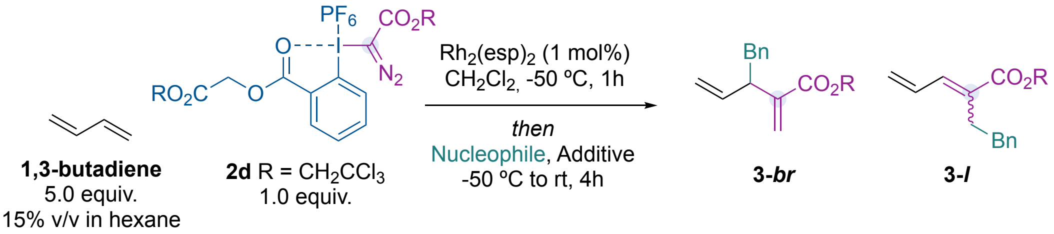

Entry	Additive (equiv.)	Nucleophile (equiv.)	Yield (%)[a]	Ratio **3, *br*:*l***[b]
1	-	$BnBF_3K$ (2.5)	41	>20:1
2	-	nBu4NBnBF$_3$ (2.5)	< 10	-
3	nBu_4NHSO_4 (1.0)	$BnBF_3K$ (2.5)	51	>20:1
4	nBu_4NHSO_4 (1.0)	$BnBF_3K$ (5.0)	65 (70)[c]	>20:1
5	nBu_4NHSO_4 (1.0)	$BnBF_3K$ (10)	53	>20:1
6	nBu_4NHSO_4 (10 mol %)	$BnBF_3K$ (2.5)	18	>20:1
7	nBu_4NHSO_4 (0.5)	$BnBF_3K$ (2.5)	36	>20:1
8	nBu_4NHSO_4 (2.5)	$BnBF_3K$ (5.0)	56	>20:1
9	18-Crown-6 (1.0)	$BnBF_3K$ (5.0)	51	>20:1
10	$nBu_4NH_2PO_4$ (1.0)	$BnBF_3K$ (5.0)	47	>20:1
11	nBu_4NPF_6 (1.0)	$BnBF_3K$ (5.0)	47	>20:1
12	nBu_4NBPh_4 (1.0)	$BnBF_3K$ (5.0)	< 10	-

[a] Yields refer to the addition of **3*br*** and **3*l*** and were reported on the basis of ^{1}H-NMR analysis using dibromomethane as internal standard. [b] Refers to the ratio of *branched* to *linear* regioisomers and were reported on the basis of ^{1}H-NMR analysis of the crude. [c] Isolated yield. 18-Crown-6 = 1,4,7,10,13,16-hexaoxacyclooctadecane.

General procedure B:

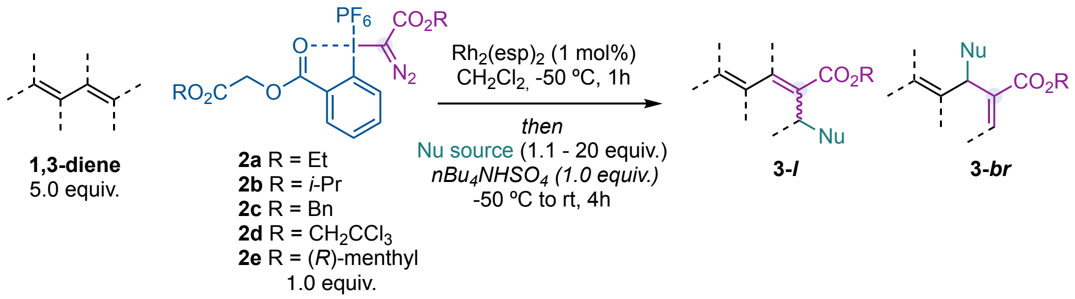

To a 10 mL oven-dried reaction tube equipped with a stirring bar was added $Rh_2(esp)_2$ (1.5 mg, 0.002 mmol, 1.0 mol%). The tube was sealed before being evacuated and backfilled with argon three times. The corresponding 1,3-diene **1** (1.0 mmol, 5.0 equiv.) and degassed dichloromethane (1.0 mL) were added and the resulting mixture was cooled at -50 ºC. Then, a solution of reagent **2** (0.2 mmol, 1.0 equiv.) in degassed dichloromethane (2.0 mL) was added dropwise during 1 h using a syringe pump. Then, the desired nucleophile (1.1 - 20 equiv.) and tetrabutylammonium hydrogensulfate (67.9 mg, 0.2 mmol, 1.0 equiv.; when indicated) were added to the reaction mixture. The tube was kept in the cooling bath and slowly warmed to room temperature during 4 hours. After that, the reaction mixture was filtered through a short plug of silica gel and washed with dichloromethane. Solvent was removed under reduced pressure and the crude residue was purified by flash column chromatography to yield dienes.

2,2,2-trichloroethyl (*Z*)-2-(bromomethyl)penta-2,4-dienoate (3a)

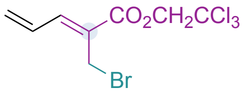

Prepared according to the general procedure B using 1,3-butadiene (492 µL 15 % v/v in hexane, 1.0 mmol), reagent **2d** (159.8 mg, 0.2 mmol) and tetrabutylammonium bromide (70.9 mg, 0.22 mmol). Ratio of *linear:branched* isomers was determined to be >20:1 (*linear* = 4.3:1, *Z/E*) using ^{1}H-NMR analysis of the crude reaction mixture. Purification by flash column chromatography on silica gel (hexane/diethyl ether 100:1) provided a mixture of 1,3-dienes as a colorless oil (53.9 mg, 82% yield).

Major isomer

^{1}H NMR (500 MHz, $CDCl_3$) δ 7.42 (dt, *J* = 11.6, 0.5 Hz, 1H), 6.79 (ddd, *J* = 16.6, 11.6, 10.0, Hz, 1H), 5.85 (ddd, *J* = 16.7, 1.5, 0.9 Hz, 1H), 5.79 (ddd, *J* = 10.0, 1.5, 0.7 Hz, 1H), 4.88 (s, 2H), 4.37 (s, 2H).

^{13}C NMR (126 MHz, $CDCl_3$) δ 164.4, 144.3, 131.0, 129.7, 127.3, 95.0, 74.7, 23.5.

HRMS (APCI) calculated for $C_8H_8Cl_3O_2^+$ $[M-Br]^+$ m/z: 240.9584, found: 240.9585.
^{1}H-^{1}H NOESY, ^{1}H-^{1}H COSY, ^{1}H-^{13}C HSQC, ^{1}H-^{13}C HMBC spectra were measured.

2,2,2-trichloroethyl (*Z*)-2-(fluoromethyl)penta-2,4-dienoate (3b)

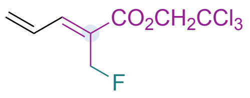

Prepared according to the general procedure B using 1,3-butadiene (492 µL 15 % v/v in hexane, 1.0 mmol), reagent **2d** (159.8 mg, 0.2 mmol) and triethylamine trihydrofluoride (108 µL, 0.6 mmol). Ratio of *linear:branched* isomers was determined to be 2.5:1 (*linear* = 4:1, *Z/E*) using ^{1}H-NMR analysis of the crude reaction mixture. Purification by flash column chromatography on silica gel (hexane/diethyl ether 100:1) provided a mixture of 1,3-dienes as a colorless oil (23.5 mg, 45% yield).
Major isomer
^{1}H NMR (500 MHz, $CDCl_3$) δ 7.56 (dd, *J* = 11.6, 3.7 Hz, 1H), 6.87 (dddd, *J* = 16.7, 11.7, 10.0, 1.8 Hz, 1H), 5.84 (dq, *J* = 16.7, 1.1 Hz, 1H), 5.79 – 5.73 (m, 1H), 5.29 (d, *J* = 47.5 Hz, 2H), 4.86 (s, 2H).
^{13}C NMR (126 MHz, $CDCl_3$) δ 164.8 (d, *J* = 2.4 Hz), 147.1 (d, *J* = 5.5 Hz), 131.1 (d, *J* = 2.1 Hz), 130.2 (d, *J* = 3.4 Hz), 125.2 (d, *J* = 14.8 Hz), 95.0, 76.1 (d, *J* = 165.0 Hz), 74.6.
^{19}F NMR (471 MHz, $CDCl_3$) δ -179.7.
HRMS (APCI) calculated for $C_8H_8Cl_3FO_2^+$ $[M]^+$ m/z: 259.9568, found: 259.9563.

2,2,2-trichloroethyl (*Z*)-2-(chloromethyl)penta-2,4-dienoate (3c)

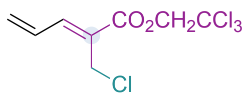

Prepared according to the general procedure B using 1,3-butadiene (492 µL 15 % v/v in hexane, 1.0 mmol), reagent **2d** (159.8 mg, 0.2 mmol) and tetrabutylammonium chloride (61.1 mg, 0.22 mmol). Ratio of *linear:branched* isomers was determined to be 4.6:1 (*linear* = 5:1, *Z/E*) using ^{1}H-NMR analysis of the crude reaction mixture. Purification by flash column chromatography on silica gel (hexane/diethyl ether 100:1) provided a mixture of 1,3-dienes as a colorless oil (40.0 mg, 72% yield).
Major isomer
^{1}H NMR (500 MHz, $CDCl_3$) δ 7.45 (dt, *J* = 11.6, 0.5 Hz, 1H), 6.80 (ddd, *J* = 16.6, 11.5, 10.0 Hz, 1H), 5.85 (ddd, *J* = 16.6, 1.4, 0.9 Hz, 1H), 5.77 (dq, *J* = 10.0, 0.7 Hz, 1H), 4.87 (s, 2H), 4.47 (s, 2H).

^{13}C NMR (126 MHz, $CDCl_3$) δ 164.5, 144.9, 130.9, 129.9, 127.0, 95.0, 74.7, 36.9.
HRMS (APCI) calculated for $C_8H_8Cl_4O_2^+$ $[M]^+$ m/z: 275.9273, found: 275.9273.

2,2,2-trichloroethyl (*Z*)-2-(methoxymethyl)penta-2,4-dienoate (3d)

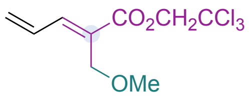

Prepared according to the general procedure B using 1,3-butadiene (492 µL 15 % v/v in hexane, 1.0 mmol), reagent **2d** (159.8 mg, 0.2 mmol) and methanol (128 µL, 4 mmol). Ratio of *linear:branched* isomers was determined to be 2.5:1 (*linear* = 3:1, *Z/E*) using ^{1}H-NMR analysis of the crude reaction mixture. Purification by flash column chromatography on silica gel (hexane/diethyl ether 50:1) provided a mixture of 1,3-dienes as a colorless oil (37.7 mg, 69% yield).

Major isomer

^{1}H NMR (400 MHz, $CDCl_3$) δ 7.49 (d, J = 11.5 Hz, 1H), 6.87 (ddd, J = 16.7, 11.4, 10.0 Hz, 1H), 5.76 (ddd, J = 16.8, 1.6, 0.9 Hz, 1H), 5.66 (ddd, J = 10.0, 1.6, 0.7 Hz, 1H), 4.84 (s, 2H), 4.32 (s, 2H), 3.38 (s, 3H).
^{13}C NMR (101 MHz, $CDCl_3$) δ 165.7, 145.3, 131.7, 128.5, 127.1, 95.2, 74.6, 65.9, 58.5.
HRMS (APCI) calculated for $C_9H_{11}Cl_3O_3^+$ $[M]^+$ m/z: 271.9768, found: 271.9771.

2,2,2-trichloroethyl (*Z*)-2-(*tert*-butoxymethyl)penta-2,4-dienoate (3e)

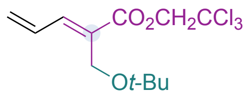

Prepared according to the general procedure B using 1,3-butadiene (492 µL 15 % v/v in hexane, 1.0 mmol), reagent **2d** (159.8 mg, 0.2 mmol) and *tert*-butanol (148 µL, 4 mmol). Ratio of *linear:branched* isomers was determined to be 7:1 (*linear* = 2.5:1, *Z/E*) using ^{1}H-NMR analysis of the crude reaction mixture. Purification by flash column chromatography on silica gel (hexane/diethyl ether 50:1) provided a mixture of 1,3-dienes as a colorless oil (22.1 mg, 35% yield).

Major isomer

¹H NMR (400 MHz, $CDCl_3$) δ 7.44 (d, *J* = 11.4 Hz, 1H), 6.87 (ddd, *J* = 16.8, 11.4, 10.0 Hz, 1H), 5.71 (ddd, *J* = 16.8, 1.7, 0.9 Hz, 1H), 5.62 (ddd, *J* = 10.0, 1.7, 0.8 Hz, 1H), 4.82 (s, 2H), 4.28 (s, 2H), 1.27 (s, 9H).

¹³C NMR (101 MHz, $CDCl_3$) δ 165.9, 144.9, 132.0, 128.2, 127.8, 95.3, 74.6, 73.9, 56.0, 27.7.

HRMS (APCI) calculated for $C_8H_8Cl_3O_2^+$ [M-O*t*-Bu]$^+$ m/z: 240.9584, found: 240.9584.

2,2,2-trichloroethyl (*Z*)-2-(((dimethoxyphosphoryl)oxy)methyl)penta-2,4-dienoate (3f)

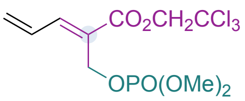

Prepared according to the general procedure B using 1,3-butadiene (492 µL 15 % v/v in hexane, 1.0 mmol), reagent **2d** (159.8 mg, 0.2 mmol) and tributyl(methyl)phosphonium dimethylphosphate (205.4 mg, 0.6 mmol) in dichloromethane (2.0 mL) dropwise during 10 min. Ratio of *linear:branched* isomers was determined to be 6:1 (*linear* = 4:1, *Z/E*) using ¹H-NMR analysis of the crude reaction mixture. Purification by flash column chromatography on silica gel (hexane/ethyl acetate 1:1) provided a mixture of 1,3-dienes as a colorless oil (35.6 mg, 48% yield).

Major isomer

¹H NMR (500 MHz, $CDCl_3$) δ 7.53 (d, *J* = 11.5 Hz, 1H), 6.92 (ddd, *J* = 16.6, 11.5, 10.0 Hz, 1H), 5.84 (dt, *J* = 16.7, 1.0 Hz, 1H), 5.75 (dt, *J* = 10.0, 1.0 Hz, 1H), 4.95 (d, *J* = 7.6 Hz, 2H), 4.86 (s, 2H), 3.75 (d, *J* = 11.1 Hz, 6H).

¹³C NMR (126 MHz, $CDCl_3$) δ 164.8, 146.7, 131.1, 130.2, 125.0 (d, *J* = 7.3 Hz), 95.0, 74.6, 60.6 (d, *J* = 5.0 Hz), 54.5 (d, *J* = 5.9 Hz).

³¹P NMR (202 MHz, $CDCl_3$) δ 4.13.

HRMS (ESI) calculated for $C_{10}H_{14}Cl_3NaO_6P^+$ [M+Na]$^+$ m/z: 388.9486, found: 388.9487.

2,2,2-trichloroethyl (*Z*)-2-(((di-*tert*-butoxyphosphoryl)oxy)methyl)penta-2,4-dienoate (3g)

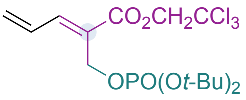

Prepared according to the general procedure B using 1,3-butadiene (492 µL 15 % v/v in hexane, 1.0 mmol), reagent **2d** (159.8 mg, 0.2 mmol) and tetrabutylammonium di-*tert*-butyl phosphate (270.6

mg, 0.6 mmol) in dichloromethane (2.0 mL) dropwise during 10 min. Ratio of *linear:branched* isomers was determined to be 15:1 (*linear* = 3:1, *Z/E*) using ^{1}H-NMR analysis of the crude reaction mixture. Purification by flash column chromatography on silica gel (hexane/ethyl acetate 2:1) provided a mixture of 1,3-dienes as a colorless oil (44.3 mg, 49% yield).

Major isomer

^{1}H NMR (500 MHz, $CDCl_3$) δ 7.49 (d, *J* = 11.5 Hz, 1H), 6.96 (ddd, *J* = 16.8, 11.5, 10.0 Hz, 1H), 5.78 (ddd, *J* = 16.7, 1.6, 0.8 Hz, 1H), 5.72 – 5.68 (m, 1H), 4.87 (d, *J* = 7.3 Hz, 2H), 4.85 (s, 2H), 1.48 (d, *J* = 0.6 Hz, 18H).

^{13}C NMR (126 MHz, $CDCl_3$) δ 165.1, 146.1, 131.7, 129.3, 125.8 (d, *J* = 7.9 Hz), 82.7 (d, *J* = 7.4 Hz), 74.6, 59.8 (d, *J* = 5.7 Hz), 30.0, 30.0.

^{31}P NMR (202 MHz, $CDCl_3$) δ -6.91.

HRMS (ESI) calculated for $C_{16}H_{26}Cl_3NaO_6P^+$ $[M+Na]^+$ m/z: 473.0402, found: 473.0399.

2,2,2-trichloroethyl (*E*)-2-(2,4,6-trimethoxybenzyl)penta-2,4-dienoate (3h)

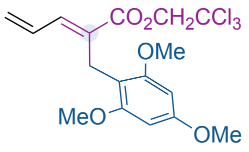

Prepared according to the general procedure B using 1,3-butadiene (492 µL 15 % v/v in hexane, 1.0 mmol), reagent **2d** (159.8 mg, 0.2 mmol) and 1,3,5-trimethoxybenzene (336.4 mg, 2 mmol). Ratio of *linear:branched* isomers was determined to be >20:1 (*linear* = 7:1, *E/Z*) using ^{1}H-NMR analysis of the crude reaction mixture. Purification by flash column chromatography on silica gel (hexane/diethyl ether 50:1) provided a mixture of 1,3-dienes as a colorless oil (36.1 mg, 72% yield).

Major isomer

^{1}H NMR (500 MHz, $CDCl_3$) δ 7.13 (dt, *J* = 11.3, 0.8 Hz, 1H), 6.78 (ddd, *J* = 16.8, 11.3, 10.0 Hz, 1H), 6.09 (s, 2H), 5.49 (ddd, *J* = 16.9, 2.0, 0.9 Hz, 1H), 5.39 (ddd, *J* = 10.0, 1.9, 0.8 Hz, 1H), 4.81 (s, 2H), 3.78 (s, 3H), 7.77 (s, 2H), 3.76 (s, 6H).

^{13}C NMR (126 MHz, $CDCl_3$) δ 167.2, 159.8, 158.7, 139.7, 132.8, 131.7, 124.6, 109.4, 95.5, 90.8, 74.6, 55.8, 55.4, 20.9.

HRMS (APCI) calculated for $C_{17}H_{20}Cl_3O_5^+$ $[M+H]^+$ m/z: 409.0371, found: 409.0381.

2,2,2-trichloroethyl (*E*)-2-(furan-2-ylmethyl)penta-2,4-dienoate (3i)

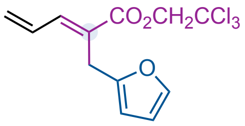

Prepared according to the general procedure B using 1,3-butadiene (492 µL 15 % v/v in hexane, 1.0 mmol), reagent **2d** (159.8 mg, 0.2 mmol), sodium hydrogencarbonate (34 mg, 0.4 mmol) and furan (136 mg, 2.0 mmol). Ratio of *linear:branched* isomers was determined to be >20:1 (*C2:C3 attack* >20:1, *C2* = 2.8:1 *(E/Z)*) using ^{1}H-NMR analysis of the crude reaction mixture. Purification by flash column chromatography on silica gel (hexane/ethyl acetate 100:1) provided a mixture of 1,3-dienes as a colorless oil (35.3 mg, 57% yield).

Major isomer

^{1}H NMR (400 MHz, $CDCl_3$) δ 7.45 (d, *J* = 11.4 Hz, 1H), 7.29 (dd, *J* = 1.9, 1.0 Hz, 1H), 6.81 (ddd, *J* = 16.7, 11.4, 10.0 Hz, 1H), 6.26 (dd, *J* = 3.2, 1.9 Hz, 1H), 6.02 (dq, *J* = 3.2, 1.0 Hz, 1H), 5.74 (ddd, *J* = 16.7, 1.6, 0.8 Hz, 1H), 5.63 (dd, *J* = 10.0, 1.6, 0.8 Hz, 1H), 4.81 (s, 2H), 3.84 (s, 2H).

^{13}C NMR (101 MHz, $CDCl_3$) δ 165.9, 152.5, 142.7, 141.6, 131.9, 127.5, 126.8, 110.5, 106.3, 95.2, 74.6, 25.8.

HRMS (APCI) calculated for $C_{12}H_{12}Cl_3O_3^+$ $[M+H]^+$ m/z: 308.9847, found: 308.9846.

^{1}H-^{1}H NOESY, ^{1}H-^{1}H COSY, ^{1}H-^{13}C HSQC, ^{1}H-^{13}C HMBC spectra were measured.

2,2,2-trichloroethyl (*E*)-2-(thiophen-2-ylmethyl)penta-2,4-dienoate (3j)

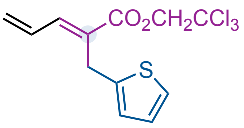

Prepared according to the general procedure B using 1,3-butadiene (492 µL 15 % v/v in hexane, 1.0 mmol), reagent **2d** (159.8 mg, 0.2 mmol) and thiophene (168 mg, 2.0 mmol). Ratio of *linear:branched* isomers was determined to be >20:1 (*C2:C3 attack* = 2:1, *C2* = 2:1 *(E/Z)*, *C3* = 2:1 *(E/Z)*) using ^{1}H-NMR analysis of the crude reaction mixture. Purification by flash column chromatography on silica gel (hexane/ethyl acetate 100:1) provided a mixture of 1,3-dienes as a colorless oil (30.9 mg, 44% yield).

Major isomer

^{1}H NMR (500 MHz, $CDCl_3$) δ 7.44 (d, *J* = 11.4 Hz, 1H), 7.11 (dd, *J* = 5.1, 1.2 Hz, 1H), 6.89 (dd, *J* = 5.1, 3.5 Hz, 1H), 6.84 – 6.82 (m, 1H), 6.82 – 6.76 (m, 1H), 5.76 (dd, *J* = 16.7, 2.5 Hz, 1H), 5.64 (dd, *J* = 10.0, 2.4 Hz, 1H), 4.82 (s, 2H), 4.01 (s, 2H).

^{13}C NMR (126 MHz, $CDCl_3$) δ 165.9, 142.0, 141.6, 131.7, 129.2, 127.7, 127.0, 125.2, 124.0, 95.2, 74.6, 27.2.

HRMS (ESI) calculated for $C_{12}H_{11}Cl_3NaO_2S^+$ $[M+H]^+$ m/z: 346.9438, found: 346.9436.

^{1}H-^{1}H NOESY, ^{1}H-^{1}H COSY, ^{1}H-^{13}C HSQC, ^{1}H-^{13}C HMBC spectra were measured.

tert-butyl (_E_)-3-(2-((2,2,2-trichloroethoxy)carbonyl)penta-2,4-dien-1-yl)-1_H_-pyrrole-1-carboxylate (3k)

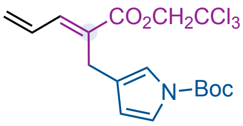

Prepared according to the general procedure B using 1,3-butadiene (492 μL 15 % v/v in hexane, 1.0 mmol), reagent **2d** (159.8 mg, 0.2 mmol) and N-Boc-pyrrole (335 mg, 2.0 mmol). Ratio of *linear:branched* isomers was determined to be >20:1 (*C3:C2 attack* = 1.1:1, *C3* = 3:1 *(E/Z)*, *C2* = 6:1 *(E/Z)*) using ^{1}H-NMR analysis of the crude reaction mixture. Purification by flash column chromatography on silica gel (hexane/ethyl acetate 50:1) provided a mixture of 1,3-dienes as a colorless oil (32.7 mg, 40% yield).

Major isomer

^{1}H NMR (500 MHz, $CDCl_3$) δ 7.40 (d, *J* = 11.4 Hz, 1H), 7.12 (t, *J* = 2.7 Hz, 1H), 6.98 (d, *J* = 4.6 Hz, 1H), 6.79 (ddd, *J* = 16.8, 11.4, 10.0 Hz, 1H), 6.07 (dd, *J* = 3.3, 1.7 Hz, 1H), 5.72 (ddd, *J* = 16.8, 1.7, 0.9 Hz, 1H), 5.59 (dd, *J* = 10.0, 2.4 Hz, 1H), 4.81 (s, 2H), 3.62 (s, 2H), 1.56 (s, 9H).

^{13}C NMR (126 MHz, $CDCl_3$) δ 166.3, 149.0, 141.4, 131.9, 129.7, 126.9, 124.7, 120.3, 117.5, 112.8, 95.3, 83.6, 74.6, 28.1, 24.5.

HRMS (ESI) calculated for $C_{17}H_{20}Cl_3NNaO_4^+$ $[M+H]^+$ m/z: 430.0350, found: 430.0346.

^{1}H-^{1}H NOESY, ^{1}H-^{1}H COSY, ^{1}H-^{13}C HSQC, ^{1}H-^{13}C HMBC spectra were measured.

2,2,2-trichloroethyl (*E*)-2-benzylpenta-2,4-dienoate (3l)

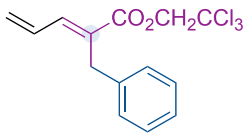

Prepared according to the general procedure B using 1,3-butadiene (492 µL 15 % v/v in hexane, 1.0 mmol), reagent **2d** (159.8 mg, 0.2 mmol), potassium phenyltrifluoroborate (184.0 mg, 1.0 mmol) and tetrabutylammonium hydrogensulfate (67.9 mg, 0.2 mmol). Ratio of *linear:branched* isomers was determined to be >20:1 (*linear* = 1.4:1, *E/Z*) using ^{1}H-NMR analysis of the crude reaction mixture. Purification by flash column chromatography on silica gel (hexane/diethyl ether 100:1) provided a mixture of 1,3-dienes as a colorless oil (35.8 mg, 56% yield).

Major isomer

^{1}H NMR (500 MHz, $CDCl_3$) δ 7.49 (d, *J* = 11.4 Hz, 1H), 7.29 – 7.16 (m, 5H), 6.82 (ddd, *J* = 16.7, 11.4, 10.1 Hz, 1H), 5.75 (ddd, *J* = 16.7, 0.8, 0.7 Hz, 1H), 5.64 – 5.60 (m, 1H), 4.77 (s, 2H), 3.85 (s, 2H).

^{13}C NMR (126 MHz, $CDCl_3$) δ 166.3, 142.0, 139.0, 132.0, 129.7, 128.6, 128.5, 127.2, 126.5, 95.2, 74.6, 32.7.

HRMS (APCI) calculated for $C_{14}H_{14}Cl_3O_2^+$ $[M+H]^+$ m/z: 319.0054, found: 319.0047.

Other phenyl nucleophiles were tested without the addition of tetrabutylammonium hydrogensulfate providing product **3i** in bad yields (10 – 20%): benzene (15%), phenyl boronic acid pinacol ester (10%), phenyl boronic acid (20%) and tributylphenylstannate (10%).

2,2,2-trichloroethyl (*E*)-2-(4-methoxybenzyl)penta-2,4-dienoate (3m)

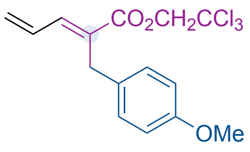

Prepared according to the general procedure B using 1,3-butadiene (492 µL 15 % v/v in hexane, 1.0 mmol), reagent **2d** (159.8 mg, 0.2 mmol), potassium (4-fluorophenyl)trifluoroborate (214.0 mg, 1.0 mmol) and tetrabutylammonium hydrogensulfate (67.9 mg, 0.2 mmol). Ratio of *linear:branched* isomers was determined to be >20:1 (*linear* = 2.6:1, *E/Z*) using ^{1}H-NMR analysis of the crude reaction

mixture. Purification by flash column chromatography on silica gel (hexane/diethyl ether 100:1) provided a mixture of 1,3-dienes as a colorless oil (37.8 mg, 54% yield).

Major isomer

^{1}H NMR (400 MHz, $CDCl_3$) δ 7.46 (d, J = 11.4 Hz, 1H), 7.14 – 7.09 (m, 2H), 6.90 – 6.81 (m, 1H), 6.82 – 6.78 (m, 2H), 5.74 (ddd, J = 16.7, 1.7, 0.9 Hz, 1H), 5.61 (ddd, J = 10.0, 1.7, 0.8 Hz, 1H), 4.77 (d, J = 2.3 Hz, 2H), 3.79 – 3.77 (m, 2H), 3.77 (s, 3H).

^{13}C NMR (101 MHz, $CDCl_3$) δ 166.3, 158.3, 141.6, 132.0, 131.1, 130.2, 129.4, 127.1, 114.0, 95.2, 74.5, 55.4, 31.8.

HRMS (APCI) calculated for $C_{15}H_{16}Cl_3O_3^+$ $[M+H]^+$ m/z: 349.0160, found: 349.0159.

2,2,2-trichloroethyl (*E*)-2-(4-fluorobenzyl)penta-2,4-dienoate (3n)

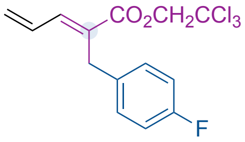

Prepared according to the general procedure B using 1,3-butadiene (492 µL 15 % v/v in hexane, 1.0 mmol), reagent **2d** (159.8 mg, 0.2 mmol), potassium (4-fluorophenyl)trifluoroborate (202.0 mg, 1.0 mmol) and tetrabutylammonium hydrogensulfate (67.9 mg, 0.2 mmol). Ratio of *linear:branched* isomers was determined to be >20:1 (*linear* = 2.2:1, *E/Z*) using ^{1}H-NMR analysis of the crude reaction mixture. Purification by flash column chromatography on silica gel (hexane/diethyl ether 100:1) provided a mixture of 1,3-dienes as a colorless oil (26.3 mg, 39% yield).

Major isomer

^{1}H NMR (500 MHz, $CDCl_3$) δ 7.48 (dt, J = 11.4, 0.7 Hz, 1H), 7.18 – 7.13 (m, 2H), 7.00 – 6.92 (m, 2H), 6.79 (ddt, J = 16.7, 11.4, 10.0 Hz, 1H), 5.77 (ddd, J = 16.7, 1.6, 0.9 Hz, 1H), 5.64 (ddd, J = 10.0, 1.6, 0.7 Hz, 1H), 4.77 (s, 2H), 3.80 (s, 2H).

^{13}C NMR (126 MHz, $CDCl_3$) δ 166.1, 160.7, 142.0, 131.7, 129.9 (d, J = 7.9 Hz), 127.8, 127.6, 124.1 (d, J = 2.8 Hz), 115.4 (d, J = 21.4 Hz), 95.1, 74.5, 31.9.

LRMS (ESI) calculated for $C_{14}H_{12}Cl_3FO_2^+$ $[M]^+$ m/z: 335.99, found: 335.99.

2,2,2-trichloroethyl (*E*)-2-(3-methylbenzyl)penta-2,4-dienoate (3o)

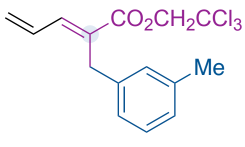

Prepared according to the general procedure B using 1,3-butadiene (492 µL 15 % v/v in hexane, 1.0 mmol), reagent **2d** (159.8 mg, 0.2 mmol), potassium (3-methylphenyl)trifluoroborate (198.0 mg, 1.0 mmol) and tetrabutylammonium hydrogensulfate (67.9 mg, 0.2 mmol). Ratio of *linear:branched* isomers was determined to be >20:1 (*linear* = 2:1, *E/Z*) using ^{1}H-NMR analysis of the crude reaction mixture. Purification by flash column chromatography on silica gel (hexane/diethyl ether 100:1) provided a mixture of 1,3-dienes as a colorless oil (23.4 mg, 35% yield).

Major isomer

^{1}H NMR (500 MHz, $CDCl_3$) δ 7.47 (dd, *J* = 11.3, 7.5 Hz, 1H), 7.17 – 7.13 (m, 1H), 7.11 – 7.05 (m, 2H), 7.03 – 6.95 (m, 1H), 6.82 (ddd, *J* = 16.7, 11.3, 10.0 Hz, 1H), 5.74 (ddd, *J* = 16.7, 1.7, 0.9 Hz, 1H), 5.61 (ddd, *J* = 10.0, 1.7, 0.9 Hz, 1H), 4.77 (s, 2H), 3.80 (s, 2H), 2.30 (s, 3H)

^{13}C NMR (126 MHz, $CDCl_3$) δ 166.3, 142.6, 141.9, 136.0, 132.0, 129.3, 128.3, 127.2, 127.1, 125.4, 95.2, 74.5, 32.2, 21.1, 20.0.

HRMS (ESI) calculated for $C_{15}H_{15}Cl_3NaO_2$+ [M+Na]+ m/z: 355.0030, found: 355.0035.

2,2,2-trichloroethyl (*E*)-2-(2-methylbenzyl)penta-2,4-dienoate (3p)

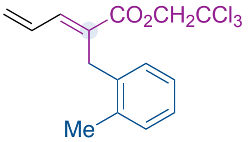

Prepared according to the general procedure B using 1,3-butadiene (492 µL 15 % v/v in hexane, 1.0 mmol), reagent **2d** (159.8 mg, 0.2 mmol), potassium (2-methylphenyl)trifluoroborate (198.0 mg, 1.0 mmol) and tetrabutylammonium hydrogensulfate (67.9 mg, 0.2 mmol). Ratio of *linear:branched* isomers was determined to be >20:1 (*linear* = 2:1, *E/Z*) using ^{1}H-NMR analysis of the crude reaction mixture. Purification by flash column chromatography on silica gel (hexane/diethyl ether 100:1) provided a mixture of 1,3-dienes as a colorless oil (21.4 mg, 32% yield).

Major isomer

^{1}H NMR (500 MHz, $CDCl_3$) δ 7.57 (d, *J* = 11.4 Hz, 1H), 7.15 (dt, *J* = 7.3, 3.7 Hz, 1H), 7.10 – 7.08 (m, 2H), 7.03 – 7.00 (m, 1H), 6.66 (ddd, *J* = 16.7, 11.4, 10.1 Hz, 1H), 5.74 (ddd, *J* = 16.7, 1.7, 0.8 Hz, 1H), 5.58 (dd, *J* = 10.0, 0.9 Hz, 1H), 4.76 (s, 2H), 3.80 (s, 2H), 2.38 (s, 3H).

^{13}C NMR (126 MHz, $CDCl_3$) δ 166.3, 142.6, 138.9, 136.9, 132.0, 130.2, 129.3, 128.5, 127.2, 126.4, 125.4, 95.2, 74.5, 29.8, 20.0.

HRMS (ESI) calculated for $C_{15}H_{15}Cl_3NaO_2^+$ $[M+Na]^+$ m/z: 355.0030, found: 355.0028.

2,2,2-trichloroethyl (*E*)-2-(2,4,6-trimethylbenzyl)penta-2,4-dienoate (3q)

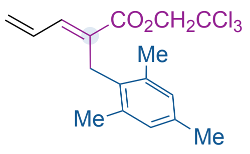

Prepared according to the general procedure B using 1,3-butadiene (492 µL 15 % v/v in hexane, 1.0 mmol), reagent **2d** (159.8 mg, 0.2 mmol), potassium (2,4,6-trimethylphenyl)trifluoroborate (226.1 mg, 1.0 mmol) and tetrabutylammonium hydrogensulfate (67.9 mg, 0.2 mmol). Ratio of *linear:branched* isomers was determined to be >20:1 (*linear* = 2.4:1, *E/Z*) using ^{1}H-NMR analysis of the crude reaction mixture. Purification by flash column chromatography on silica gel (hexane/diethyl ether 100:1) provided a mixture of 1,3-dienes as a colorless oil (29.7 mg, 41% yield).

Major isomer

^{1}H NMR (400 MHz, $CDCl_3$) δ 7.31 (ddt, *J* = 12.0, 1.5, 0.7 Hz, 1H), 6.82 (s, 2H), 6.41 (ddd, *J* = 16.7, 11.4, 10.0 Hz, 1H), 5.56 (ddd, *J* = 16.7, 1.8, 0.9 Hz, 1H), 5.42 (ddd, *J* = 10.0, 1.8, 0.8 Hz, 1H), 4.78 (s, 2H), 3.84 – 3.80 (m, 2H), 2.28 (s, 6H), 2.25 (s, 3H).

^{13}C NMR (101 MHz, $CDCl_3$) δ 166.4, 140.9, 137.1, 132.7, 131.3, 129.3, 129.1, 126.6, 124.7, 95.3, 74.4, 28.6, 20.7, 19.9.

HRMS (APCI) calculated for $C_{17}H_{20}Cl_3O_2^+$ $[M+H]^+$ m/z: 361.0523, found: 361.0522.

2,2,2-trichloroethyl (*E*)-2-cinnamylpenta-2,4-dienoate (3r)

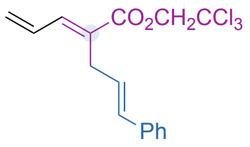

Prepared according to the general procedure B using 1,3-butadiene (492 µL 15 % v/v in hexane, 1.0 mmol), reagent **2d** (159.8 mg, 0.2 mmol), potassium *trans*-styryltrifluoroborate (210.0 mg, 1.0 mmol) and tetrabutylammonium hydrogensulfate (67.9 mg, 0.2 mmol). Ratio of *linear:branched* isomers was determined to be >20:1 (*linear* = 2:1, *E/Z*) using ^{1}H-NMR analysis of the crude reaction mixture. Purification by flash column chromatography on silica gel (hexane/diethyl ether 100:1) provided a mixture of 1,3-dienes as a colorless oil (33.2 mg, 48% yield).

Major isomer

^{1}H NMR (500 MHz, $CDCl_3$) δ 7.41 (d, *J* = 11.3 Hz, 1H), 7.33 – 7.27 (m, 4H), 7.22 – 7.17 (m, 1H), 6.77 (ddd, *J* = 16.7, 11.3, 10.0 Hz, 1H), 6.44 (dt, *J* = 15.8, 1.7 Hz, 1H), 6.22 (dt, *J* = 15.8, 6.5 Hz, 1H), 5.72 (ddd, *J* = 16.7, 1.7, 0.9 Hz, 1H), 5.61 (ddd, *J* = 10.0, 1.7, 0.8 Hz, 1H), 4.84 (s, 2H), 3.40 (dd, *J* = 6.5, 1.7 Hz, 2H).

^{13}C NMR (126 MHz, $CDCl_3$) δ 166.2, 141.8, 137.4, 131.8, 131.4, 128.8, 128.6, 127.4, 127.0, 126.7, 126.3, 95.3, 74.6, 30.3.

HRMS (ESI) calculated for $C_{16}H_{15}Cl_3NaO_2^+$ $[M+Na]^+$ m/z: 367.0030, found: 367.0028.

2,2,2-trichloroethyl (*E*)-2-allylidenehex-5-enoate (3s)

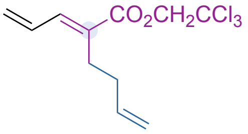

Prepared according to the general procedure B using 1,3-butadiene (492 µL 15 % v/v in hexane, 1.0 mmol), reagent **2d** (159.8 mg, 0.2 mmol), potassium allyltrifluoroborate (134.0 mg, 1.0 mmol) and tetrabutylammonium hydrogensulfate (67.9 mg, 0.2 mmol). Ratio of *linear:branched* isomers was determined to be >20:1 (*linear* = 2:1, *E/Z*) using ^{1}H-NMR analysis of the crude reaction mixture. Purification by flash column chromatography on silica gel (hexane/diethyl ether 100:1) provided a mixture of 1,3-dienes as a colorless oil (19.8 mg, 35% yield).

Major isomer

^{1}H NMR (500 MHz, $CDCl_3$) δ 7.33 (d, *J* = 11.4 Hz, 1H), 6.69 (ddd, *J* = 16.8, 11.4, 10.0 Hz, 1H), 5.82 (ddt, *J* = 17.0, 10.2, 6.8 Hz, 1H), 5.67 (ddd, *J* = 16.8, 1.7, 0.9 Hz, 1H), 5.56 (ddd, *J* = 10.0, 1.7, 0.8 Hz, 1H), 5.04 (dq, *J* = 17.0, 1.6 Hz, 1H), 4.97 (ddt, *J* = 10.2, 1.6, 1.1 Hz, 1H), 4.83 (s, 2H), 2.57 (dd, *J* = 8.8, 6.7 Hz, 2H), 2.24 (dtd, *J* = 8.8, 6.7, 1.1 Hz, 2H).

^{13}C NMR (126 MHz, $CDCl_3$) δ 166.4, 141.3, 137.6, 131.9, 130.6, 126.4, 115.6, 95.3, 74.5, 33.7, 26.8.

HRMS (APCI) calculated for $C_{11}H_{14}Cl_3O_2^+$ $[M+H]^+$ m/z: 283.0054, found: 283.0054.

2,2,2-trichloroethyl (*E*)-2-(3-phenylprop-2-yn-1-yl)penta-2,4-dienoate (3t)

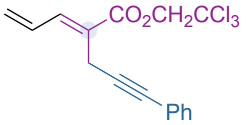

Prepared according to the general procedure B using 1,3-butadiene (492 µL 15 % v/v in hexane, 1.0 mmol), reagent **2d** (159.8 mg, 0.2 mmol), potassium (phenylethynyl)trifluoroborate (208.0 mg, 1.0 mmol) and tetrabutylammonium hydrogensulfate (67.9 mg, 0.2 mmol). Ratio of *linear:branched* isomers was determined to be >20:1 (*linear* = 2:1, *E/Z*) using ^{1}H-NMR analysis of the crude reaction mixture. Purification by flash column chromatography on silica gel (hexane/diethyl ether 100:1) provided a mixture of 1,3-dienes as a colorless oil (27.5 mg, 40% yield).

Major isomer

^{1}H NMR (500 MHz, $CDCl_3$) δ 7.41 (dt, *J* = 11.3, 0.8 Hz, 1H), 7.38 – 7.35 (m, 2H), 7.28 – 7.24 (m, 3H), 6.90 (ddd, *J* = 16.7, 11.3, 10.0 Hz, 1H), 5.76 (ddd, *J* = 16.7, 1.6, 0.9 Hz, 1H), 5.67 (ddd, *J* = 10.0, 1.6, 0.9 Hz, 1H), 4.87 (s, 2H), 3.60 (s, 2H).

^{13}C NMR (126 MHz, $CDCl_3$) δ 165.5, 142.3, 131.8, 131.7, 128.3, 128.0, 127.8, 126.1, 123.6, 95.2, 86.4, 81.1, 74.7, 17.6.

HRMS (ESI) calculated for $C_{16}H_{14}Cl_3O_2^+$ $[M+H]^+$ m/z: 343.0054, found: 343.0069.

2,2,2-trichloroethyl 3-benzyl-2-methylenepent-4-enoate (3u)

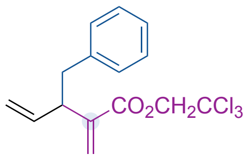

Prepared according to the general procedure B using 1,3-butadiene (492 µL 15 % v/v in hexane, 1.0 mmol), reagent **2d** (159.8 mg, 0.2 mmol), potassium benzyltrifluoroborate (198.0 mg, 1.0 mmol) and tetrabutylammonium hydrogensulfate (67.9 mg, 0.2 mmol). Ratio of *branched:linear* isomers was determined to be >20:1 using ^{1}H-NMR analysis of the crude reaction mixture. Purification by flash column chromatography on silica gel (hexane/diethyl ether 100:1) provided the title 1,4-diene as a colorless oil (46.5 mg, 70% yield).

^{1}H NMR (500 MHz, $CDCl_3$) δ 7.29 – 7.24 (m, 2H), 7.20 – 7.14 (m, 3H), 6.40 (d, *J* = 0.7 Hz, 1H), 5.89 (ddd, *J* = 17.1, 10.3, 7.7 Hz, 1H), 5.70 (s, 1H), 5.05 (dt, *J* = 6.9, 1.2 Hz, 1H), 5.04 (dt, *J* = 13.8, 1.2 Hz, 1H), 4.79 (d, *J* = 0.6 Hz, 2H), 3.64 (tdd, *J* = 8.0, 6.9, 1.1 Hz, 1H), 3.04 – 2.83 (m, 2H).

^{13}C NMR (126 MHz, $CDCl_3$) δ 165.1, 141.5, 139.6, 139.0, 129.4, 128.3, 127.6, 126.3, 116.3, 95.1, 74.5, 46.9, 40.3.

HRMS (APCI) calculated for $C_{15}H_{16}Cl_3O_2^+$ $[M+H]^+$ m/z: 333.0210, found: 333.0210.

2,2,2-trichloroethyl 3-(4-methylbenzyl)-2-methylenepent-4-enoate (3v)

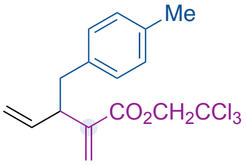

Prepared according to the general procedure B using 1,3-butadiene (492 μL 15 % v/v in hexane, 1.0 mmol), reagent **2d** (159.8 mg, 0.2 mmol), potassium (4-methylbenzyl)trifluoroborate (212.1 mg, 1.0 mmol) and tetrabutylammonium hydrogensulfate (67.9 mg, 0.2 mmol). Ratio of *branched:linear* isomers was determined to be 4:1 using ^{1}H-NMR analysis of the crude reaction mixture. Purification by flash column chromatography on silica gel (hexane/diethyl ether 100:1) provided a mixture of 1,3- and 1,4-diene as a colorless oil (42.1 mg, 61% yield).

Major isomer

^{1}H NMR (500 MHz, $CDCl_3$) δ 7.08 – 7.03 (m, 4H), 6.40 (d, *J* = 0.7 Hz, 1H), 5.88 (ddd, *J* = 17.1, 10.4, 7.6 Hz, 1H), 5.69 (m, 1H), 5.06 – 5.04 (m, 1H), 5.03 – 5.00 (m, 1H), 4.79 (d, *J* = 0.6 Hz, 2H), 3.62 (tdd, *J* = 7.9, 6.8, 1.1 Hz, 1H), 2.95 (dd, *J* = 13.7, 6.8 Hz, 1H), 2.83 (dd, *J* = 13.7, 8.1 Hz, 1H), 2.30 (s, 3H).

^{13}C NMR (126 MHz, $CDCl_3$) δ 165.2, 141.5, 139.1, 136.4, 135.7, 129.2, 129.0, 127.6, 116.2, 95.1, 74.5, 46.8, 39.8, 21.2.

HRMS (APCI) calculated for $C_{16}H_{18}Cl_3O_2^+$ $[M+H]^+$ m/z: 347.0366, found: 347.0367.

2,2,2-trichloroethyl 3-(4-(*tert*-butyl)benzyl)-2-methylenepent-4-enoate (3w)

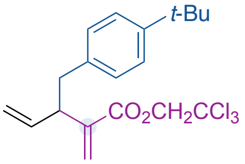

Prepared according to the general procedure B using 1,3-butadiene (492 μL 15 % v/v in hexane, 1.0 mmol), reagent **2d** (159.8 mg, 0.2 mmol), potassium (4-*tert*-butylbenzyl)trifluoroborate (254.1 mg, 1.0 mmol) and tetrabutylammonium hydrogensulfate (67.9 mg, 0.2 mmol). Ratio of *branched:linear* isomers was determined to be >20:1 using ^{1}H-NMR analysis of the crude reaction mixture. Purification by flash column chromatography on silica gel (hexane/diethyl ether 100:1) provided the title 1,4-diene as a colorless oil (31.9 mg, 41% yield).

^{1}**H NMR** (500 MHz, $CDCl_3$) δ 7.29 – 7.26 (m, 2H), 7.11 – 7.08 (m, 2H), 6.41 (d, *J* = 0.7 Hz, 1H), 5.90 (ddd, *J* = 16.7, 10.7, 7.5 Hz, 1H), 5.71 (t, *J* = 0.9 Hz, 1H), 5.07 – 5.06 (m, 1H), 5.05–5.02 (m, 1H), 4.78 (d, *J* = 1.2 Hz, 2H), 3.69–3.62 (m, 1H), 2.98 – 2.82 (m, 2H), 1.30 (s, 9H).

^{13}**C NMR** (126 MHz, $CDCl_3$) δ 165.2, 149.0, 141.6, 139.2, 136.4, 129.0, 127.6, 125.2, 116.1, 95.1, 74.4, 46.5, 39.8, 34.5, 31.5.

HRMS (APCI) calculated for $C_{19}H_{24}Cl_3O_2^+$ $[M+H]^+$ m/z: 389.0836, found: 389.0834.

2,2,2-trichloroethyl 3-([1,1'-biphenyl]-4-ylmethyl)-2-methylenepent-4-enoate (3x)

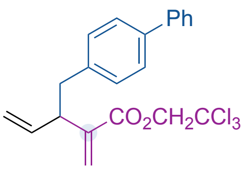

Prepared according to the general procedure B using 1,3-butadiene (492 μL 15 % v/v in hexane, 1.0 mmol), reagent **2d** (159.8 mg, 0.2 mmol), potassium ([1,1'-biphenyl]-4-ylmethyl)trifluoroborate (274.1 mg, 1.0 mmol) and tetrabutylammonium hydrogensulfate (67.9 mg, 0.2 mmol). Ratio of *branched:linear* isomers was determined to be 4:1 using ^{1}H-NMR analysis of the crude reaction mixture. Purification by flash column chromatography on silica gel (hexane/diethyl ether 100:1) provided a mixture of 1,3- and 1,4-diene as a colorless oil (37.8 mg, 46% yield).

Major isomer

^{1}**H NMR** (500 MHz, $CDCl_3$) δ 7.60 – 7.55 (m, 2H), 7.52 – 7.48 (m, 2H), 7.45 – 7.40 (m, 2H), 7.35 – 7.30 (m, 1H), 7.24 (d, *J* = 8.4 Hz, 2H), 6.43 (d, *J* = 0.7 Hz, 1H), 5.92 (ddd, *J* = 17.0, 10.4, 7.6 Hz, 1H), 5.73 (d, *J* = 0.7 Hz, 1H), 5.10 – 5.03 (m, 2H), 4.81 (s, 2H), 3.68 (q, *J* = 7.9 Hz, 1H), 3.04 (dd, *J* = 13.6, 6.9 Hz, 1H), 2.92 (dd, *J* = 13.6, 8.1 Hz, 1H).

^{13}**C NMR** (126 MHz, $CDCl_3$) δ 165.1, 141.5, 141.1, 139.2, 139.0, 138.7, 129.8, 128.9, 127.7, 127.2, 127.1, 127.1, 116.4, 95.1, 74.5, 46.7, 39.9.

HRMS (ESI) calculated for $C_{21}H_{19}Cl_3NaO_2^+$ $[M+Na]^+$ m/z: 431.0343 found: 431.0332.

2,2,2-trichloroethyl 2-methylene-3-(4-(trifluoromethoxy)benzyl)pent-4-enoate (3y)

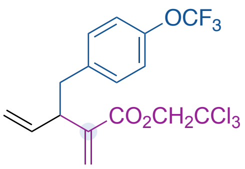

Prepared according to the general procedure B using 1,3-butadiene (492 µL 15 % v/v in hexane, 1.0 mmol), reagent **2d** (159.8 mg, 0.2 mmol), potassium (4-(trifluoromethoxy)benzyl)trifluoroborate (282.0 mg, 1.0 mmol) and tetrabutylammonium hydrogensulfate (67.9 mg, 0.2 mmol). Ratio of *branched:linear* isomers was determined to be 5:1 using ^{1}H-NMR analysis of the crude reaction mixture. Purification by flash column chromatography on silica gel (hexane/diethyl ether 100:1) provided a mixture of 1,3- and 1,4-diene as a colorless oil (57.7 mg, 69% yield).

Major isomer

^{1}H NMR (500 MHz, $CDCl_3$) δ 7.20 – 7.15 (m, 2H), 7.13 – 7.08 (m, 2H), 6.42 (d, *J* = 0.6 Hz, 1H), 5.86 (ddd, *J* = 17.1, 10.2, 7.7 Hz, 1H), 5.70 (m, 1H), 5.0 – 5.05 (m, 1H), 5.05 – 5.00 (m, 1H), 4.79 (d, *J* = 1.4 Hz, 2H), 3.64 – 3.56 (m, 1H), 2.99 (dd, *J* = 13.7, 6.8 Hz, 1H), 2.86 (dd, *J* = 13.7, 8.2 Hz, 1H).

^{13}C NMR (126 MHz, $CDCl_3$) δ 165.0, 147.8 (q, *J* = 1.86 Hz), 141.2, 138.5, 138.3, 130.6, 127.8, 120.9, 120.6 (q, *J* = 256.67 Hz), 116.7, 95.1, 74.5, 46.8, 39.6.

HRMS (APCI) calculated for $C_{16}H_{15}Cl_3F_3O_3^+$ $[M+H]^+$ m/z: 417.0033, found: 417.0031.

methyl 4-(3-((2,2,2-trichloroethoxy)carbonyl)-2-vinylbut-3-en-1-yl)benzoate (3z)

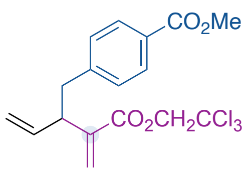

Prepared according to the general procedure B using 1,3-butadiene (492 µL 15 % v/v in hexane, 1.0 mmol), reagent **2d** (159.8 mg, 0.2 mmol), potassium methyl (4-(trifluoroboraneyl)methyl)benzoate (256.1 mg, 1.0 mmol) and tetrabutylammonium hydrogensulfate (67.9 mg, 0.2 mmol). Ratio of *branched:linear* isomers was determined to be >20:1 using ^{1}H-NMR analysis of the crude reaction mixture. Purification by flash column chromatography on silica gel (hexane/diethyl ether 100:1) provided a mixture of 1,3-diene as a colorless oil (31.9 mg, 41% yield).

^{1}H NMR (500 MHz, $CDCl_3$) δ 7.95 – 7.90 (m, 2H), 7.24 – 7.21 (m, 2H), 6.40 (d, *J* = 0.6 Hz, 1H), 5.85 (ddd, *J* = 17.1, 10.2, 7.7 Hz, 1H), 5.69 (br s, 1H), 5.04 (m, 1H), 5.03 – 4.98 (m, 1H), 4.80 (d, *J* = 2.6 Hz, 2H), 3.89 (s, 3H), 3.67 – 3.58 (m, 1H), 3.11 – 2.85 (m, 2H).

^{13}C NMR (126 MHz, $CDCl_3$) δ 167.2, 164.9, 145.0, 141.1, 138.4, 129.7, 129.4, 128.3, 127.8, 116.7, 95.0, 74.4, 52.1, 46.8, 40.2.

HRMS (APCI) calculated for $C_{17}H_{18}Cl_3O_4^+$ $[M+H]^+$ m/z: 391.0265, found: 391.0261.

2,2,2-trichloroethyl 3-([1,1'-biphenyl]-4-ylmethyl)-2-methylenepent-4-enoate (3aa)

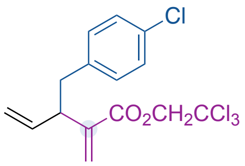

Prepared according to the general procedure B using 1,3-butadiene (492 µL 15 % v/v in hexane, 1.0 mmol), reagent **2d** (159.8 mg, 0.2 mmol), potassium (4-chlorobenzyl)trifluoroborate (233.0 mg, 1.0 mmol) and tetrabutylammonium hydrogensulfate (67.9 mg, 0.2 mmol). Ratio of *branched:linear* isomers was determined to be 5:1 using ^{1}H-NMR analysis of the crude reaction mixture. Purification by flash column chromatography on silica gel (hexane/diethyl ether 100:1) provided a miture of 1,3- and 1,4-diene as a colorless oil (23.6 mg, 32% yield).

Major isomer

^{1}H NMR (400 MHz, $CDCl_3$) δ 7.24 – 7.20 (m, 2H), 7.11 – 7.06 (m, 2H), 6.41 (d, J = 0.7 Hz, 1H), 5.85 (ddd, J = 17.1, 10.3, 7.7 Hz, 1H), 5.69 (t, J = 0.7 Hz, 1H), 5.08 – 4.97 (m, 2H), 4.80 (d, J = 1.0 Hz, 2H), 3.62 – 3.54 (m, 1H), 2.97 (dd, J = 13.7, 6.7 Hz, 1H), 2.82 (dd, J = 13.7, 8.3 Hz, 1H).

^{13}C NMR (101 MHz, $CDCl_3$) δ 165.0, 141.2, 138.5, 138.0, 132.1, 130.7, 128.5, 127.8, 116.7, 95.1, 74.5, 46.9, 39.6.

HRMS (ESI) calculated for $C_{15}H_{14}Cl_4NaO_2^+$ $[M+Na]^+$ m/z: 388.9640, found: 388.9653.

2,2,2-trichloroethyl 3-(3-methylbenzyl)-2-methylenepent-4-enoate (3ab)

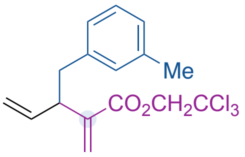

Prepared according to the general procedure B using 1,3-butadiene (492 µL 15 % v/v in hexane, 1.0 mmol), reagent **2d** (159.8 mg, 0.2 mmol), potassium (3-methylbenzyl)trifluoroborate (212.1 mg, 1.0 mmol) and tetrabutylammonium hydrogensulfate (67.9 mg, 0.2 mmol). Ratio of *branched:linear* isomers was determined to be 5:1 using ^{1}H-NMR analysis of the crude reaction mixture. Purification by flash column chromatography on silica gel (hexane/diethyl ether 100:1) provided a mixture of 1,3- and 1,4-diene as a colorless oil (27.8 mg, 40% yield).

Major isomer

^{1}H NMR (400 MHz, $CDCl_3$) δ 7.14 (t, *J* = 7.8 Hz, 1H), 6.93 – 7.01 (m, 3H), 6.40 (s, 1H), 5.88 (ddd, *J* = 17.0, 10.4, 7.4 Hz, 1H), 5.70 (s, 1H), 5.06 – 4.99 (m, 2H), 4.80 (s, 2H), 3.63 (q, *J* = 7.4 Hz, 1H), 2.96 (dd, *J* = 13.6, 6.8 Hz, 1H), 2.82 (dd, *J* = 13.6, 8.2 Hz, 1H), 2.31 (s, 3H).

^{13}C NMR (126 MHz, $CDCl_3$) δ 165.2, 141.5, 139.5, 139.1, 137.9, 130.1, 128.2, 127.6, 127.0, 126.4, 116.2, 95.2, 74.5, 46.8, 40.2, 21.6.

HRMS (ESI) calculated for $C_{16}H_{17}Cl_3NaO_2^+$ $[M+Na]^+$ m/z: 369.0186, found: 369.0198.

2,2,2-trichloroethyl 3-(2-methylbenzyl)-2-methylenepent-4-enoate (3ac)

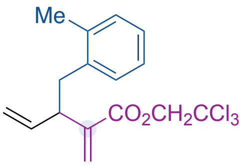

Prepared according to the general procedure B using 1,3-butadiene (492 μL 15 % v/v in hexane, 1.0 mmol), reagent **2d** (159.8 mg, 0.2 mmol), potassium (2-methylbenzyl)trifluoroborate (212.1 mg, 1.0 mmol) and tetrabutylammonium hydrogensulfate (67.9 mg, 0.2 mmol). Ratio of *branched:linear* isomers was determined to be >20:1 using ^{1}H-NMR analysis of the crude reaction mixture. Purification by flash column chromatography on silica gel (hexane/diethyl ether 100:1) provided the title 1,4-diene as a colorless oil (36.2 mg, 52% yield).

^{1}H NMR (400 MHz, $CDCl_3$) δ 7.17 – 7.05 (m, 4H), 6.42 (s, 1H), 5.93 (ddd, *J* = 17.1, 10.3, 7.6 Hz, 1H), 5.74 (t, *J* = 0.9 Hz, 1H), 5.07 – 4.97 (m, 2H), 4.77 (s, 2H), 3.64 (tdd, *J* = 8.0, 6.9, 1.0 Hz, 1H), 2.97 (dd, *J* = 13.7, 6.9 Hz, 1H), 2.90 (dd, *J* = 13.7, 8.1 Hz, 1H), 2.34 (s, 3H).

^{13}C NMR (101 MHz, $CDCl_3$) δ 165.1, 141.9, 139.1, 137.7, 136.5, 130.4, 130.2, 127.4, 126.4, 125.8, 116.1, 95.1, 74.4, 45.6, 37.8, 19.7.

HRMS (ESI) calculated for $C_{16}H_{17}Cl_3NaO_2^+$ $[M+Na]^+$ m/z: 369.0186, found: 369.0195.

2,2,2-trichloroethyl 2-methylene-3-(2,4,6-trimethylbenzyl)pent-4-enoate (3ad)

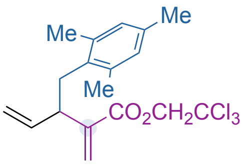

Prepared according to the general procedure B using 1,3-butadiene (492 µL 15 % v/v in hexane, 1.0 mmol), reagent **2d** (159.8 mg, 0.2 mmol), potassium (2,4,6-trimethylbenzyl)trifluoroborate (240.1 mg, 1.0 mmol) and tetrabutylammonium hydrogensulfate (67.9 mg, 0.2 mmol). Ratio of *branched:linear* isomers was determined to be >20:1 using ^{1}H-NMR analysis of the crude reaction mixture. Purification by flash column chromatography on silica gel (hexane/diethyl ether 100:1) provided the title 1,4-diene as a colorless oil (18.8 mg, 25% yield).

^{1}H NMR (500 MHz, $CDCl_3$) δ 6.81 (d, J = 1.2 Hz, 2H), 6.40 (d, J = 0.8 Hz, 1H), 5.95 (ddd, J = 17.1, 10.2, 7.8 Hz, 1H), 5.78 (t, J = 0.9 Hz, 1H), 4.97 (ddd, J = 10.2, 1.5, 0.9 Hz, 1H), 4.93 (dt, J = 16.9, 1.2 Hz, 1H), 4.72 (s, 2H), 3.55 (dddd, J = 8.7, 7.6, 6.4, 1.0 Hz, 1H), 2.96 (dd, J = 13.7, 8.8 Hz, 1H), 2.89 (dd, J = 13.7, 6.3 Hz, 1H), 2.27 (s, 6H), 2.24 (s, 3H).

^{13}C NMR (126 MHz, $CDCl_3$) δ 165.1, 142.3, 138.9, 136.7, 135.3, 133.2, 129.0, 127.0, 115.7, 95.0, 74.3, 45.6, 33.9, 20.8, 20.5.

HRMS (ESI) calculated for $C_{18}H_{21}Cl_3NaO_2^+$ $[M+Na]^+$ m/z: 397.0499, found: 397.0495.

2,2,2-trichloroethyl 2-methylene-3-(1-phenylethyl)pent-4-enoate (mixture of diastereoisomers) (3ae)

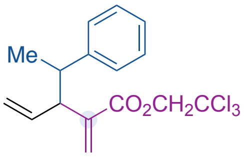

Prepared according to the general procedure B using 1,3-butadiene (492 µL 15 % v/v in hexane, 1.0 mmol), reagent **2d** (159.8 mg, 0.2 mmol), potassium (1-phenylethyl)trifluoroborate (212.1 mg, 1.0 mmol) and tetrabutylammonium hydrogensulfate (67.9 mg, 0.2 mmol). Ratio of *branched:linear* isomers was determined to be >20:1 (*dr* = 1:1) using ^{1}H-NMR analysis of the crude reaction mixture. Purification by flash column chromatography on silica gel (hexane/diethyl ether 100:1) provided a mixture of diastereoisomers as a colorless oil (32.7 mg, 47% yield).

^{1}H NMR (500 MHz, $CDCl_3$) δ 7.30 – 7.27 (m, 2H), 7.24 – 7.17 (m, 3H), 7.17 – 7.12 (m, 5H), 6.47 (d, J = 0.8 Hz, 1H), 6.20 (d, J = 0.8 Hz, 1H), 5.97 (ddd, J = 17.0, 10.1, 9.0 Hz, 1H), 5.71 (ddd, J = 17.0, 10.3, 8.4 Hz, 1H), 5.67 – 5.65 (m, 1H), 5.53 – 5.51 (m, 1H), 5.17 – 5.10 (m, 2H), 4.90 (ddd, J = 10.3, 1.6, 0.8 Hz, 1H), 4.88 (ddd, J = 17.0, 1.6, 1.0 Hz, 1H), 4.85 (s, 2H), 4.77 – 4.66 (m, 2H), 3.50 – 3.43 (m, 2H), 3.17 – 3.07 (m, 2H), 1.28 (d, J = 7.0 Hz, 3H), 1.24 (d, J = 7.0 Hz, 3H).

^{13}C NMR (126 MHz, $CDCl_3$) δ 165.3, 165.0, 145.3, 144.6, 141.2, 141.0, 138.7, 138.2, 128.4, 128.4, 128.3, 128.2, 128.1, 127.8, 126.4, 126.3, 117.2, 116.6, 95.2, 95.1, 74.5, 74.3, 53.6, 52.9, 43.0, 42.8, 20.5, 20.5.
HRMS (APCI) calculated for $C_{16}H_{18}Cl_3O_2^+$ $[M+H]^+$ m/z: 347.0367, found: 347.0369.

ethyl 3-benzyl-2-methylenepent-4-enoate (3af)

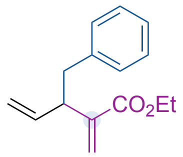

Prepared according to the general procedure B using 1,3-butadiene (492 μL 15 % v/v in hexane, 1.0 mmol), reagent **2a** (118.4 mg, 0.2 mmol), potassium benzyltrifluoroborate (198.0 mg, 1.0 mmol) and tetrabutylammonium hydrogensulfate (67.9 mg, 0.2 mmol). Ratio of *branched:linear* isomers was determined to be 10:1 using ^{1}H-NMR analysis of the crude reaction mixture. Purification by flash column chromatography on silica gel (hexane/diethyl ether 100:1) provided a mixture of 1,3- and 1,4-diene as a colorless oil (29.9 mg, 65% yield).
Major isomer
^{1}H NMR (500 MHz, $CDCl_3$) δ 7.40 – 7.38 (m, 1H), 7.31 – 7.26 (m, 2H), 7.22 – 7.17 (m, 2H), 6.24 (d, J = 1.0 Hz, 1H), 5.88 (ddd, J = 17.1, 10.3, 7.7 Hz, 1H), 5.57 (t, J = 1.1 Hz, 1H), 5.05 – 4.99 (m, 2H), 4.21 (qd, J = 7.1, 0.6 Hz, 2H), 3.63 (dddd, J = 8.6, 7.6, 6.6, 1.0 Hz, 1H), 2.98 (dd, J = 13.6, 6.6 Hz, 1H), 2.84 (dd, J = 13.6, 8.3 Hz, 1H), 1.32 (t, J = 7.1 Hz, 3H).
^{13}C NMR (126 MHz, $CDCl_3$) δ 166.8, 142.8, 139.8, 139.3, 129.4, 128.1, 126.0, 124.8, 115.7, 60.7, 46.6, 40.2, 14.2.
HRMS (ESI) calculated for $C_{15}H_{18}NaO_2^+$ $[M+Na]^+$ m/z: 253.1199, found: 253.1200.

isopropyl 3-benzyl-2-methylenepent-4-enoate (3ag)

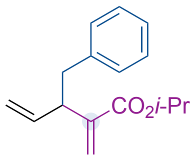

Prepared according to the general procedure B using 1,3-butadiene (492 µL 15 % v/v in hexane, 1.0 mmol), reagent **2b** (124.0 mg, 0.2 mmol), potassium benzyltrifluoroborate (198.0 mg, 1.0 mmol) and tetrabutylammonium hydrogensulfate (67.9 mg, 0.2 mmol). Ratio of *branched:linear* isomers was determined to be > 20:1 using ^{1}H-NMR analysis of the crude reaction mixture. Purification by flash column chromatography on silica gel (hexane/diethyl ether 100:1) provided the title 1,4-diene as a colorless oil (30.8 mg, 63% yield).

^{1}H NMR (500 MHz, $CDCl_3$) δ 7.30 – 7.25 (m, 2H), 7.22 – 7.16 (m, 3H), 6.20 (d, J = 1.2 Hz, 1H), 5.87 (ddd, J = 17.1, 10.3, 7.7 Hz, 1H), 5.53 (t, J = 1.1 Hz, 1H), 5.08 (p, J = 6.2 Hz, 1H), 5.05 – 4.97 (m, 2H), 3.61 (qd, J = 7.6, 1.1 Hz, 1H), 2.98 (dd, J = 13.5, 6.5 Hz, 1H), 2.82 (dd, J = 13.6, 8.4 Hz, 1H), 1.29 (dd, J = 6.2, 2.5 Hz, 6H).

^{13}C NMR (126 MHz, $CDCl_3$) δ 166.4, 143.3, 139.9, 139.5, 129.3, 128.2, 126.1, 124.6, 115.7, 68.2, 46.8, 40.4, 21.9.

HRMS (ESI) calculated for $C_{16}H_{20}NaO_2^+$ $[M+Na]^+$ m/z: 267.1356, found: 267.1361.

benzyl 3-benzyl-2-methylenepent-4-enoate (3ah)

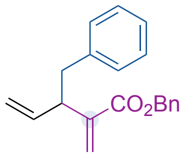

Prepared according to the general procedure B using 1,3-butadiene (492 µL 15 % v/v in hexane, 1.0 mmol), reagent **2c** (143.3 mg, 0.2 mmol), potassium benzyltrifluoroborate (198.0 mg, 1.0 mmol) and tetrabutylammonium hydrogensulfate (67.9 mg, 0.2 mmol). Ratio of *branched:linear* isomers was determined to be 5:1 using ^{1}H-NMR analysis of the crude reaction mixture. Purification by flash column chromatography on silica gel (hexane/diethyl ether 100:1) provided a mixture of 1,3- and 1,4-diene as a colorless oil (18.7 mg, 32% yield).

Major isomer

^{1}H NMR (400 MHz, $CDCl_3$) δ 7.26 (dd, J = 9.9, 2.1 Hz, 4H), 7.16 – 7.11 (m, 2H), 7.09 – 7.06 (m, 2H), 7.02 (dd, J = 6.7, 1.5 Hz, 2H), 6.17 (d, J = 0.9 Hz, 1H), 5.76 (ddd, J = 17.1, 10.3, 7.7 Hz, 1H), 5.48 (t, J = 1.0 Hz, 1H), 5.08 (s, 2H), 4.94 – 4.85 (m, 2H), 3.56 – 3.48 (m, 1H), 2.86 (dd, J = 13.8, 6.9 Hz, 1H), 2.71 (dd, J = 13.6, 8.2 Hz, 1H).

^{13}C NMR (101 MHz, $CDCl_3$) δ 166.7, 142.6, 139.8, 139.3, 136.1, 129.3, 128.6, 128.3, 128.2, 128.2, 126.1, 125.6, 115.9, 66.6, 46.7, 40.3.

HRMS (ESI) calculated for $C_{20}H_{20}NaO_2^+$ $[M+Na]^+$ m/z: 315.1356, found: 315.1342.

(1*R*,2*R*,5*R*)-2-isopropyl-5-methylcyclohexyl (*S/R*)-3-benzyl-2-methylenepent-4-enoate (3ai)

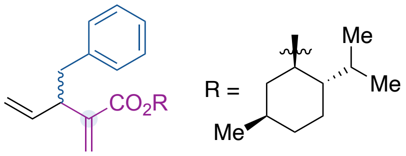

Prepared according to the general procedure B using 1,3-butadiene (492 µL 15 % v/v in hexane, 1.0 mmol), reagent **2e** (162.0 mg, 0.2 mmol), potassium benzyltrifluoroborate (198.0 mg, 1.0 mmol) and tetrabutylammonium hydrogensulfate (67.9 mg, 0.2 mmol). Ratio of *branched:linear* isomers was determined to be 10:1 (*dr* (*branched)*= 1:1) using ^{1}H-NMR analysis of the crude reaction mixture. Purification by flash column chromatography on silica gel (hexane/diethyl ether 100:1) provided a mixture of 1,3- and 1,4-diene as a colorless oil (17.0 mg, 25% yield).

Major isomer (*dr* (*branched)*= 1:1)

^{1}H NMR (400 MHz, $CDCl_3$) δ 7.28 – 7.22 (m, 2H), 7.20 – 7.13 (m, 3H), 6.17 (dd, *J* = 12.6, 1.1 Hz, 1H), 5.93 – 5.79 (m, 1H), 5.50 (dt, *J* = 7.4, 1.0 Hz, 1H), 5.03 – 4.94 (m, 2H), 4.75 (td, *J* = 10.7, 4.3 Hz, 1H), 3.59 (q, *J* = 7.9 Hz, 1H), 2.96 (ddd, *J* = 13.5, 6.7, 4.8 Hz, 1H), 2.80 (ddd, *J* = 13.6, 8.2, 2.1 Hz, 1H), 2.04 – 1.94 (m, 1H), 1.91 – 1.80 (m, 1H), 1.74 – 1.64 (m, 2H), 1.51 – 1.39 (m, 2H), 1.13 – 0.96 (m, 2H), 0.93 – 0.85 (m, 7H), 0.75 (dd, *J* = 6.9, 5.0 Hz, 3H).

^{13}C NMR (101 MHz, $CDCl_3$) δ 166.5, 143.2, 139.9, 139.5, 129.3, 128.2, 126.1, 124.6, 115.6, 74.6, 47.3, 40.9, 40.3, 34.4, 31.5, 26.6, 23.6, 22.1, 20.9, 16.4.

HRMS (ESI) calculated for $C_{23}H_{32}NaO_2^+$ $[M+Na]^+$ m/z: 363.2295, found: 363.2305.

Reaction with isoprene (4a)

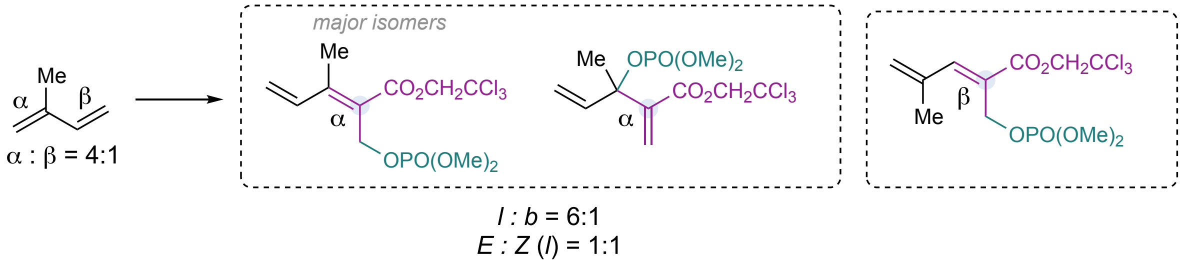

Prepared according to the general procedure B using isoprene (68 mg, 1.0 mmol), reagent **2d** (159.8 mg, 0.2 mmol) and tributyl(methyl)phosphonium dimethyl phosphate (103 mg, 0.3 mmol) in dichloromethane (2.0 mL) dropwise during 10 min. Ratio of *linear:branched* isomers was determined to be = 10:1 (*1l:2l* = 4:1, *1l* = 1:1 (*E/Z)*) using ^{1}H-NMR analysis of the crude reaction mixture.

Purification by flash column chromatography on silica gel (hexane/ethyl acetate 1:1) provided a mixture of isomers as a colorless oil (38.2 mg, 50% yield).

Major isomers

^{1}H NMR (500 MHz, $CDCl_3$) δ 7.37 (dd, J = 17.3, 11.0 Hz, 1H), 6.97 (dd, J = 17.1, 10.9 Hz, 1H), 5.77 (dd, J = 17.1, 0.8 Hz, 1H), 5.70 (dd, J = 17.3, 0.9 Hz, 1H), 5.61 (dd, J = 11.0, 0.8 Hz, 1H), 5.50 (dd, J = 11.0, 0.9 Hz, 1H), 5.01 (d, J = 5.0 Hz, 2H), 5.00 (d, J = 5.0 Hz, 2H), 4.86 (s, 2H), 4.86 (s, 2H), 3.74 (dd, J = 11.2, 2.2 Hz, 12H), 2.28 (s, 3H), 2.16 (s, 3H).

^{13}C NMR (126 MHz, $CDCl_3$) δ 165.9, 165.1, 151.3, 151.1, 149.5, 139.6, 135.6, 134.5, 127.8, 123.9, 123.2, 121.9, 95.0 (d, J = 3.2 Hz), 83.4 (d, J = 5.7 Hz), 74.7, 74.6, 64.4 (d, J = 5.3 Hz), 63.0 (d, J = 5.2 Hz), 54.5, 54.5, 17.0, 15.7.

^{31}P NMR (202 MHz, $CDCl_3$) δ 4.11, 4.00.

HRMS (ESI) calculated for $C_{11}H_{16}Cl_3NaO_6P^+$ $[M+Na]^+$ m/z: 402.9642, found: 402.9655.

Reaction with (*E*)-buta-1,3-dien-1-ylbenzene (4b)

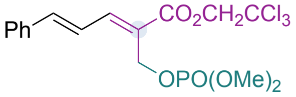

Prepared according to the general procedure B using (*E*)-buta-1,3-dien-1-ylbenzene (137 mg, 1.0 mmol), reagent **2d** (159.8 mg, 0.2 mmol) and tributyl(methyl)phosphonium dimethyl phosphate (103 mg, 0.3 mmol) in dichloromethane (2.0 mL) dropwise during 10 min. Ratio of *linear:branched* isomers was determined to be >20:1 (*linear* >20:1, *E/Z)* using ^{1}H-NMR analysis of the crude reaction mixture. Purification by flash column chromatography on silica gel (hexane/ethyl acetate 1:1) provided the title 1,3-diene as a colorless oil (35.4 mg, 40% yield).

^{1}H NMR (500 MHz, $CDCl_3$) δ 7.74 (dd, J = 11.7, 0.9 Hz, 1H), 7.59 – 7.55 (m, 2H), 7.43 – 7.33 (m, 4H), 7.09 (d, J = 15.3 Hz, 1H), 5.07 (d, J = 7.8 Hz, 2H), 4.88 (s, 2H), 3.76 (d, J = 11.1 Hz, 6H).

^{13}C NMR (126 MHz, $CDCl_3$) δ 165.0, 147.1, 144.9, 135.7, 130.1, 129.1, 128.0, 123.5 (d, J = 6.9 Hz), 122.5, 95.2, 74.6, 60.9 (d, J = 5.5 Hz), 54.5 (d, J = 6.4 Hz).

^{31}P NMR (202 MHz, $CDCl_3$) δ 4.46.

HRMS (ESI) calculated for $C_{16}H_{18}Cl_3NaO_6P^+$ $[M+Na]^+$ m/z: 464.9799, found: 464.9808.

Reaction with (*Z*)-penta-2,4-dien-2-ylbenzene (4c)

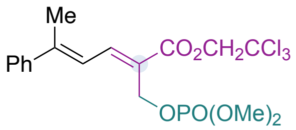

Prepared according to the general procedure B using (*Z*)-penta-2,4-dien-2-ylbenzene (144 mg, 1.0 mmol), reagent **2d** (159.8 mg, 0.2 mmol) and tributyl(methyl)phosphonium dimethyl phosphate (103 mg, 0.3 mmol) in dichloromethane (2.0 mL) dropwise during 10 min. Ratio of *linear:branched* isomers was determined to be >20:1 (*linear* >20:1, *E/Z)* using ^{1}H-NMR analysis of the crude reaction mixture. Purification by flash column chromatography on silica gel (hexane/ethyl acetate 1:1) provided the title 1,3-diene as a colorless oil (31.2 mg, 34% yield).

^{1}H NMR (500 MHz, $CDCl_3$) δ 8.11 (d, *J* = 12.3 Hz, 1H), 7.60 – 7.56 (m, 2H), 7.42 – 7.34 (m, 3H), 7.05 (dq, *J* = 12.3, 1.4 Hz, 1H), 5.06 (d, *J* = 7.4 Hz, 2H), 4.89 (s, 2H), 3.75 (d, *J* = 11.1 Hz, 6H), 2.37 (d, *J* = 1.4 Hz, 3H).

^{13}C NMR (126 MHz, $CDCl_3$) δ 165.3, 150.4, 142.5, 141.7, 129.3, 128.8, 126.4, 123.2 (d, *J* = 7.4 Hz), 120.9, 95.3, 74.5, 60.8 (d, *J* = 5.1 Hz), 54.5 (d, *J* = 6.0 Hz), 16.9.

^{31}P NMR (202 MHz, $CDCl_3$) δ 4.29.

HRMS (ESI) calculated for $C_{17}H_{20}Cl_3NaO_6P^+$ $[M+Na]^+$ m/z: 478.9955, found: 478.9965.

Reaction with 1,3-cyclohexadiene (4d)

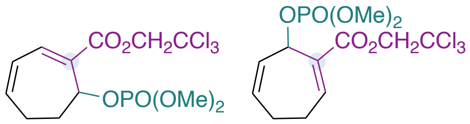

Prepared according to the general procedure B using 1,3-cyclohexadiene (80 mg, 1.0 mmol), reagent **2d** (159.8 mg, 0.2 mmol) and tributyl(methyl)phosphonium dimethyl phosphate (103 mg, 0.3 mmol) in dichloromethane (2.0 mL) dropwise during 10 min. Ratio of *1,3:1,4-diene* isomers was determined to be 6:1 using ^{1}H-NMR analysis of the crude reaction mixture. Purification by flash column chromatography on silica gel (hexane/ethyl acetate 1:1) provided a mixture of 1,3- and 1,4-diene as a colorless oil (22.0 mg, 28% yield).

Major isomer

^{1}H NMR (500 MHz, Acetone-*d$_6$*) δ 7.44 (d, *J* = 7.6 Hz, 1H), 6.57 (ddd, *J* = 11.0, 7.3, 3.1 Hz, 1H), 6.13 (ddd, *J* = 11.0, 7.6, 3.0 Hz, 1H), 5.79 (t, *J* = 6.7 Hz, 1H), 5.03 (d, *J* = 12.2 Hz, 1H), 4.95 (d, *J* =

12.2 Hz, 1H), 3.71 (dd, J = 13.5, 11.2 Hz, 6H), 2.72 – 2.62 (m, 1H), 2.60 – 2.51 (m, 1H), 2.49 – 2.40 (m, 1H), 1.78 – 1.70 (m, 1H).

^{13}C NMR (126 MHz, Acetone-d_6) δ 165.9, 147.4, 140.0, 131.3 (d, J = 7.4 Hz), 123.2, 96.3, 75.0, 73.4 (d, J = 5.1 Hz), 54.7 (d, J = 6.1 Hz), 54.5 (d, J = 6.0 Hz), 29.1 (d, J = 2.8 Hz), 25.8.

^{31}P NMR (202 MHz, Acetone-d_6) δ 2.83.

HRMS (ESI) calculated for $C_{12}H_{16}Cl_3NaO_6P^+$ $[M+Na]^+$ m/z: 414.9642, found: 414.9631.

General procedure C:[2]

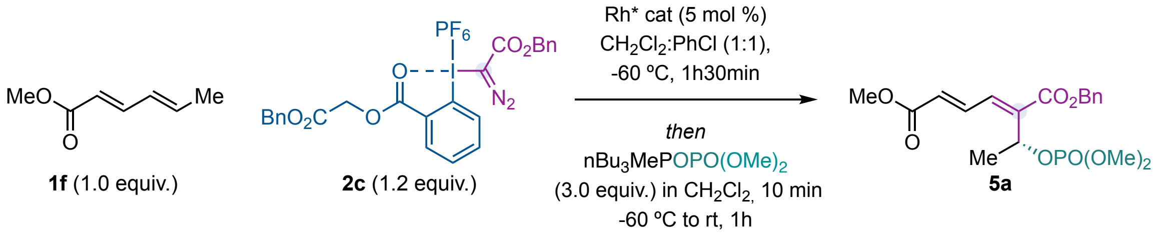

To a 10 mL oven-dried reaction tube equipped with a stirring bar was added the corresponding enantiopure dirhodium catalyst (0.005 mmol, 5.0 mol%). The tube was sealed before being evacuated and backfilled with argon three times. 1,3-diene **1f** (12.6 mg, 0.1 mmol, 1.0 equiv.) and a degassed mixture of dichloromethane and chlorobenzene (0.5 mL, 1:1) were added and the resulting mixture was cooled at -60 °C. Then, a solution of reagent **2c** (86 mg, 0.12 mmol, 1.2 equiv.) in a degassed mixture of dichloromethane and chlorobenzene (1.0 mL, 1:1) was added dropwise during 30 min using a syringe pump and after stirred for 60 min at -60 °C. After this, tributyl(methyl)phosphonium dimethyl phosphate (102.7 mg, 0.3 mmol, 3.0 equiv.) in dichloromethane (1.0 mL) was added dropwise during 10 min. Then the resulting reaction mixture was allowed to warm to room temperature during 1 h followed by the removal of solvent under reduced pressure. The crude residue was purified by column chromatography to yield the corresponding chiral allylic phosphate **5a**. Racemic compounds were prepared using $Rh_2(esp)_2$ as a catalyst.

Table S3. Optimization table for enantiopure dirhodium catalysts

Entry	$Rh_2(L^*)_4$	Yield **5a**[a]	Ratio *E:Z*[b]	*e.r.*
1	$Rh_2(S\text{-PTTL})_4$	11%	-	-
2	$Rh_2(S\text{-DOSP})_4$	n.d.	-	-
3	$Rh_2(S\text{-NTTL})_4(AcOEt)_2$	86%[c]	>20:1	92.5:7.5
4	$Rh_2(S\text{-NPTTL})_4$	10%	-	-
5	$Rh_2(R\text{-PTAD})_4$	6%	-	-
6	$Rh_2(S\text{-BLBE})_4$	n.d.	-	-
7	$Rh_2(R\text{-BTPCP})_4$	n.d.	-	-

[a]Yields were reported on the basis of ^{1}H-NMR analysis using dibromomethane as internal standard. [b]Refers to the ratio of diastereoisomers and were reported on the basis of ^{1}H-NMR analysis of the crude. [c]Isolated yield. n.d. = not detected.

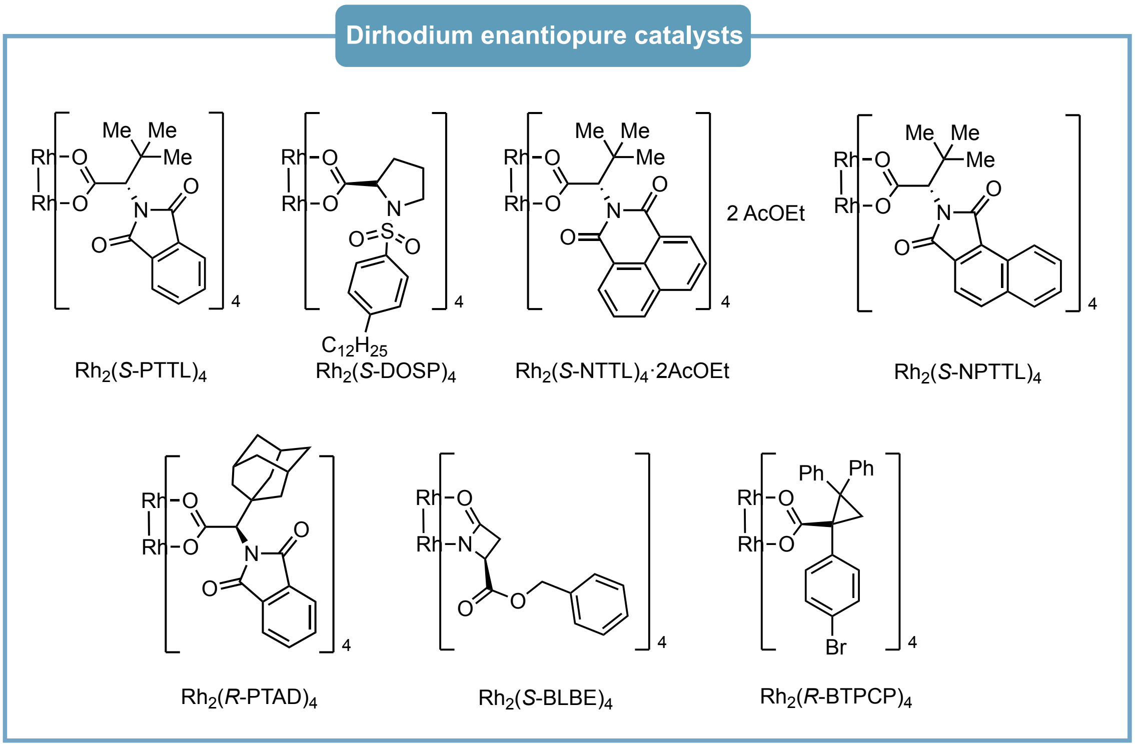

General procedure D:

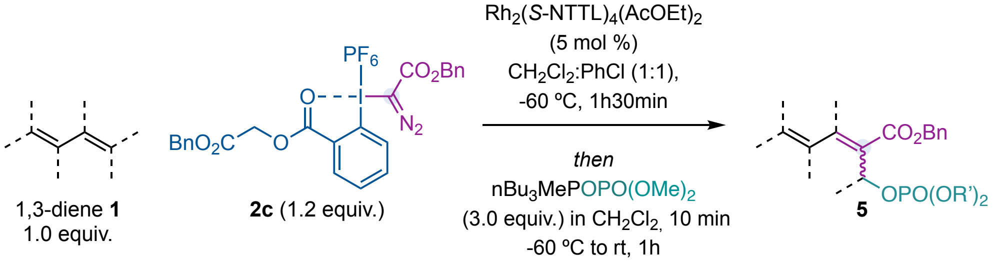

To a 10 mL oven-dried reaction tube equipped with a stirring bar was added Rh_2(*S*-NTTL)$_4$(AcOEt)$_2$ (16.2 mg, 0.02 mmol, 5.0 mol%). The tube was sealed before being evacuated and backfilled with argon three times. The corresponding 1,3-diene **1** (0.2 mmol, 1.0 equiv.) and a degassed mixture of dichloromethane and chlorobenzene (1.0 mL, 1:1) were added and the resulting mixture was cooled at -60 ºC. Then, a solution of reagent **2c** (172 mg, 0.24 mmol, 1.2 equiv.) in a degassed mixture of dichloromethane and chlorobenzene (2.0 mL, 1:1) was added dropwise during 30 min using a syringe pump and after stirred for 60 min at -60 ºC. After this, the corresponding phosphate nucleophile (0.6 mmol, 3.0 equiv.) in dichloromethane (2.0 mL) was added dropwise during 10 min. Then the resulting reaction mixture was allowed to warm to room temperature during 1 h followed by the removal of solvent under reduced pressure. The crude residue was purified by column chromatography to yield the corresponding chiral compounds **5**. Racemic compounds were prepared using $Rh_2(esp)_2$ as a catalyst.

1-benzyl 6-methyl (2*E*,4*E*)-2-((*R*)-1-((dimethoxyphosphoryl)oxy)ethyl)hexa-2,4-dienedioate (5a)

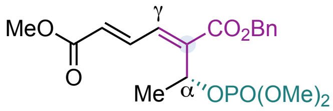

Prepared according to the general procedure D using methyl sorbate (25.2 mg, 0.2 mmol) and tributyl(methyl)phosphonium dimethyl phosphate (205.4 mg, 0.6 mmol). Ratio of $\alpha:\gamma$ isomers was determined to be >20:1 (γ >20:1, *E/Z)* using ^{1}H-NMR analysis of the crude reaction mixture. Purification by flash column chromatography (hexane/ethyl acetate 1:1) provided the title chiral 1,3-diene as colourless oil (68.6 mg, 86% yield). Enantiomeric ratio was determined to be 92.5:7.5 by SFC analysis on a chiral stationary phase (IC-3, 1 mL/min, 10% ethanol, λ = 270 nm, t_r(major) = 1.786 min, t_r(minor) = 2.106 min).

$[\alpha]_D^{26}$ = -18.6 (c = 0.05, $CHCl_3$).

^{1}H NMR (500 MHz, $CDCl_3$) δ 7.89 (dd, J = 15.3, 12.1 Hz, 1H), 7.40 – 7.33 (m, 5H), 7.29 (dd, J = 12.2, 0.9 Hz, 1H), 6.18 (dd, J = 15.3, 0.9 Hz, 1H), 5.66 (dq, J = 7.7, 6.6 Hz, 1H), 5.26 (d, J = 12.4 Hz, 1H), 5.23 (d, J = 12.4 Hz, 1H), 3.78 (s, 3H), 3.73 (d, J = 11.2 Hz, 3H), 3.66 (d, J = 11.2 Hz, 3H), 1.63 (d, J = 7.2 Hz, 3H).

^{13}C NMR (126 MHz, $CDCl_3$) δ 166.3, 165.3, 137.5, 137.4, 135.6, 129.6, 128.8, 128.6, 128.5, 71.7 (d, J = 5.3 Hz), 67.3, 54.5, 52.1, 22.7 (d, J = 5.5 Hz).

^{31}P NMR (202 MHz, $CDCl_3$) δ 3.12.

HRMS: (ESI) calculated for $C_{18}H_{23}NaO_8P$ $[M+Na]^+$ m/z: 421.1023, found: 421.1025.

5a

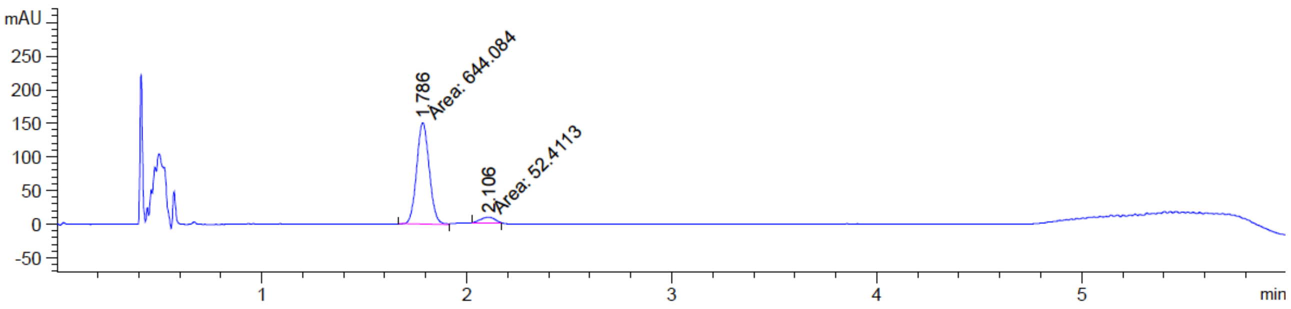

Peak #	RetTime [min]	Type	Width [min]	Area [mAU*s]	Height [mAU]	Area %
1	1.786	MM	0.0712	644.08435	150.78558	92.4750
2	2.106	MM	0.0872	52.41127	10.01234	7.5250

***racemic*-5a**

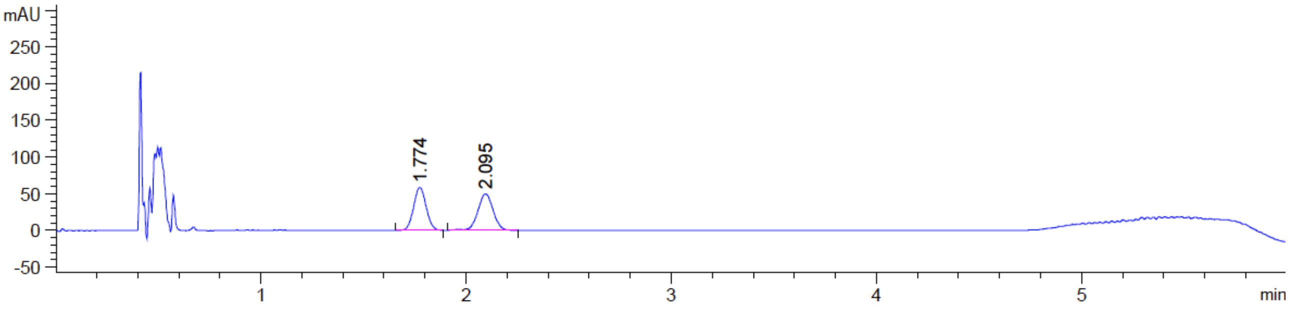

Peak #	RetTime [min]	Type	Width [min]	Area [mAU*s]	Height [mAU]	Area %
1	1.774	VB R	0.0674	250.35439	58.48584	49.1799
2	2.095	VV R	0.0823	258.70401	49.67060	50.8201

1-benzyl 6-ethyl (2*E*,4*E*)-2-((*R*)-1-((di-*tert*-butoxyphosphoryl)oxy)ethyl)hexa-2,4-dienedioate (5b)

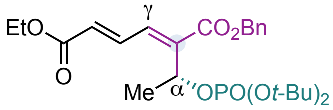

Prepared according to the general procedure D using ethyl sorbate (28.0 mg, 0.2 mmol) and tetrabutylammonium di-*tert*-butyl phosphate (271 mg, 0.6 mmol). Ratio of $\alpha{:}\gamma$ isomers was determined to be = 10:1 (γ >20:1, *E/Z)* using ^{1}H-NMR analysis of the crude reaction mixture. Purification by flash column chromatography (hexane/ethyl acetate 2:1) provided the title chiral 1,3-diene as colourless oil (49.6 mg, 50% yield). Enantiomeric ratio was determined to be 97.5:2.5 by SFC analysis on a chiral stationary phase (Whelk-O, 2 mL/min, 3% methanol/CO_2, 140 Bar, λ = 269 nm, t_r(major) = 11.12 min, t_r(minor) = 10.32 min).

$[\alpha]_D^{23}$ = +40.7 (c = 0.145, $CHCl_3$).

^{1}H NMR (500 MHz, $CDCl_3$) δ 7.90 (dd, *J* = 15.3, 12.1 Hz, 1H), 7.39 – 7.34 (m, 5H), 7.24 (d, *J* = 15.3 Hz, 1H), 6.13 (dd, *J* = 15.3, 0.9 Hz, 1H), 5.58 (dq, *J* = 8.5, 6.6 Hz, 1H), 5.23 (d, *J* = 1.1 Hz, 2H), 4.22 (q, *J* = 7.1 Hz, 2H), 1.59 (d, *J* = 6.6 Hz, 3H), 1.44 (d, *J* = 0.6 Hz, 9H), 1.40 (d, *J* = 0.6 Hz, 9H), 1.29 (t, *J* = 7.1 Hz, 3H).

^{13}C NMR (126 MHz, $CDCl_3$) δ 165.9, 165.6, 138.0 (d, *J* = 4.4 Hz), 137.8, 137.1, 135.7, 129.5, 128.7, 128.6, 128.4, 128.3, 82.6 (dd, *J* = 16.5, 7.4 Hz), 70.3 (d, *J* = 5.6 Hz), 67.1, 60.9, 29.9 (dd, *J* = 8.4, 4.3 Hz), 22.7 (d, *J* = 5.6 Hz), 14.4.

^{31}P NMR (162 MHz, $CDCl_3$) δ -7.56.

HRMS: (ESI) calculated for $C_{25}H_{37}NaO_8P$ $[M+Na]^+$ m/z: 519.2195, found: 519.2199.

5b

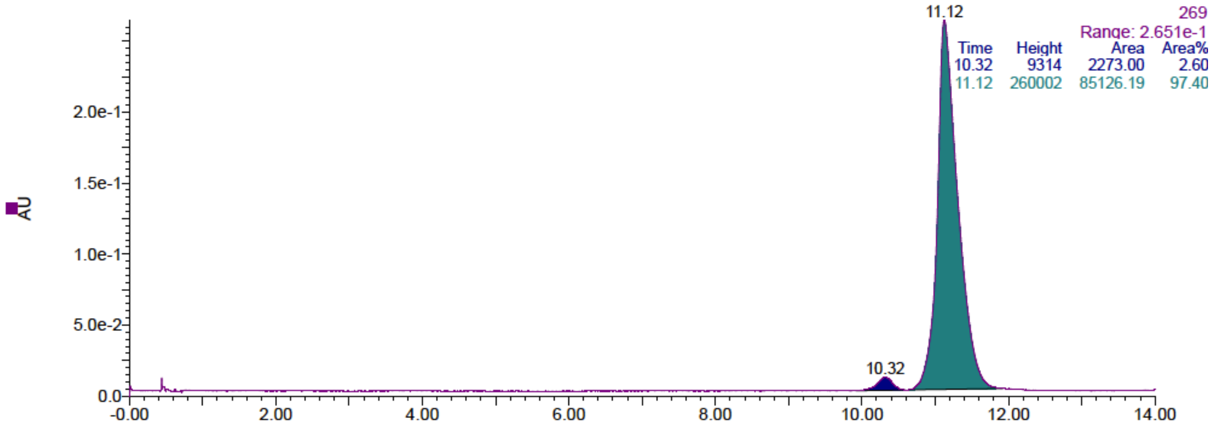

racemic-5b

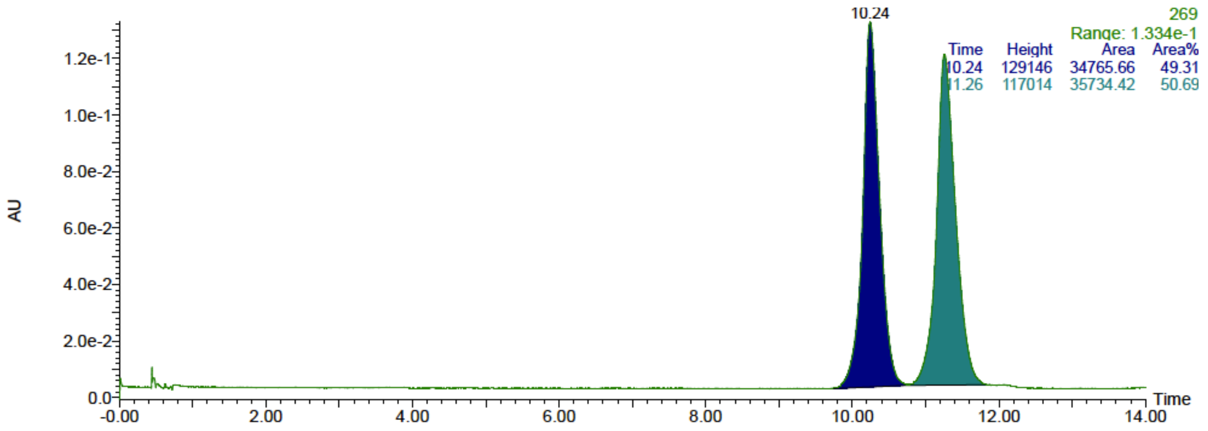

dibenzyl (2*E*,4*E*)-2-((*R*)-1-((dimethoxyphosphoryl)oxy)ethyl)hexa-2,4-dienedioate (5c)

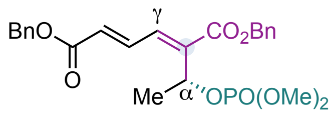

Prepared according to the general procedure D using benzyl sorbate (40.4 mg, 0.2 mmol) and tributyl(methyl)phosphonium dimethyl phosphate (205.4 mg, 0.6 mmol). Ratio of *α:γ* isomers was determined to be >20:1 (*γ* >20:1, *E/Z)* using ^{1}H-NMR analysis of the crude reaction mixture. Purification by flash column chromatography (hexane/ethyl acetate 1:1) provided the title chiral 1,3-diene as a colourless oil (34.2 mg, 36% yield). Enantiomeric ratio was determined to be 93:7 by SFC analysis on a chiral stationary phase (IC-3, 1 mL/min, 25% methanol, λ = 270 nm, t_r(major) = 1.731 min, t_r(minor) = 1.920 min).

$[\alpha]_D^{26}$ = +23.8 (c = 0.06, $CHCl_3$).

^{1}H NMR (500 MHz, $CDCl_3$) δ 7.94 (dd, *J* = 15.3, 12.2 Hz, 1H), 7.39 – 7.32 (m, 10H), 7.29 (dd, *J* = 12.2, 0.8 Hz, 1H), 6.22 (dd, *J* = 15.3, 0.8 Hz, 1H), 5.66 (dq, *J* = 7.7, 6.6 Hz, 1H), 5.28 – 5.21 (m, 3H), 5.19 (d, *J* = 12.4 Hz, 1H), 3.69 (d, *J* = 11.2 Hz, 3H), 3.63 (d, *J* = 11.2 Hz, 3H), 1.62 (d, *J* = 6.6 Hz, 3H).

^{13}C NMR (126 MHz, $CDCl_3$) δ 165.6, 165.2, 137.7, 137.4, 135.6 (d, *J* = 22.2 Hz), 129.5, 128.6 (d, *J* = 4.4 Hz), 128.4 (d, *J* = 6.5 Hz), 128.3 (d, *J* = 4.5 Hz), 71.6 (d, *J* = 5.0 Hz), 67.2, 66.7, 54.3, 22.6 (d, *J* = 5.2 Hz).

^{31}P NMR (202 MHz, $CDCl_3$) δ 3.07.

HRMS: (ESI) calculated for $C_{24}H_{28}O_8P$ $[M+H]^+$ m/z: 475.1516, found: 475.1511.

5c

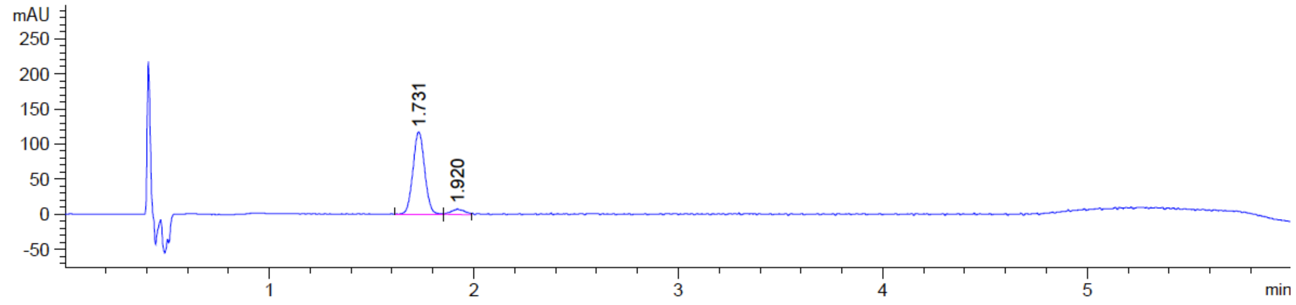

Peak #	RetTime [min]	Type	Width [min]	Area [mAU*s]	Height [mAU]	Area %
1	1.731	VV R	0.0622	463.45938	117.14707	92.9860
2	1.920	BV	0.0536	34.95920	8.05916	7.0140

***racemic*-5c**

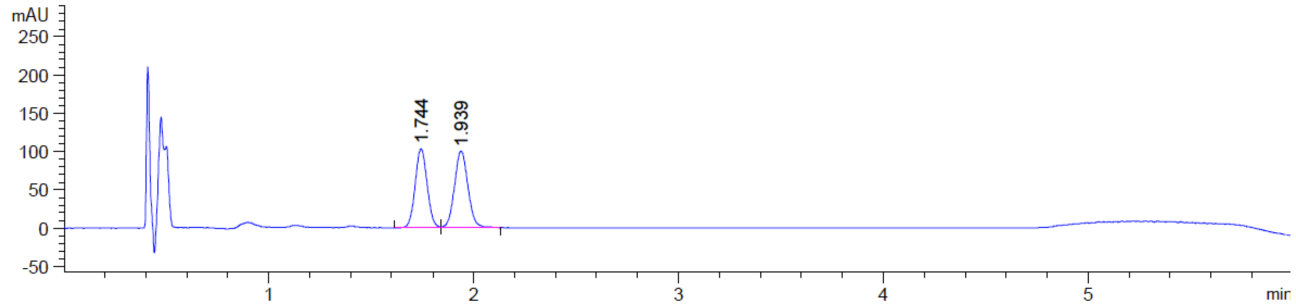

Peak #	RetTime [min]	Type	Width [min]	Area [mAU*s]	Height [mAU]	Area %
1	1.744	VV R	0.0634	419.26605	103.21999	47.7871
2	1.939	VV R	0.0705	458.09647	99.96250	52.2129

benzyl (2*E*,4*E*)-2-((*R*)-1-((dimethoxyphosphoryl)oxy)ethyl)-5-(4-(trifluoromethyl)phenyl)penta-2,4-dienoate (5d)

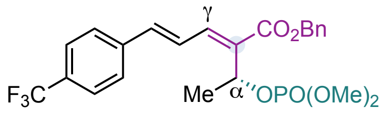

Prepared according to the general procedure D using 1-((1*E*,3*E*)-penta-1,3-dien-1-yl)-4-(trifluoromethyl)benzene (42.4 mg, 0.2 mmol) and tributyl(methyl)phosphonium dimethyl phosphate (205.4 mg, 0.6 mmol. Ratio of α:γ isomers was determined to be >20:1 (γ >20:1, *E/Z)* using ^{1}H-NMR analysis of the crude reaction mixture. Purification by flash column chromatography (hexane/ethyl acetate 1:1) provided the title chiral 1,3-diene as a colourless oil (24.2 mg, 25% yield). Enantiomeric ratio was determined to be 70.5:29.5 by SFC analysis on a chiral stationary phase (IC-3, 1 mL/min, 10% methanol, λ = 270 nm, t_r(major) = 1.735 min, t_r(minor) = 1.987 min).

$[\alpha]_D^{26}$ = +9.6 (c = 0.12, $CHCl_3$).

^{1}H NMR (500 MHz, $CDCl_3$) δ 7.62 – 7.58 (m, 4H), 7.56 – 7.51 (m, 1H), 7.47 (d, J = 11.9 Hz, 1H), 7.41 – 7.36 (m, 5H), 6.92 (d, J = 15.1 Hz, 1H), 5.73 (dq, J = 7.8, 6.6 Hz, 1H), 5.26 (d, J = 1.8 Hz, 2H), 3.73 (d, J = 11.2 Hz, 3H), 3.65 (d, J = 11.2 Hz, 3H), 1.67 (dd, J = 6.6, 0.7 Hz, 3H).

^{13}C NMR (126 MHz, $CDCl_3$) δ 165.7, 140.8, 140.4, 139.4, 135.9, 131.3 (d, J = 4.1 Hz), 128.6, 128.3, 128.3, 127.6, 125.8 (q, J = 3.8 Hz), 125.2, 71.8 (d, J = 5.4 Hz), 66.8, 54.2 (d, J = 6.3 Hz), 22.3 (d, J = 5.8 Hz).

^{19}F NMR (471 MHz, $CDCl_3$) δ -62.7.

^{31}P NMR (202 MHz, $CDCl_3$) δ 3.49.

HRMS: (ESI) calculated for $C_{23}H_{24}F_3NaO_6P$ $[M+Na]^+$ m/z: 507.1206, found: 507.1210.

5d

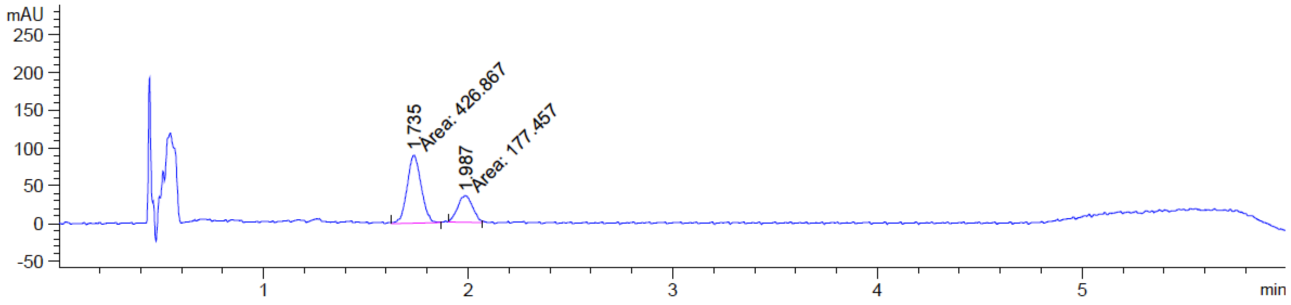

Peak #	RetTime [min]	Type	Width [min]	Area [mAU*s]	Height [mAU]	Area %
1	1.735	MM	0.0791	426.86737	89.90204	70.6354
2	1.987	MM	0.0844	177.45721	35.06144	29.3646

racemic-5d

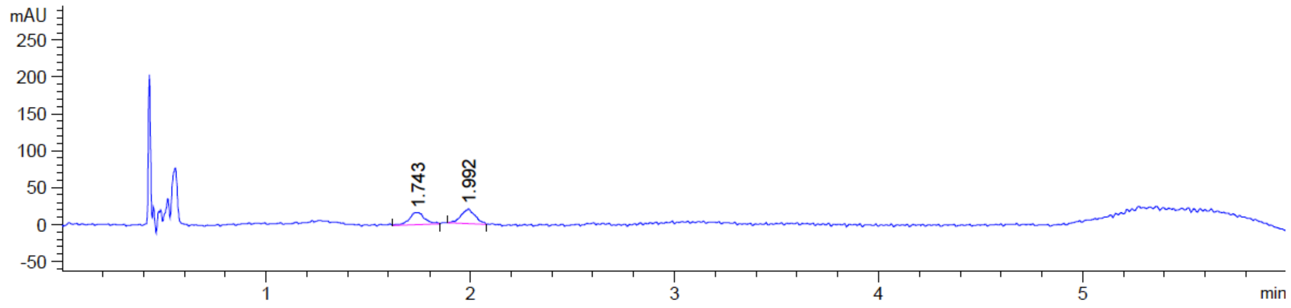

Peak #	RetTime [min]	Type	Width [min]	Area [mAU*s]	Height [mAU]	Area %
1	1.743	BV R	0.0657	87.47940	16.54936	47.5598
2	1.992	VB R	0.0591	96.45603	20.23417	52.4402

4. Low temperature ^{1}H NMR studies and detection of cyclopropyl-I(III) intermediates

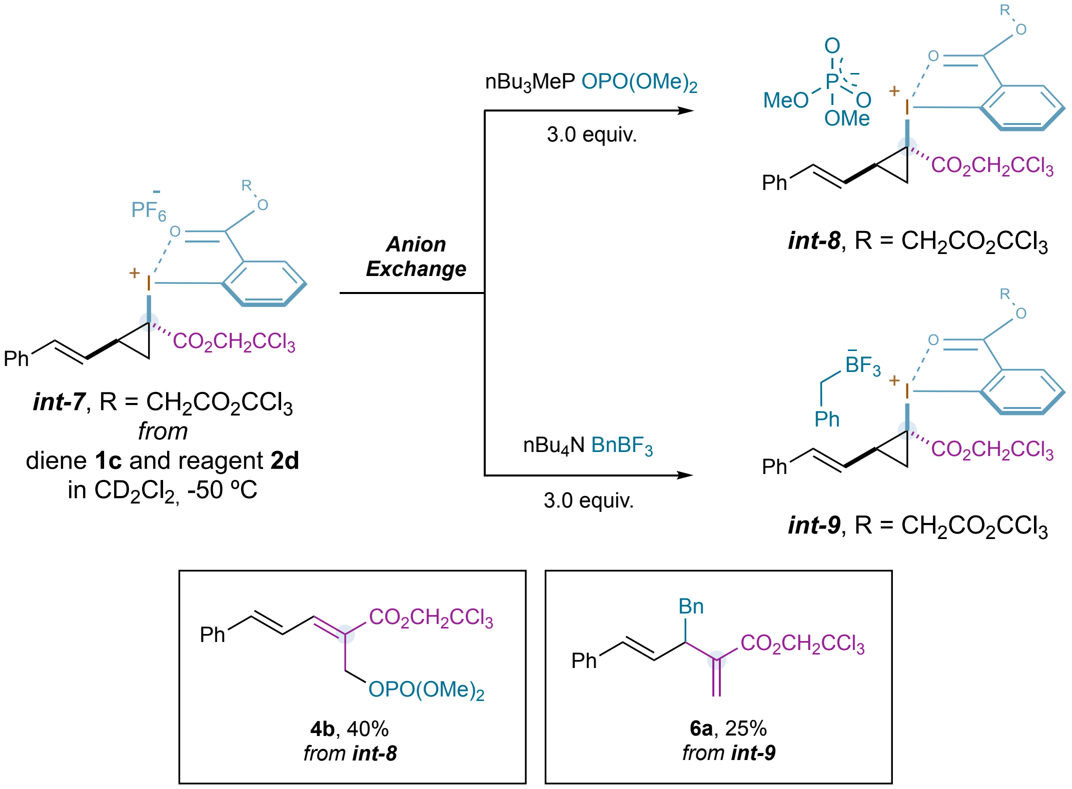

To a 10 mL reaction tube equipped with a stirring bar was added $Rh_2(esp)_2$ (0.8 mg, 0.001 mmol, 1 mol%) and (*E*)-buta-1,3-dien-1-ylbenzene **1c** (26.0 mg, 0.2 mmol). The tube was sealed before being evacuated and backfilled with argon three times. Deuterated dichloromethane (0.5 mL) was added and the resulting mixture was cooled at -50 ºC. Then, a solution of reagent **2d** (79.9 mg, 0.1 mmol, 1.0 equiv.) in deuterated dichloromethane (1.0 mL) was added dropwise during 1 h using a syringe pump. The reaction was transferred (0.6 mL) to a previously backfilled with argon NMR tube at -50 ºC and ^{1}H NMR of intermediate ***int-7*** was measured. After this, a solution of tributyl(methyl)phosphonium dimethyl phosphate (102.7 mg, 0.3 mmol, 3 equiv.) or tetrabutylammonium benzyltrifluoroborate (120.4 mg, 0.3 mmol, 3 equiv.) in deuterated dichloromethane (1.0 mL) was added dropwise during 10 minutes. The reaction was then transferred (0.6 mL) to a previously backfilled with argon NMR tube at -50 ºC and ^{1}H-NMR of species ***int-8*** and ***int-9*** were measured. The same NMR tubes were then subjected to a temperature ramp where ^{1}H-NMR were measured every 10 ºC until reaching room temperature.

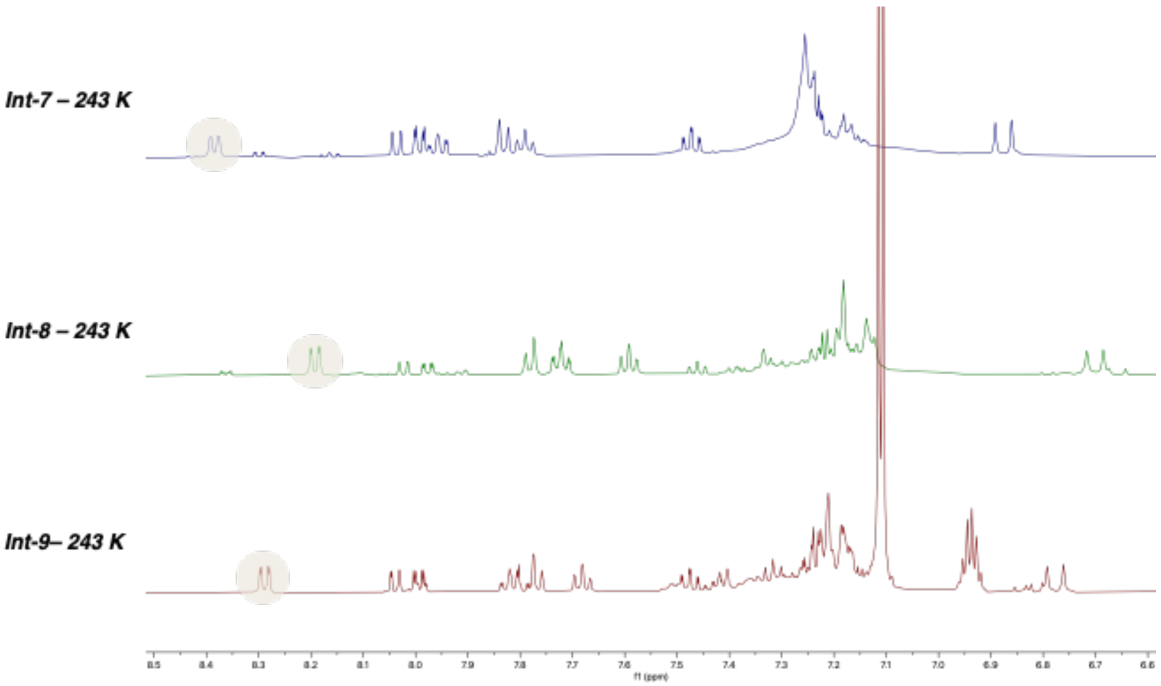

Figure 1. ^{1}H-NMR expansion of ***int-7***, ***int-8*** and ***int-9*** at -30 ºC. Highlited the aromatic C–H chemical shift displacement.

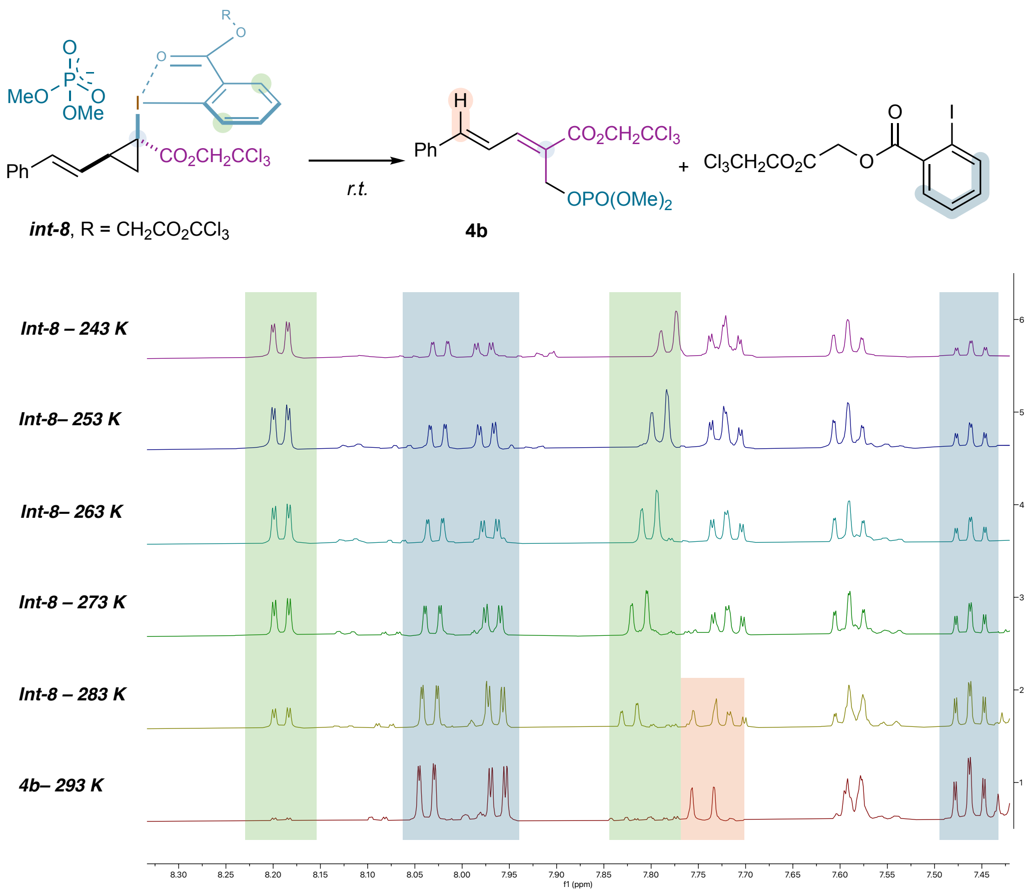

Figure 2. ^{1}H-NMR expansion of ***int-8*** evolution to **4b** with temperature increase.

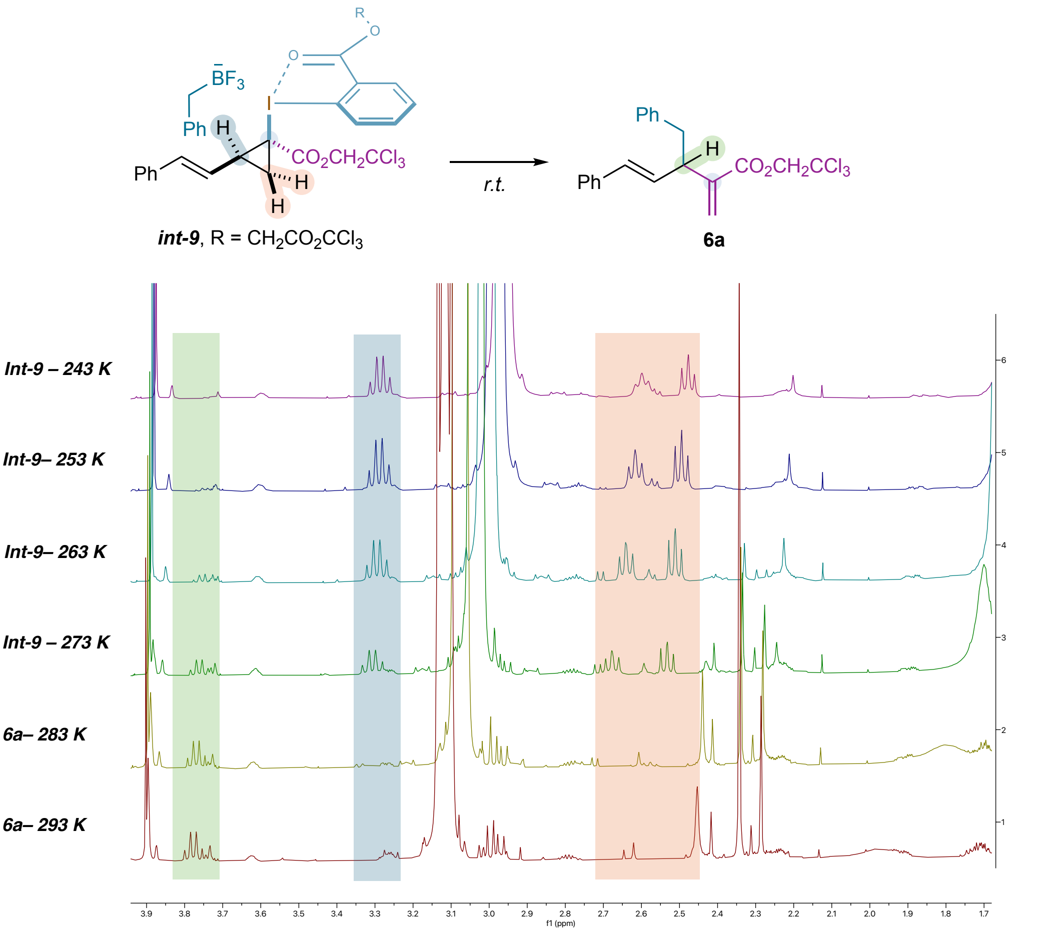

Figure 3. ^{1}H-NMR expansion of ***int-9*** evolution to **6a** with temperature increase.

int-7

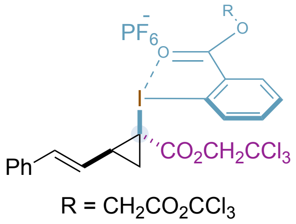

^{1}H NMR (500 MHz, CD_2Cl_2) δ 8.39 (dd, J = 7.7, 1.7 Hz, 1H), 7.97 – 7.93 (m, 1H), 7.85 – 7.83 (m, 1H), 7.79 (td, J = 7.5, 0.9 Hz, 1H), 6.88 (dd, J = 15.7, 1.0 Hz, 1H), 6.07 (dd, J = 15.6, 7.4 Hz, 1H), 5.16 (s, 2H), 4.87 (s, 2H), 4.85 (d, J = 2.6 Hz, 2H), 3.40 (td, J = 8.8, 7.0 Hz, 1H), 2.96 (t, J = 8.6 Hz, 1H), 2.57 (dd, J = 8.8, 7.5 Hz, 1H).

^{13}C NMR (126 MHz, CD_2Cl_2) δ 173.7, 141.2, 138.9, 134.1, 132.8, 132.0, 129.8, 127.5, 123.9, 109.1, 94.0, 93.7, 74.0, 73.9, 63.0, 34.3, 24.3.

^{1}H-^{1}H NOESY, ^{1}H-^{13}C HSQC, ^{1}H-^{13}C HMBC spectra were measured.

2,2,2-trichloroethyl (*E*)-3-benzyl-2-methylene-5-phenylpent-4-enoate (6a)

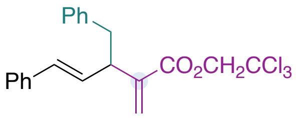

^{1}H NMR (500 MHz, $CDCl_3$) δ 7.30 – 7.25 (m, 6H), 7.22 – 7.18 (m, 4H), 6.42 (s, 1H), 6.37 (d, *J* = 15.9 Hz, 1H), 6.27 (dd, *J* = 15.9, 7.9 Hz, 1H), 5.75 (t, *J* = 0.9 Hz, 1H), 4.82 (d, *J* = 0.7 Hz, 2H), 3.78 (q, *J* = 7.9 Hz, 1H), 3.09 (dd, *J* = 13.6, 7.0 Hz, 1H), 2.98 (dd, *J* = 13.6, 8.0 Hz, 1H).

^{13}C NMR (101 MHz, $CDCl_3$) δ 165.1, 141.6, 139.5, 137.3, 131.5, 130.8, 129.4, 128.6, 128.4, 127.7, 127.5, 126.4, 126.4, 95.1, 74.5, 46.5, 40.7.

HRMS: (ESI) calculated for $C_{21}H_{19}Cl_3NaO_2$ $[M+Na]^+$ m/z: 431.0343, found: 431.0331.

5. References

1 T. D. Nelson, Z. J. Song, A. S. Thompson, M. Zhao, A. DeMarco, R. A. Reamer, M. F. Huntington, E. J. J. Grabowski, P. J. Reider, *Tetrahedron Letters,* 2000, **41**, 1877–1881.

2 W. J. Teo, J. Esteve Guasch, L. Jiang, B. Li, M. G. Suero, *J. Am. Chem. Soc.,* 2024, **146**, 21837–21846.

3 D. J. Miller, F. Yu, N. J. Young, R. K. Allemann, *Org. Biomol. Chem.,* 2007, **5**, 3287.

4 S. G. Davies, A. M. Fletcher, P. M. Roberts, A. D. Smith, *Tetrahedron,* 2009, **65**, 10192–10213.

5 A. Mori, Y. Miyakawa, E. Ohashi, T. Haga, T. Maegawa, H. Sajiki, *Org. Lett.,* 2006, **8**, 3279–3281.

6 Osmo E. O. Hormi, *Synthetic Communications,* 1986, **16**, 1003–1013.

7 Z. Wang, L. Jiang, P. Sarró, M. G. Suero, *J. Am. Chem. Soc.,* 2019, **141**, 15509–15514.

8 L. Fu, J. D. Mighion, E. A. Voight, H. M. L. Davies, *Chemistry A European J,* 2017, **23**, 3272–3275.

9 A. R. Mazzotti, M. G. Campbell, P. Tang, J. M. Murphy, T. Ritter, *J. Am. Chem. Soc.,* 2013, **135**, 14012–14015.

10 G. A. Molander, B. Biolatto, *J. Org. Chem.,* 2003, **68**, 4302–4314.

11 M. Li, C. Wang, H. Ge, *Org. Lett.,* 2011, **13**, 2062–2064.

12 M. Ferger, S. M. Berger, F. Rauch, M. Schönitz, J. Rühe, J. Krebs, A. Friedrich, T. B. Marder, *Chemistry A European J,* 2021, **27**, 9094–9101.

13 Y. Yasu, T. Koike, M. Akita, *Adv. Synth. Catal.,* 2012, **354**, 3414–3420.

14 C. Li, R. Ding, H.-Y. Guo, S. Xia, L. Shu, P.-L. Wang, H. Li, *Green Chem.,* 2022, **24**, 7883–7888.

15 W. Liu, P. Liu, L. Lv, C. Li, *Angew. Chem., Int. Ed.,* 2018, **130**, 13687–13691.

16 J. Luo, B. Hu, W. Wu, M. Hu, T. L. Liu, *Angew. Chem., Int. Ed.,* 2021, **60**, 6107–6116.

17 J. C. Tellis, D. N. Primer, G. A. Molander, *Science,* 2014, **345**, 433–436.

6. NMR spectra

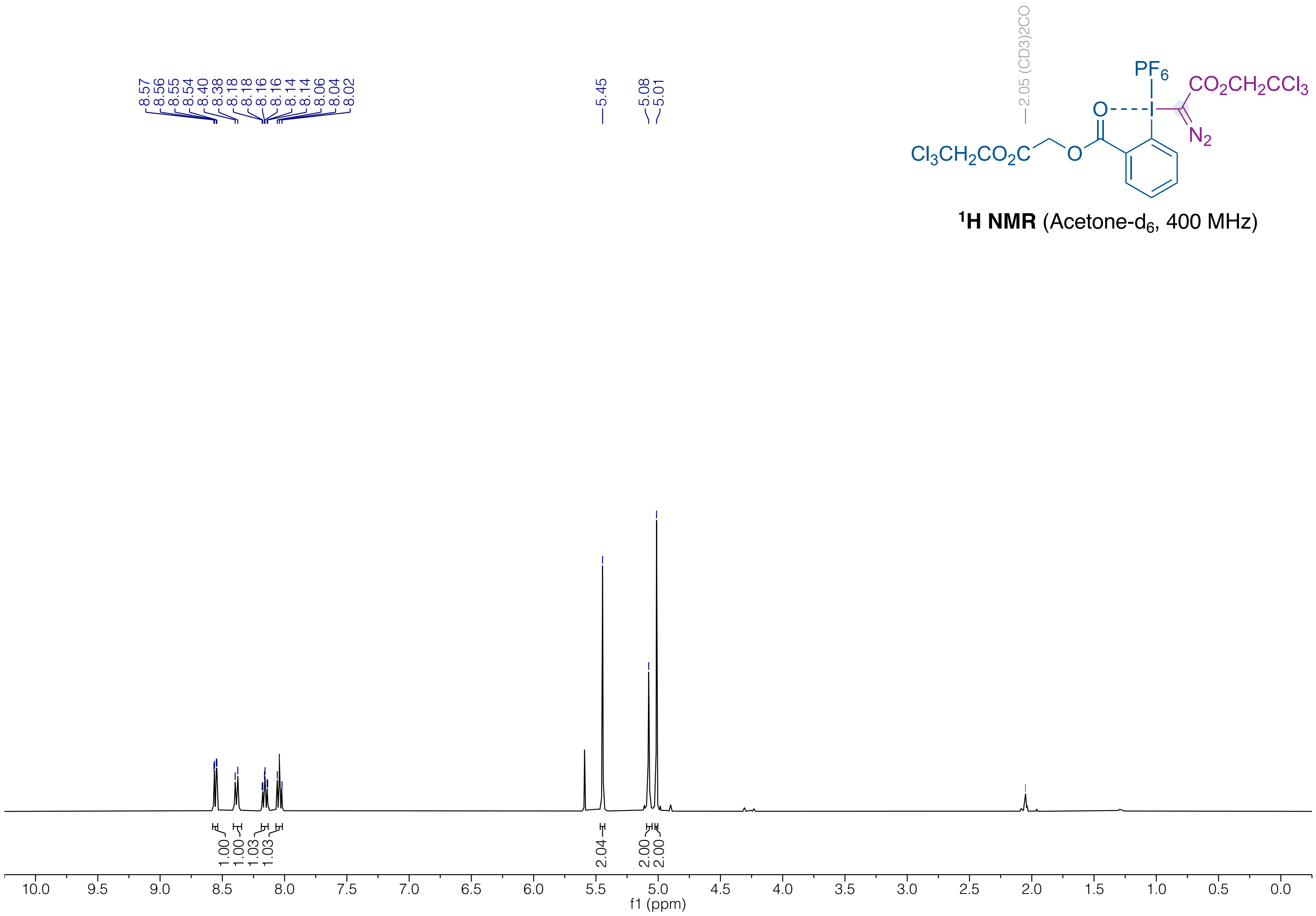

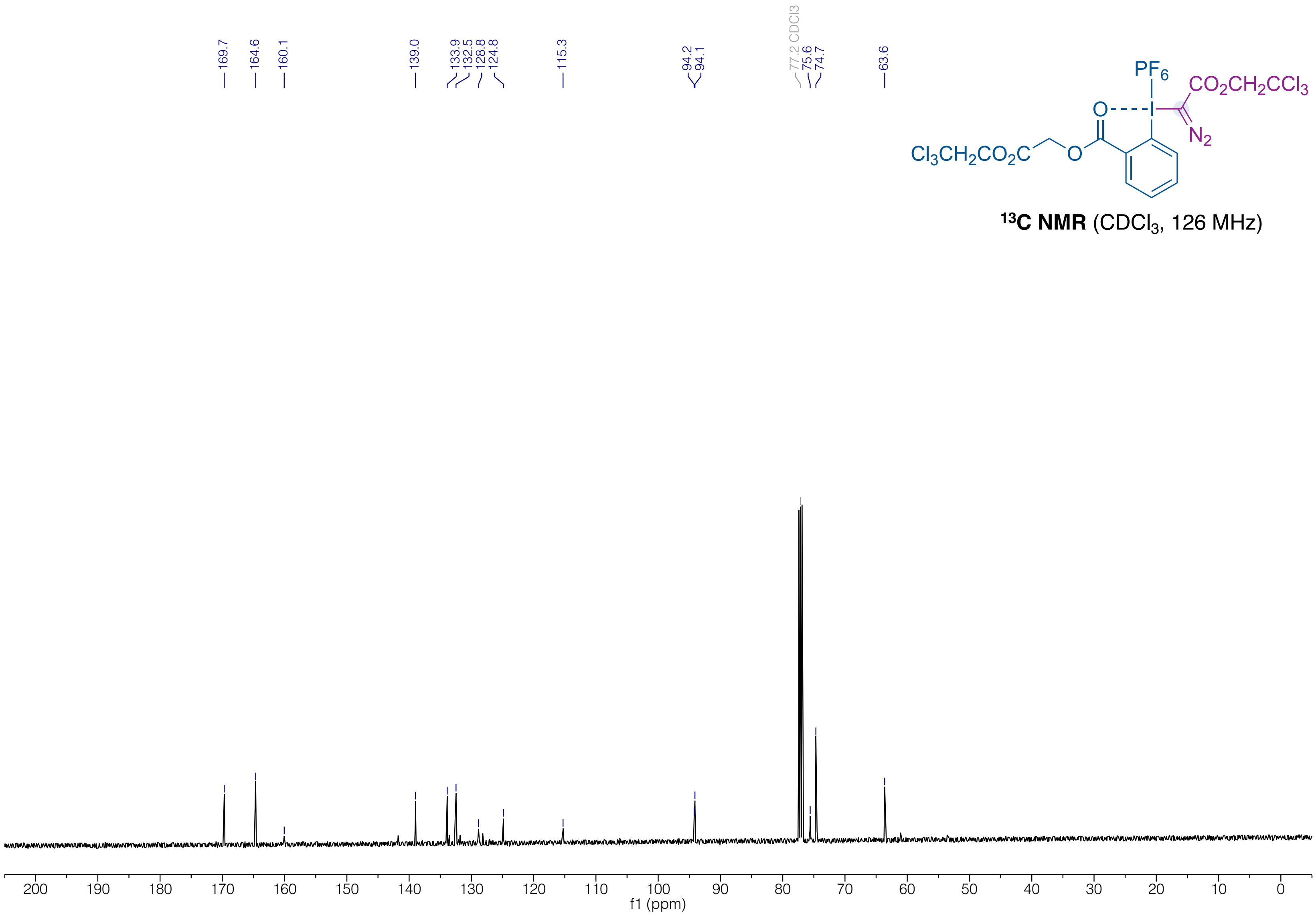

^{13}C NMR ($CDCl_3$, 126 MHz)

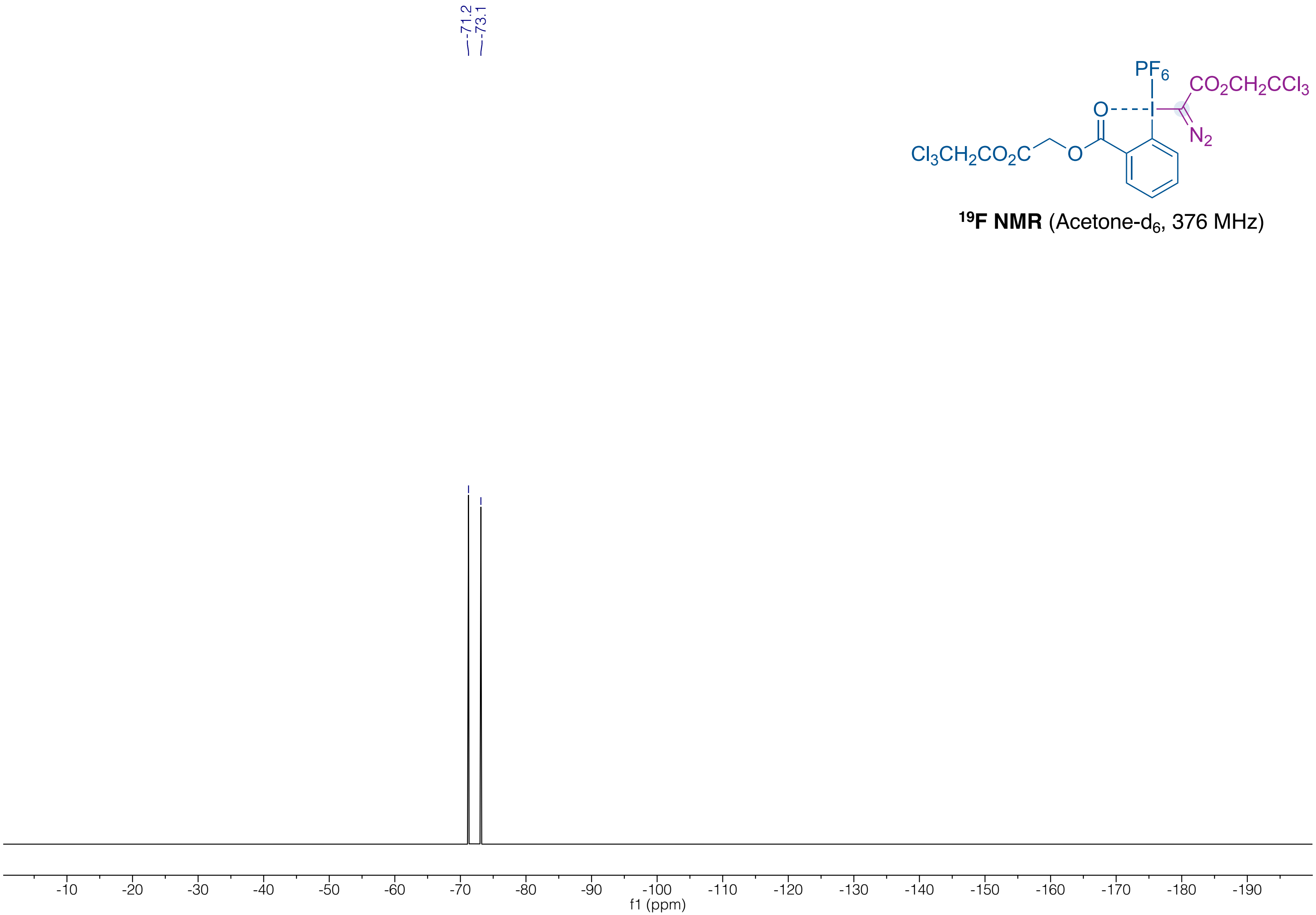

^{19}F NMR (Acetone-d$_6$, 376 MHz)

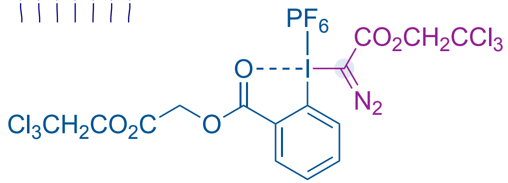

^{31}**P NMR** (Acetone-d_6, 162 MHz)

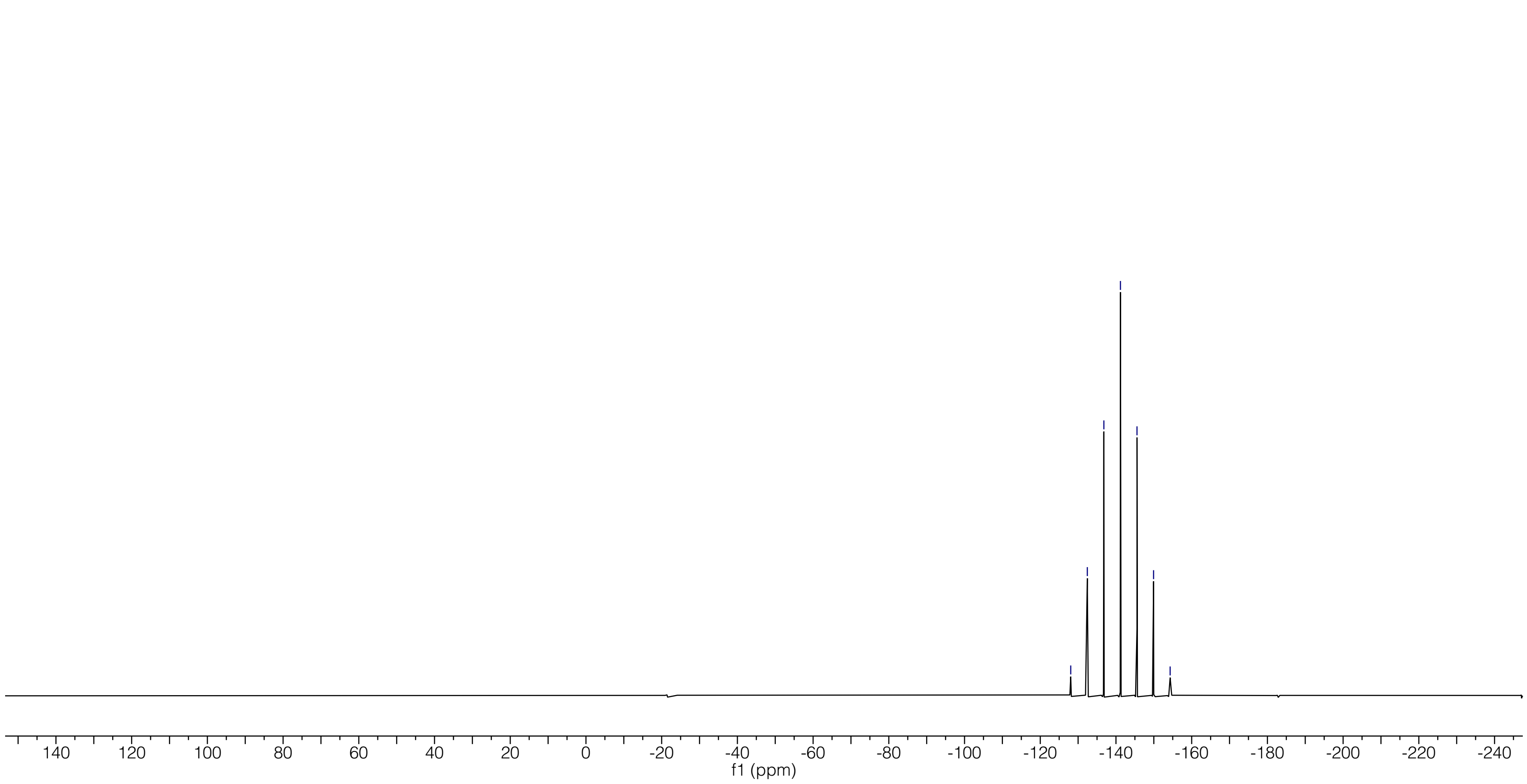

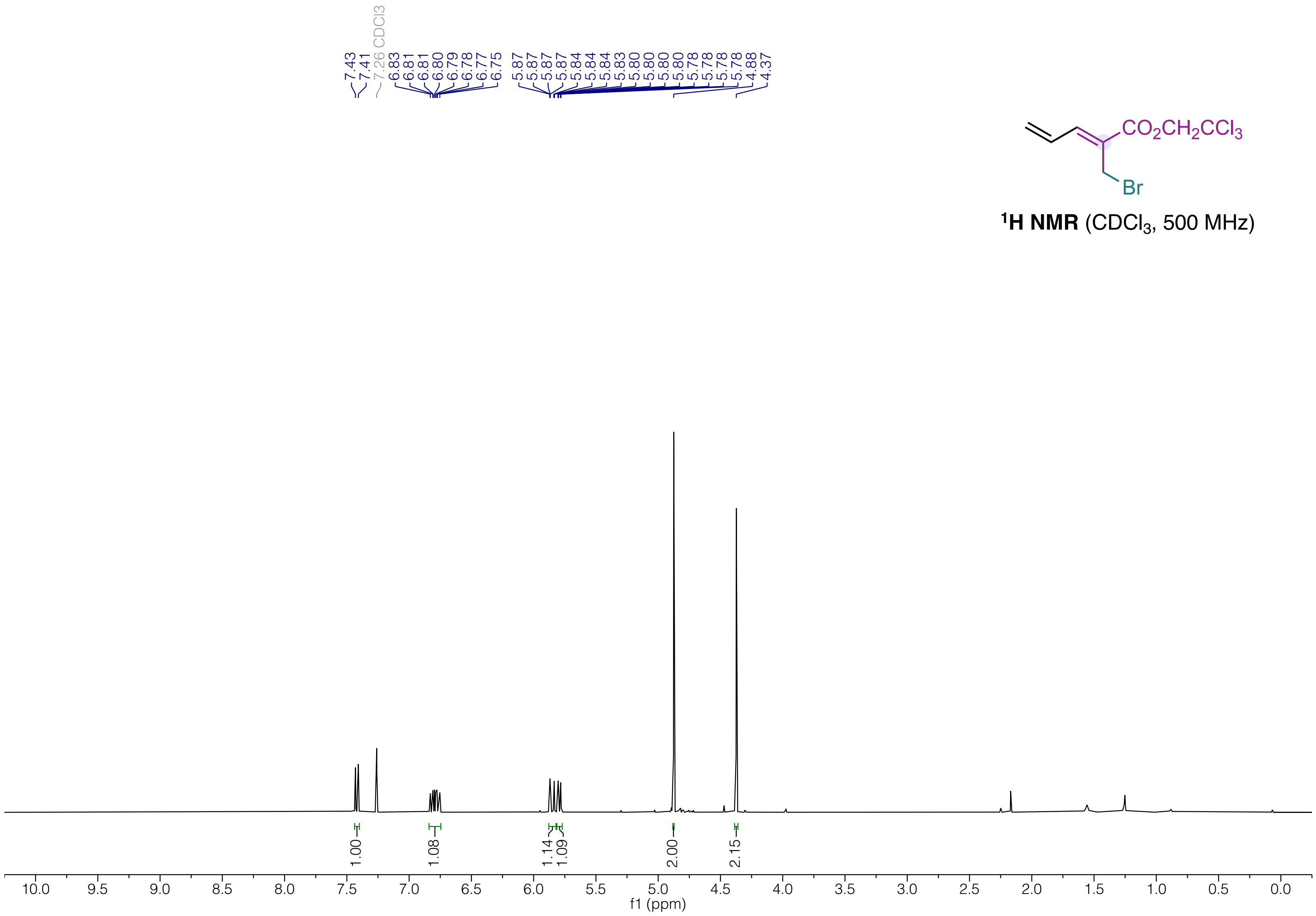

¹H NMR ($CDCl_3$, 500 MHz)

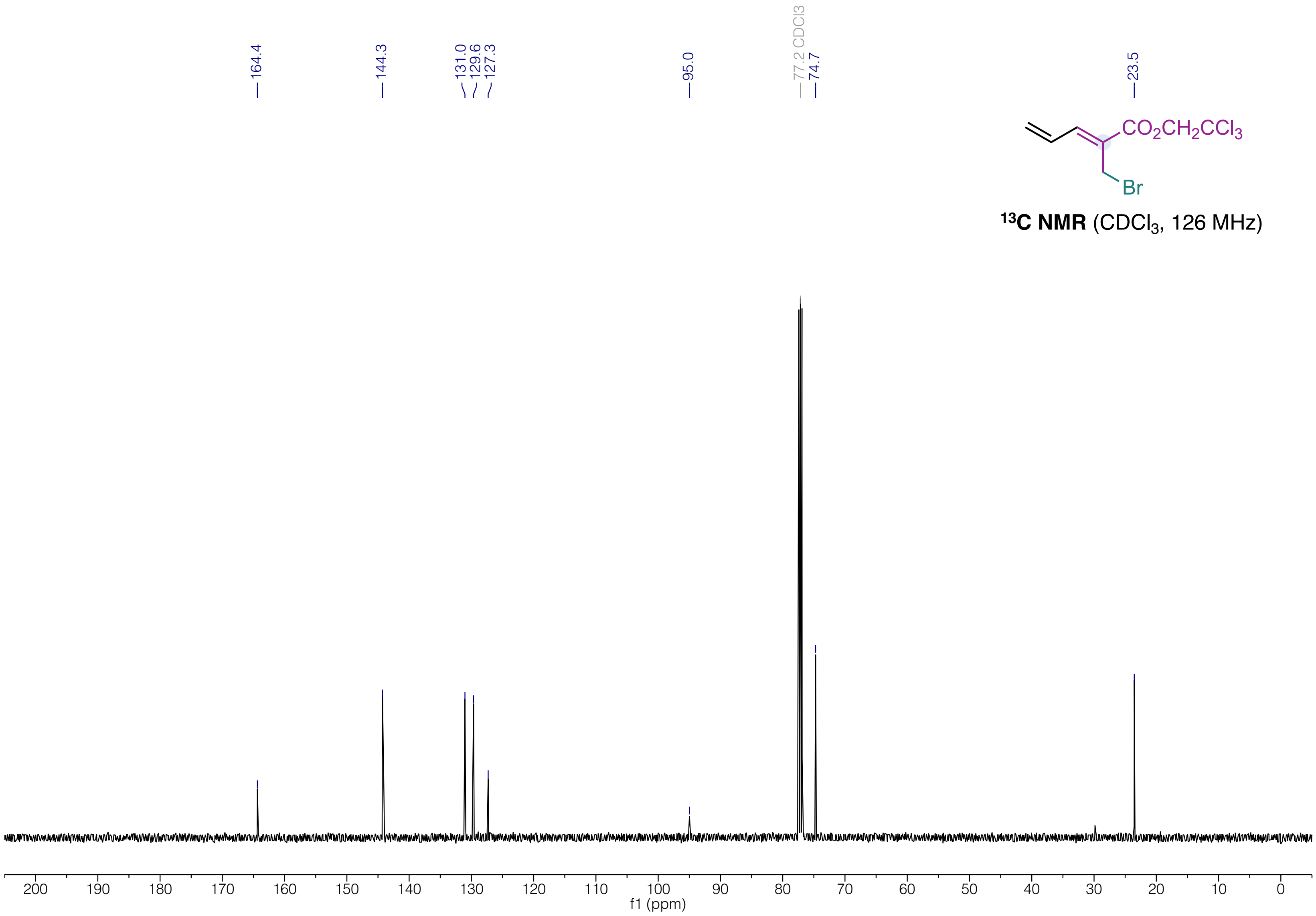
164.4
144.3
131.0
129.6
127.3
95.0
77.2 CDCl3
74.7
23.5
$CO_2CH_2CCl_3$
Br
^{13}C NMR ($CDCl_3$, 126 MHz)
200
190
180
170
160
150
140
130
120
110
100
90
80
70
60
50
40
30
20
10
0
f1 (ppm)

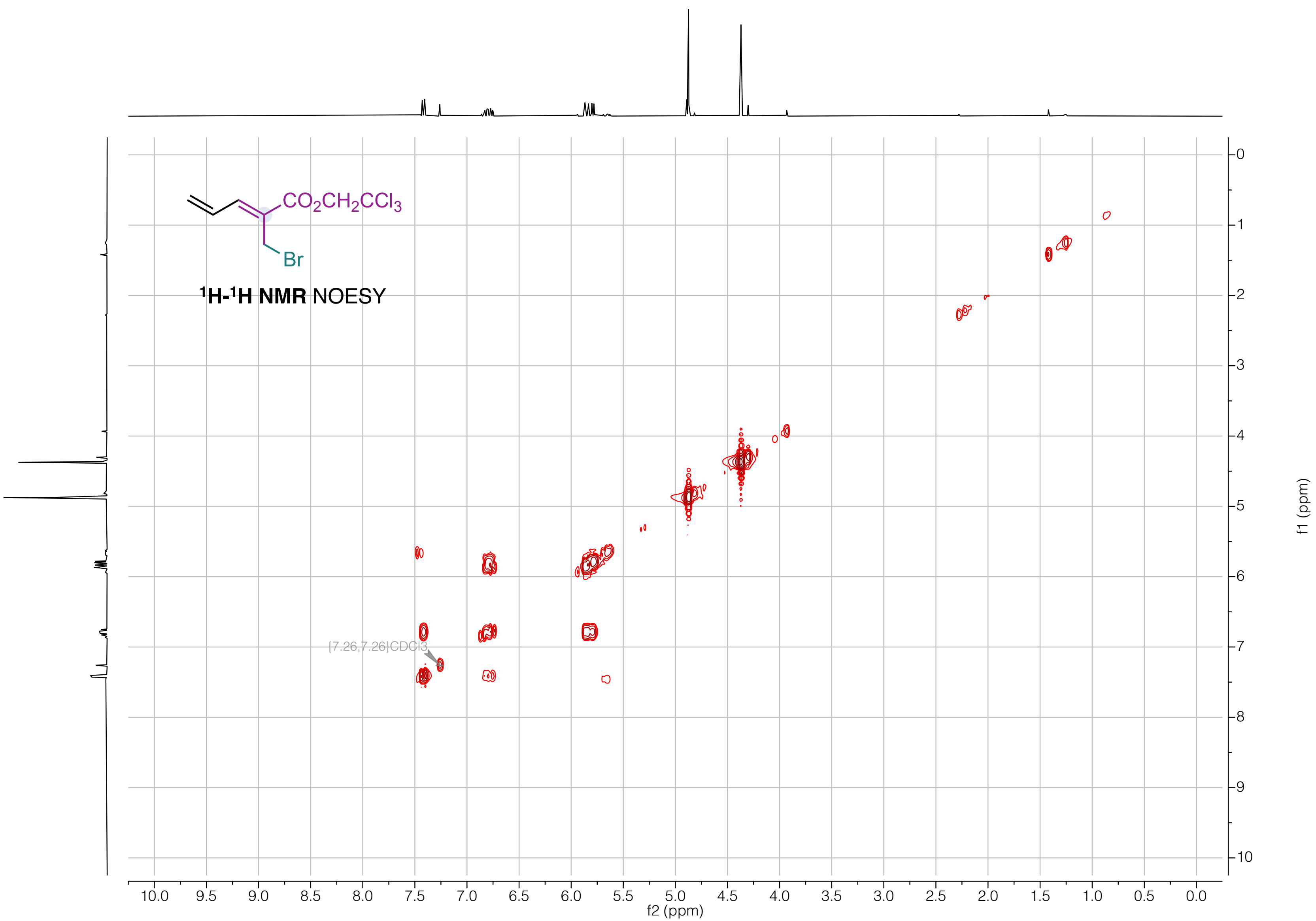
CO2CH2CCl3
Br
1H-1H NMR NOESY
{7.26,7.26}CDCl3
f2 (ppm)
f1 (ppm)

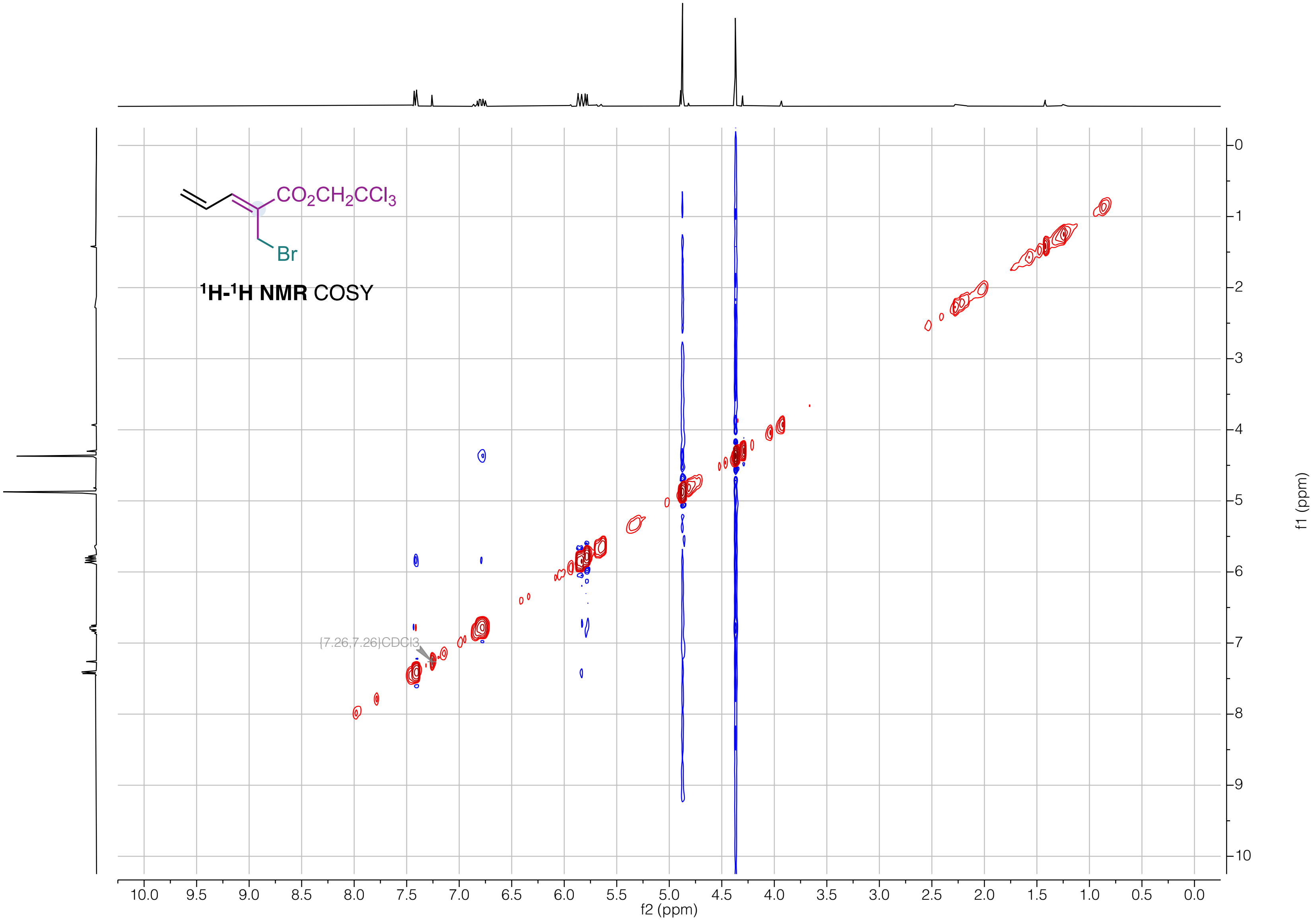
CO2CH2CCl3
Br
1H-1H NMR COSY
[7.26,7.26]CDCl3
f2 (ppm)
f1 (ppm)

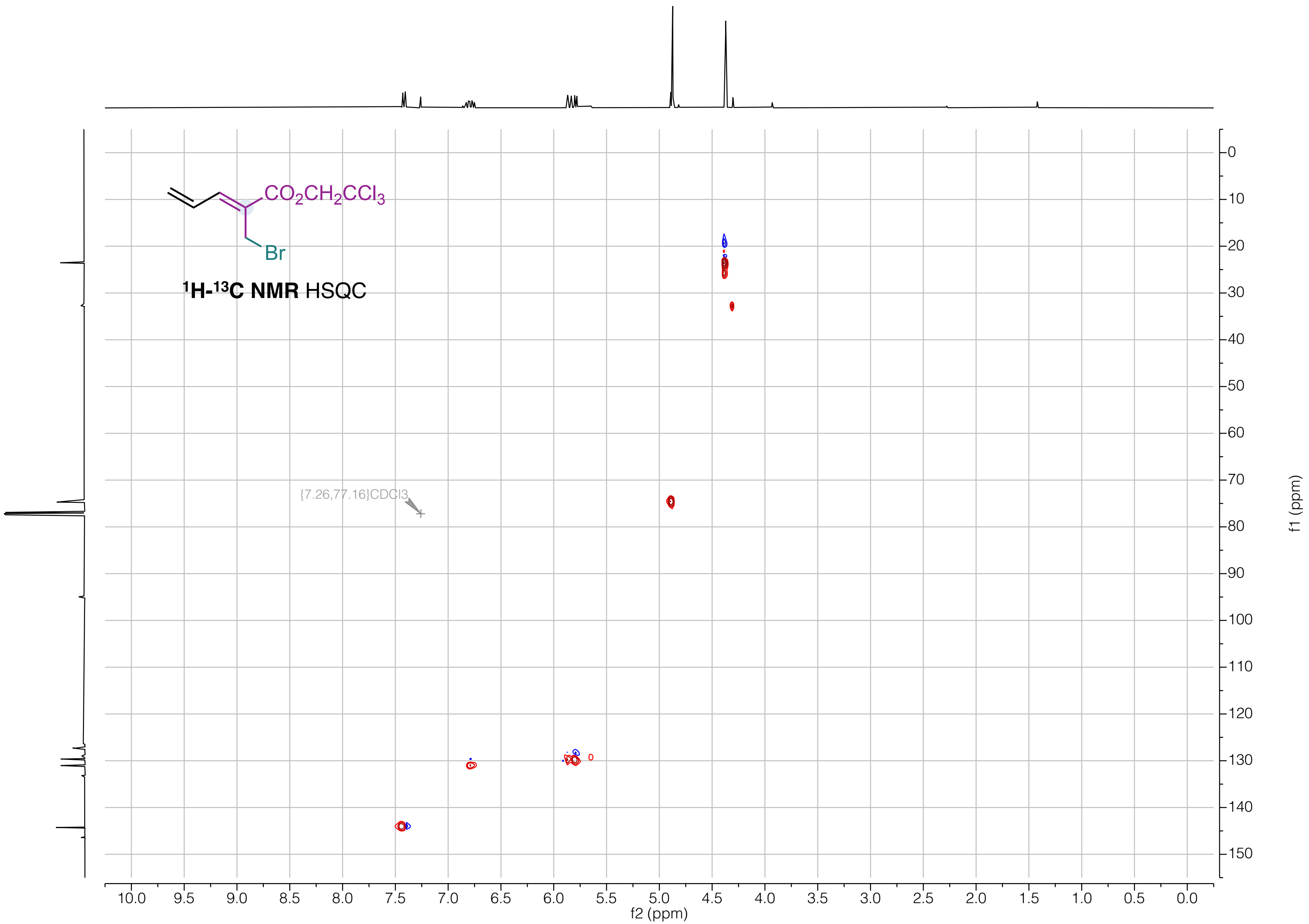

CO2CH2CCl3
Br
1H-13C NMR HSQC
{7.26,77.16}CDCl3
f2 (ppm)
f1 (ppm)

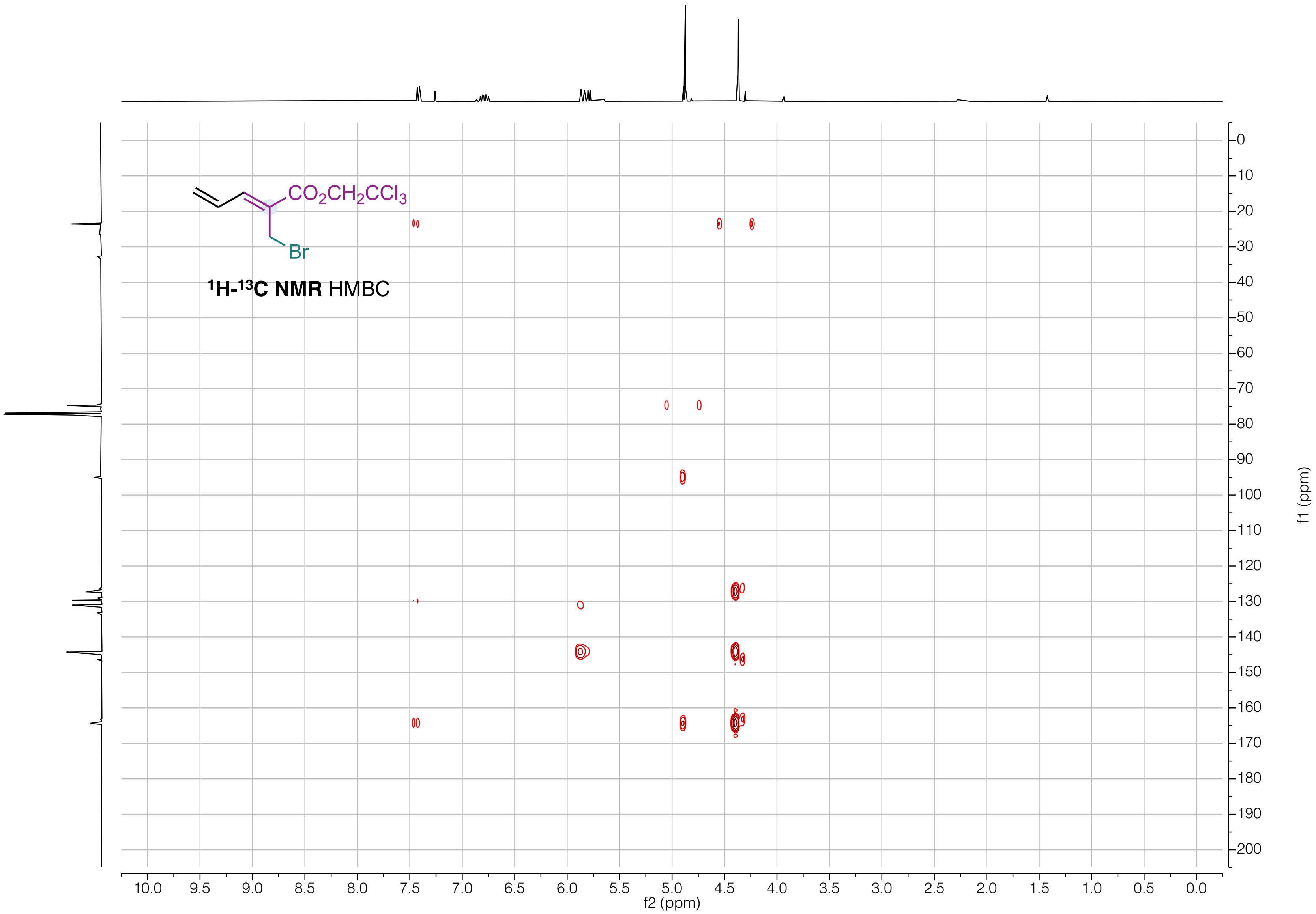

CO2CH2CCl3
Br
1H-13C NMR HMBC
f1 (ppm)
f2 (ppm)

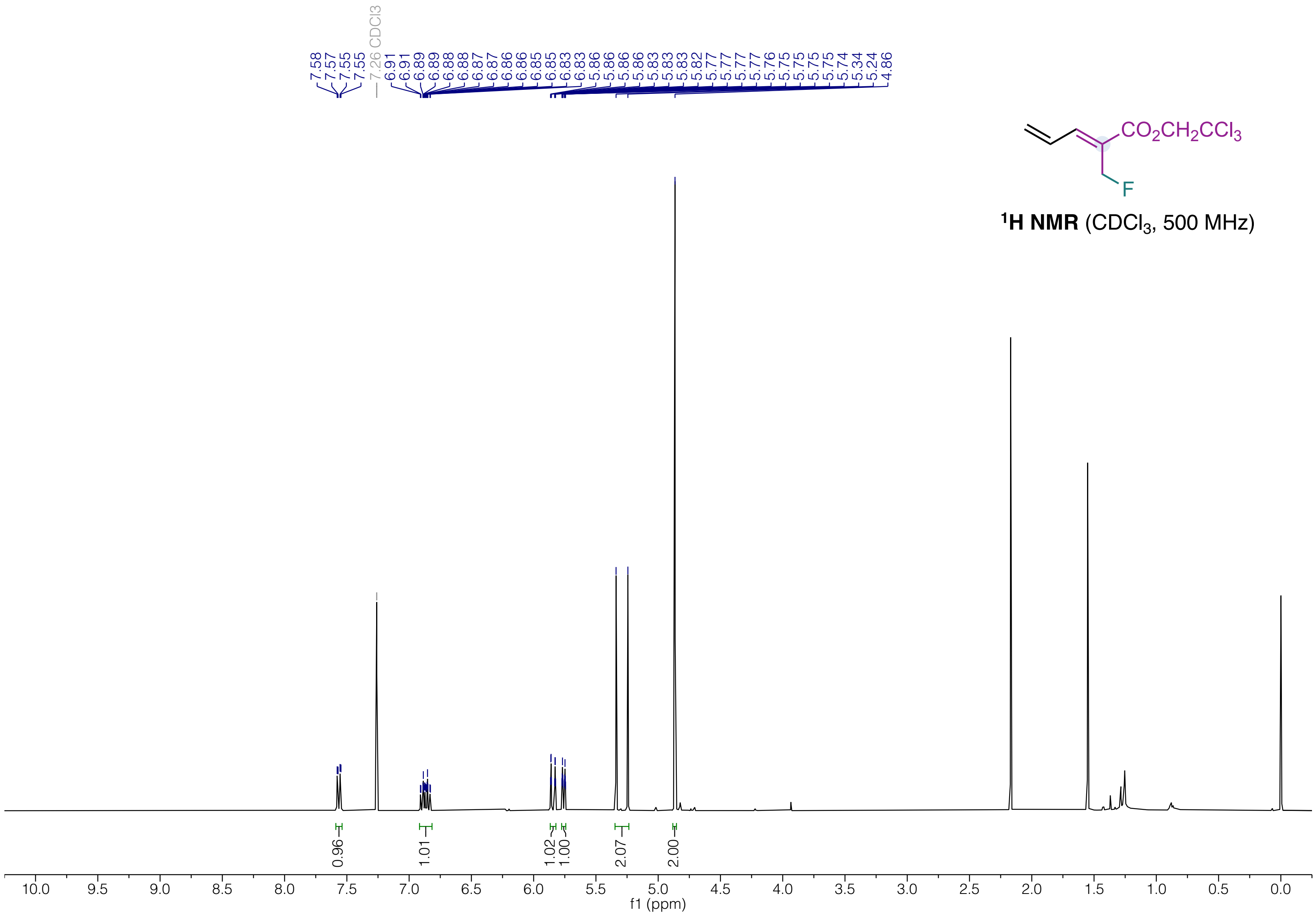

7.58
7.57
7.55
7.55
7.26 CDCl3
6.91
6.91
6.89
6.89
6.88
6.88
6.87
6.87
6.86
6.86
6.85
6.85
6.83
6.83
5.86
5.86
5.86
5.86
5.83
5.83
5.83
5.82
5.77
5.77
5.77
5.77
5.76
5.75
5.75
5.75
5.75
5.74
5.34
5.24
4.86
CO2CH2CCl3
F
1H NMR (CDCl3, 500 MHz)
0.96
1.01
1.02
1.00
2.07
2.00
10.0
9.5
9.0
8.5
8.0
7.5
7.0
6.5
6.0
5.5
5.0
4.5
4.0
3.5
3.0
2.5
2.0
1.5
1.0
0.5
0.0
f1 (ppm)

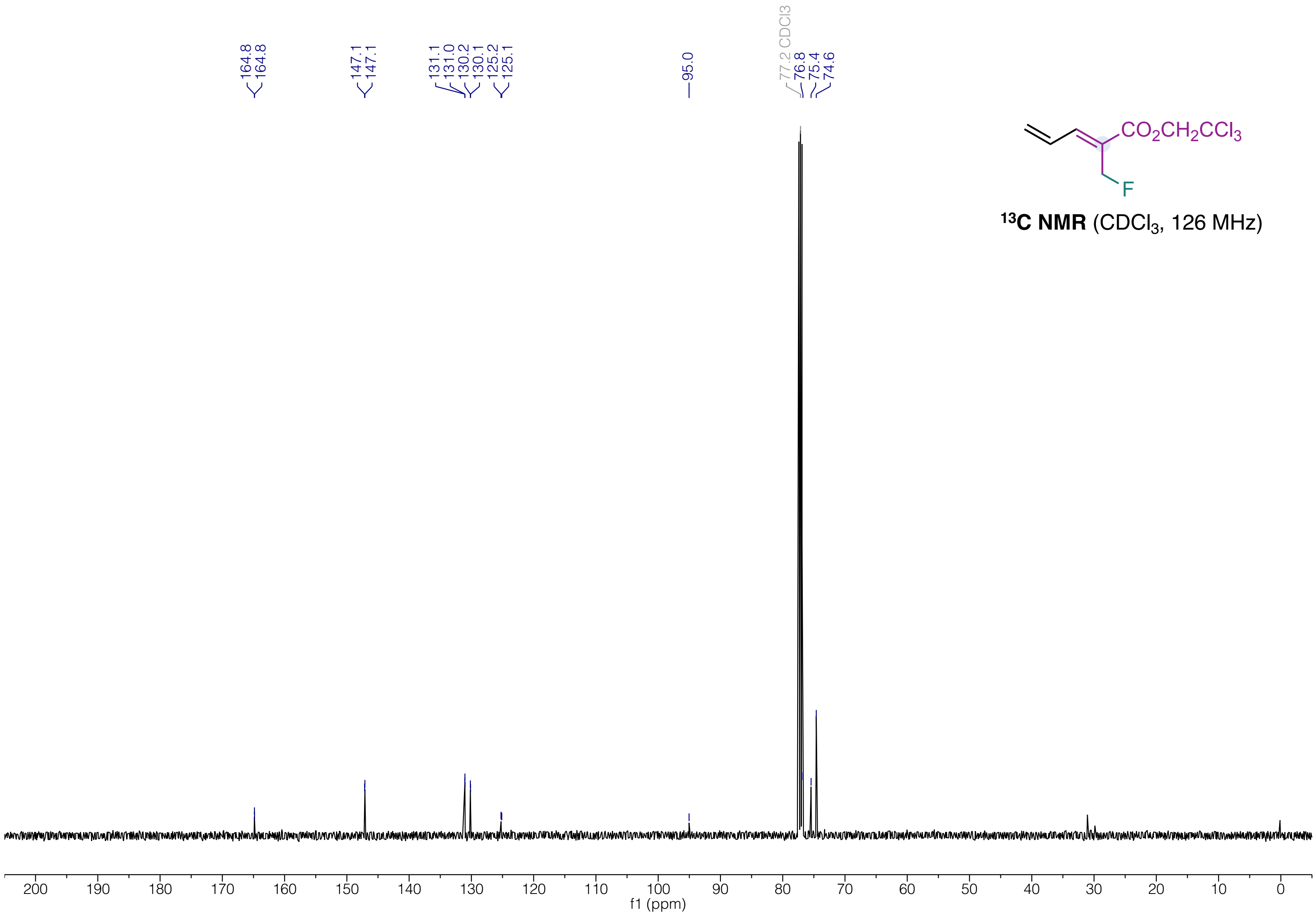
164.8
164.8
147.1
147.1
131.1
131.0
130.2
130.1
125.2
125.1
95.0
77.2 CDCl3
76.8
75.4
74.6
CO2CH2CCl3
F
13C NMR (CDCl3, 126 MHz)
200 190 180 170 160 150 140 130 120 110 100 90 80 70 60 50 40 30 20 10 0
f1 (ppm)

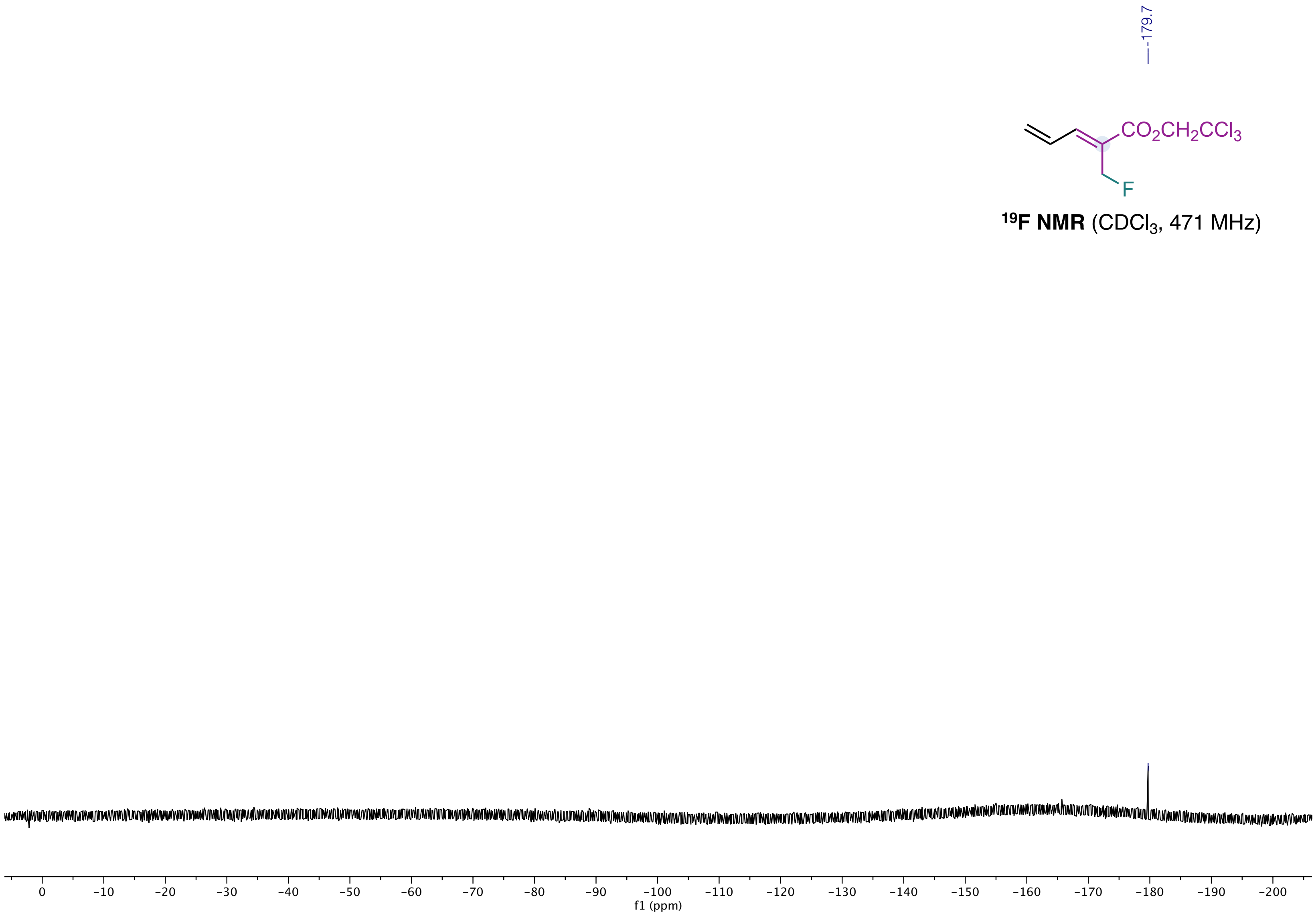
-179.7
$CO_2CH_2CCl_3$
F
^{19}F NMR ($CDCl_3$, 471 MHz)
0 -10 -20 -30 -40 -50 -60 -70 -80 -90 -100 -110 -120 -130 -140 -150 -160 -170 -180 -190 -200
f1 (ppm)

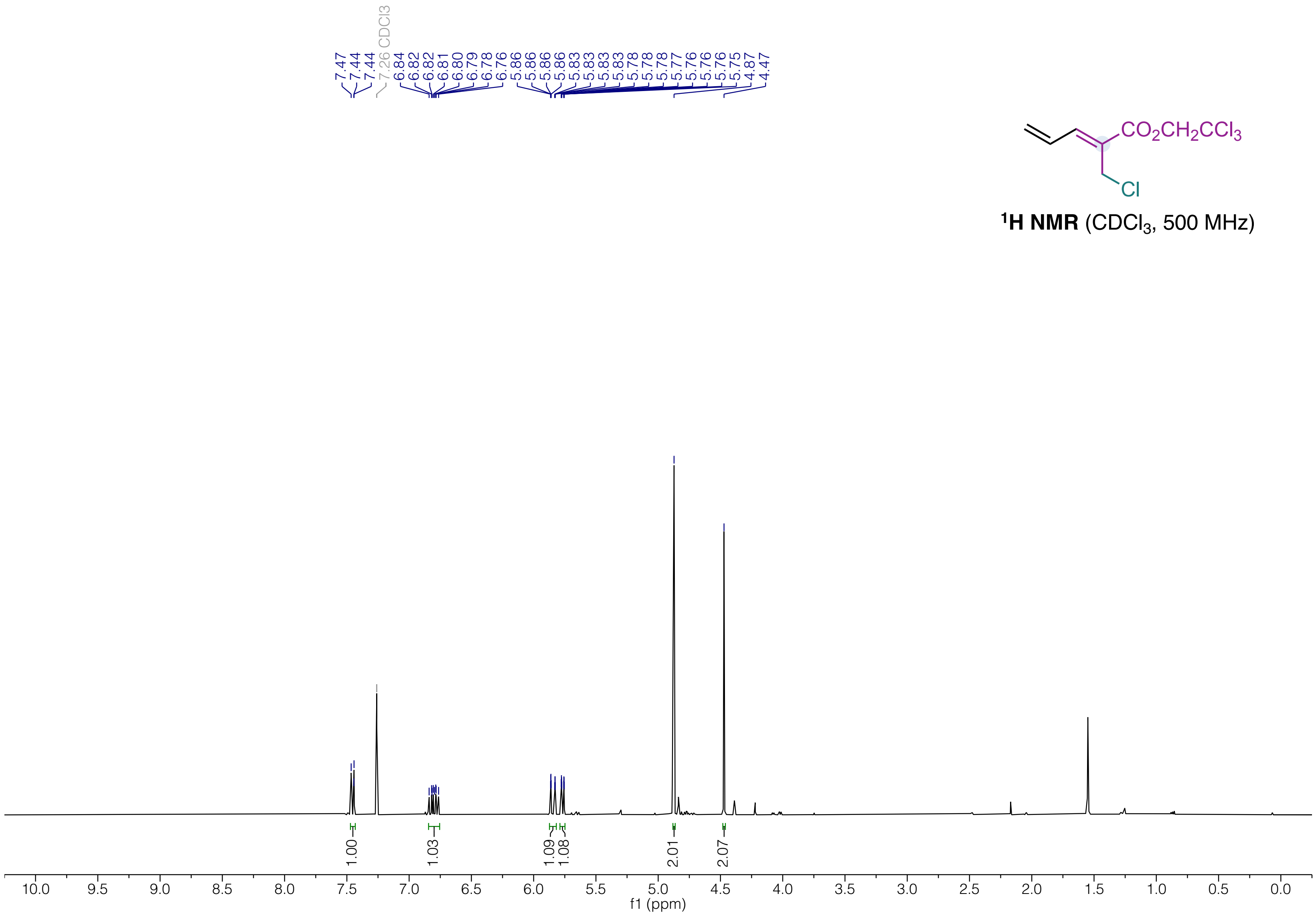

^{1}H NMR ($CDCl_3$, 500 MHz)

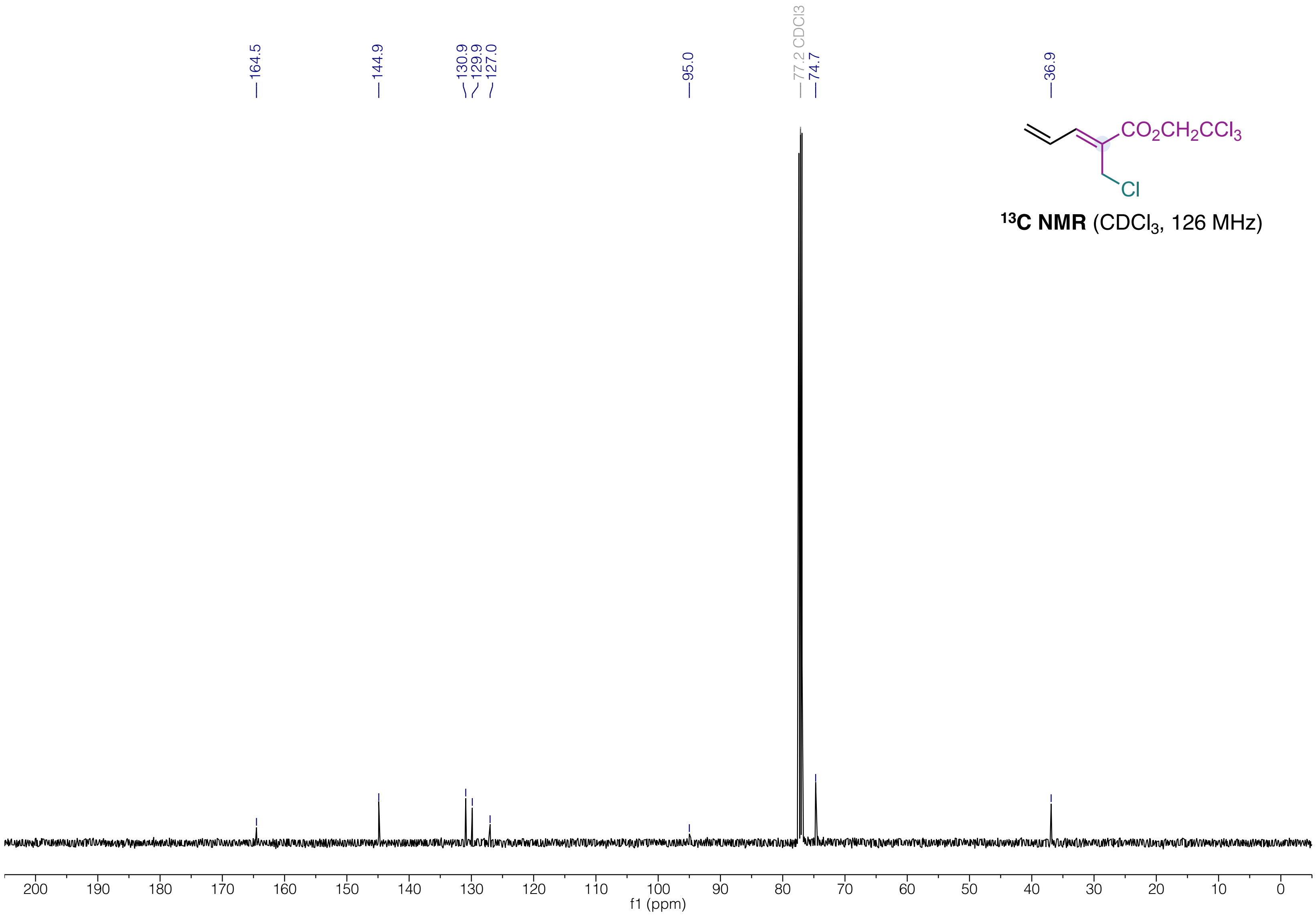
164.5
144.9
130.9
129.9
127.0
95.0
77.2 CDCl3
74.7
36.9
CO2CH2CCl3
Cl
13C NMR (CDCl3, 126 MHz)
200 190 180 170 160 150 140 130 120 110 100 90 80 70 60 50 40 30 20 10 0
f1 (ppm)

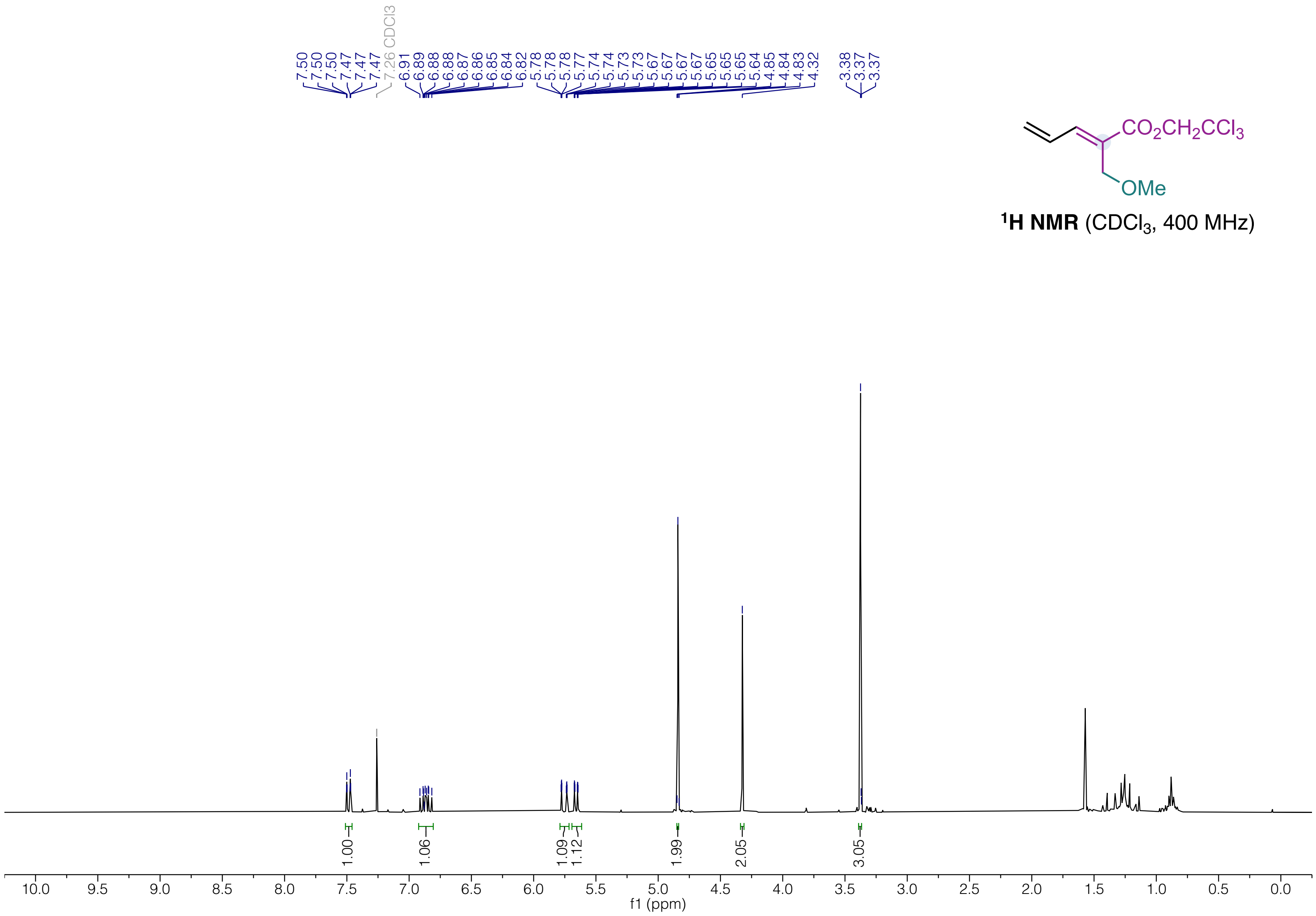
CO2CH2CCl3
OMe
1H NMR (CDCl3, 400 MHz)
7.50 7.50 7.50 7.47 7.47 7.47 7.26 CDCl3 6.91 6.89 6.88 6.87 6.86 6.85 6.84 6.82 5.78 5.78 5.77 5.74 5.74 5.73 5.73 5.67 5.67 5.67 5.65 5.65 5.65 5.64 4.85 4.84 4.83 4.32 3.38 3.37 3.37
1.00 1.06 1.09 1.12 1.99 2.05 3.05
10.0 9.5 9.0 8.5 8.0 7.5 7.0 6.5 6.0 5.5 5.0 4.5 4.0 3.5 3.0 2.5 2.0 1.5 1.0 0.5 0.0
f1 (ppm)

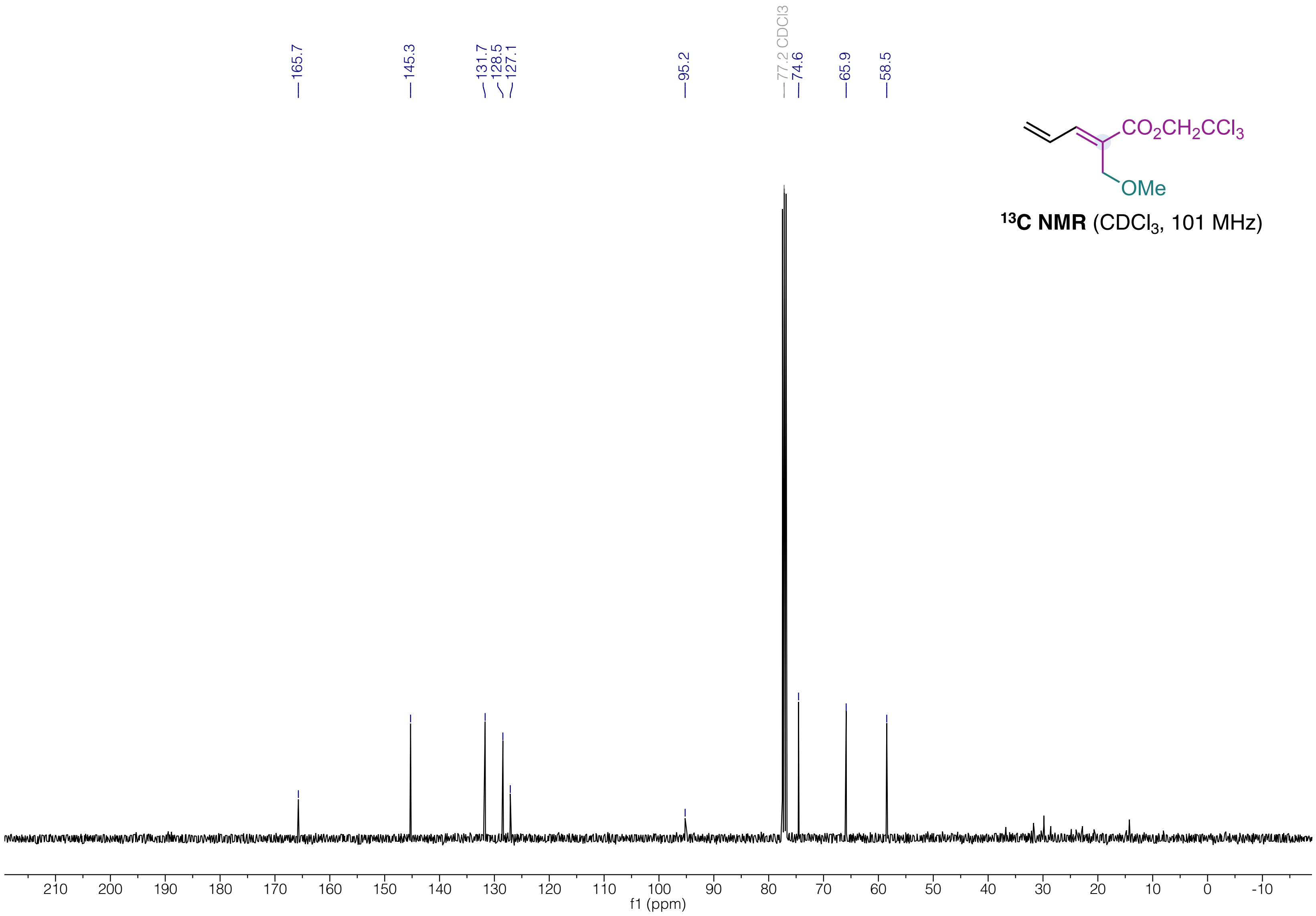

165.7
145.3
131.7
128.5
127.1
95.2
77.2 CDCl3
74.6
65.9
58.5
CO2CH2CCl3
OMe
13C NMR (CDCl3, 101 MHz)
210
200
190
180
170
160
150
140
130
120
110
100
90
80
70
60
50
40
30
20
10
0
-10
f1 (ppm)

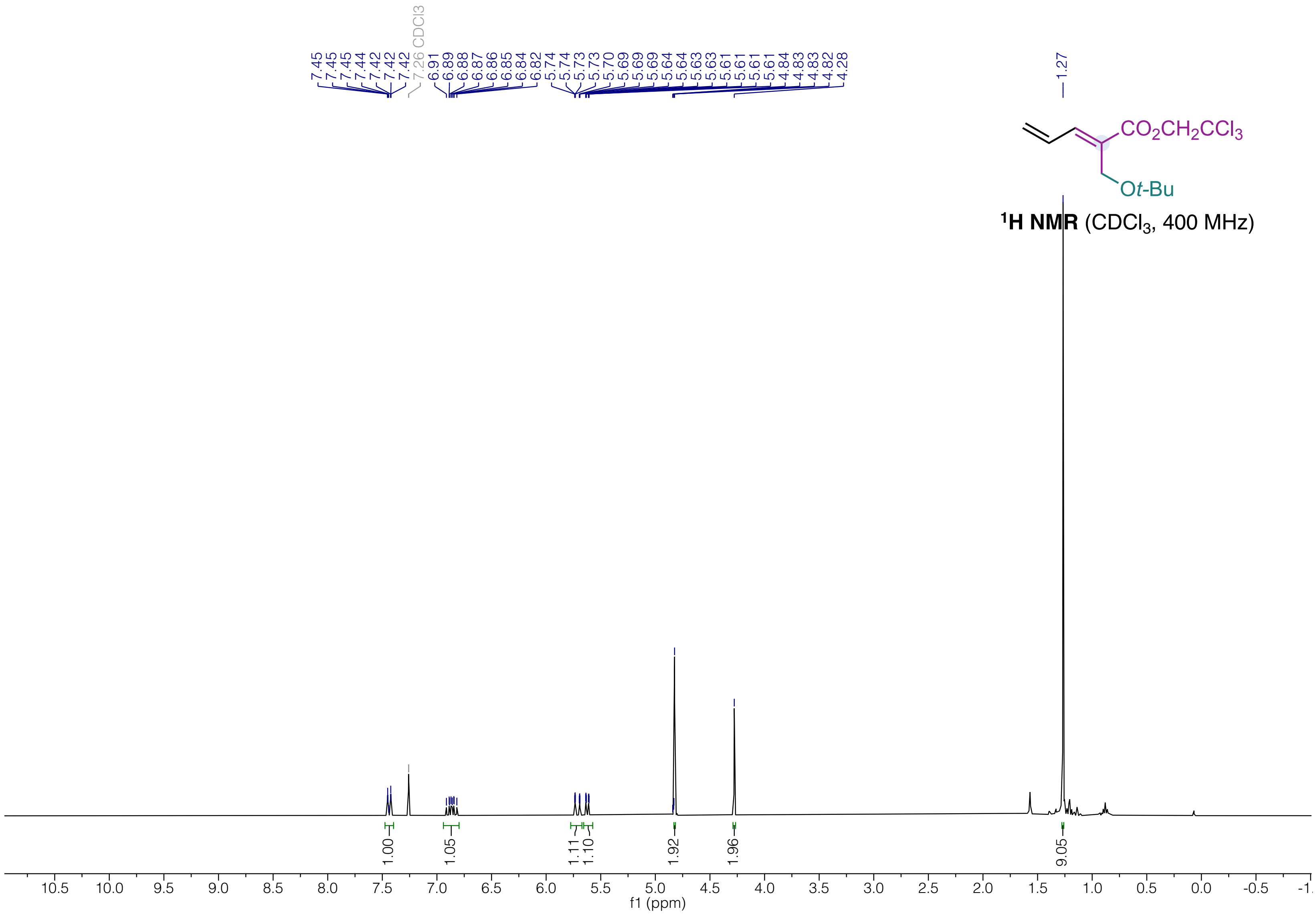

7.45
7.45
7.45
7.44
7.42
7.42
7.42
7.26 CDCl3
6.91
6.89
6.88
6.87
6.86
6.85
6.84
6.82
5.74
5.74
5.73
5.73
5.70
5.69
5.69
5.69
5.64
5.63
5.63
5.63
5.61
5.61
5.61
4.84
4.83
4.82
4.28
1.27
CO2CH2CCl3
Ot-Bu
1H NMR (CDCl3, 400 MHz)
1.00
1.05
1.11
1.10
1.92
1.96
9.05
f1 (ppm)

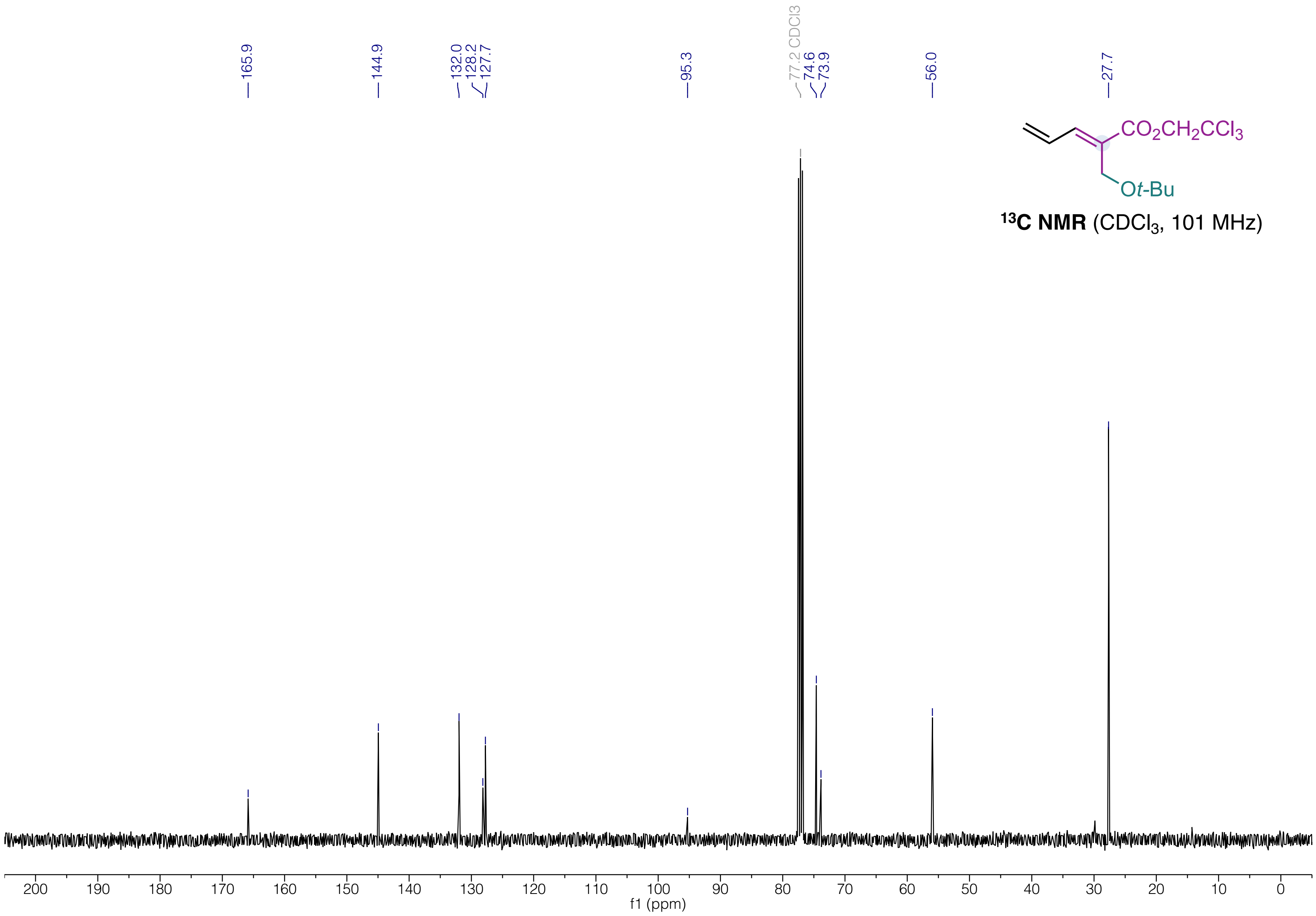

^{13}C NMR ($CDCl_3$, 101 MHz)

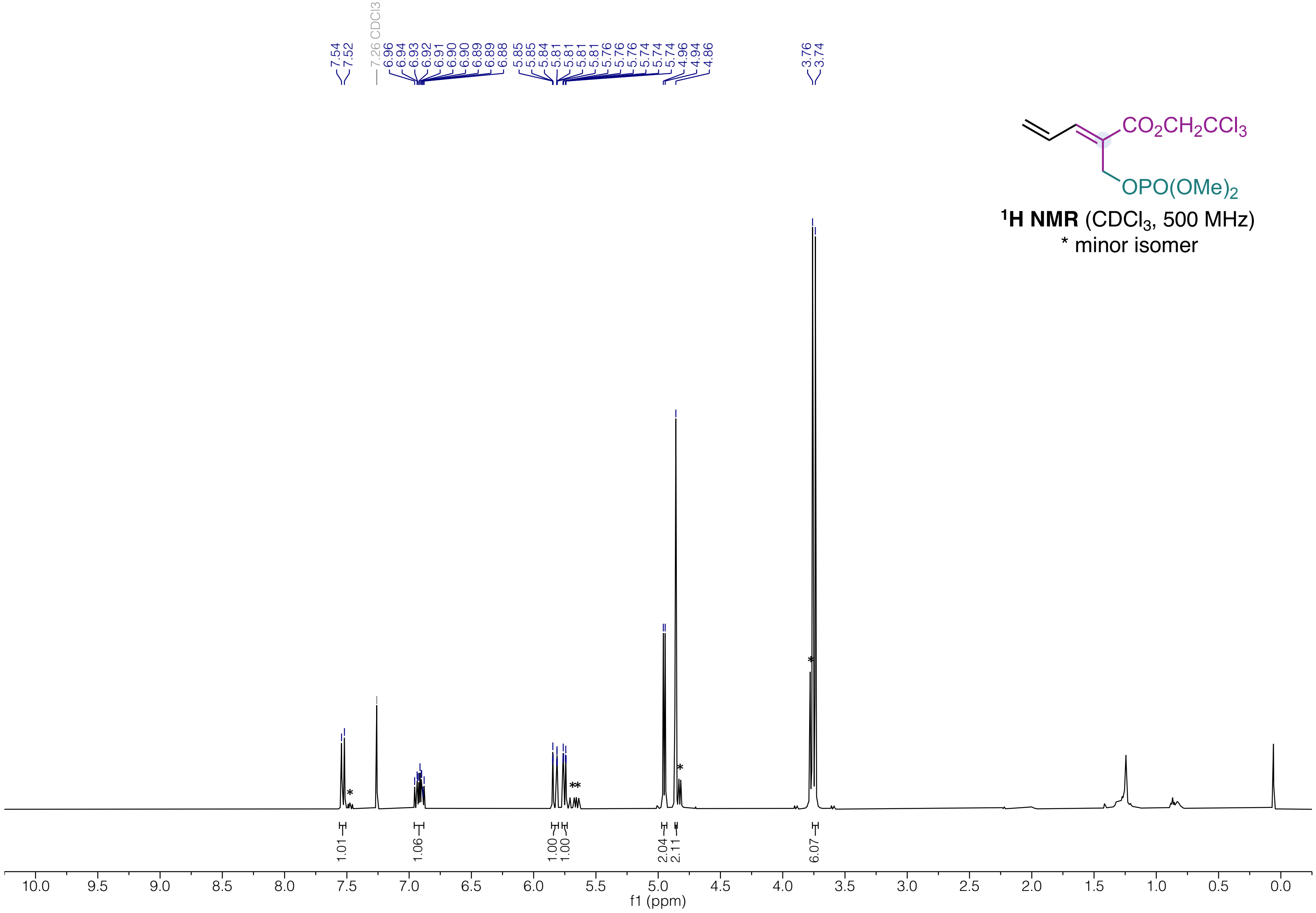

$CO_2CH_2CCl_3$
$OPO(OMe)_2$
^{1}H NMR ($CDCl_3$, 500 MHz)
* minor isomer
7.54
7.52
7.26 CDCl3
6.96
6.94
6.93
6.92
6.91
6.90
6.89
6.88
5.85
5.85
5.84
5.81
5.81
5.81
5.76
5.76
5.74
5.74
4.96
4.94
4.86
3.76
3.74
1.01
1.06
1.00
1.00
2.04
2.11
6.07
f1 (ppm)

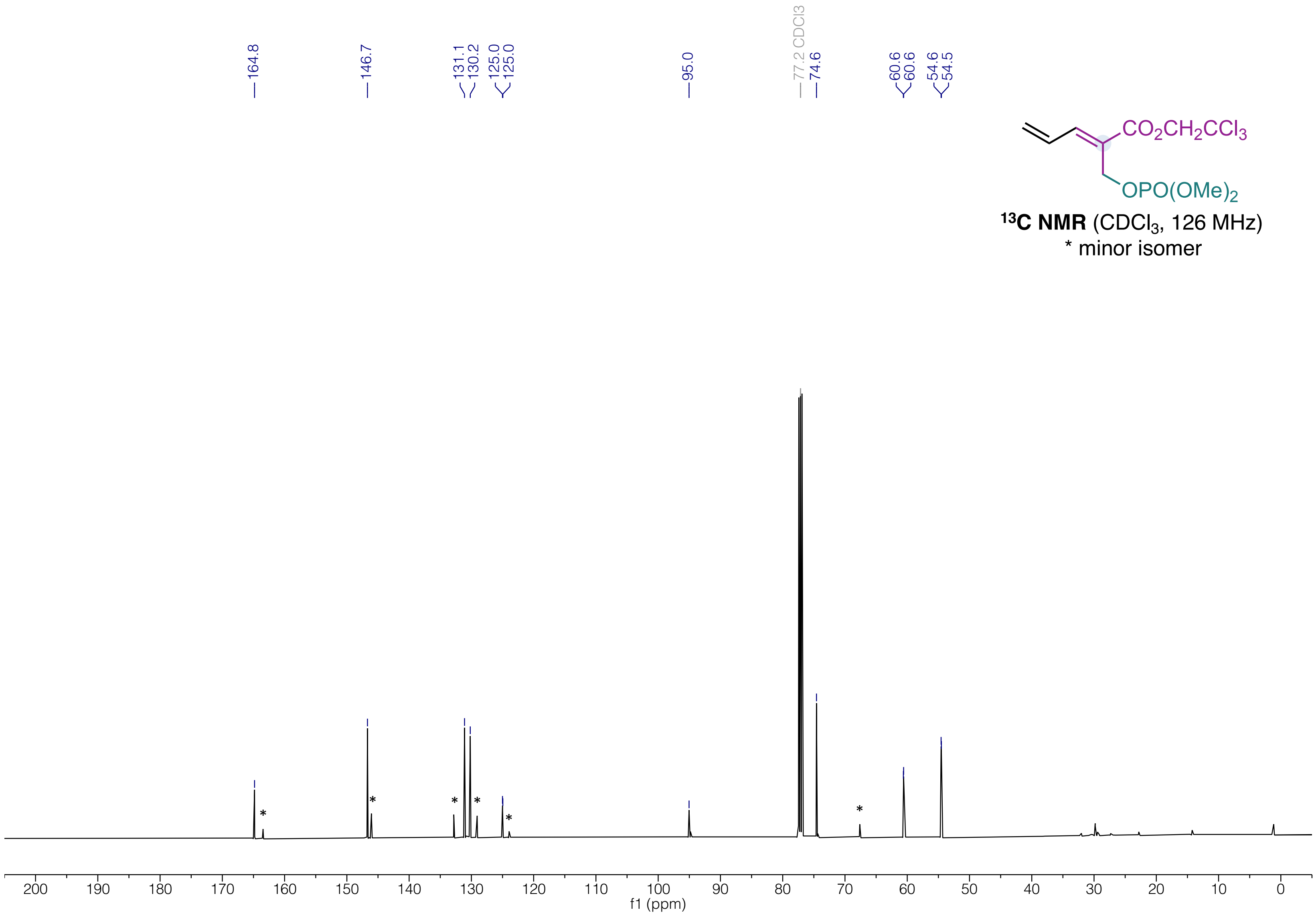

164.8
146.7
131.1
130.2
125.0
125.0
95.0
77.2 CDCl3
74.6
60.6
60.6
54.6
54.5
CO2CH2CCl3
OPO(OMe)2
13C NMR (CDCl3, 126 MHz)
* minor isomer
200 190 180 170 160 150 140 130 120 110 100 90 80 70 60 50 40 30 20 10 0
f1 (ppm)

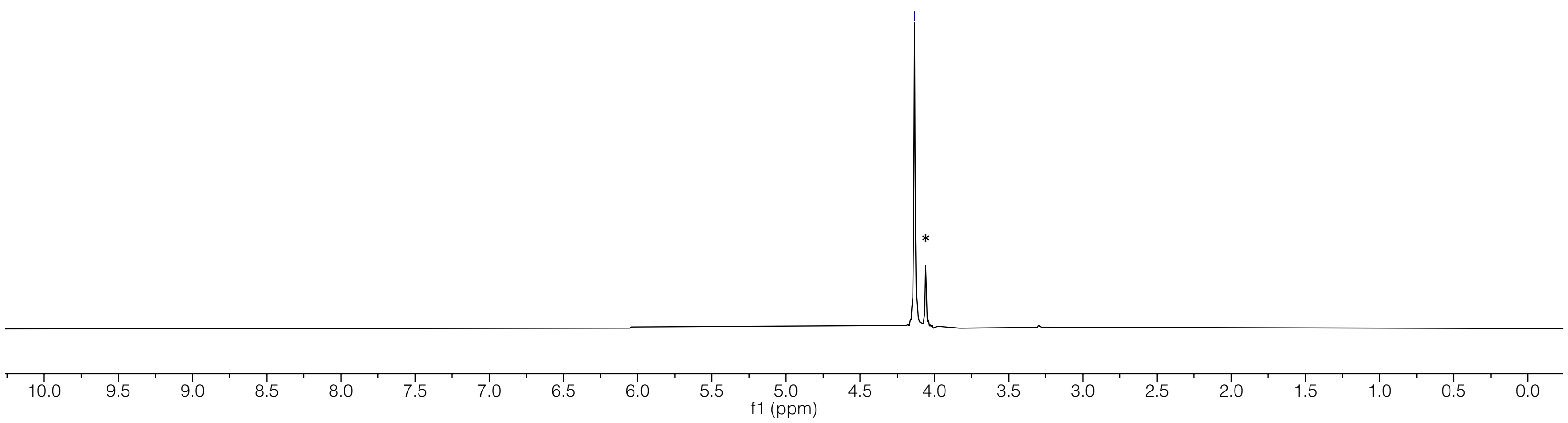

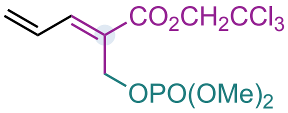

^{31}P NMR ($CDCl_3$, 202 MHz)
* minor isomer

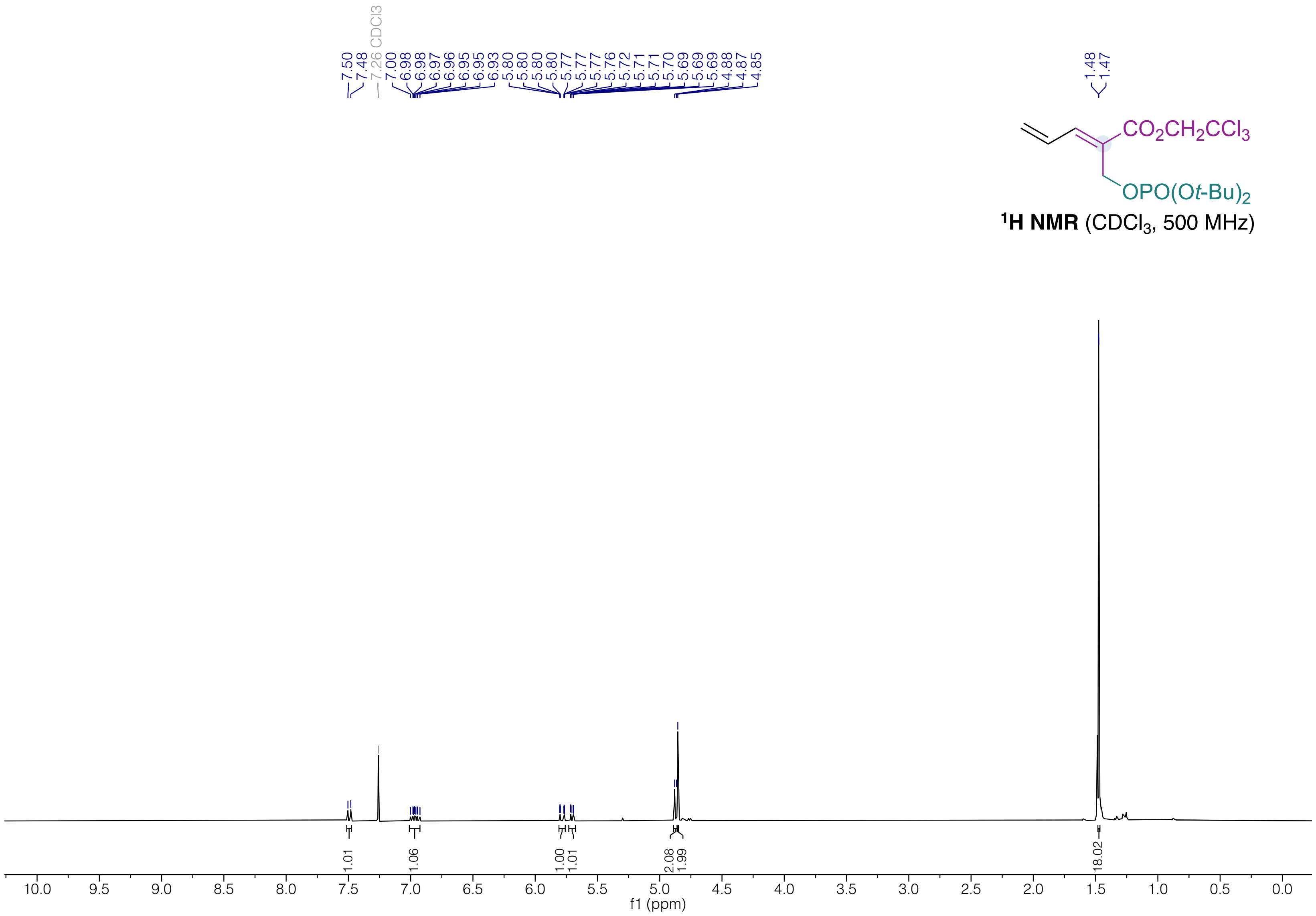
CO2CH2CCl3
OPO(Ot-Bu)2
1H NMR (CDCl3, 500 MHz)
7.50
7.48
7.26 CDCl3
7.00
6.98
6.98
6.97
6.96
6.95
6.95
6.93
5.80
5.80
5.80
5.77
5.77
5.76
5.72
5.71
5.71
5.70
5.69
5.69
5.69
4.88
4.87
4.85
1.48
1.47
1.01
1.06
1.00
1.01
2.08
1.99
18.02
f1 (ppm)

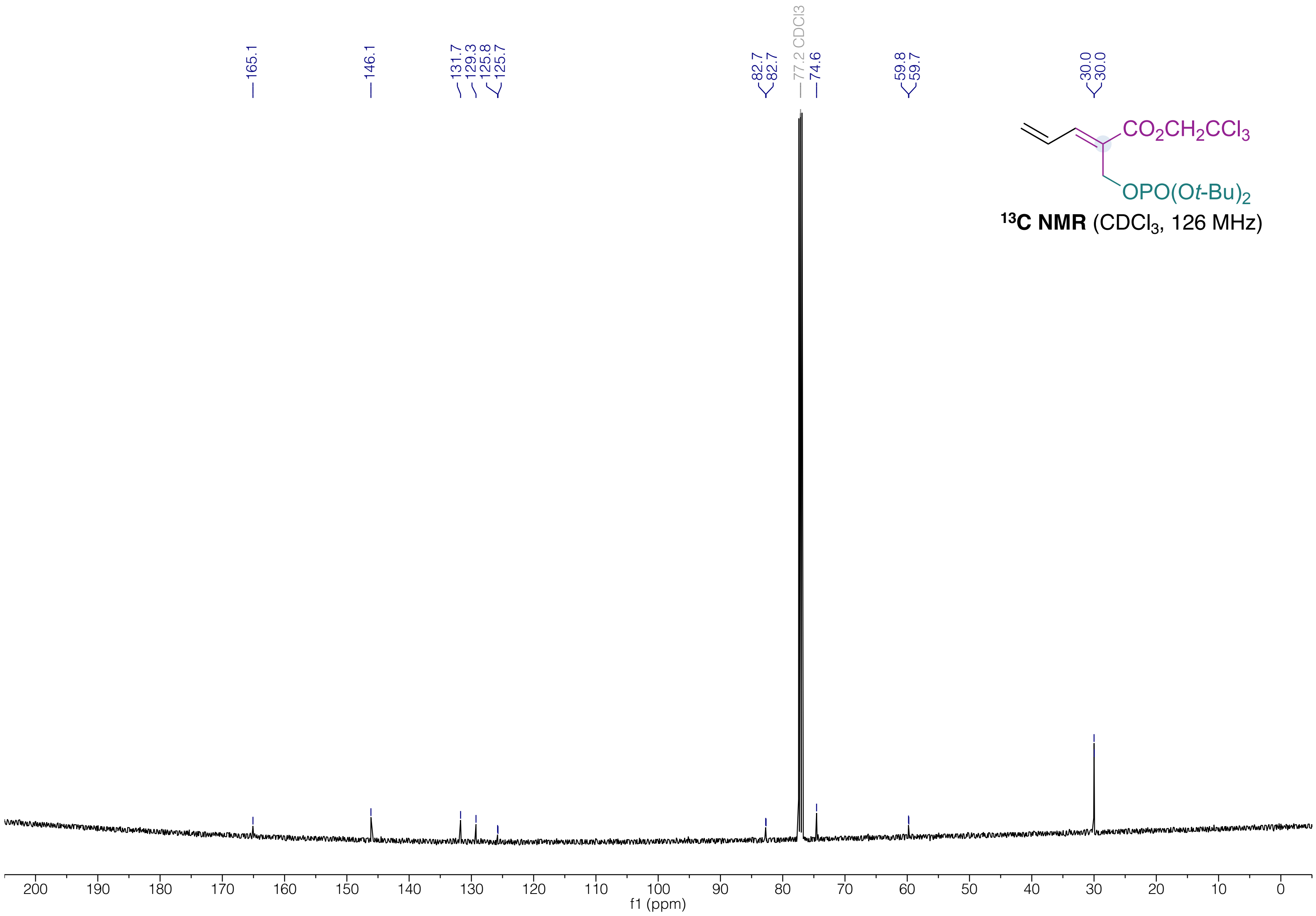

165.1
146.1
131.7
129.3
125.8
125.7
82.7
82.7
77.2 CDCl3
74.6
59.8
59.7
30.0
30.0
CO2CH2CCl3
OPO(Ot-Bu)2
13C NMR (CDCl3, 126 MHz)
200
190
180
170
160
150
140
130
120
110
100
90
80
70
60
50
40
30
20
10
0
f1 (ppm)

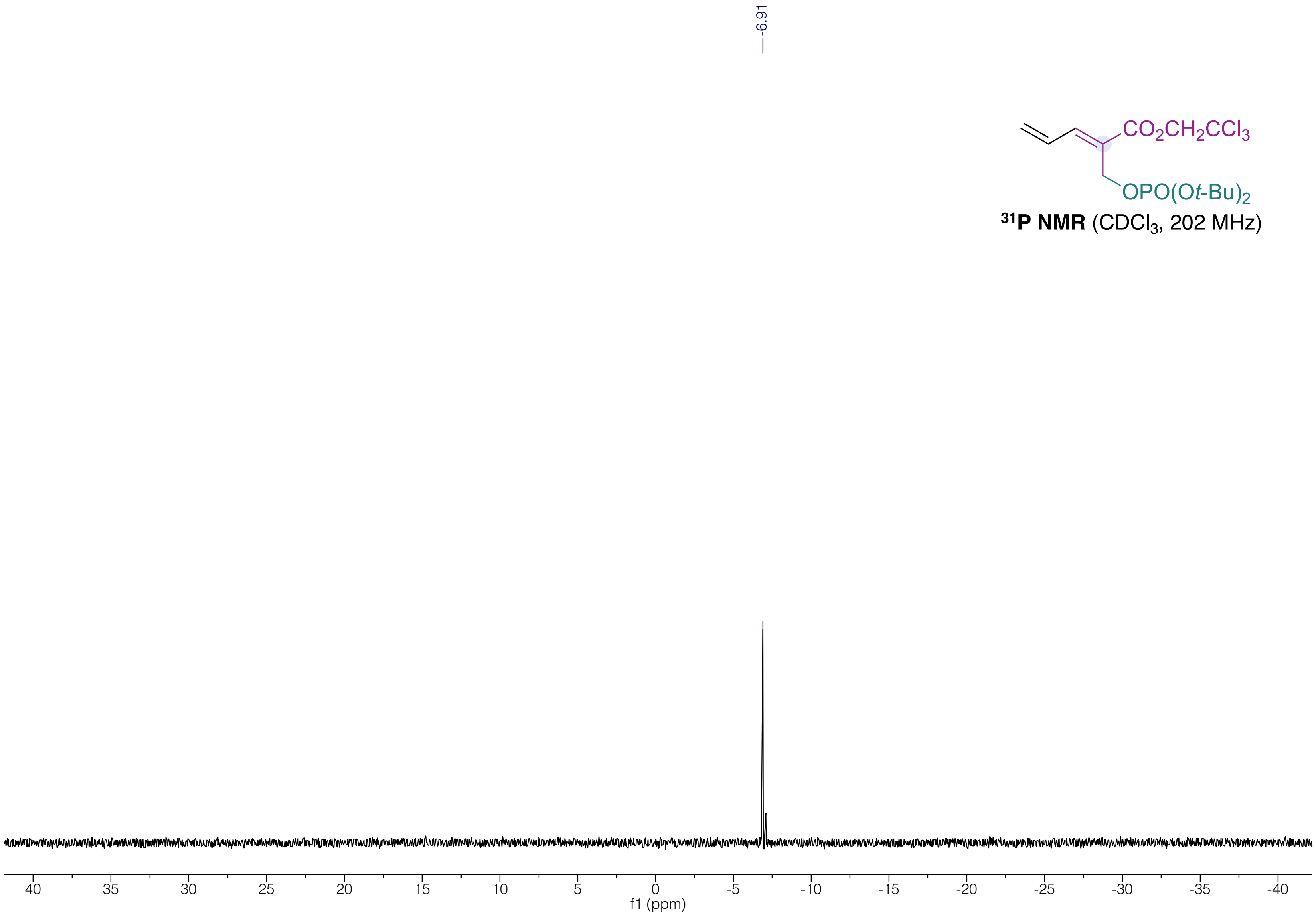

-6.91
$CO_2CH_2CCl_3$
$OPO(Ot\text{-}Bu)_2$
^{31}P NMR ($CDCl_3$, 202 MHz)
40
35
30
25
20
15
10
5
0
-5
-10
-15
-20
-25
-30
-35
-40
f1 (ppm)

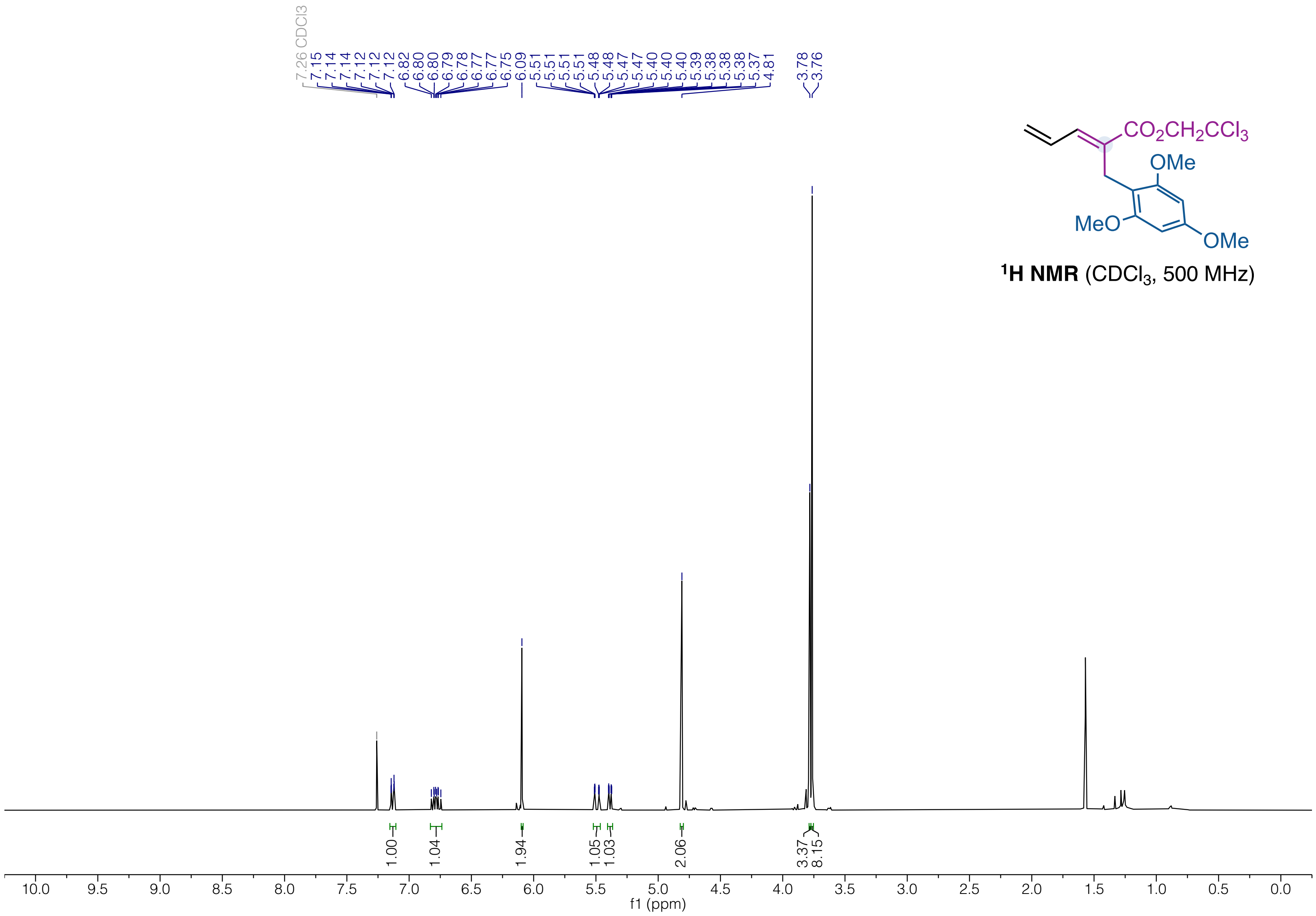
7.26 CDCl3
7.15
7.14
7.14
7.12
7.12
7.12
6.82
6.80
6.80
6.79
6.78
6.77
6.77
6.75
6.09
5.51
5.51
5.51
5.51
5.48
5.48
5.47
5.47
5.40
5.40
5.40
5.39
5.38
5.38
5.38
5.37
4.81
3.78
3.76
CO2CH2CCl3
OMe
MeO
OMe
1H NMR (CDCl3, 500 MHz)
1.00
1.04
1.94
1.05
1.03
2.06
3.37
8.15
10.0
9.5
9.0
8.5
8.0
7.5
7.0
6.5
6.0
5.5
5.0
4.5
4.0
3.5
3.0
2.5
2.0
1.5
1.0
0.5
0.0
f1 (ppm)

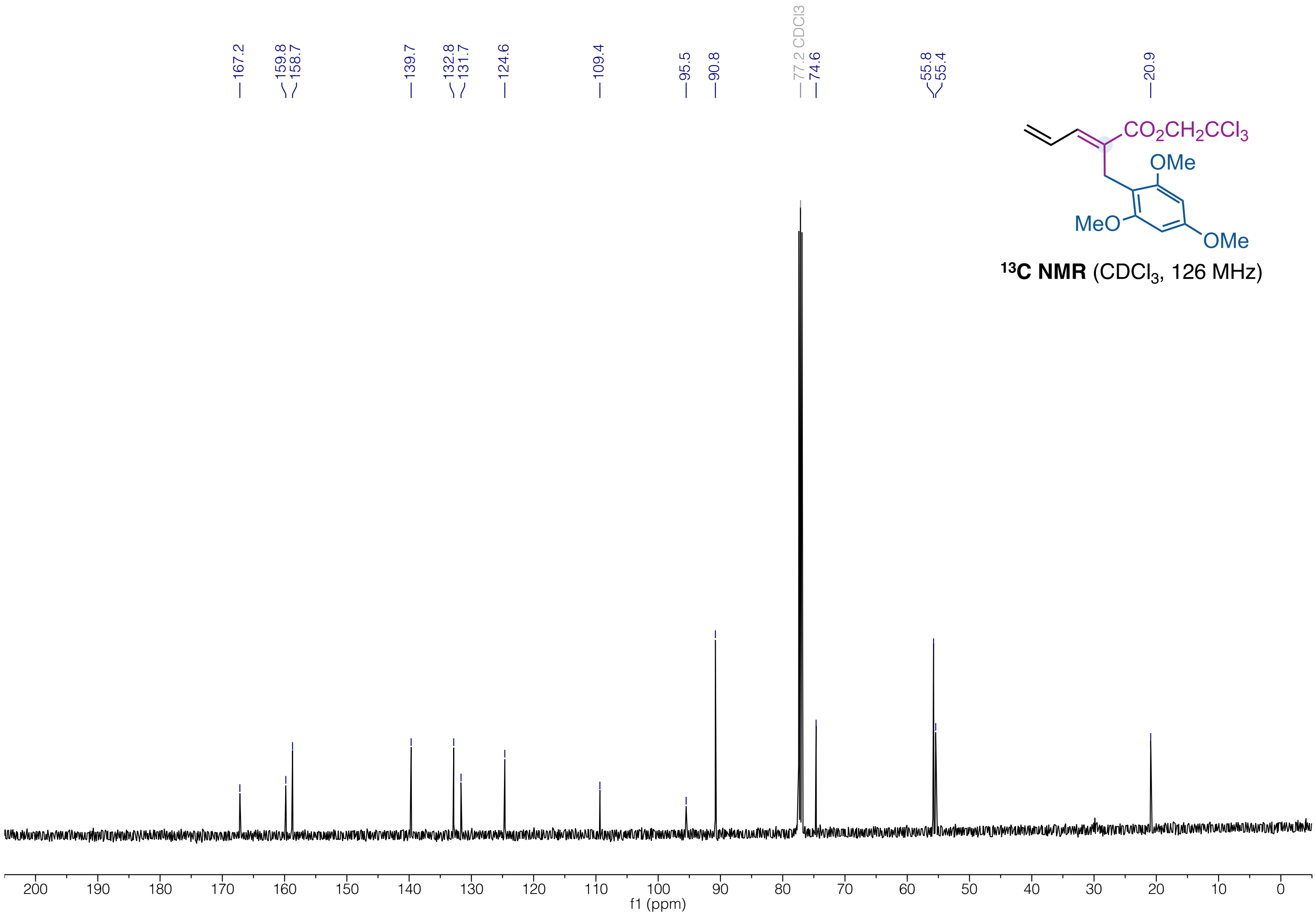

167.2
159.8
158.7
139.7
132.8
131.7
124.6
109.4
95.5
90.8
77.2 CDCl3
74.6
55.8
55.4
20.9
CO2CH2CCl3
OMe
MeO
OMe
13C NMR (CDCl3, 126 MHz)
200
190
180
170
160
150
140
130
120
110
100
90
80
70
60
50
40
30
20
10
0
f1 (ppm)

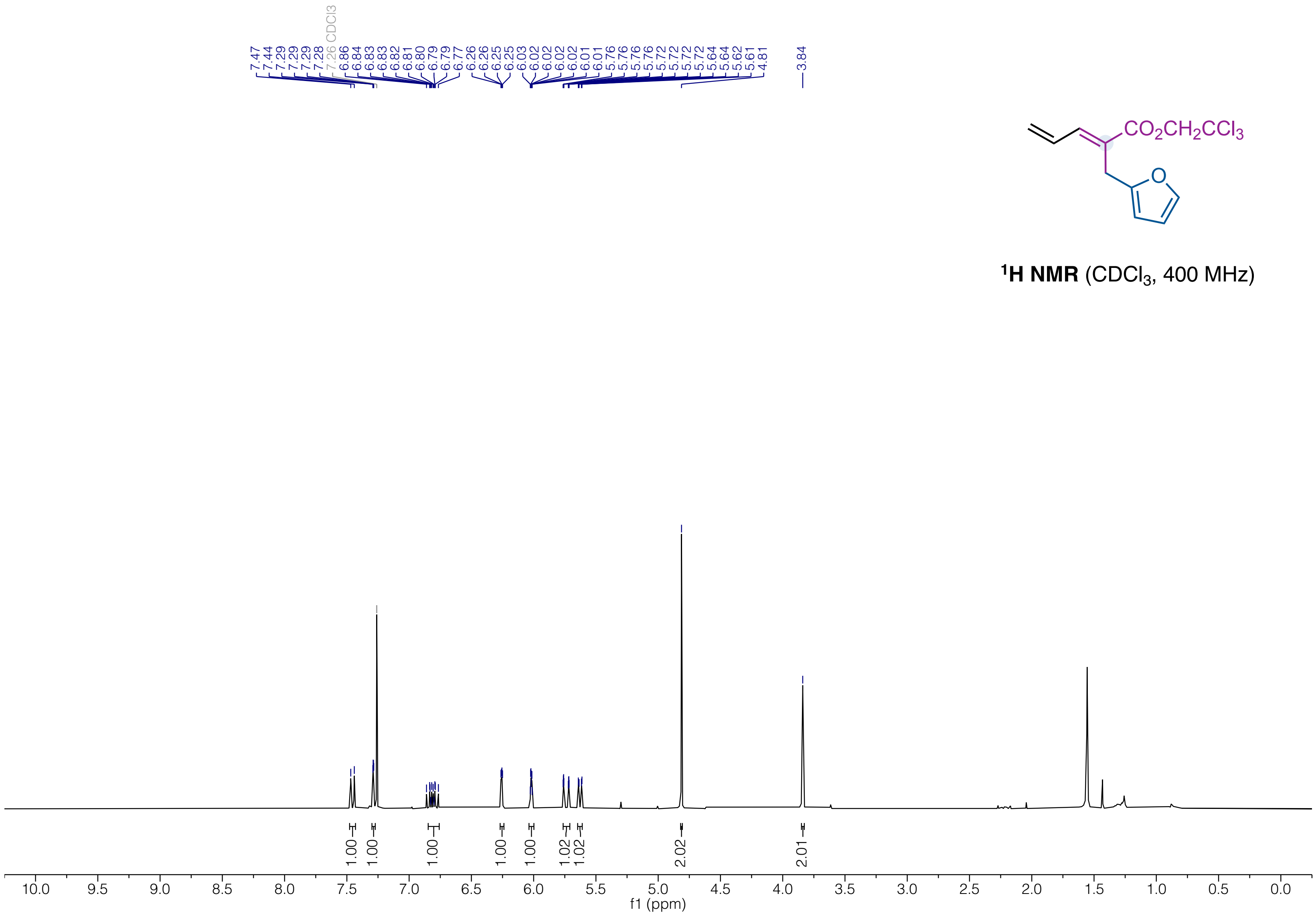

^{1}H NMR ($CDCl_3$, 400 MHz)

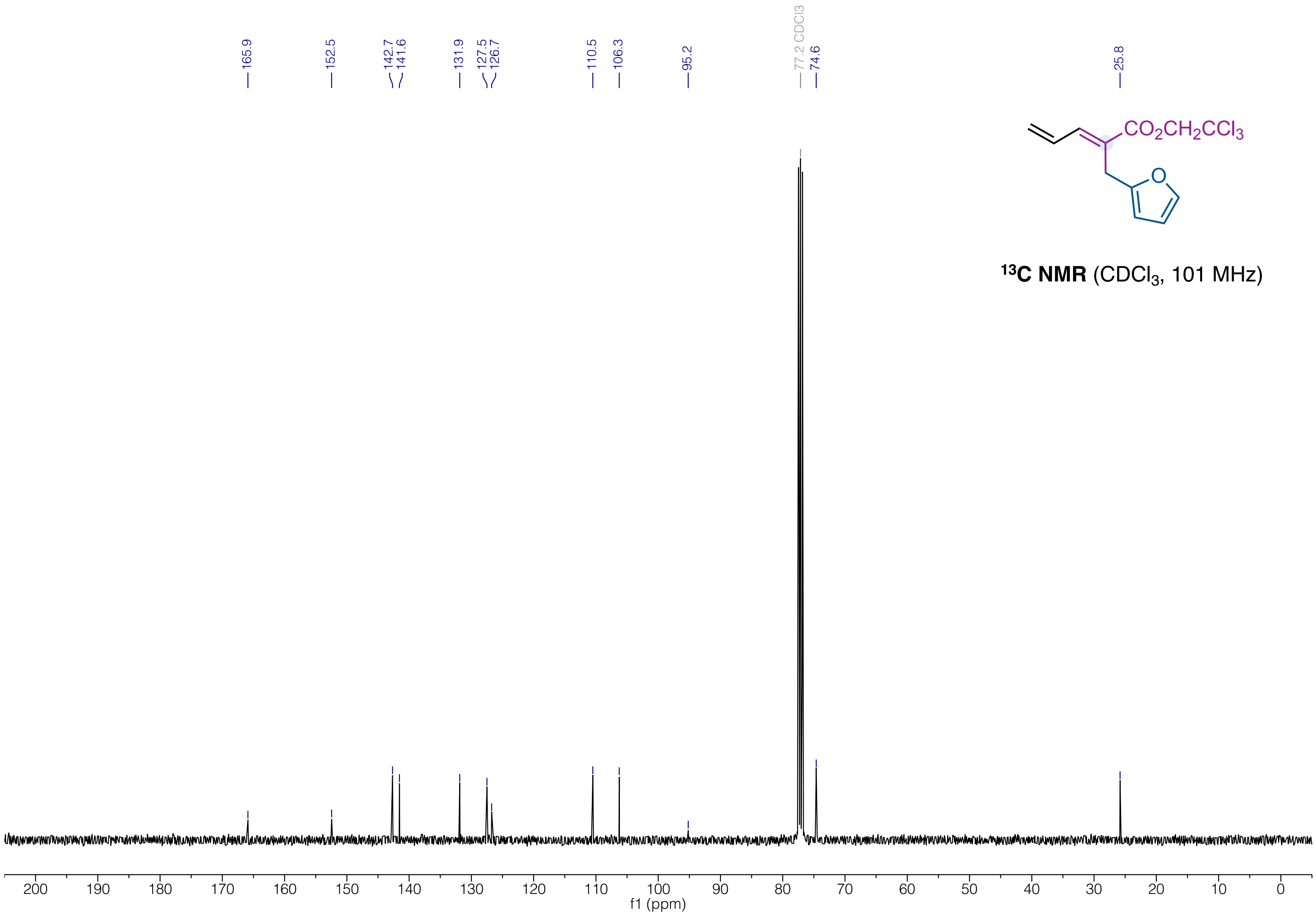

^{13}C NMR ($CDCl_3$, 101 MHz)

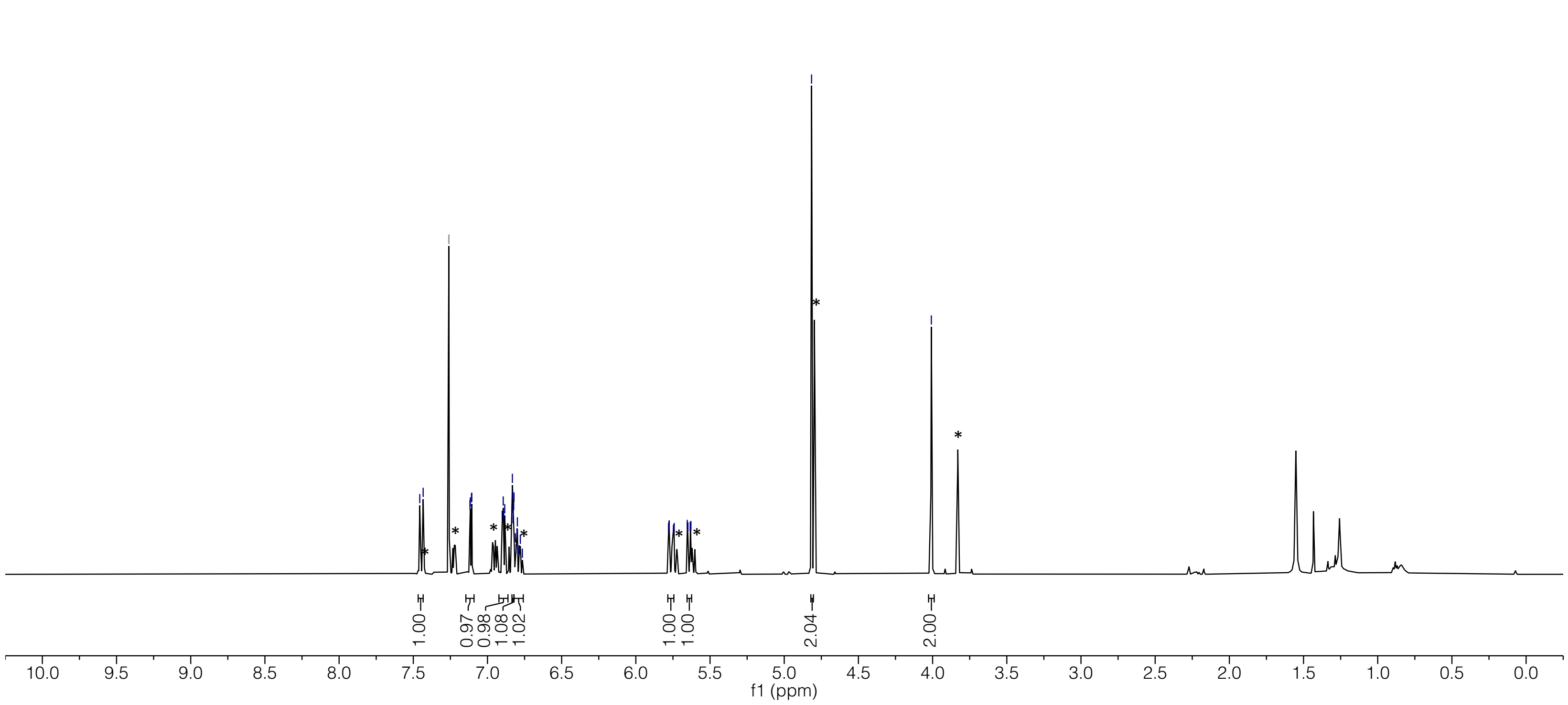

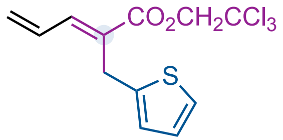

^{1}H NMR ($CDCl_3$, 500 MHz)
* minor isomer

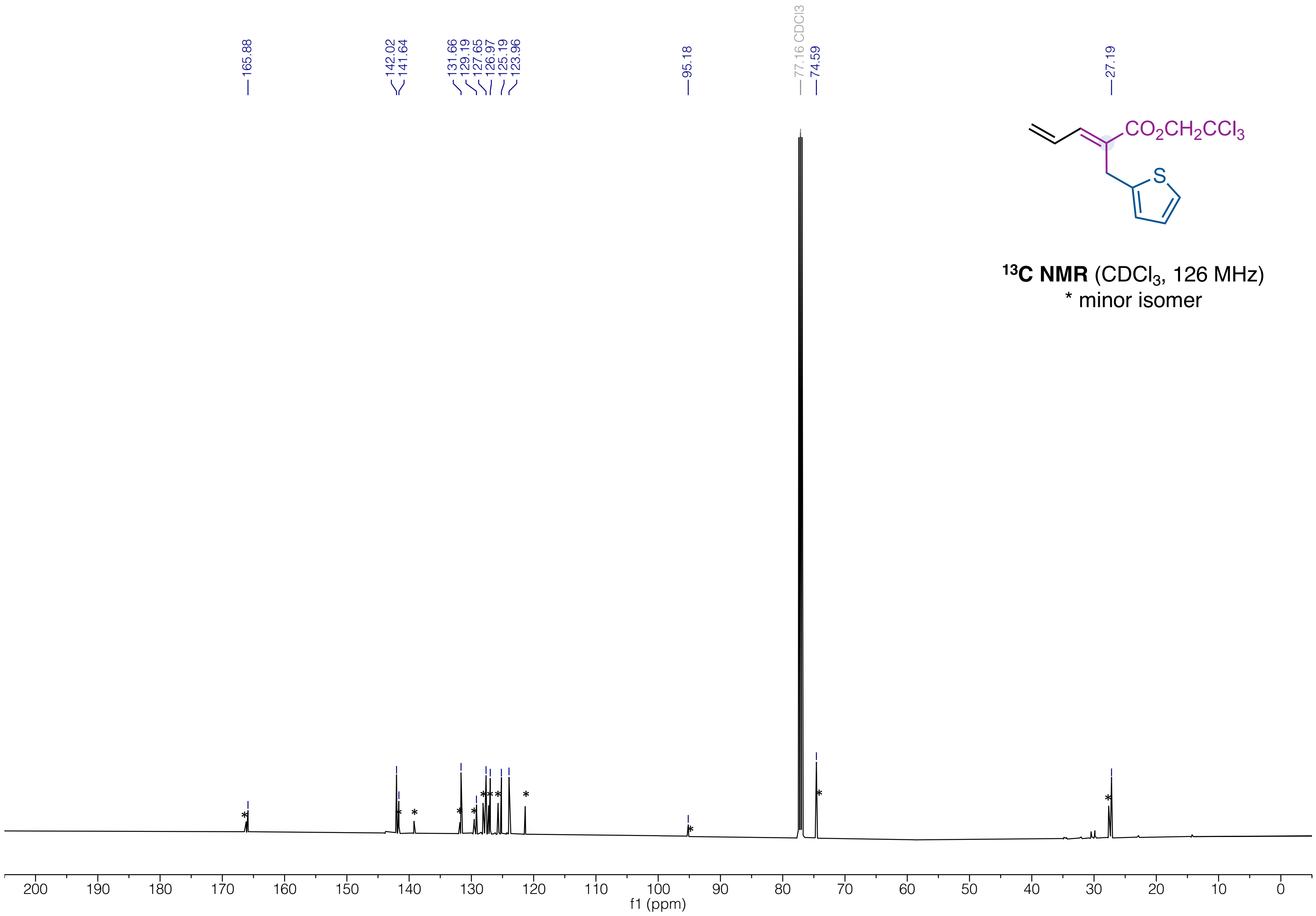

^{13}C NMR ($CDCl_3$, 126 MHz)
* minor isomer

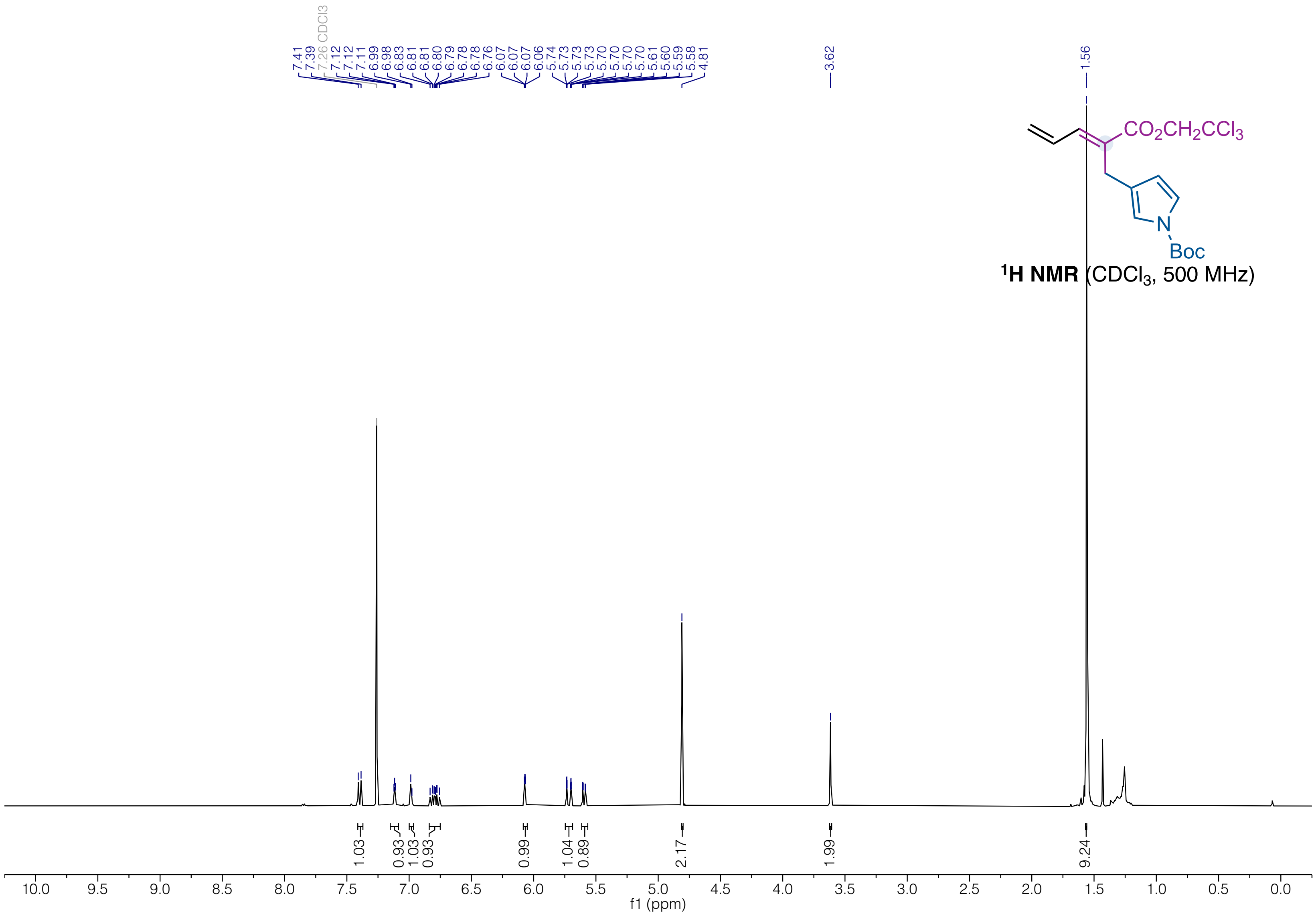

7.41
7.39
7.26 CDCl3
7.12
7.12
7.11
6.99
6.98
6.83
6.81
6.81
6.80
6.79
6.78
6.78
6.76
6.07
6.07
6.07
6.06
5.74
5.73
5.73
5.73
5.70
5.70
5.70
5.70
5.61
5.60
5.59
5.58
4.81
3.62
1.56
CO2CH2CCl3
N
Boc
1H NMR (CDCl3, 500 MHz)
1.03
0.93
1.03
0.93
0.99
1.04
0.89
2.17
1.99
9.24
10.0
9.5
9.0
8.5
8.0
7.5
7.0
6.5
6.0
5.5
5.0
4.5
4.0
3.5
3.0
2.5
2.0
1.5
1.0
0.5
0.0
f1 (ppm)

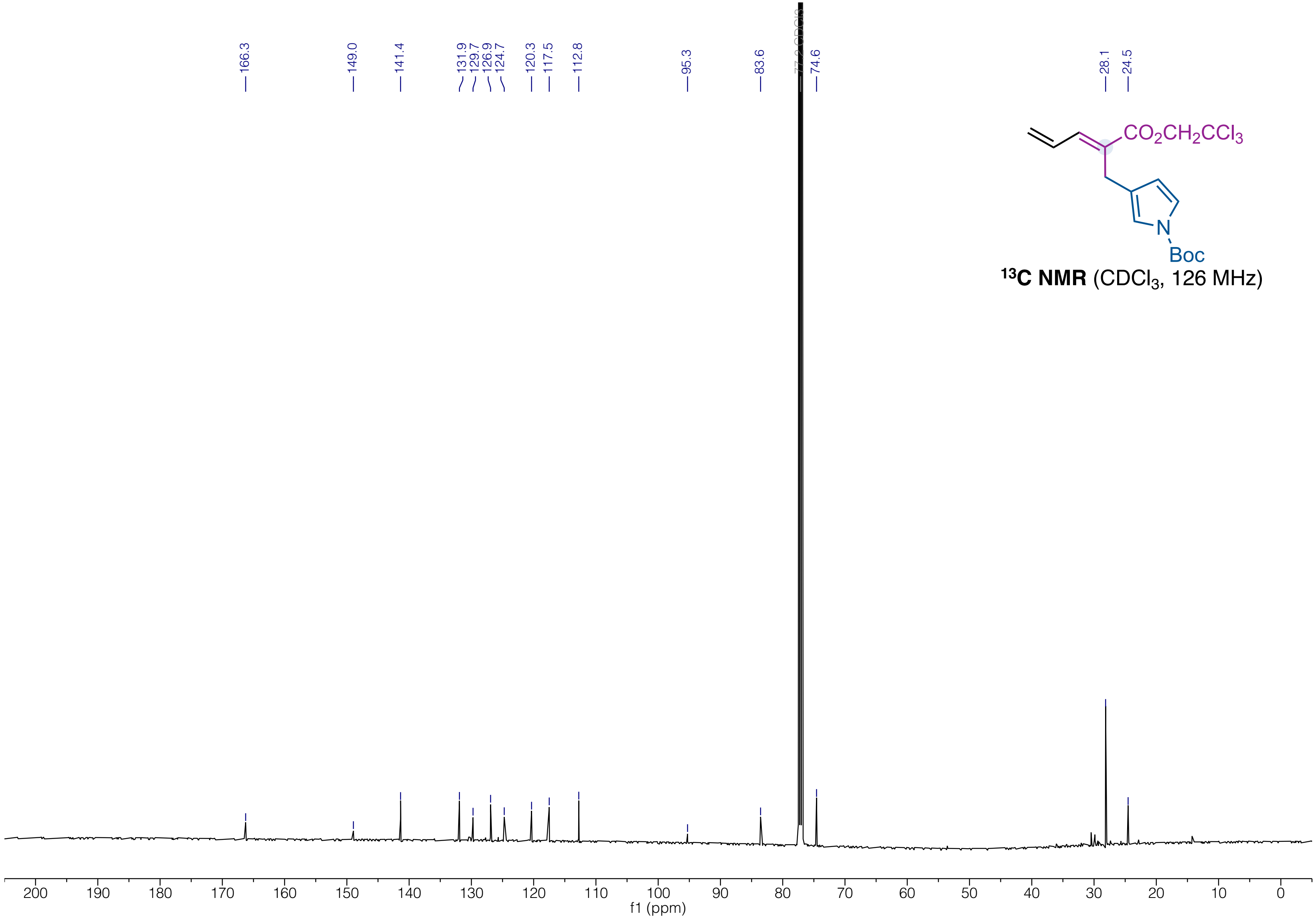
166.3
149.0
141.4
131.9
129.7
126.9
124.7
120.3
117.5
112.8
95.3
83.6
77.2 CDCl3
74.6
28.1
24.5
CO2CH2CCl3
N
Boc
13C NMR (CDCl3, 126 MHz)
200
190
180
170
160
150
140
130
120
110
100
90
80
70
60
50
40
30
20
10
0
f1 (ppm)

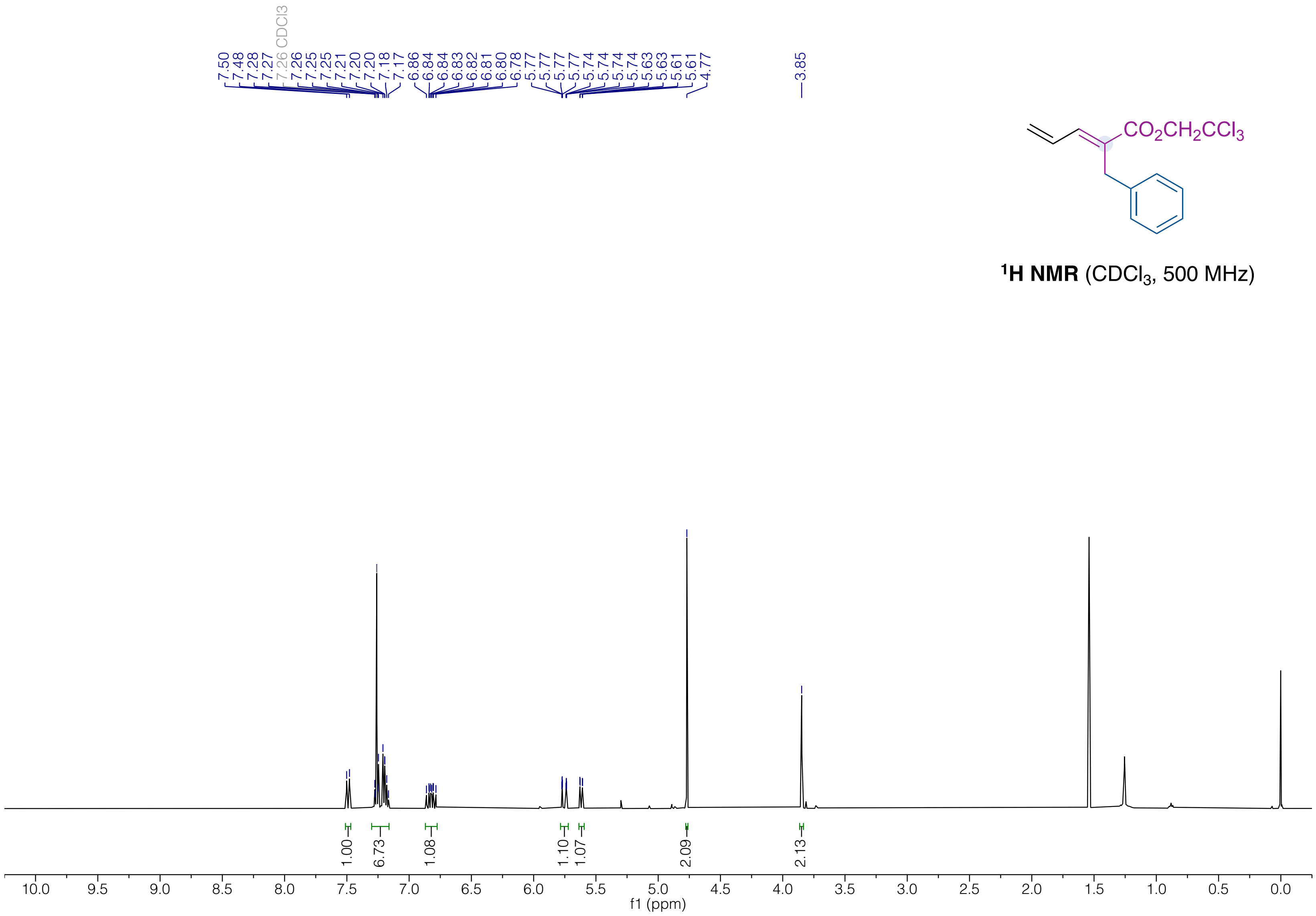

^{1}H NMR ($CDCl_3$, 500 MHz)

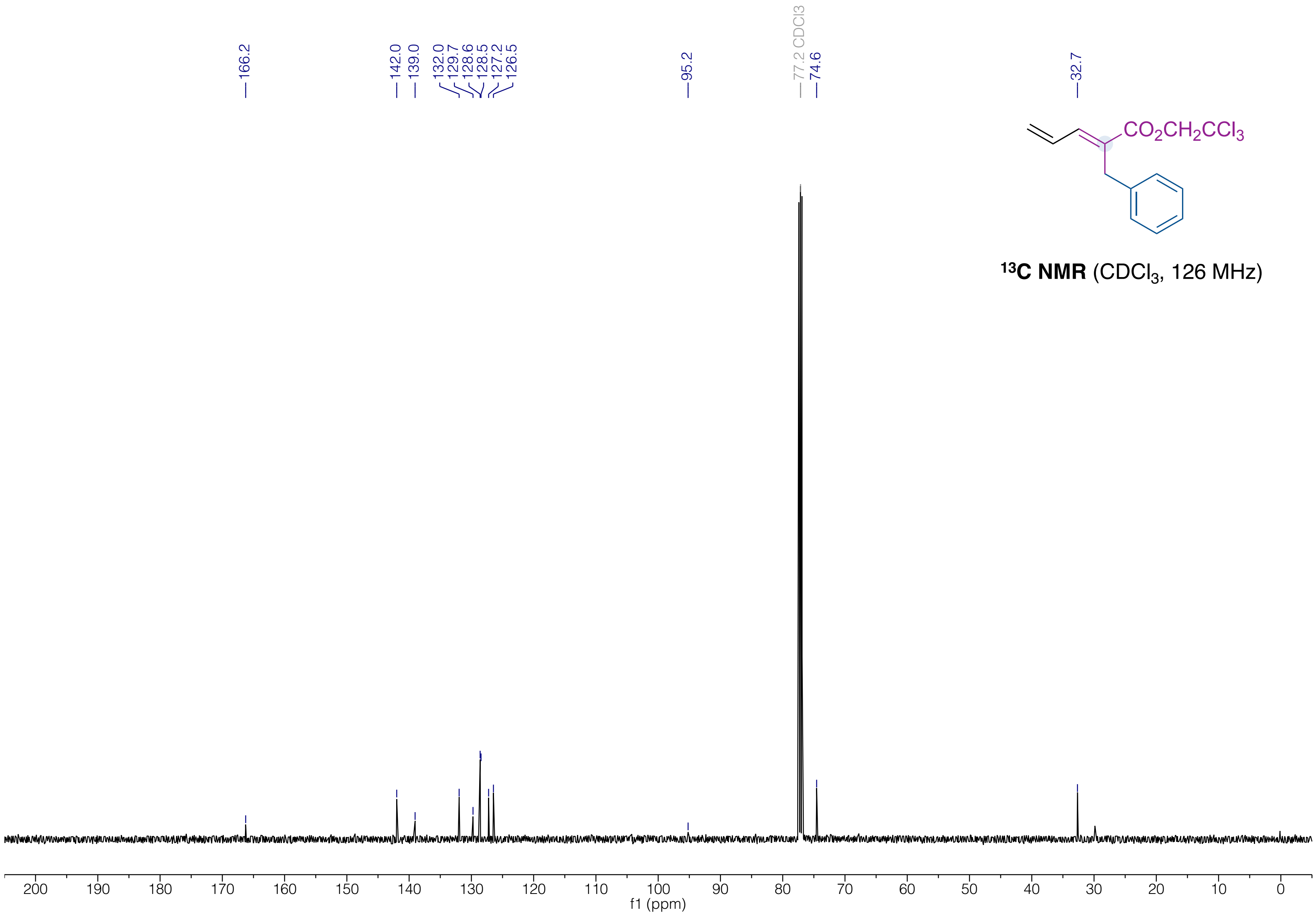

^{13}C NMR ($CDCl_3$, 126 MHz)

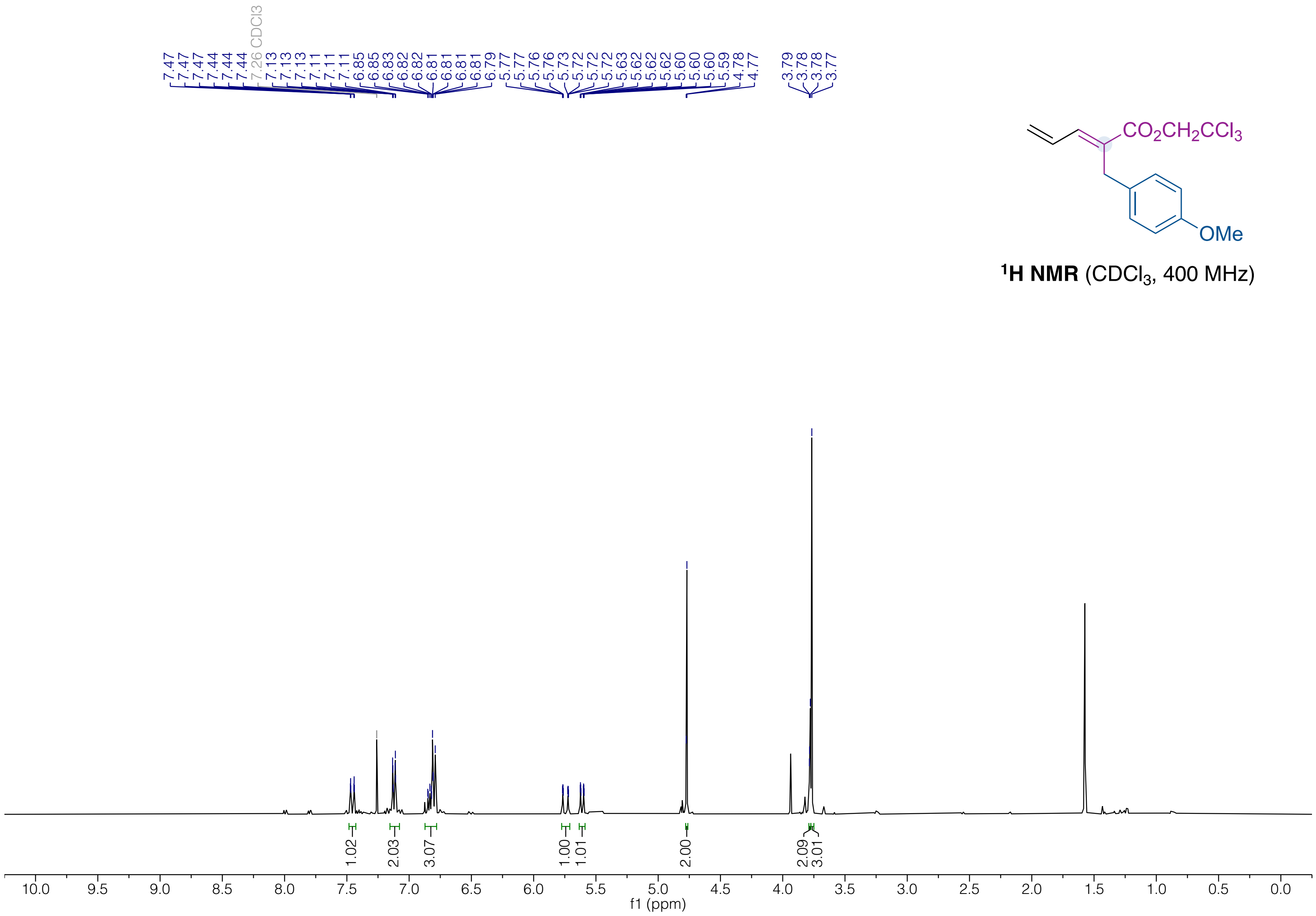

^{1}H NMR ($CDCl_3$, 400 MHz)

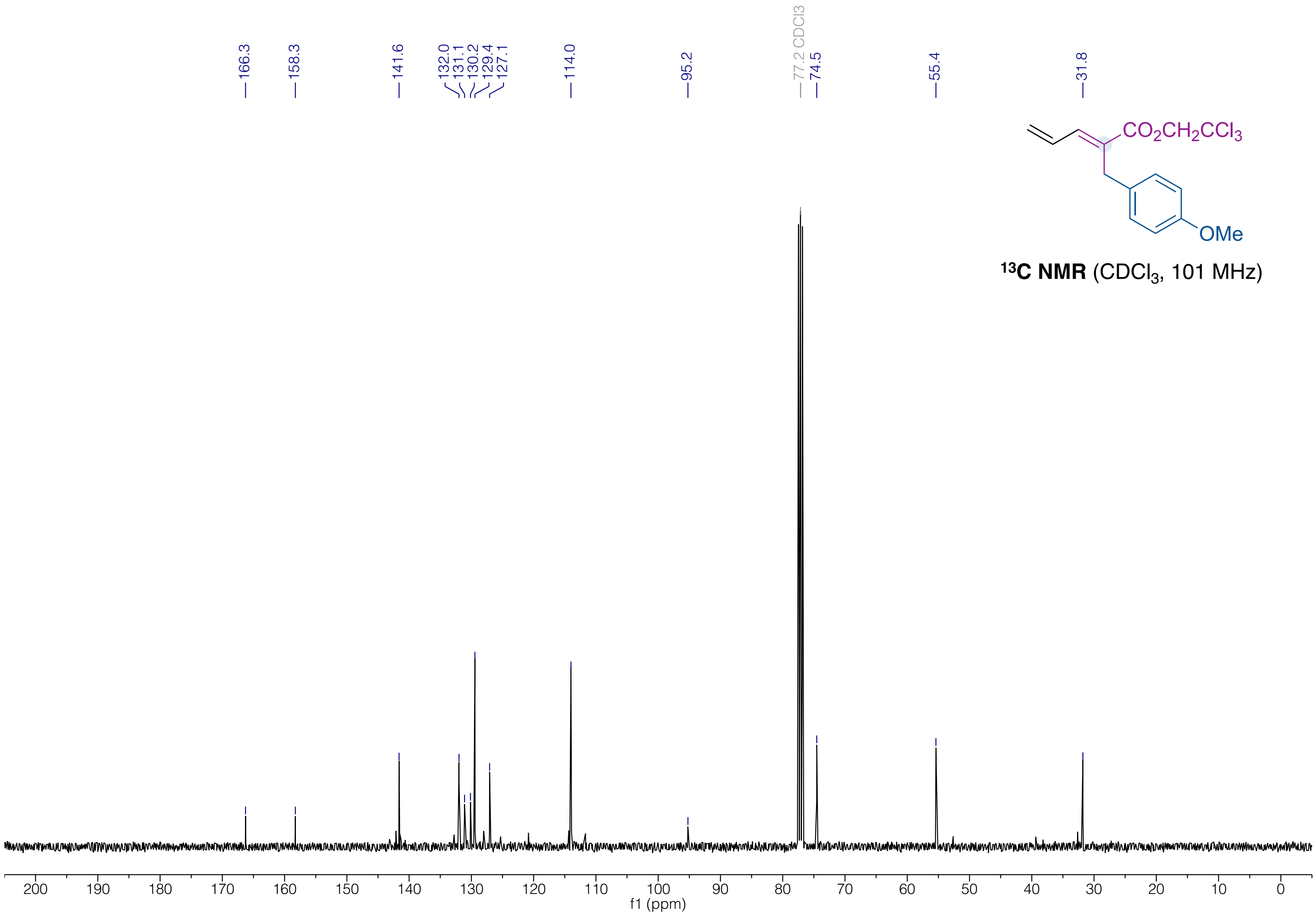

166.3
158.3
141.6
132.0
131.1
130.2
129.4
127.1
114.0
95.2
77.2 CDCl3
74.5
55.4
31.8
CO2CH2CCl3
OMe
13C NMR (CDCl3, 101 MHz)
200
190
180
170
160
150
140
130
120
110
100
90
80
70
60
50
40
30
20
10
0
f1 (ppm)

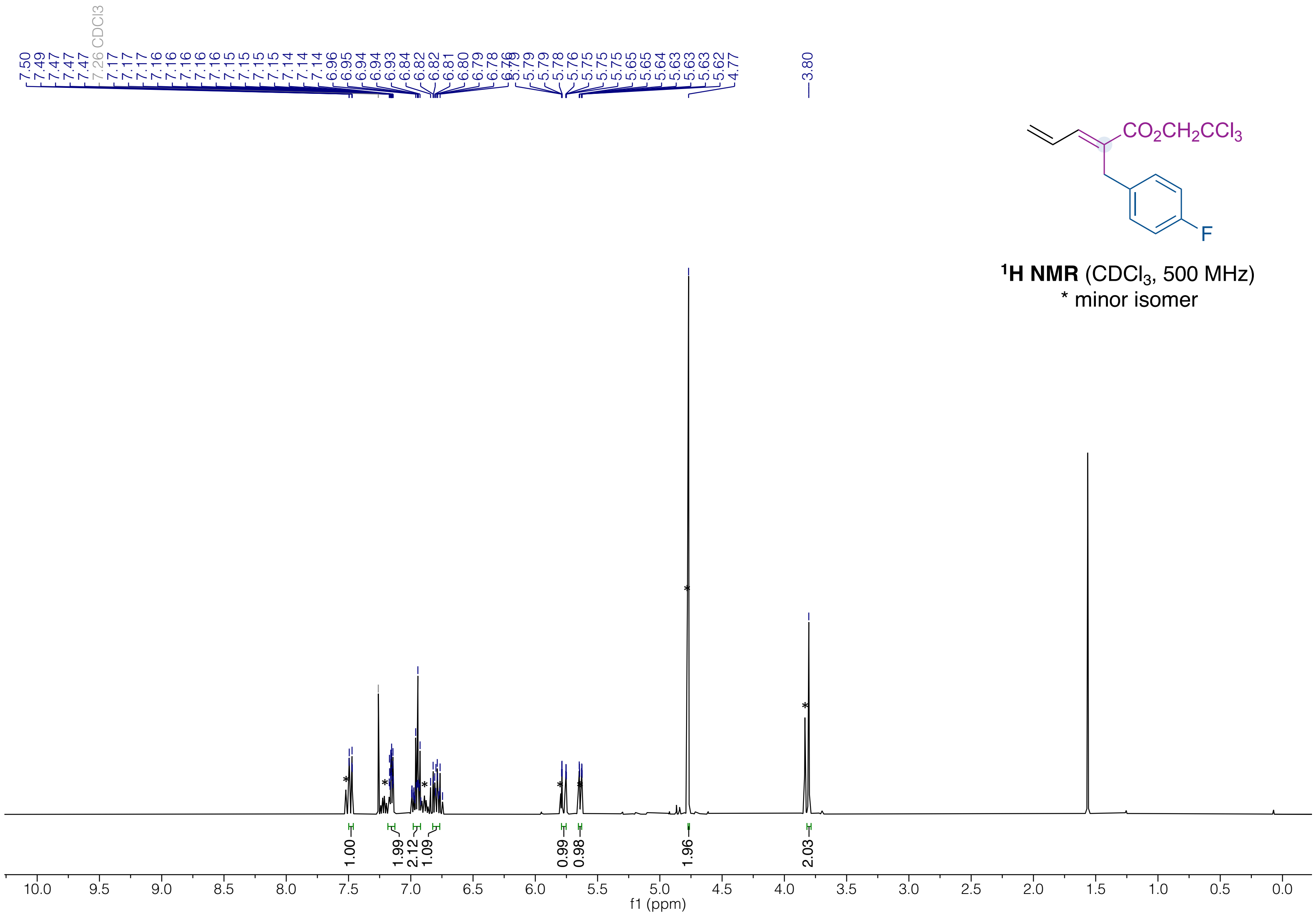
7.50 7.49 7.47 7.47 7.47 7.26 CDCl3 7.17 7.17 7.17 7.16 7.16 7.16 7.16 7.15 7.15 7.15 7.14 7.14 7.14 6.96 6.95 6.94 6.94 6.93 6.84 6.82 6.82 6.81 6.80 6.79 6.78 6.76 5.79 5.79 5.78 5.76 5.75 5.75 5.75 5.65 5.65 5.64 5.63 5.63 5.63 5.62 4.77
3.80
CO2CH2CCl3
F
1H NMR (CDCl3, 500 MHz)
* minor isomer
1.00 1.99 2.12 1.09 0.99 0.98 1.96 2.03
10.0 9.5 9.0 8.5 8.0 7.5 7.0 6.5 6.0 5.5 5.0 4.5 4.0 3.5 3.0 2.5 2.0 1.5 1.0 0.5 0.0
f1 (ppm)

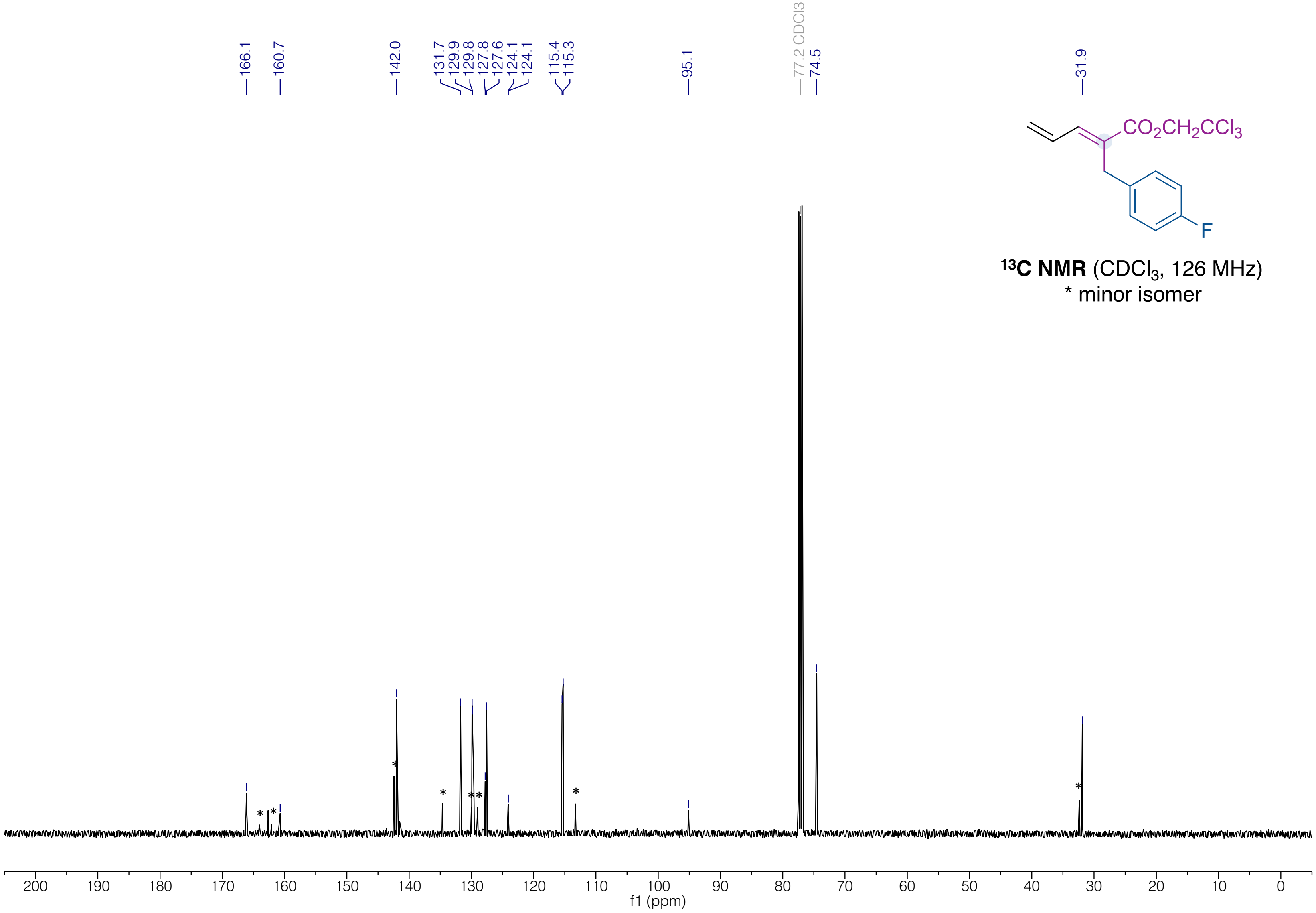
166.1
160.7
142.0
131.7
129.9
129.8
127.8
127.6
124.1
124.1
115.4
115.3
95.1
77.2 CDCl3
74.5
31.9
$CO_2CH_2CCl_3$
F
^{13}C NMR ($CDCl_3$, 126 MHz)
* minor isomer
f1 (ppm)

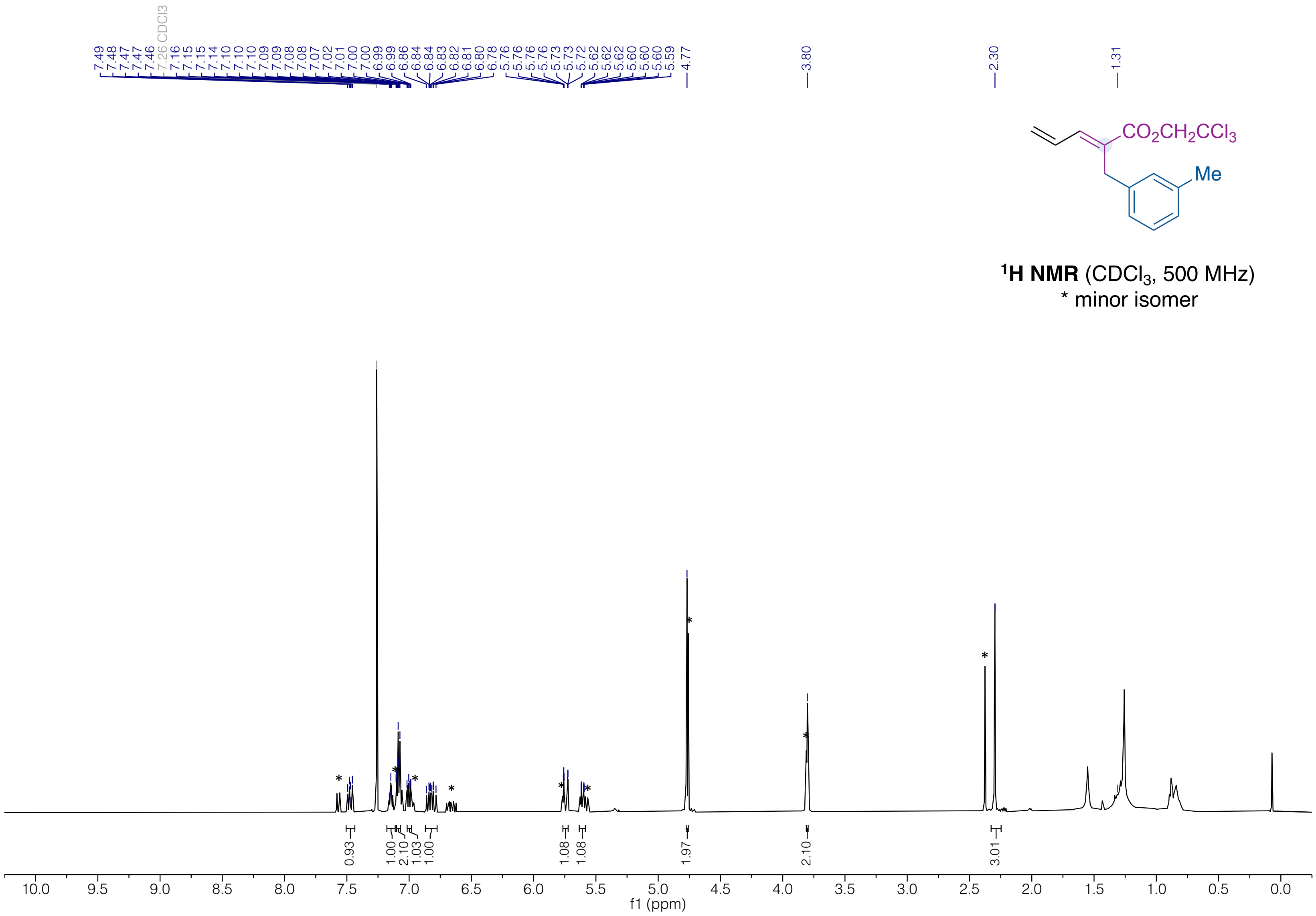

¹H NMR (CDCl$_3$, 500 MHz)
* minor isomer

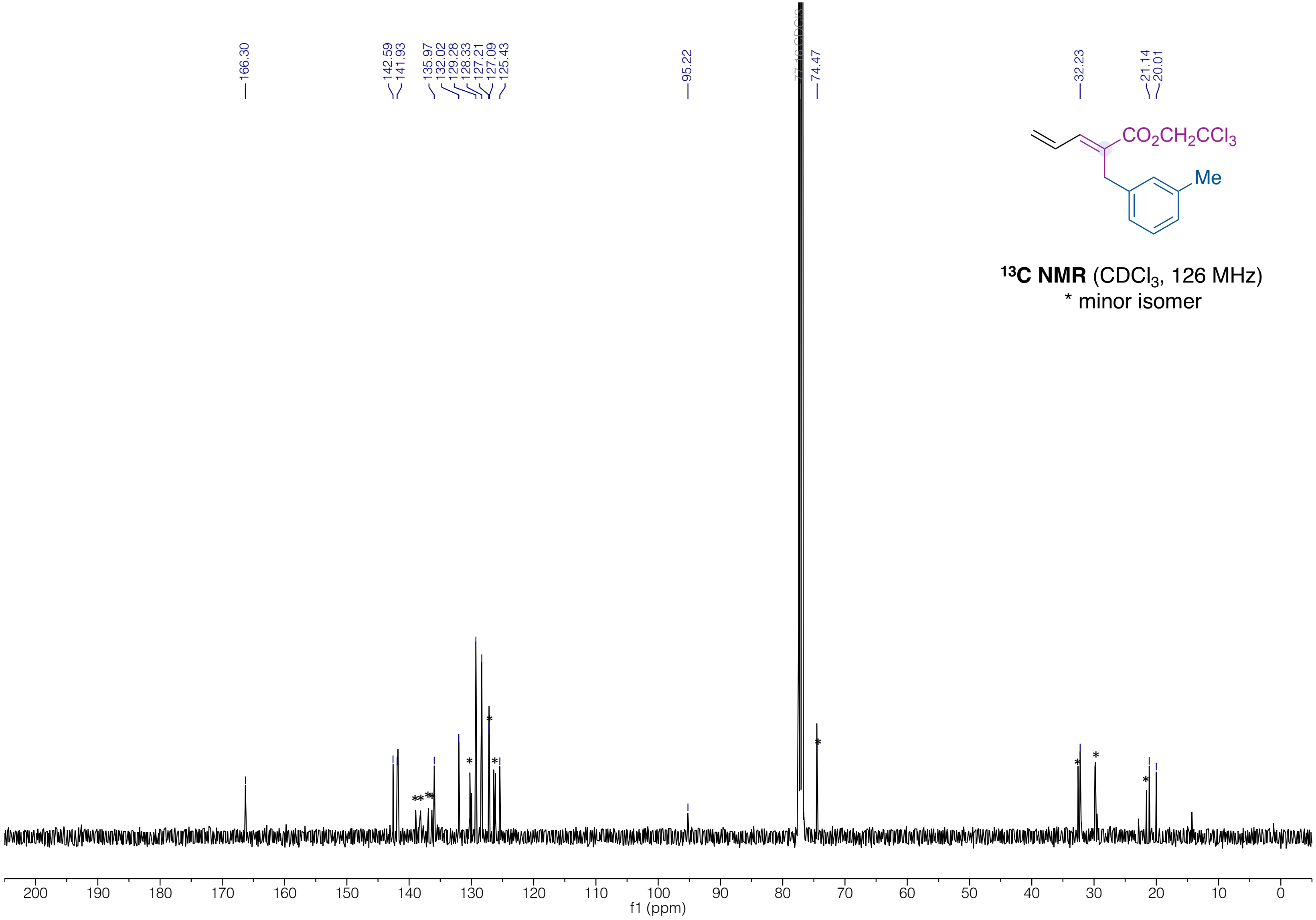

^{13}C NMR ($CDCl_3$, 126 MHz)
* minor isomer

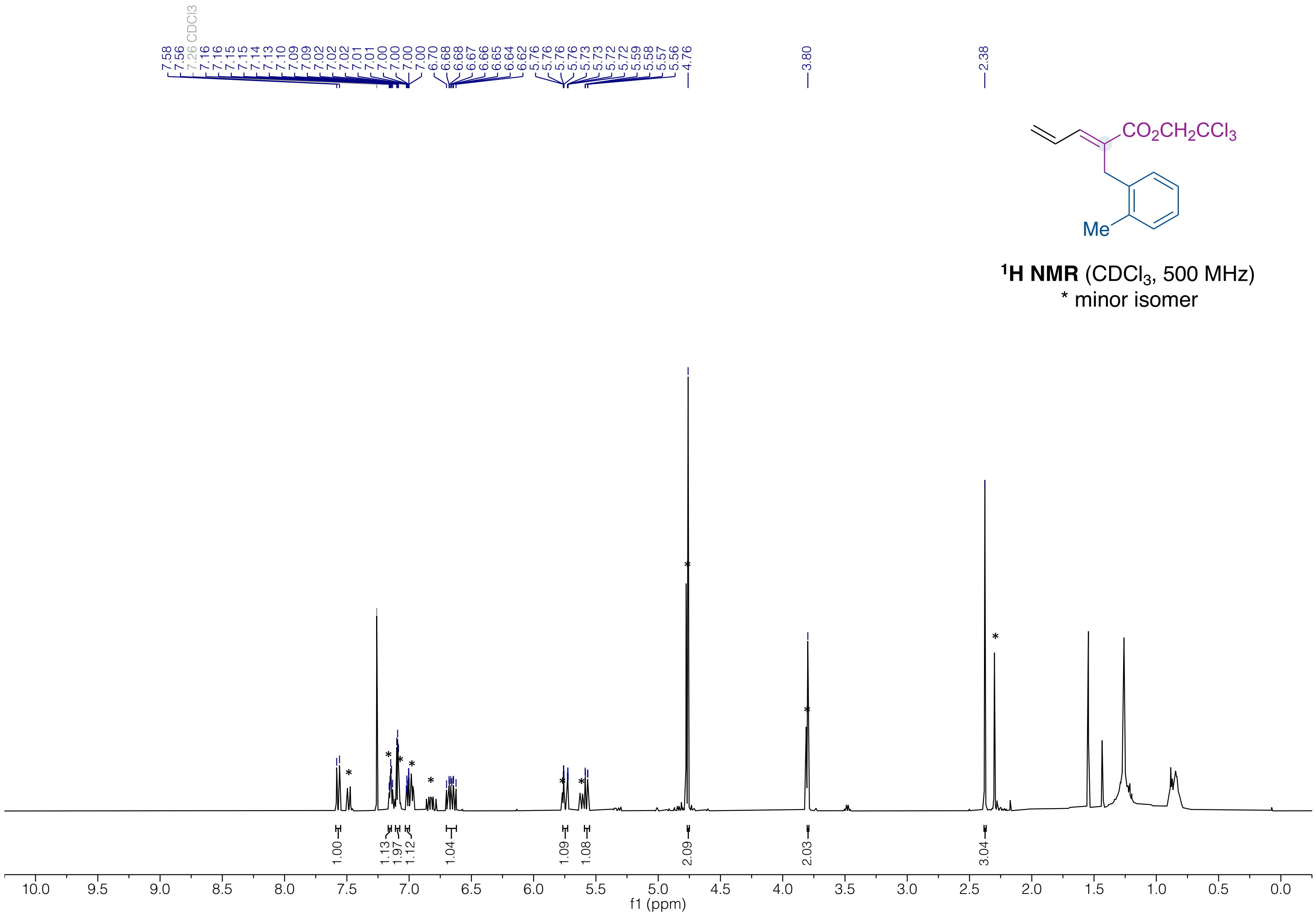

^{1}H NMR ($CDCl_3$, 500 MHz)
* minor isomer

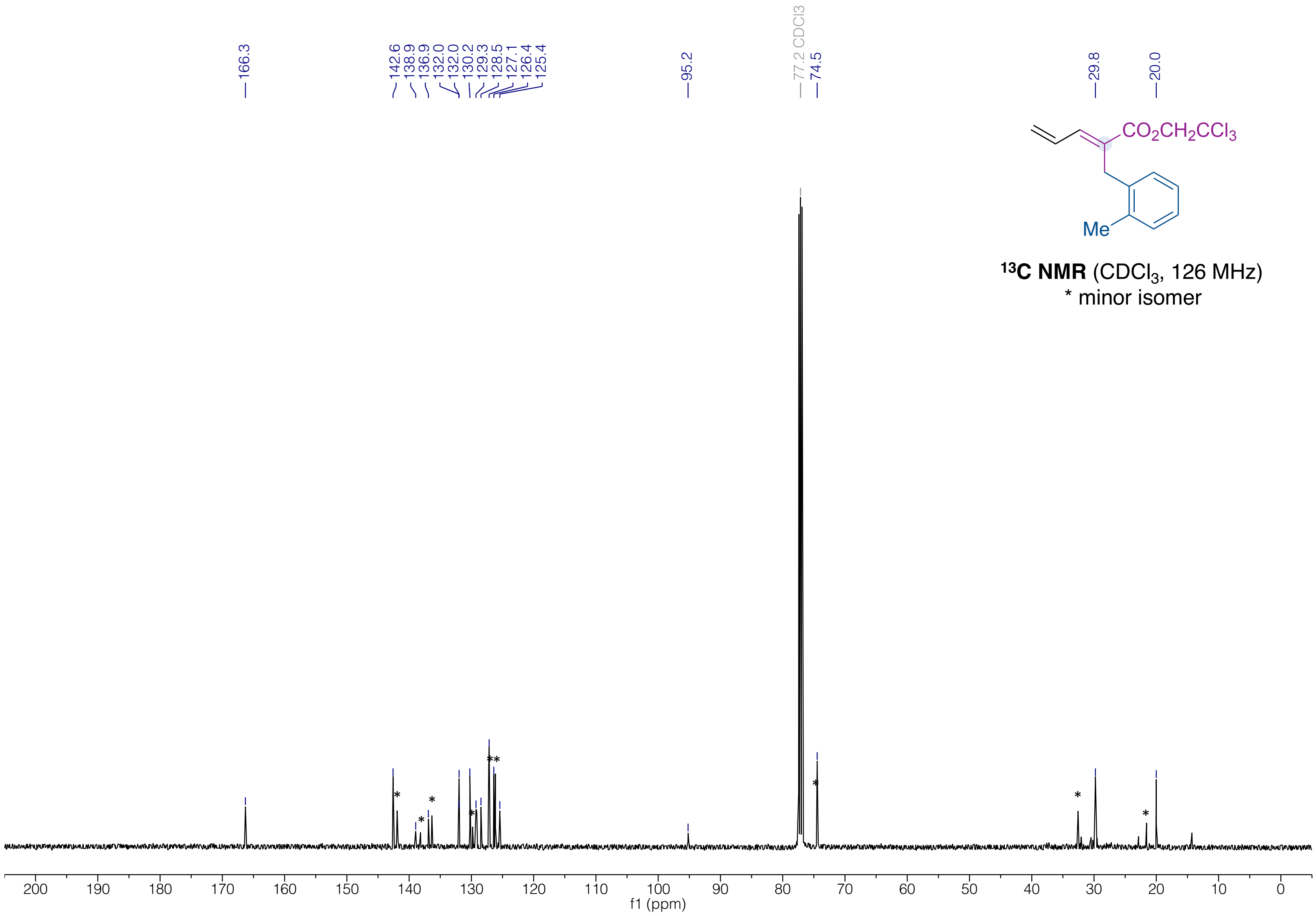
166.3
142.6
138.9
136.9
132.0
132.0
130.2
129.3
128.5
127.1
126.4
125.4
95.2
77.2 CDCl3
74.5
29.8
20.0
$CO_2CH_2CCl_3$
Me
^{13}C NMR ($CDCl_3$, 126 MHz)
* minor isomer
f1 (ppm)

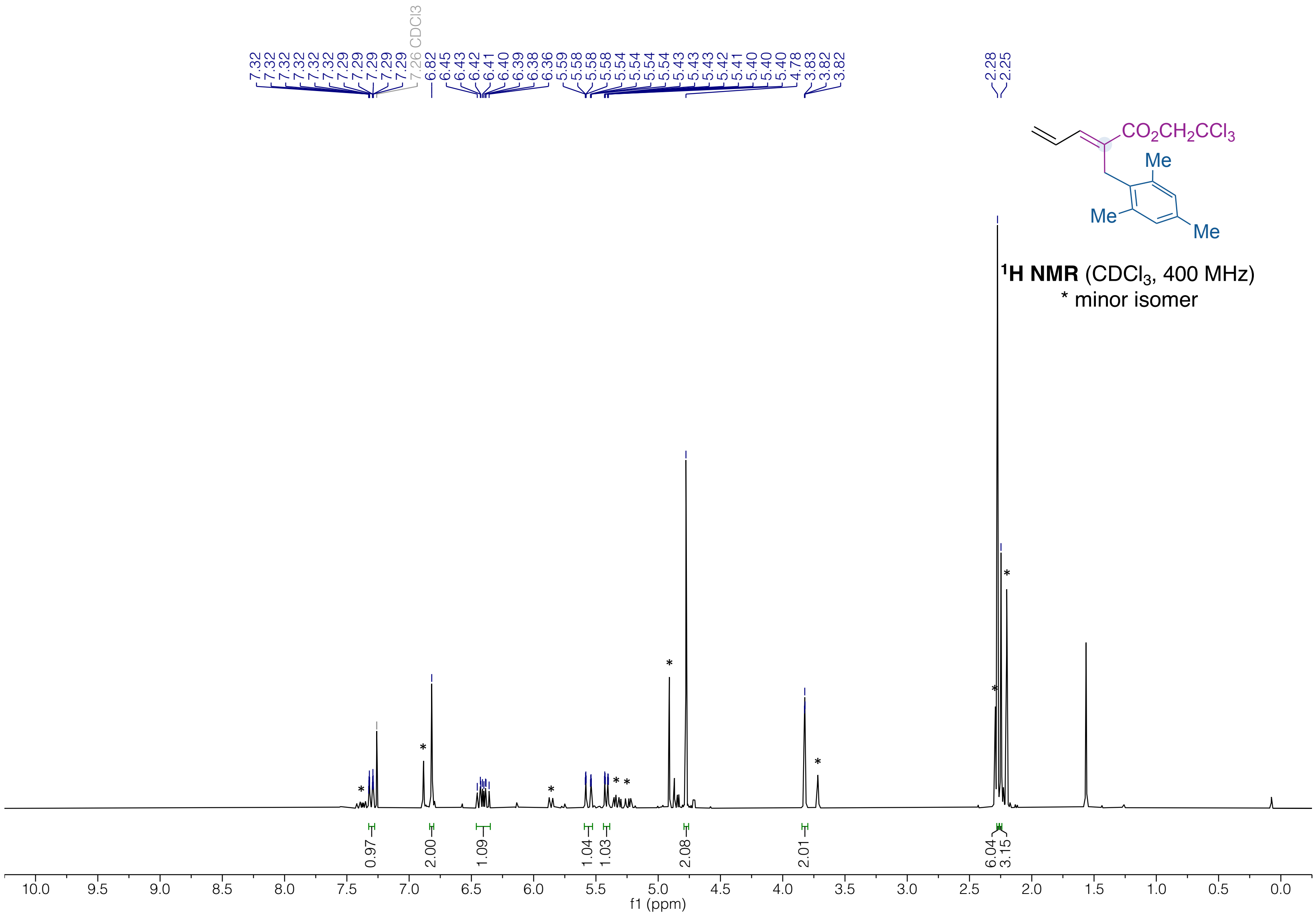

7.32
7.32
7.32
7.32
7.32
7.32
7.29
7.29
7.29
7.29
7.29
7.26 CDCl3
6.82
6.45
6.43
6.42
6.41
6.40
6.39
6.38
6.36
5.59
5.58
5.58
5.58
5.54
5.54
5.54
5.54
5.43
5.43
5.43
5.42
5.41
5.40
5.40
5.40
4.78
3.83
3.82
3.82
2.28
2.25
CO2CH2CCl3
Me
Me
Me
1H NMR (CDCl3, 400 MHz)
* minor isomer
0.97
2.00
1.09
1.04
1.03
2.08
2.01
6.04
3.15
10.0
9.5
9.0
8.5
8.0
7.5
7.0
6.5
6.0
5.5
5.0
4.5
4.0
3.5
3.0
2.5
2.0
1.5
1.0
0.5
0.0
f1 (ppm)

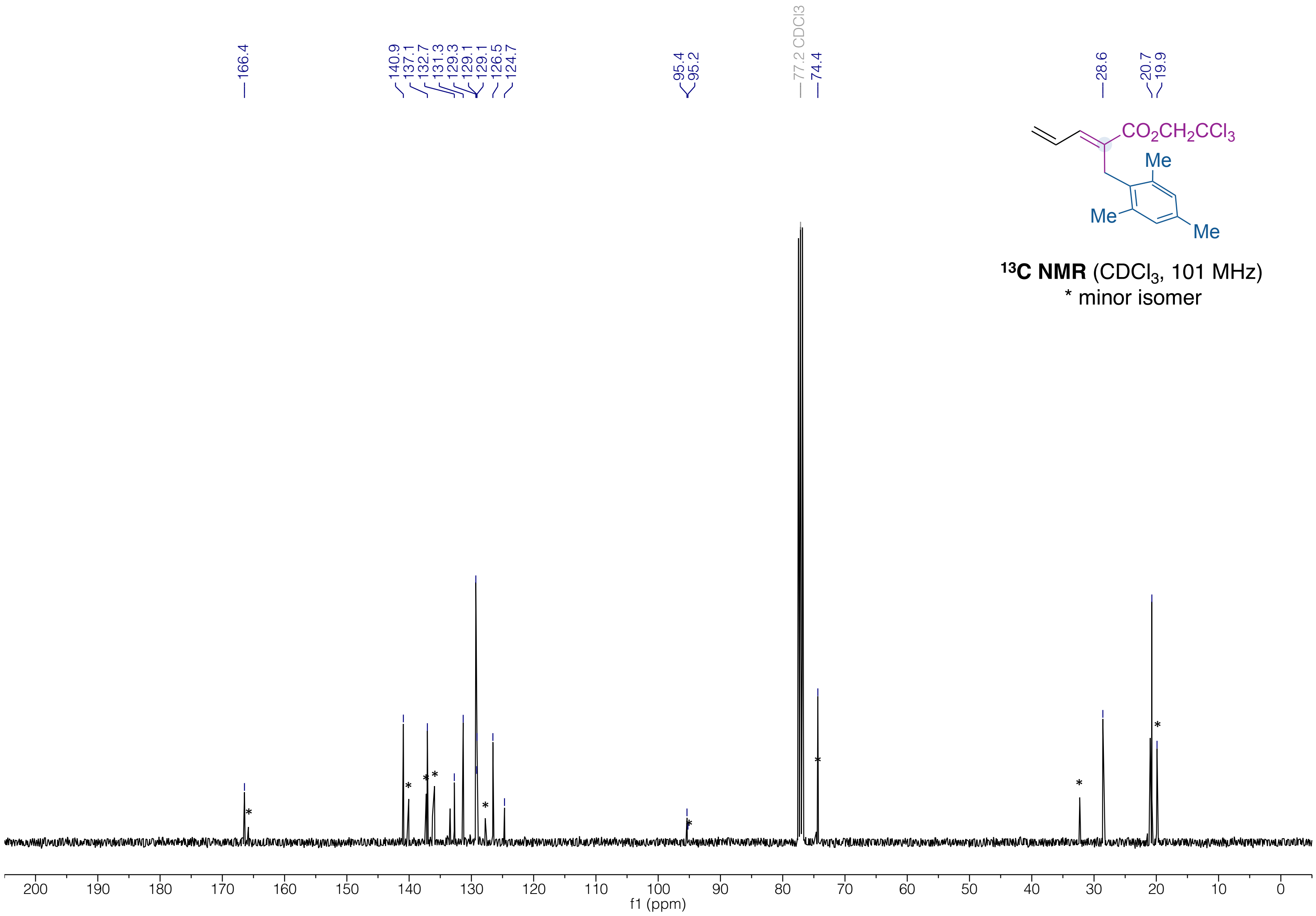
166.4
140.9
137.1
132.7
131.3
129.3
129.1
129.1
126.5
124.7
95.4
95.2
77.2 CDCl3
74.4
28.6
20.7
19.9
CO2CH2CCl3
Me
Me
Me
13C NMR (CDCl3, 101 MHz)
* minor isomer
f1 (ppm)

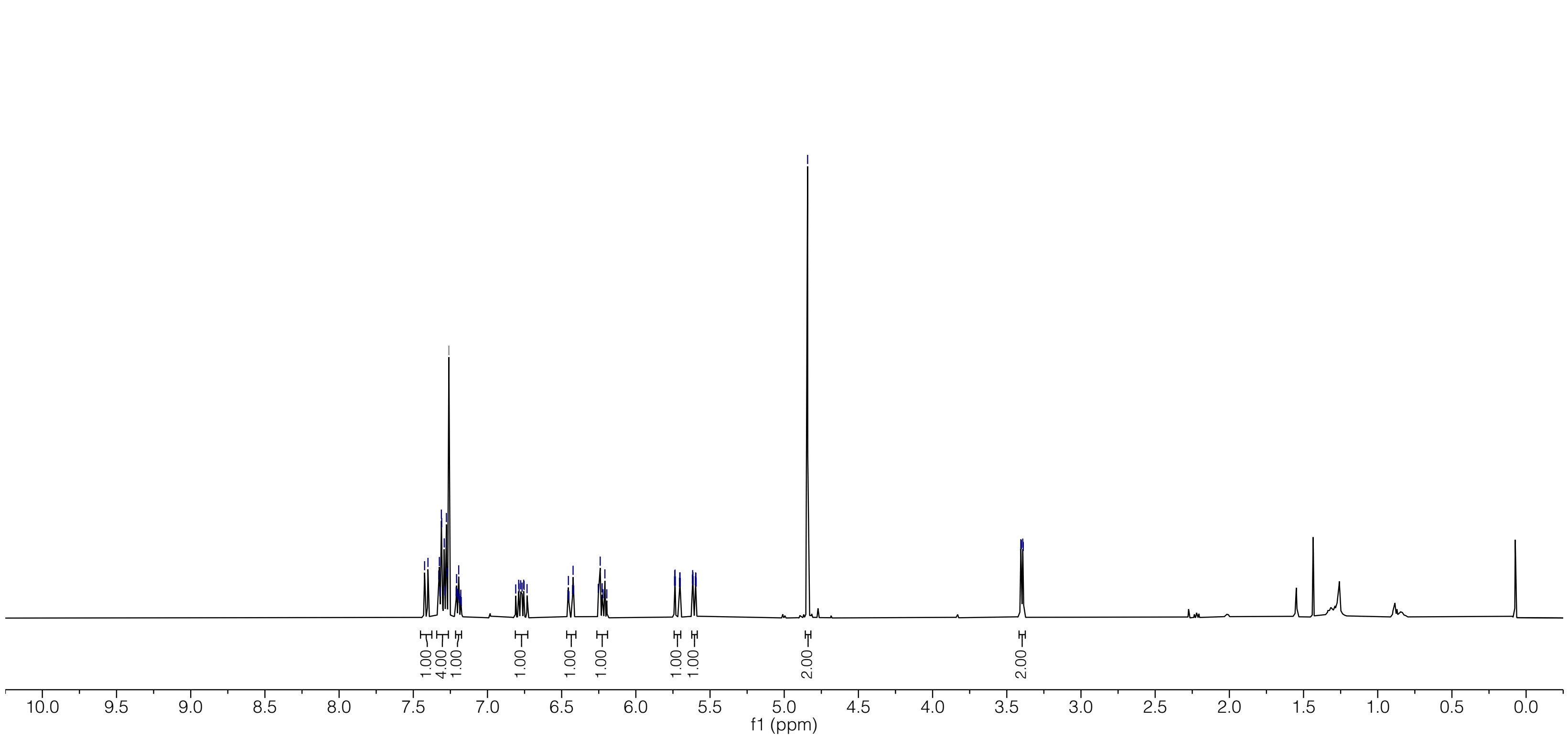

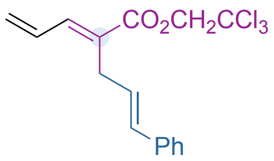

¹H NMR ($CDCl_3$, 500 MHz)

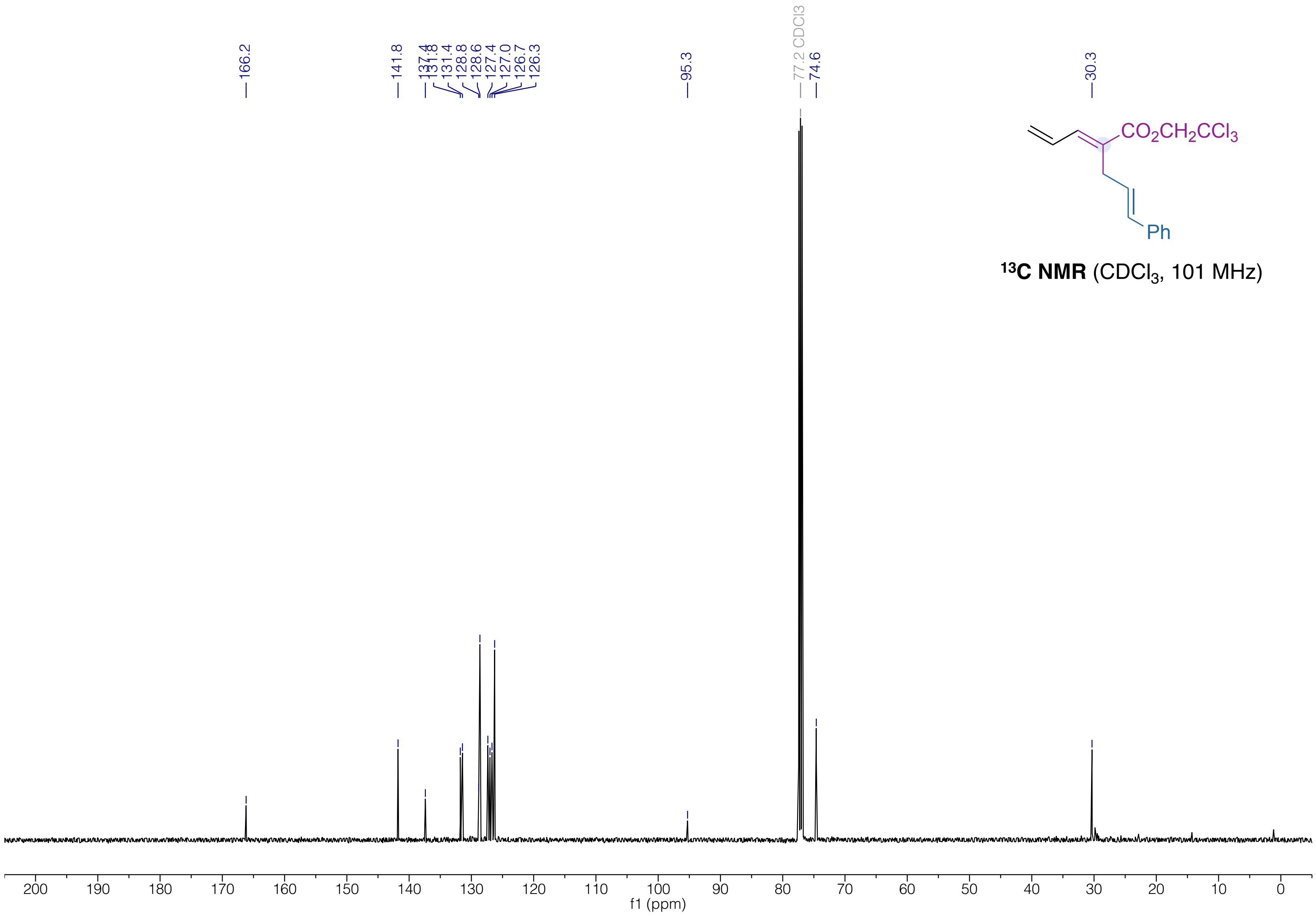
166.2
141.8
137.4
131.8
131.4
128.8
128.6
127.4
127.0
126.7
126.3
95.3
77.2 CDCl3
74.6
30.3
CO2CH2CCl3
Ph
13C NMR (CDCl3, 101 MHz)
200
190
180
170
160
150
140
130
120
110
100
90
80
70
60
50
40
30
20
10
0
f1 (ppm)

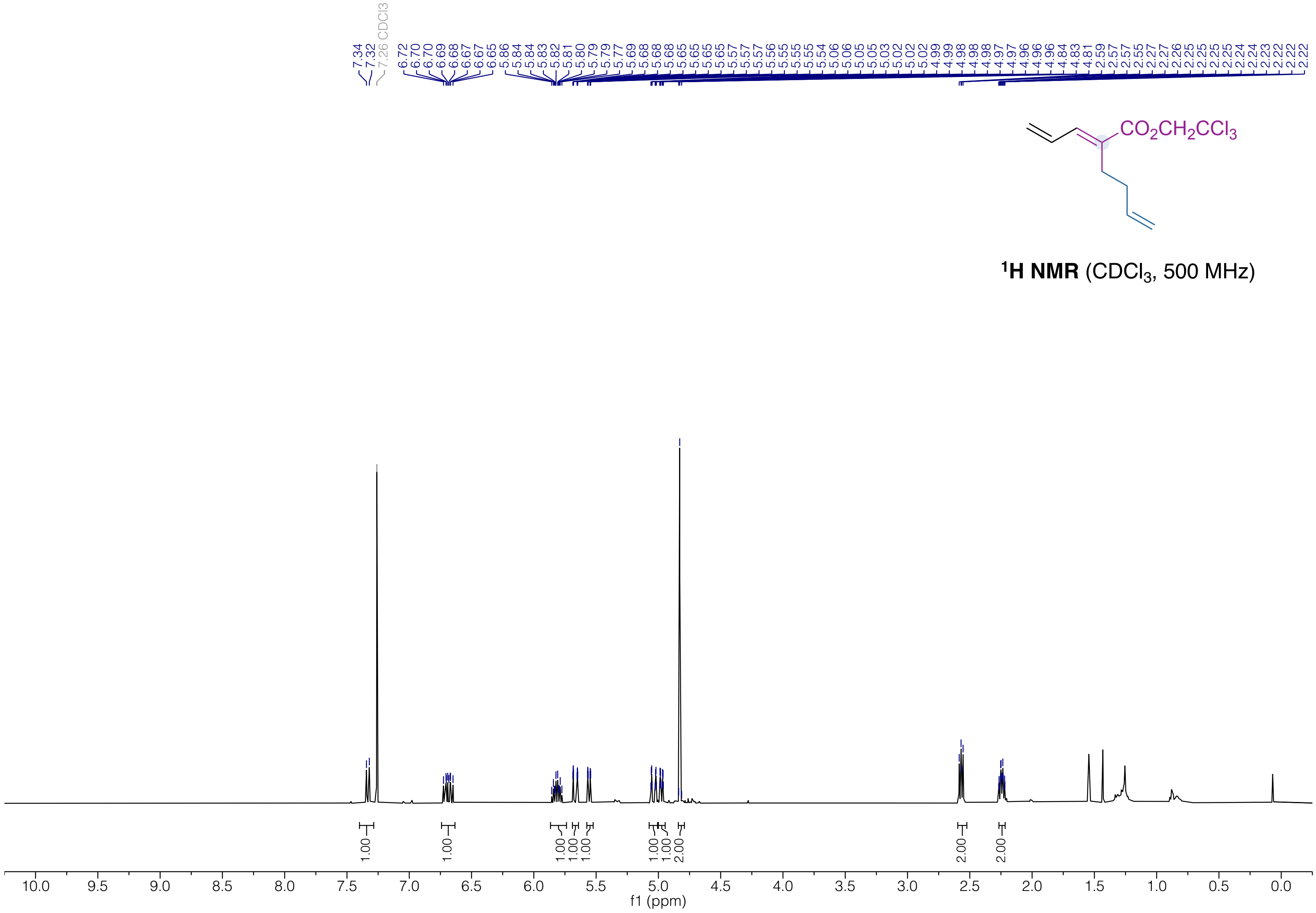

^{1}H NMR ($CDCl_3$, 500 MHz)

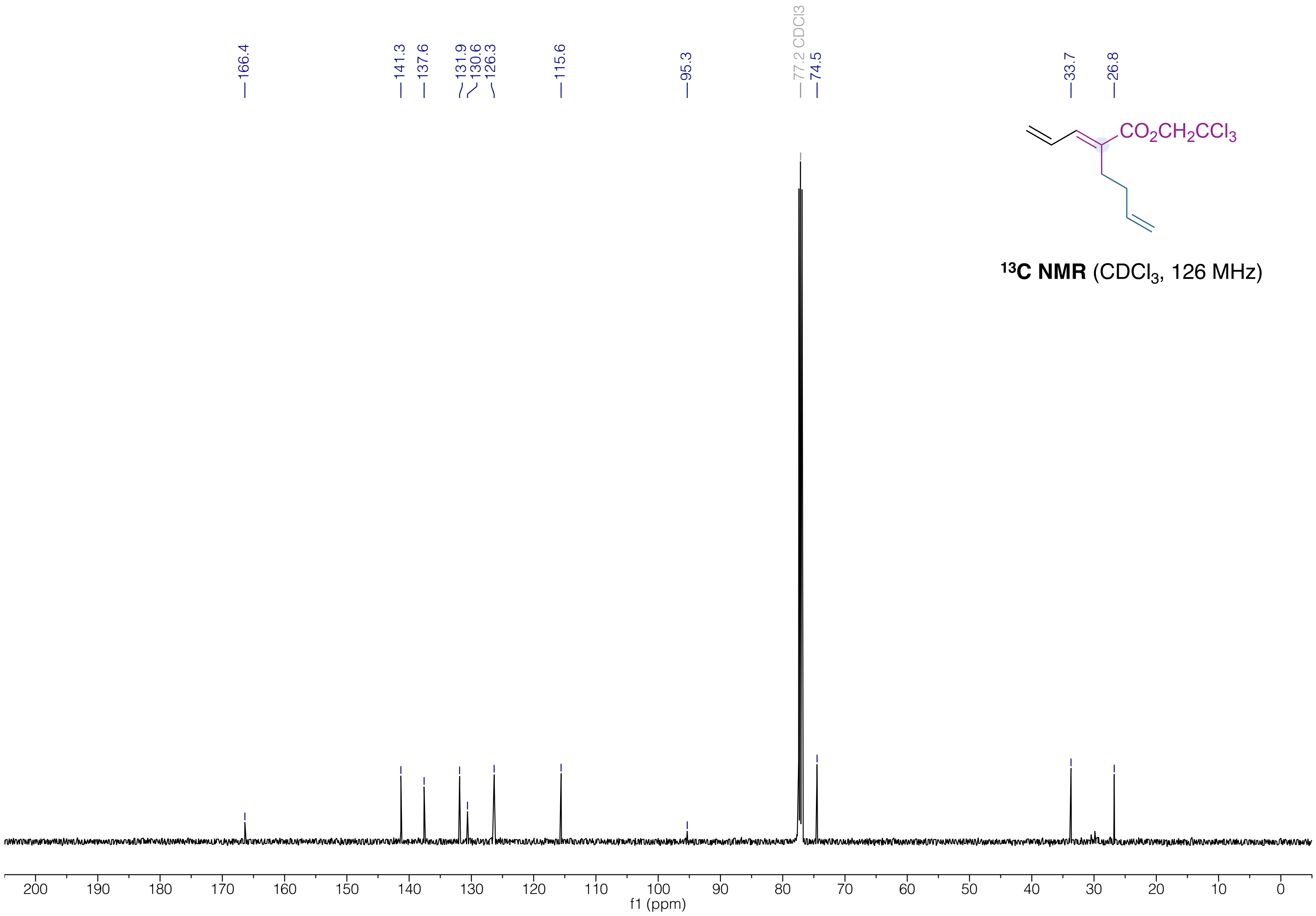

166.4
141.3
137.6
131.9
130.6
126.3
115.6
95.3
77.2 CDCl3
74.5
33.7
26.8
CO2CH2CCl3
13C NMR (CDCl3, 126 MHz)
200
190
180
170
160
150
140
130
120
110
100
90
80
70
60
50
40
30
20
10
0
f1 (ppm)

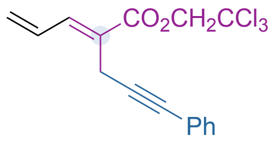

¹H NMR ($CDCl_3$, 500 MHz)

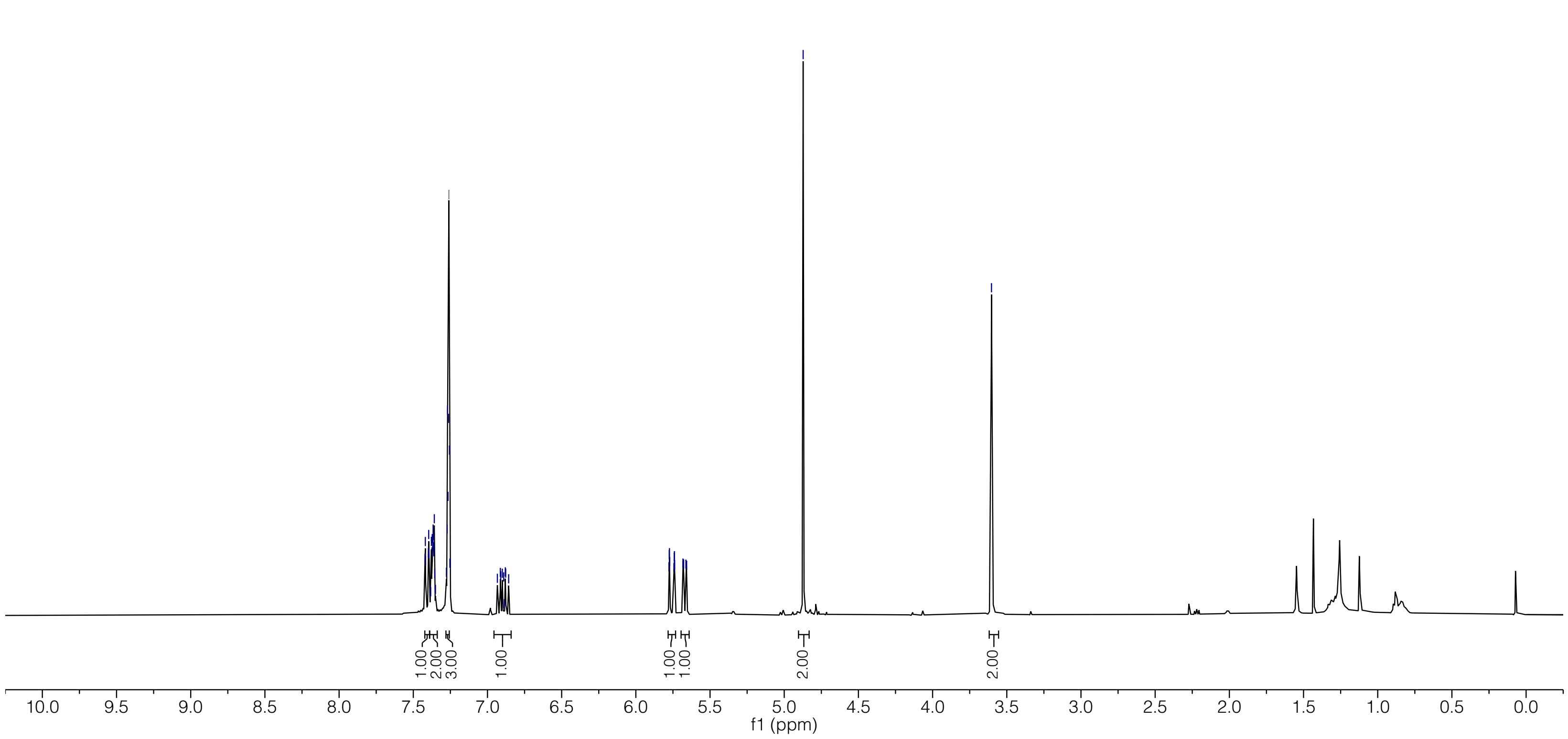

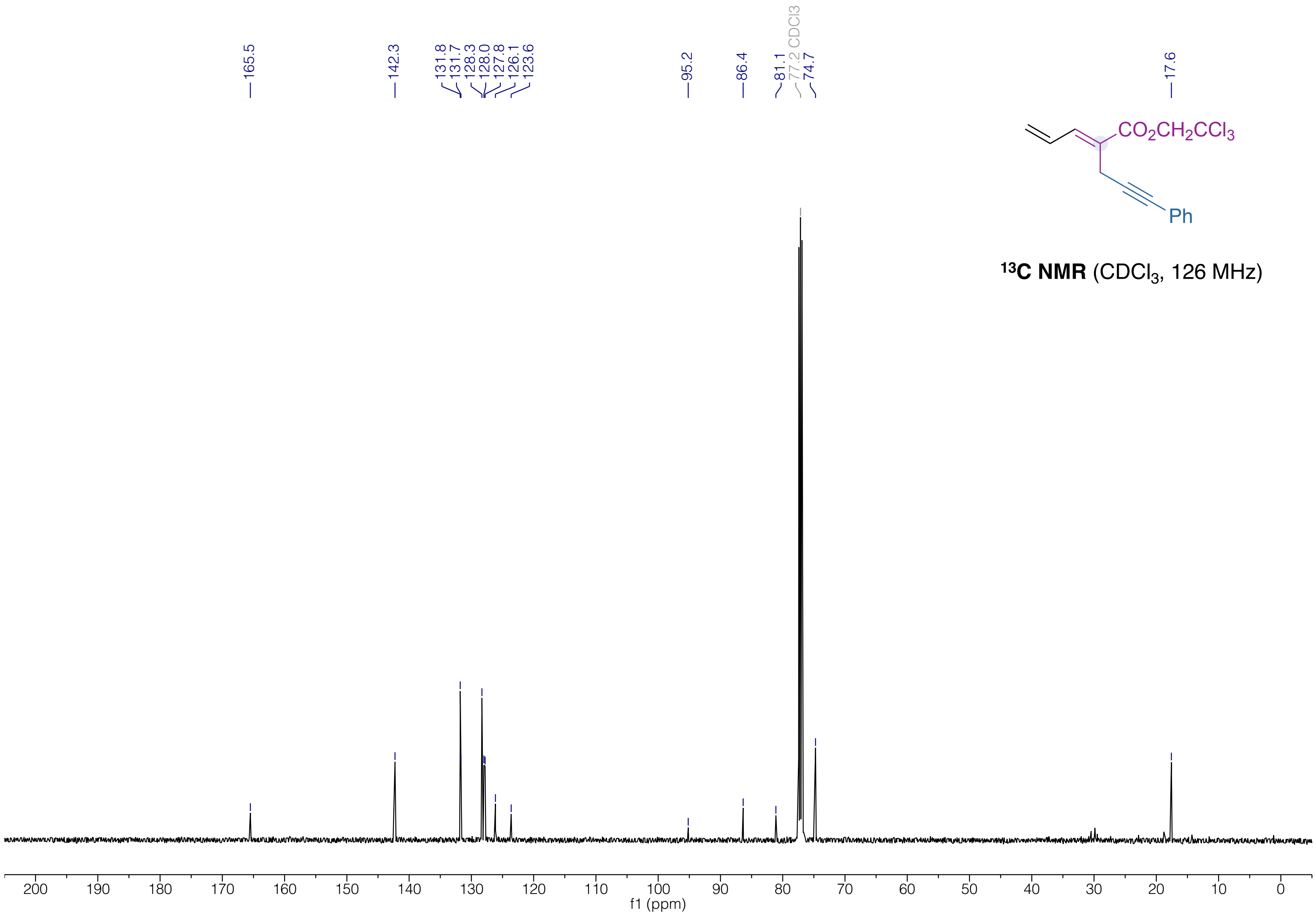

165.5
142.3
131.8
131.7
128.3
128.0
127.8
126.1
123.6
95.2
86.4
81.1
77.2 CDCl3
74.7
17.6
CO2CH2CCl3
Ph
13C NMR (CDCl3, 126 MHz)
200
190
180
170
160
150
140
130
120
110
100
90
80
70
60
50
40
30
20
10
0
f1 (ppm)

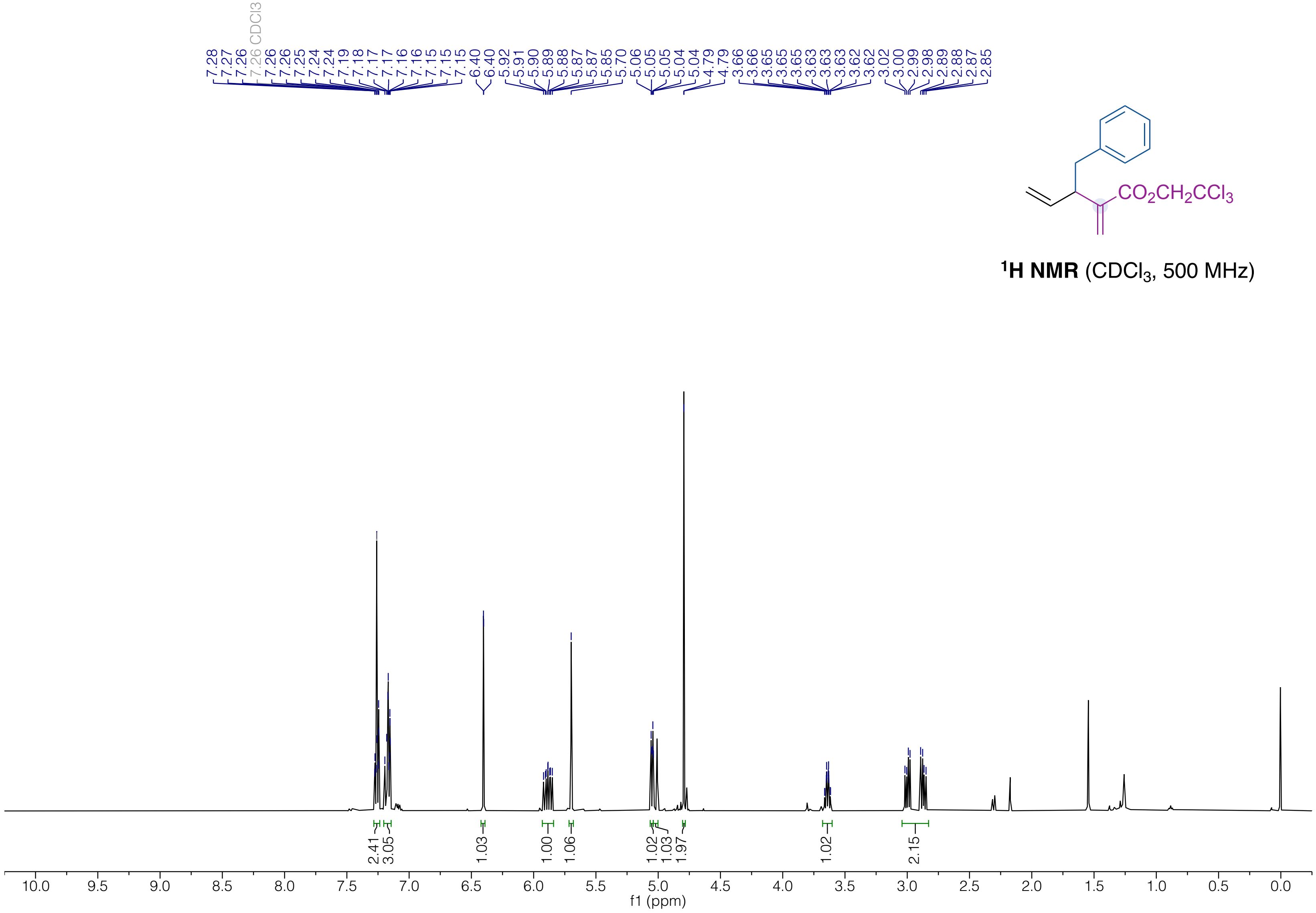

^{1}H NMR ($CDCl_3$, 500 MHz)

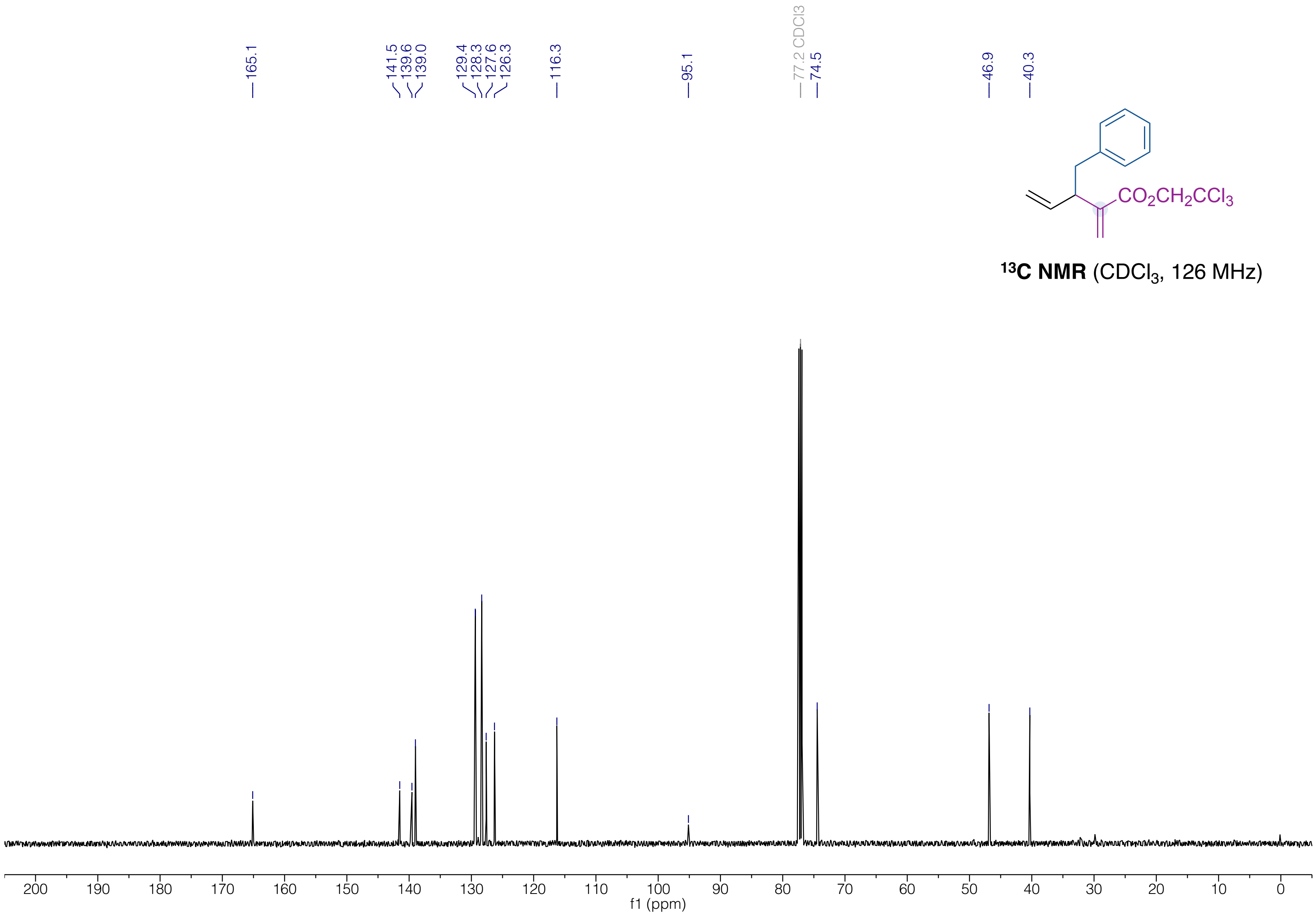

^{13}C NMR ($CDCl_3$, 126 MHz)

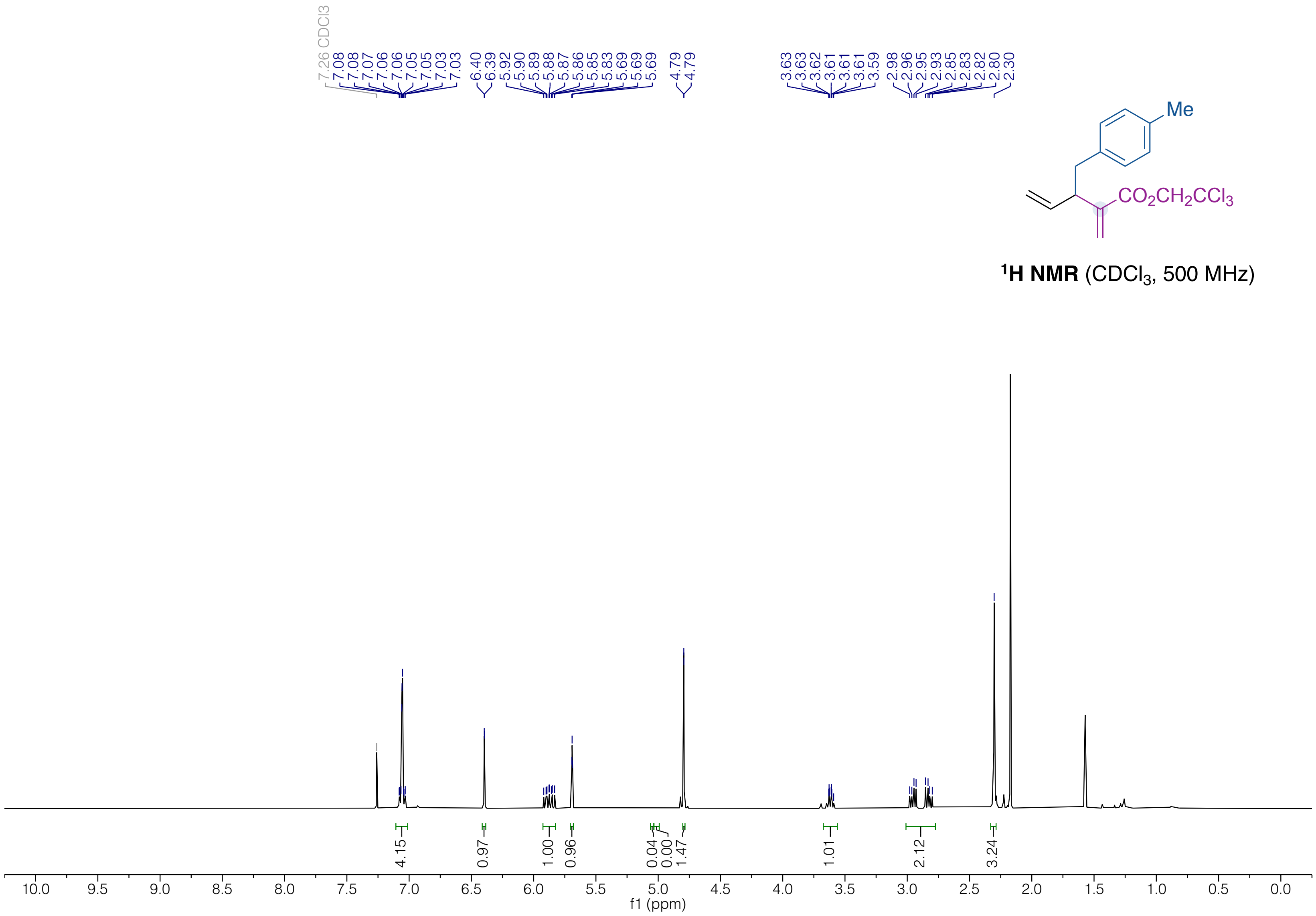

¹H NMR ($CDCl_3$, 500 MHz)

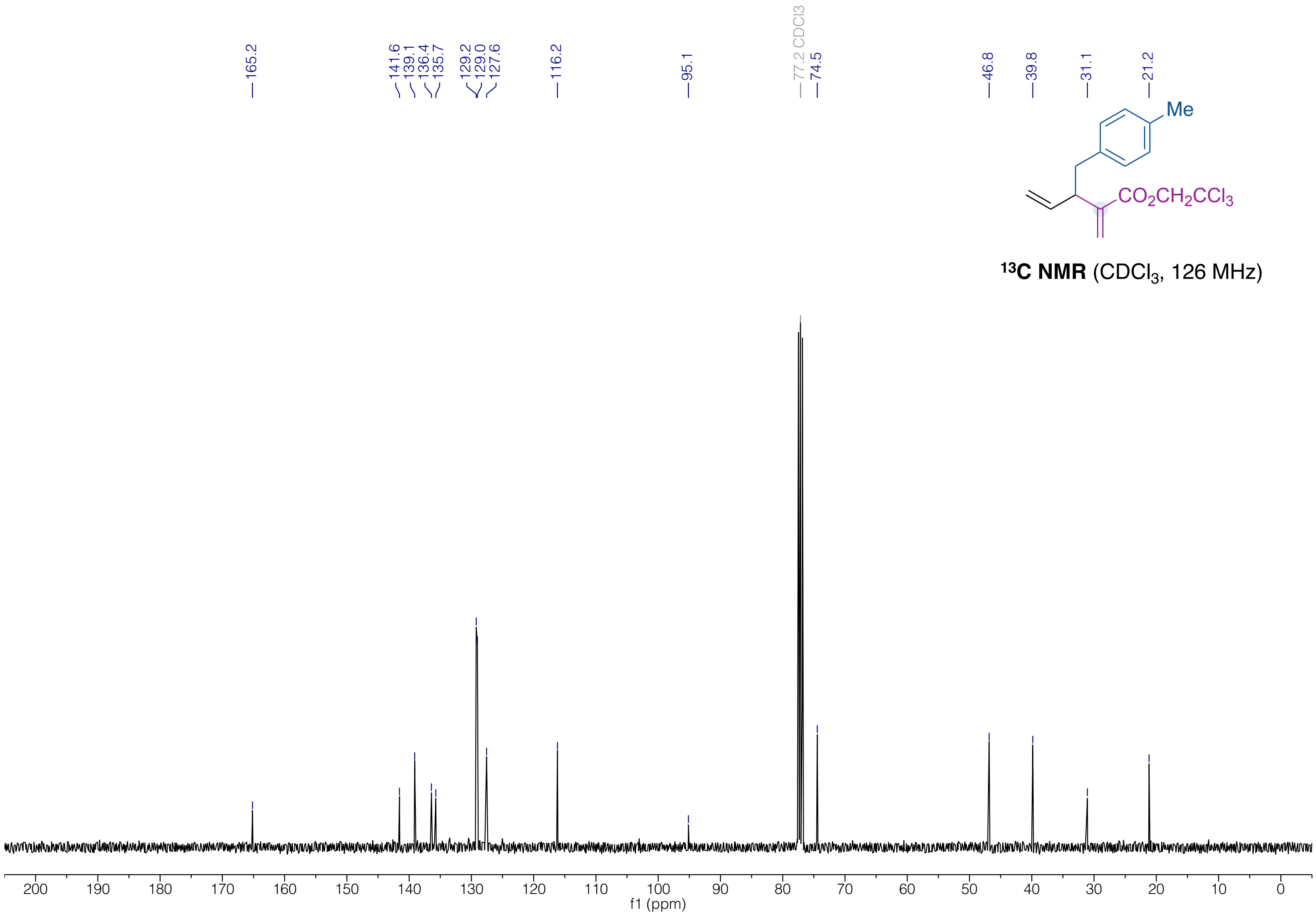

^{13}C NMR ($CDCl_3$, 126 MHz)

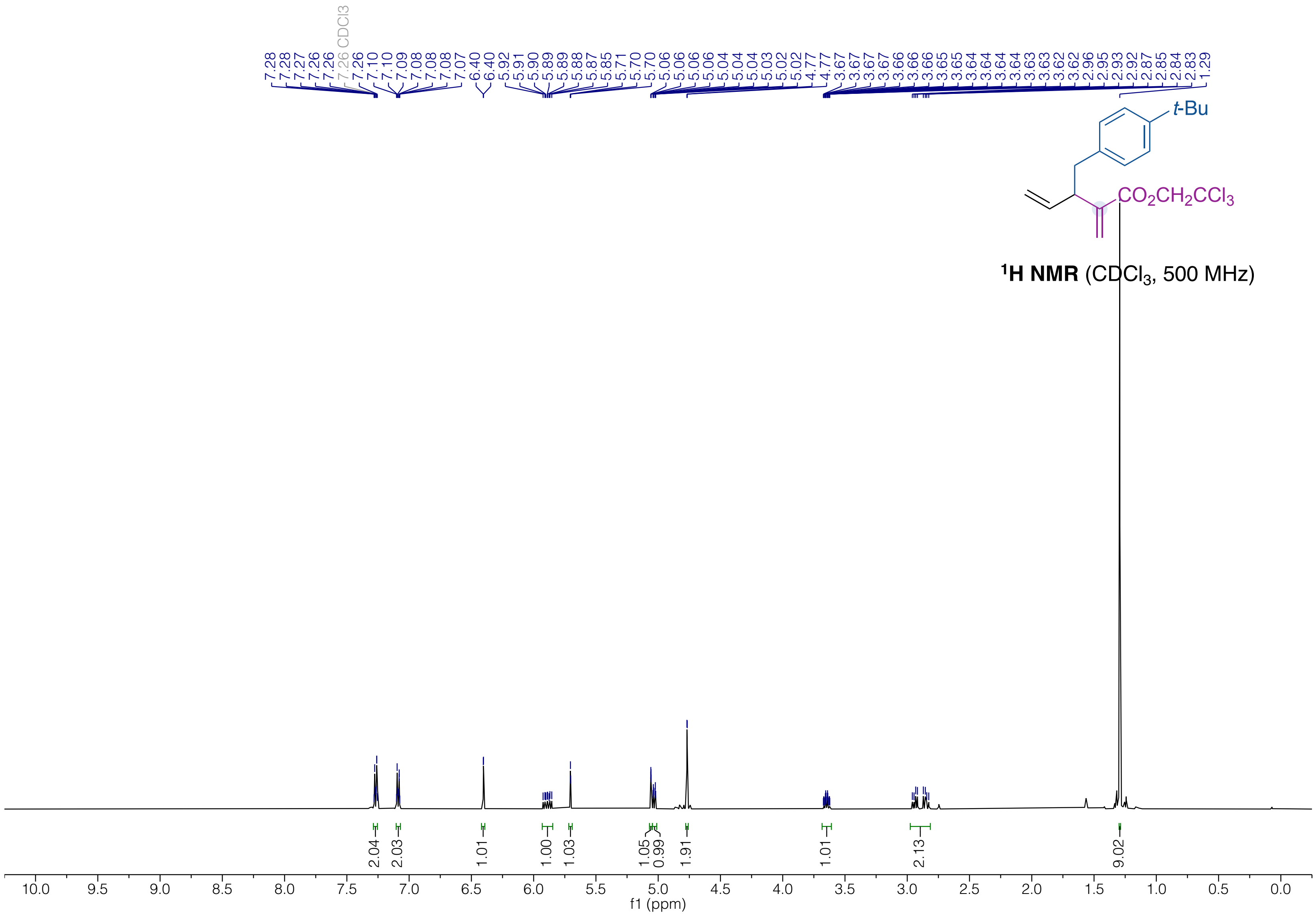
t-Bu
CO2CH2CCl3
1H NMR (CDCl3, 500 MHz)
7.28 7.28 7.27 7.26 7.26 7.26 CDCl3 7.26 7.10 7.10 7.09 7.08 7.08 7.07 6.40 6.40 5.92 5.91 5.90 5.89 5.89 5.88 5.87 5.85 5.71 5.70 5.70 5.06 5.06 5.06 5.06 5.04 5.04 5.03 5.02 5.02 4.77 4.77 3.67 3.67 3.67 3.66 3.66 3.66 3.65 3.65 3.64 3.64 3.64 3.64 3.63 3.63 3.62 3.62 2.96 2.95 2.93 2.92 2.87 2.85 2.84 2.83 1.29
2.04 2.03 1.01 1.00 1.03 1.05 0.99 1.91 1.01 2.13 9.02
10.0 9.5 9.0 8.5 8.0 7.5 7.0 6.5 6.0 5.5 5.0 4.5 4.0 3.5 3.0 2.5 2.0 1.5 1.0 0.5 0.0
f1 (ppm)

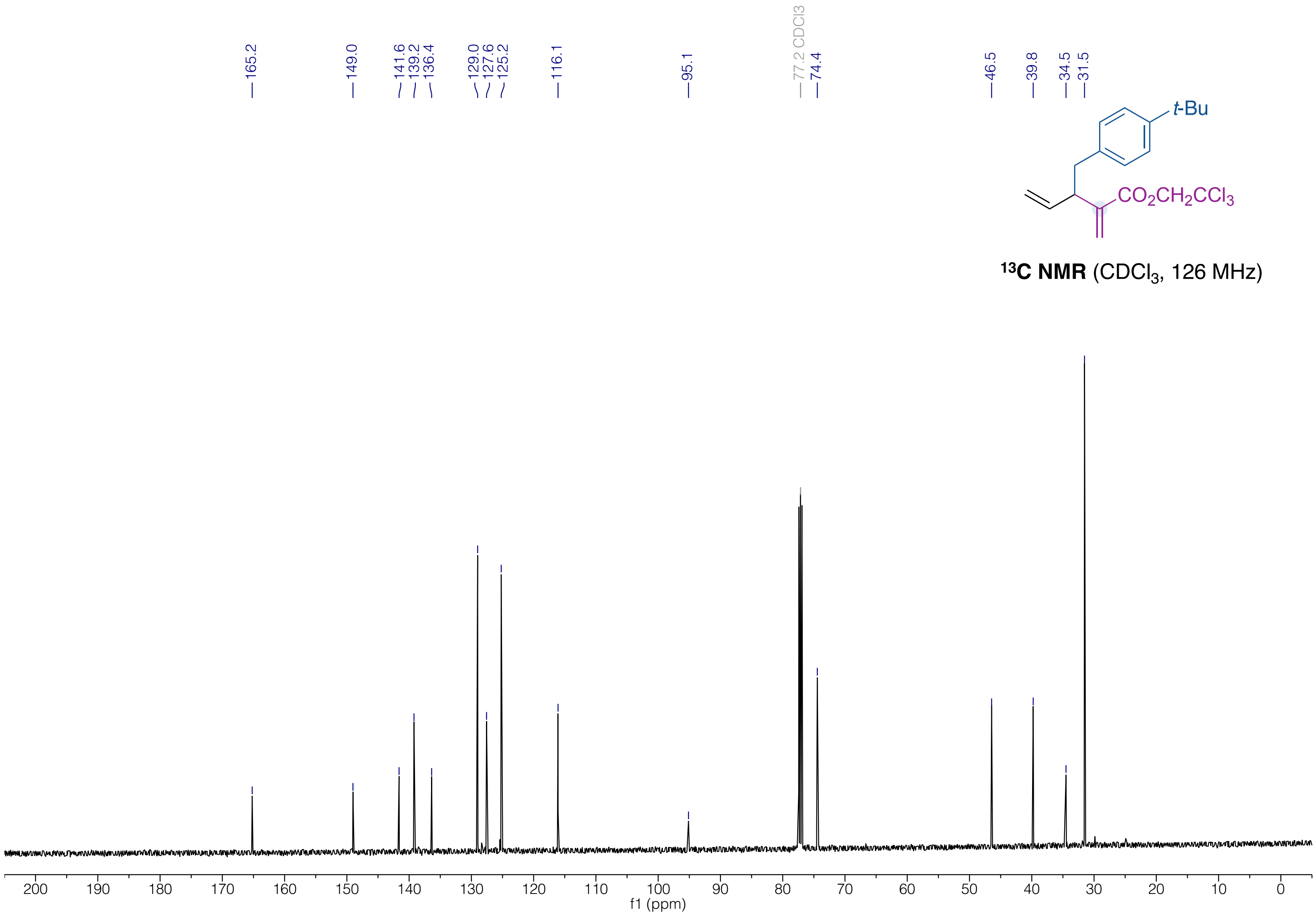
165.2
149.0
141.6
139.2
136.4
129.0
127.6
125.2
116.1
95.1
77.2 CDCl3
74.4
46.5
39.8
34.5
31.5
t-Bu
CO2CH2CCl3
13C NMR (CDCl3, 126 MHz)
f1 (ppm)

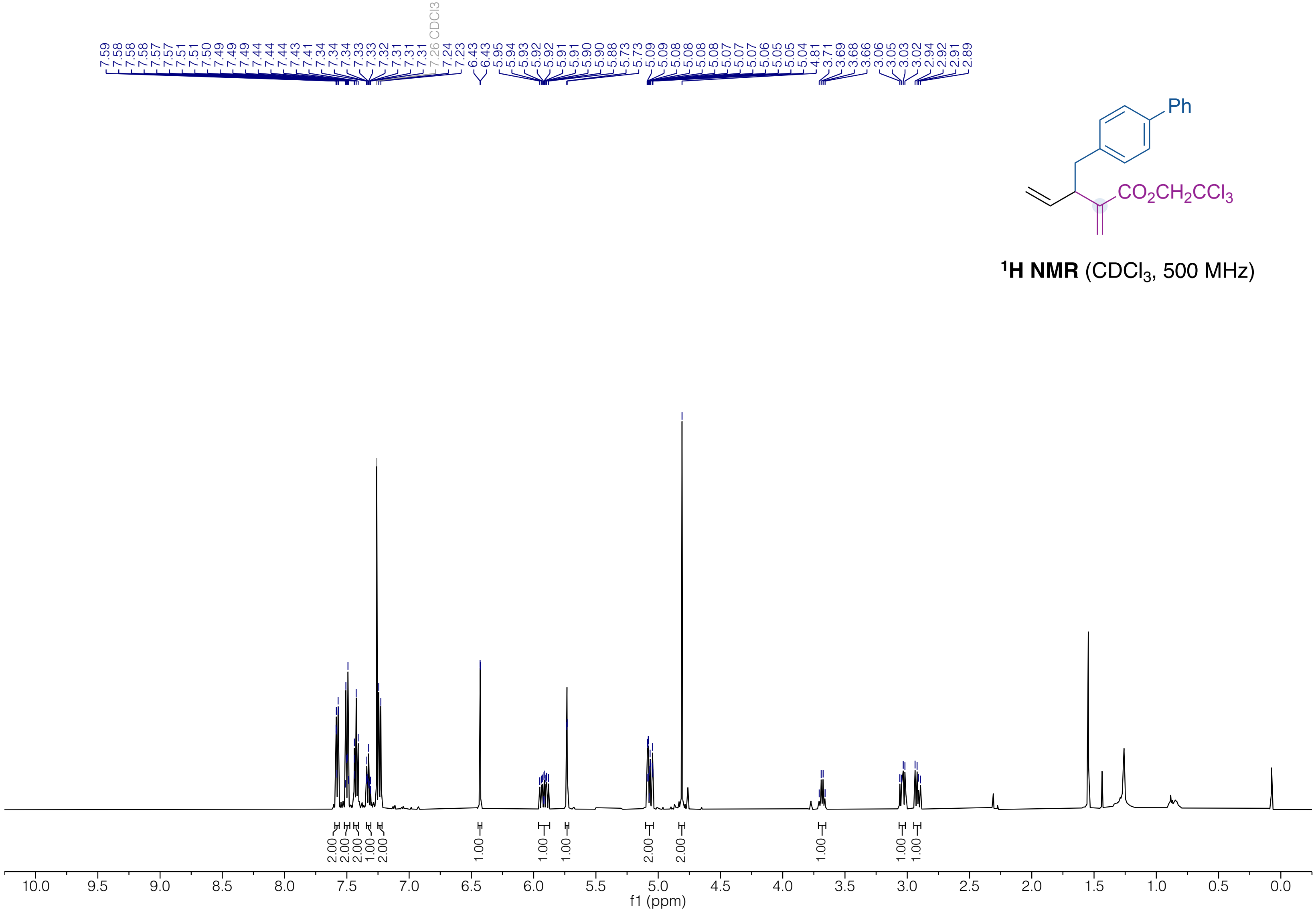

^{1}H NMR ($CDCl_3$, 500 MHz)

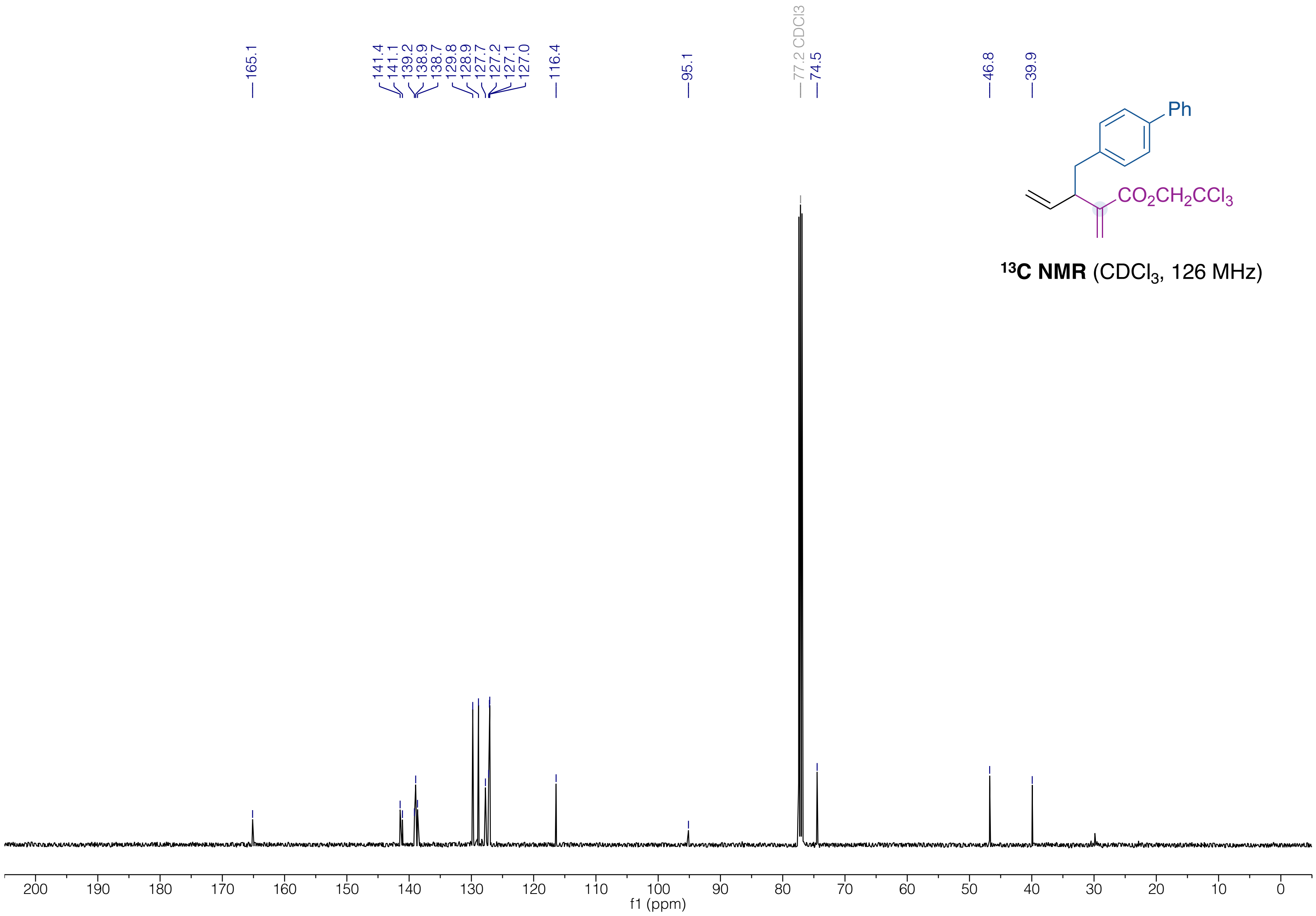

165.1
141.4
141.1
139.2
138.9
138.7
129.8
128.9
127.7
127.2
127.1
127.0
116.4
95.1
77.2 CDCl3
74.5
46.8
39.9
Ph
CO2CH2CCl3
13C NMR (CDCl3, 126 MHz)
200
190
180
170
160
150
140
130
120
110
100
90
80
70
60
50
40
30
20
10
0
f1 (ppm)

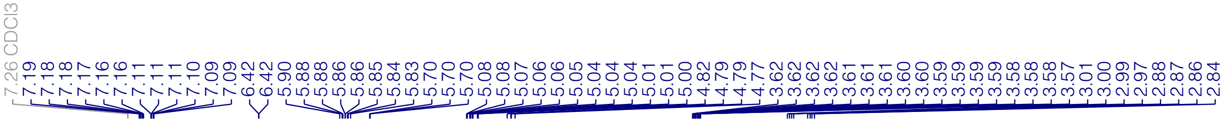

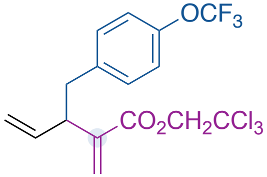

^{1}H NMR ($CDCl_3$, 500 MHz)

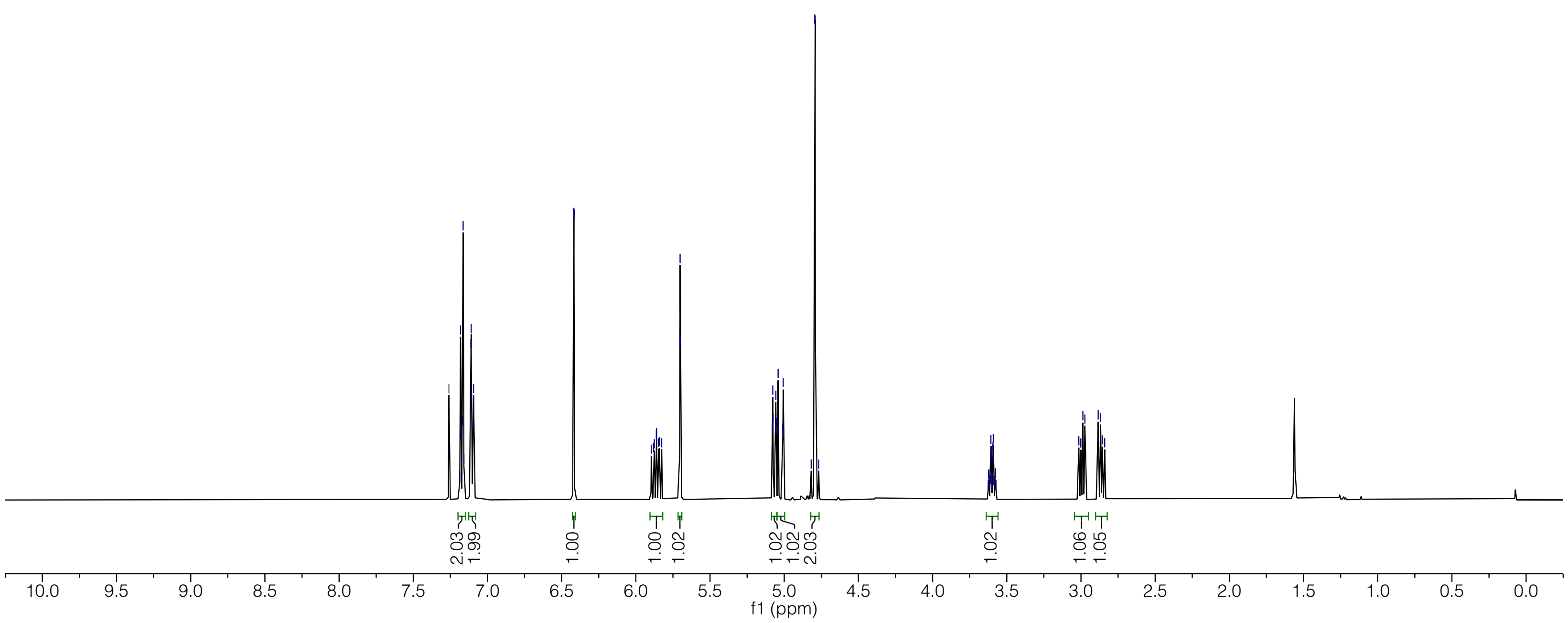

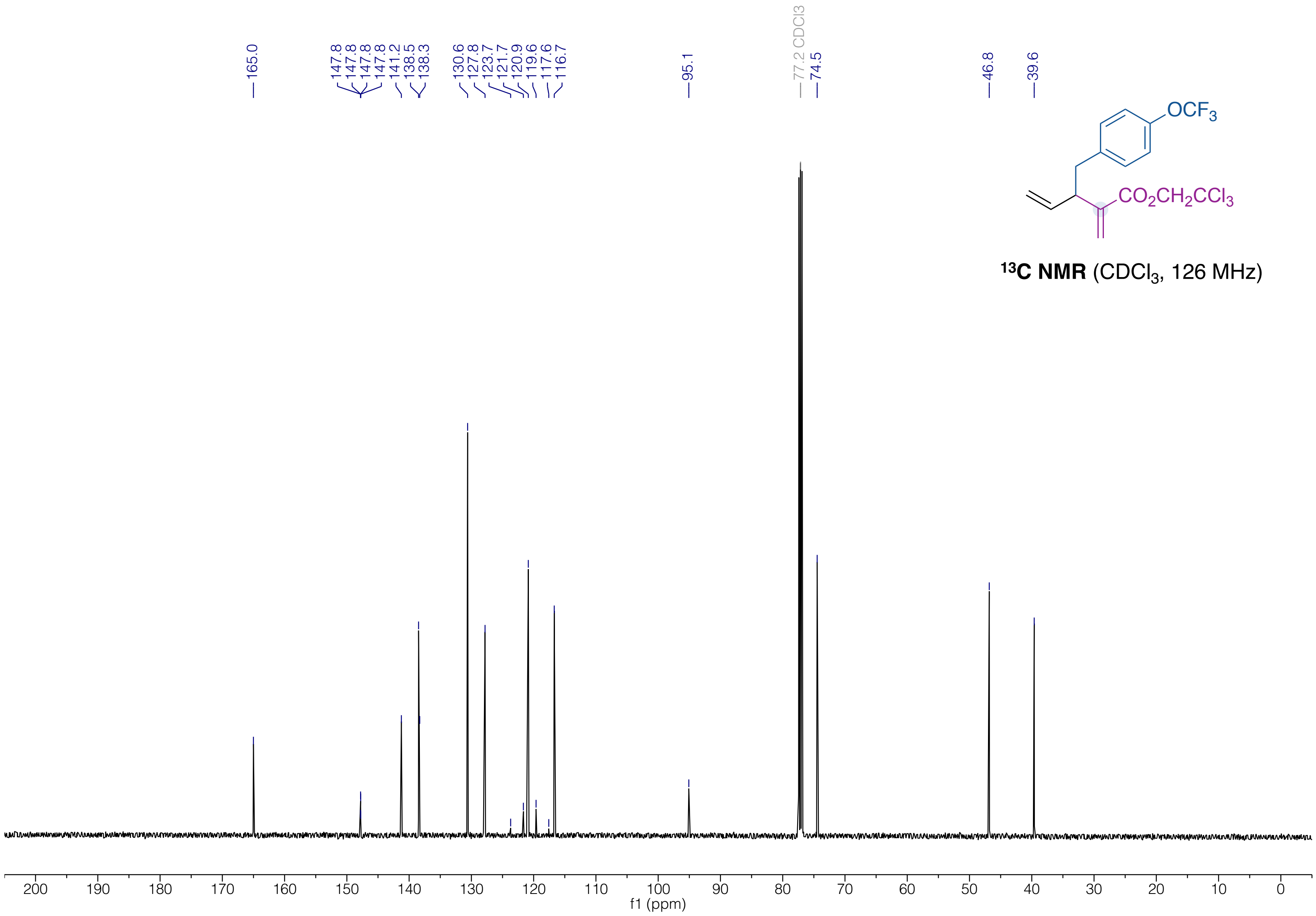
165.0
147.8
147.8
147.8
147.8
141.2
138.5
138.3
130.6
127.8
123.7
121.7
120.9
119.6
117.6
116.7
95.1
77.2 CDCl3
74.5
46.8
39.6
OCF3
CO2CH2CCl3
13C NMR (CDCl3, 126 MHz)
200
190
180
170
160
150
140
130
120
110
100
90
80
70
60
50
40
30
20
10
0
f1 (ppm)

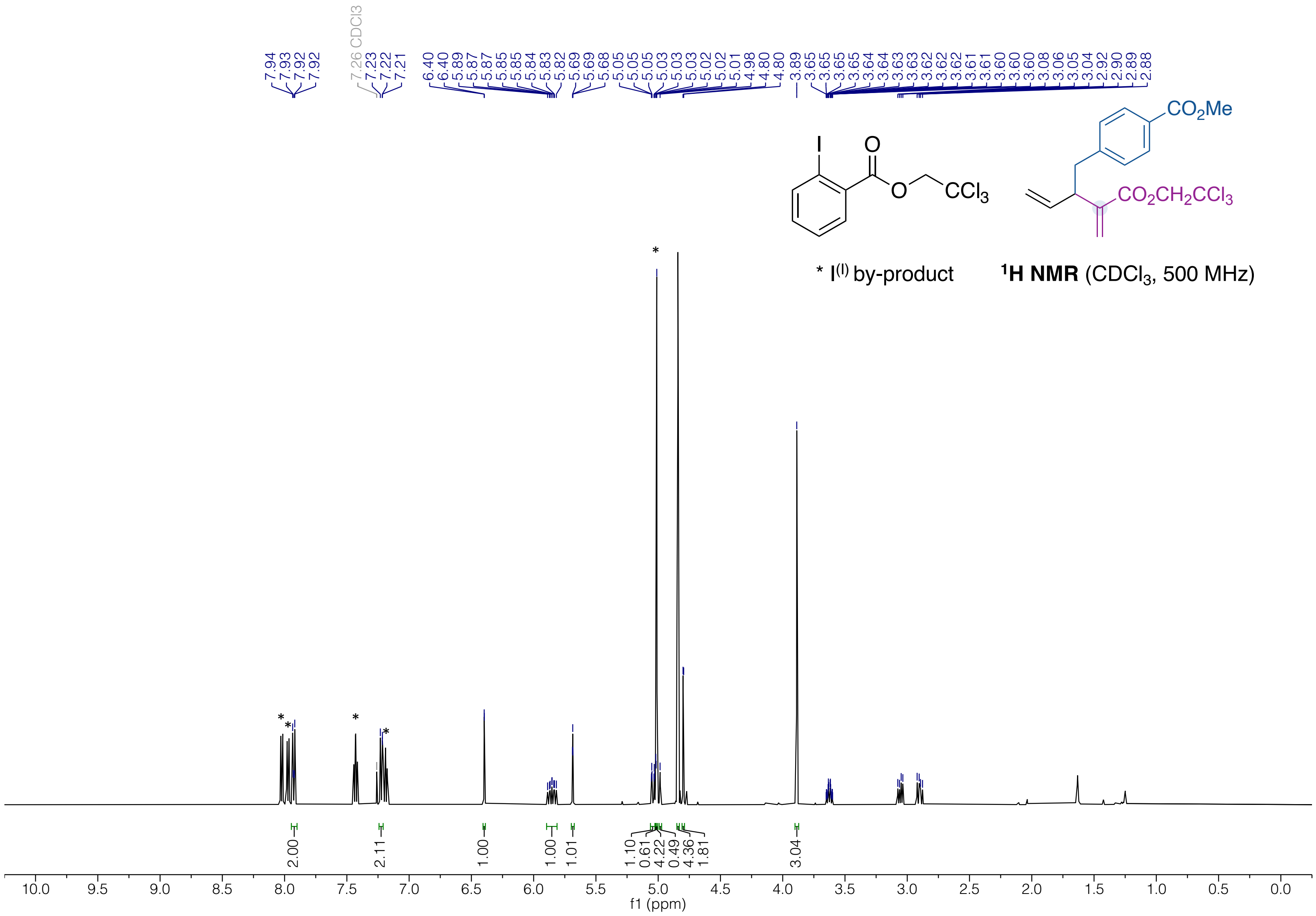

7.94
7.93
7.92
7.92
7.26 CDCl3
7.23
7.22
7.21
6.40
6.40
5.89
5.87
5.85
5.85
5.84
5.83
5.82
5.69
5.69
5.68
5.05
5.05
5.03
5.03
5.03
5.02
5.01
4.98
4.80
3.89
3.65
3.65
3.65
3.64
3.64
3.63
3.63
3.62
3.62
3.61
3.61
3.60
3.60
3.60
3.08
3.06
3.05
3.04
2.92
2.90
2.89
2.88
I
O
O
CCl3
CO2Me
CO2CH2CCl3
* I(I) by-product
1H NMR (CDCl3, 500 MHz)
2.00
2.11
1.00
1.00
1.01
1.10
0.61
4.22
0.49
4.36
1.81
3.04
10.0
9.5
9.0
8.5
8.0
7.5
7.0
6.5
6.0
5.5
5.0
4.5
4.0
3.5
3.0
2.5
2.0
1.5
1.0
0.5
0.0
f1 (ppm)

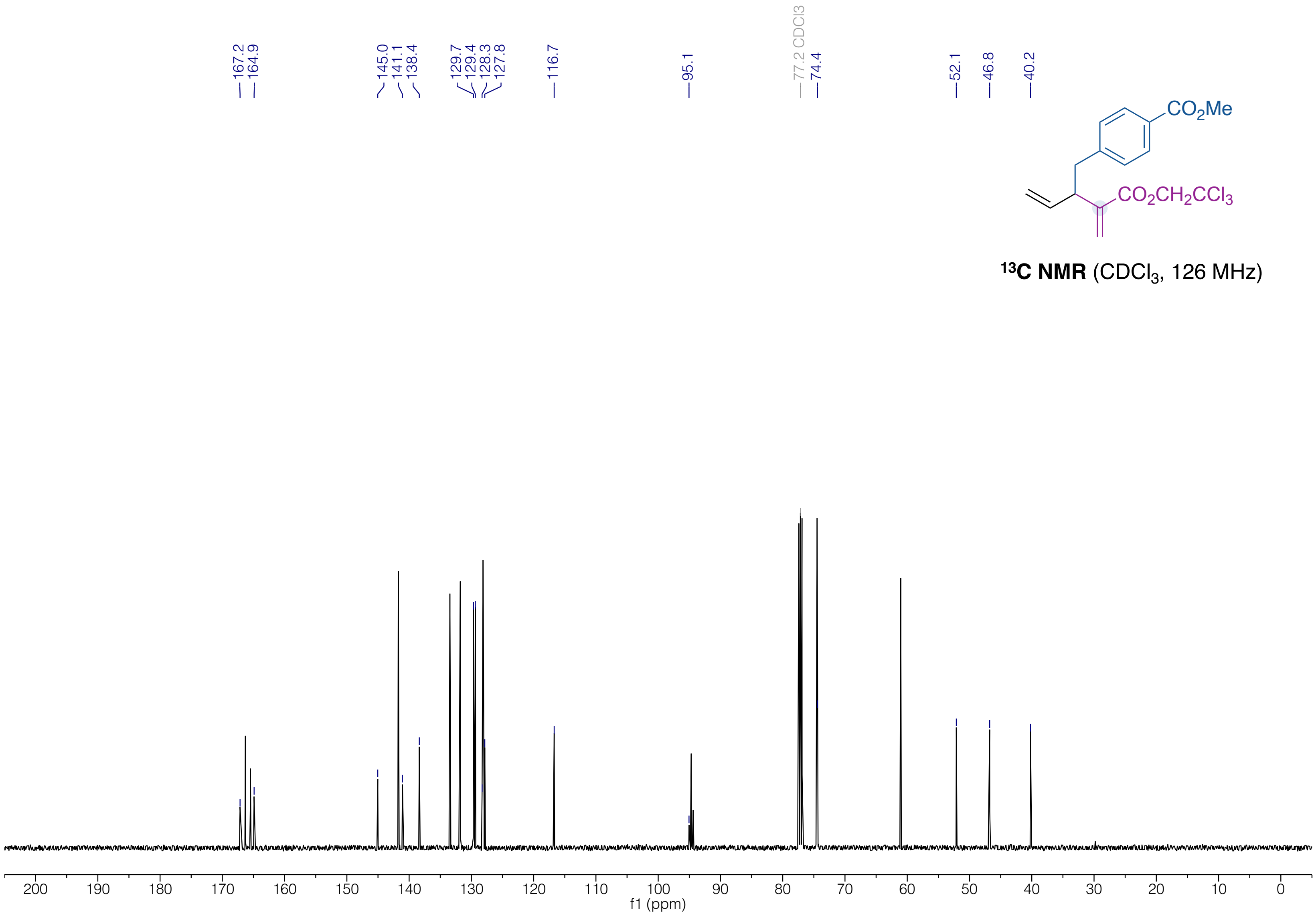

167.2
164.9
145.0
141.1
138.4
129.7
129.4
128.3
127.8
116.7
95.1
77.2 CDCl3
74.4
52.1
46.8
40.2
CO2Me
CO2CH2CCl3
13C NMR (CDCl3, 126 MHz)
200
190
180
170
160
150
140
130
120
110
100
90
80
70
60
50
40
30
20
10
0
f1 (ppm)

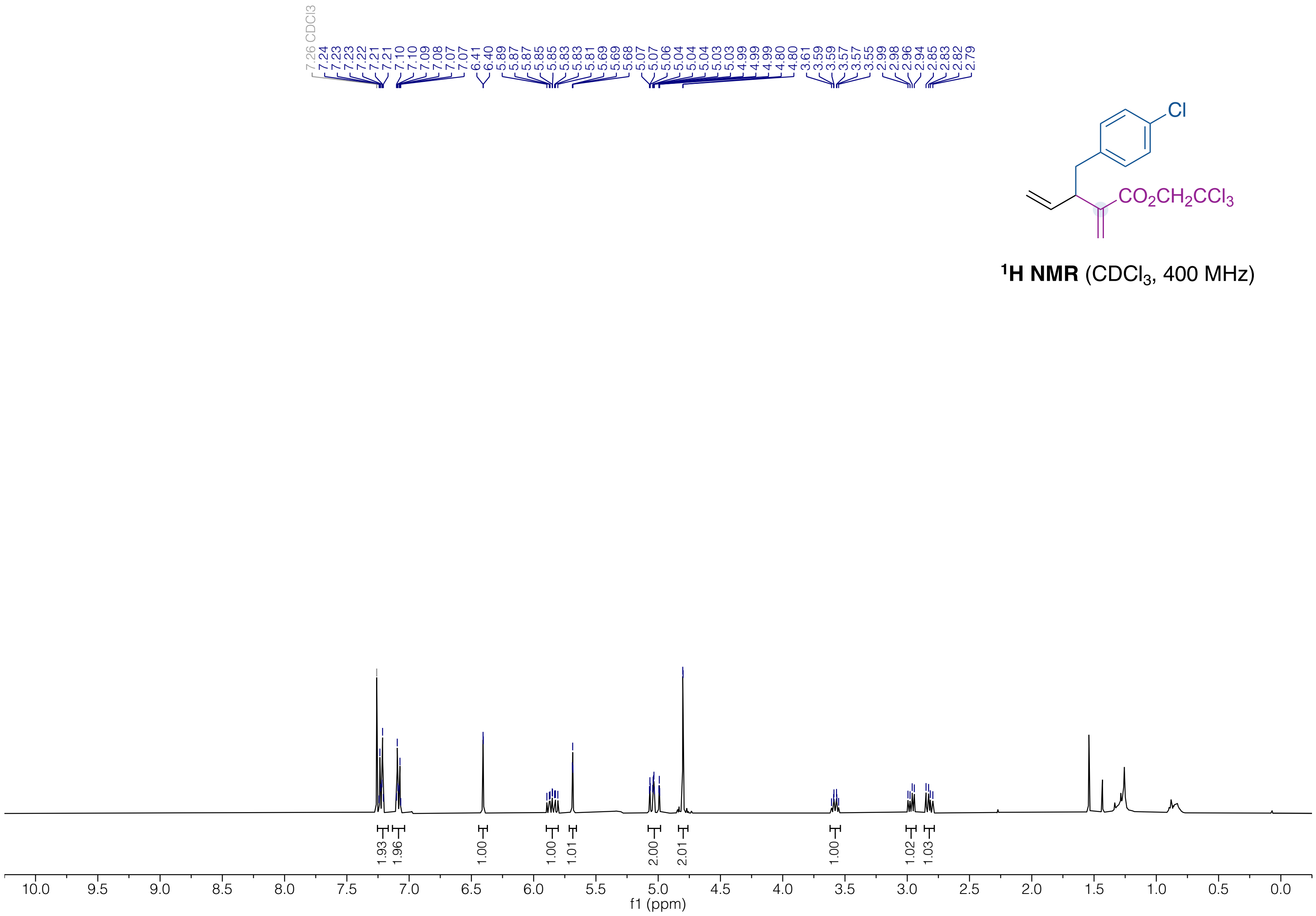

^{1}H NMR ($CDCl_3$, 400 MHz)

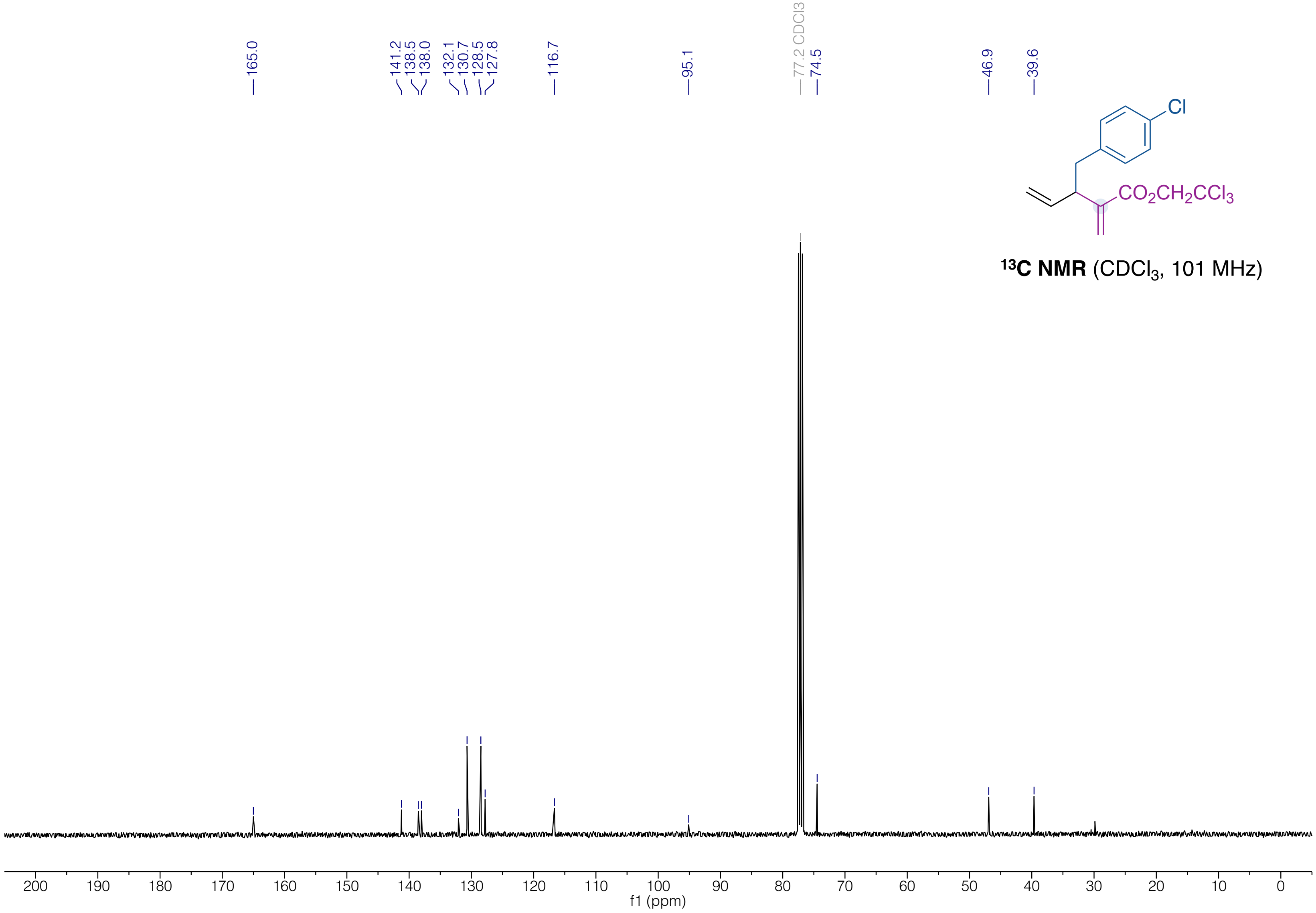

^{13}C NMR ($CDCl_3$, 101 MHz)

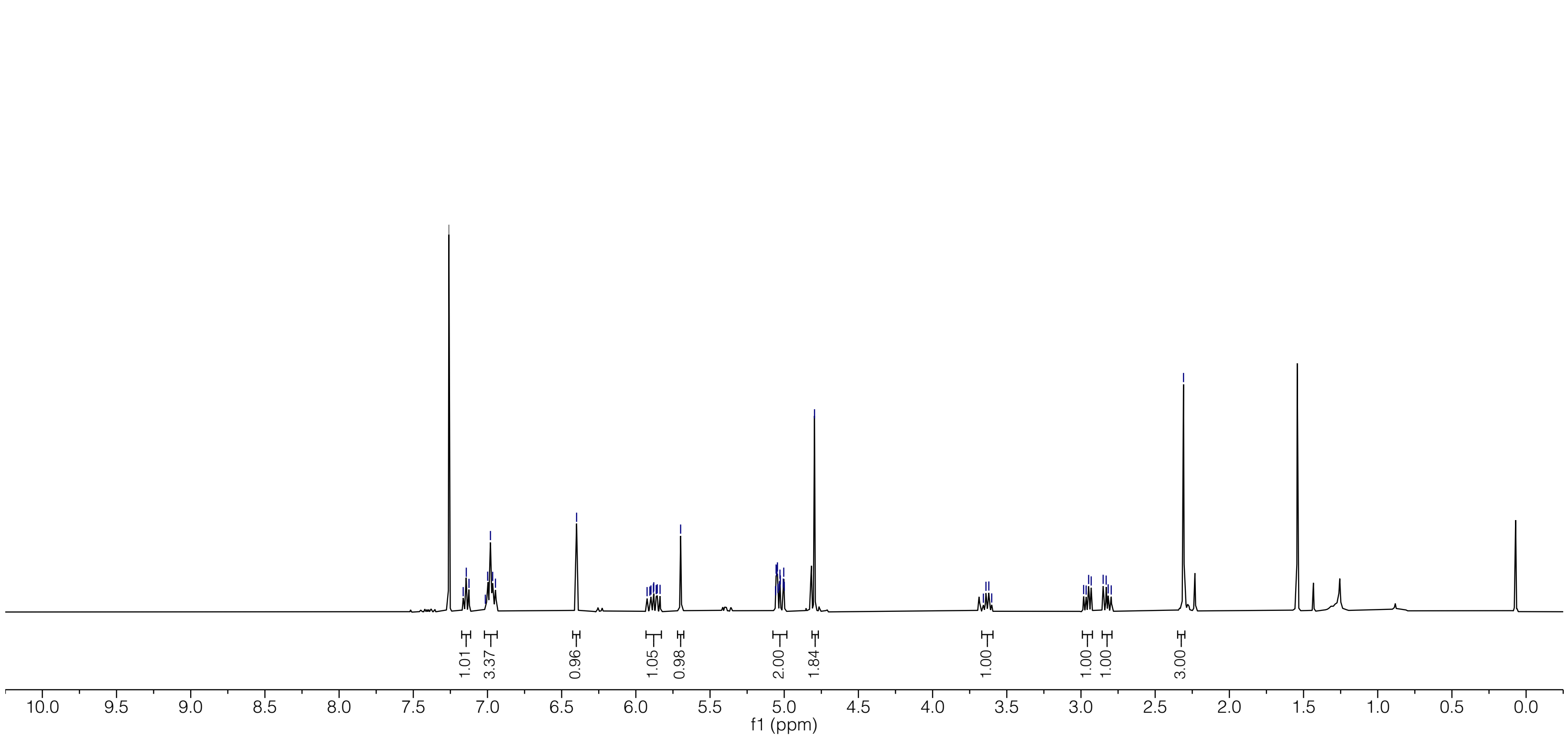

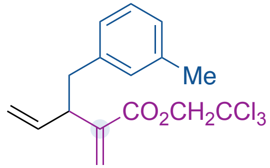

^{1}H NMR ($CDCl_3$, 400 MHz)

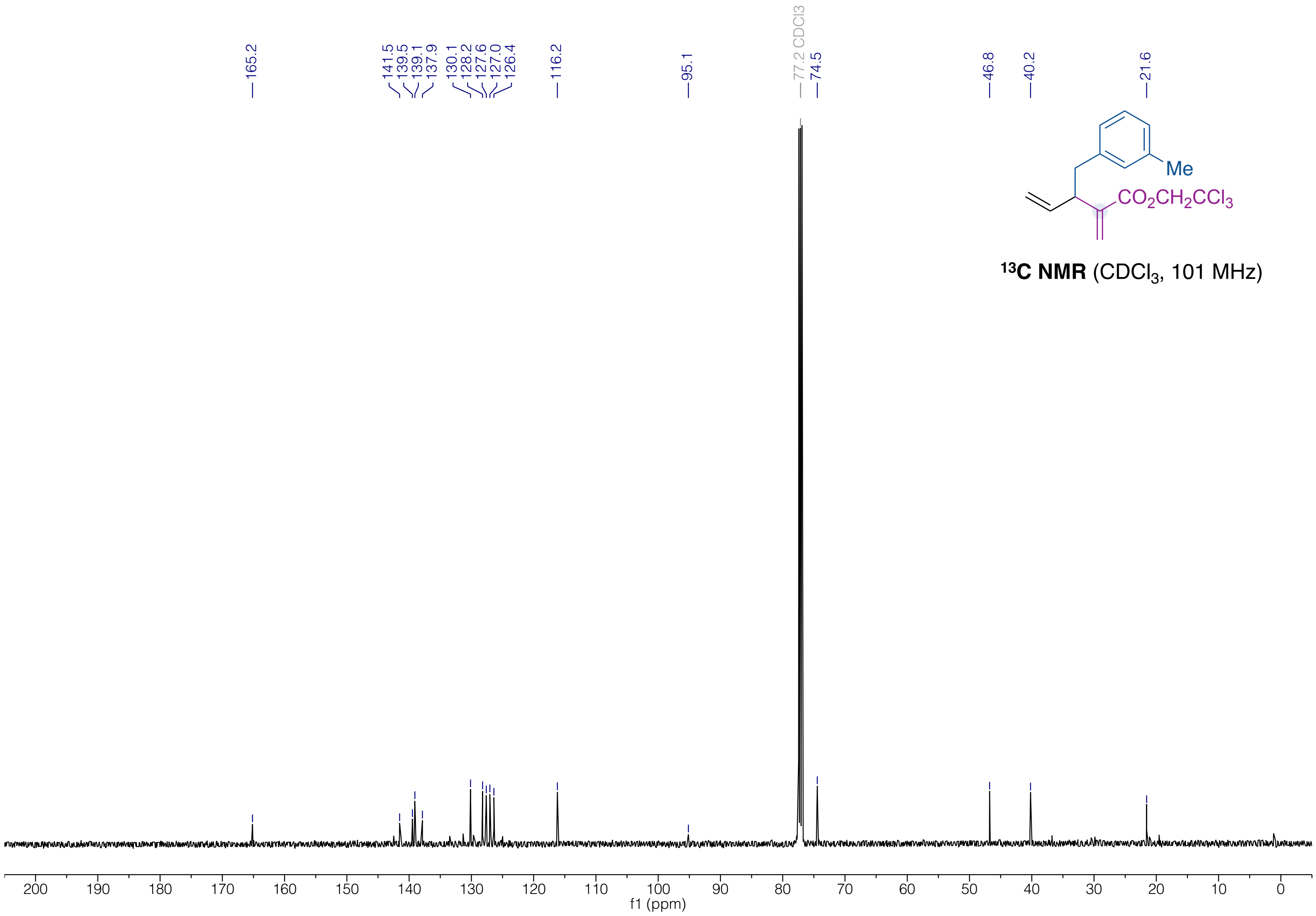

165.2
141.5
139.5
139.1
137.9
130.1
128.2
127.6
127.0
126.4
116.2
95.1
77.2 CDCl3
74.5
46.8
40.2
21.6
Me
CO2CH2CCl3
13C NMR (CDCl3, 101 MHz)
200
190
180
170
160
150
140
130
120
110
100
90
80
70
60
50
40
30
20
10
0
f1 (ppm)

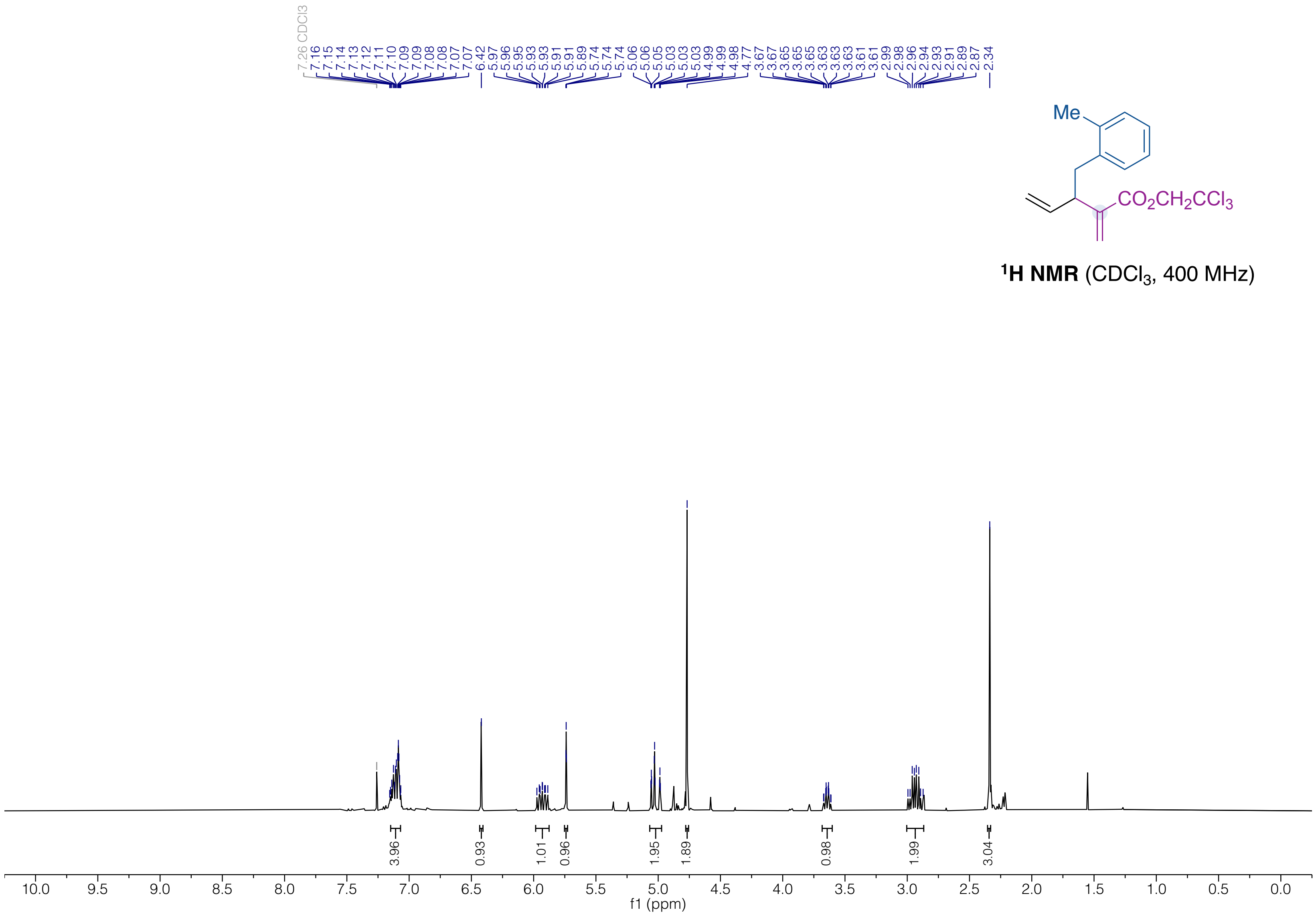

1H NMR ($CDCl_3$, 400 MHz)

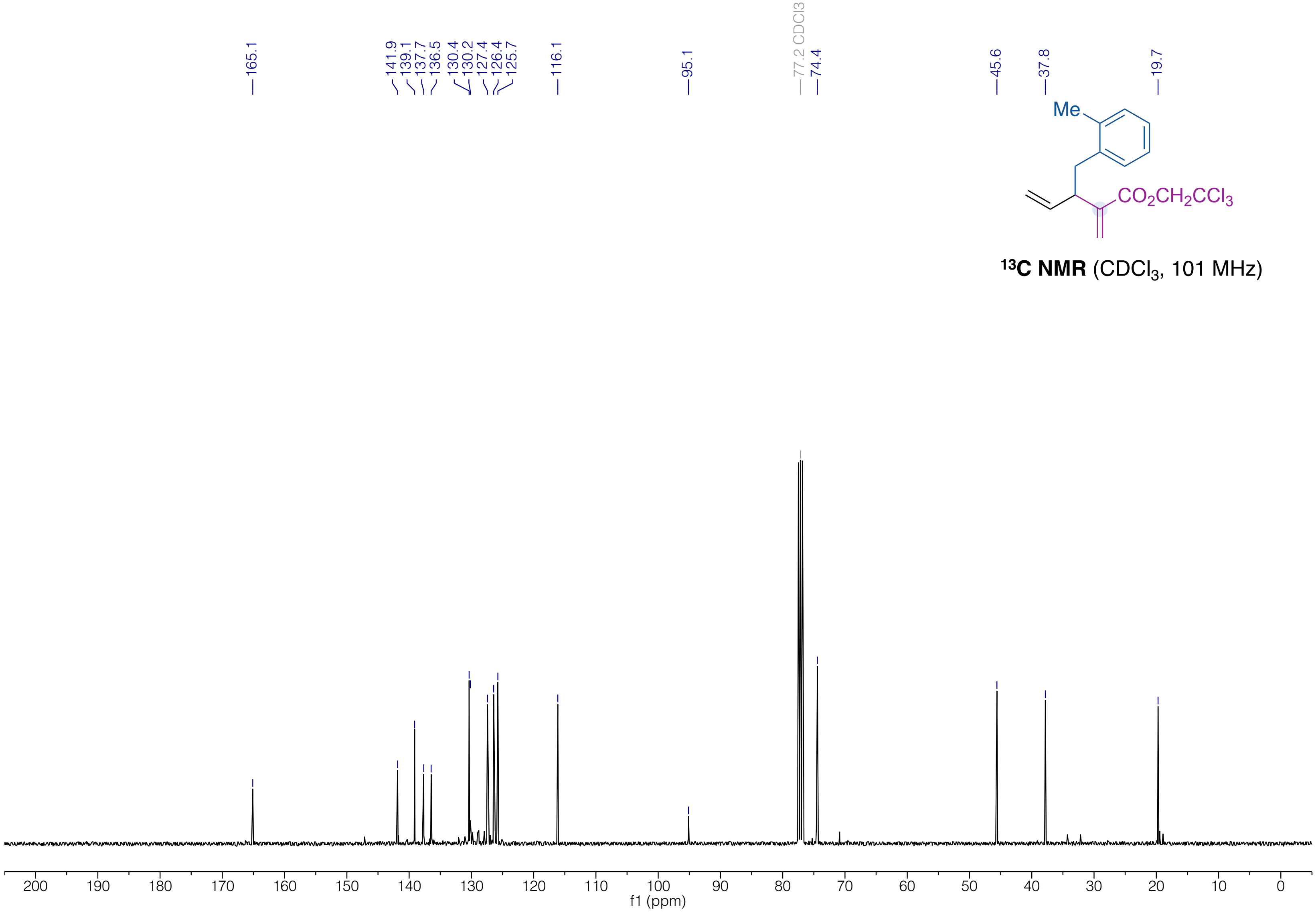
165.1
141.9
139.1
137.7
136.5
130.4
130.2
127.4
126.4
125.7
116.1
95.1
77.2 CDCl3
74.4
45.6
37.8
19.7
Me
$CO_2CH_2CCl_3$
^{13}C NMR ($CDCl_3$, 101 MHz)
f1 (ppm)

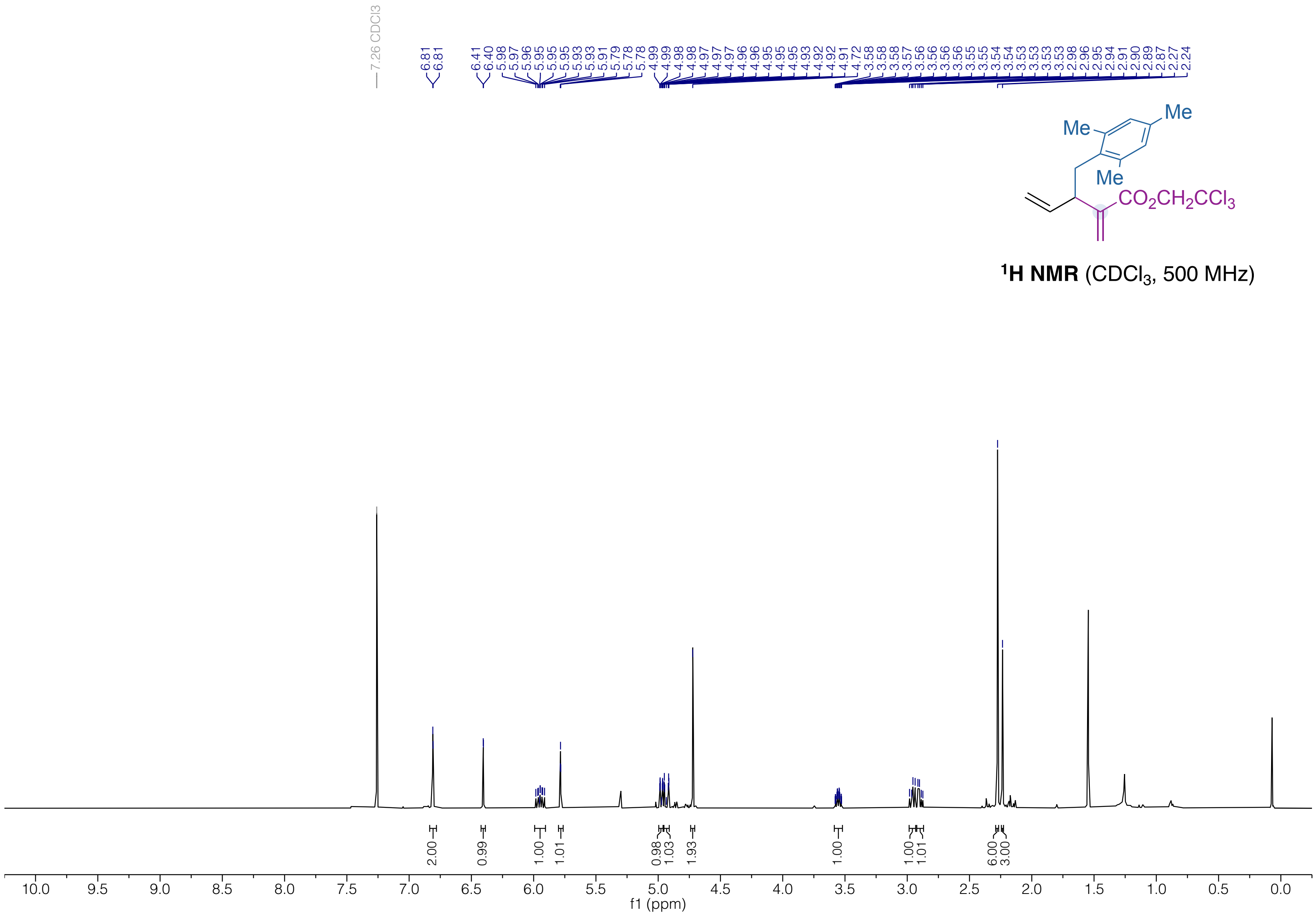

^{1}H NMR ($CDCl_3$, 500 MHz)

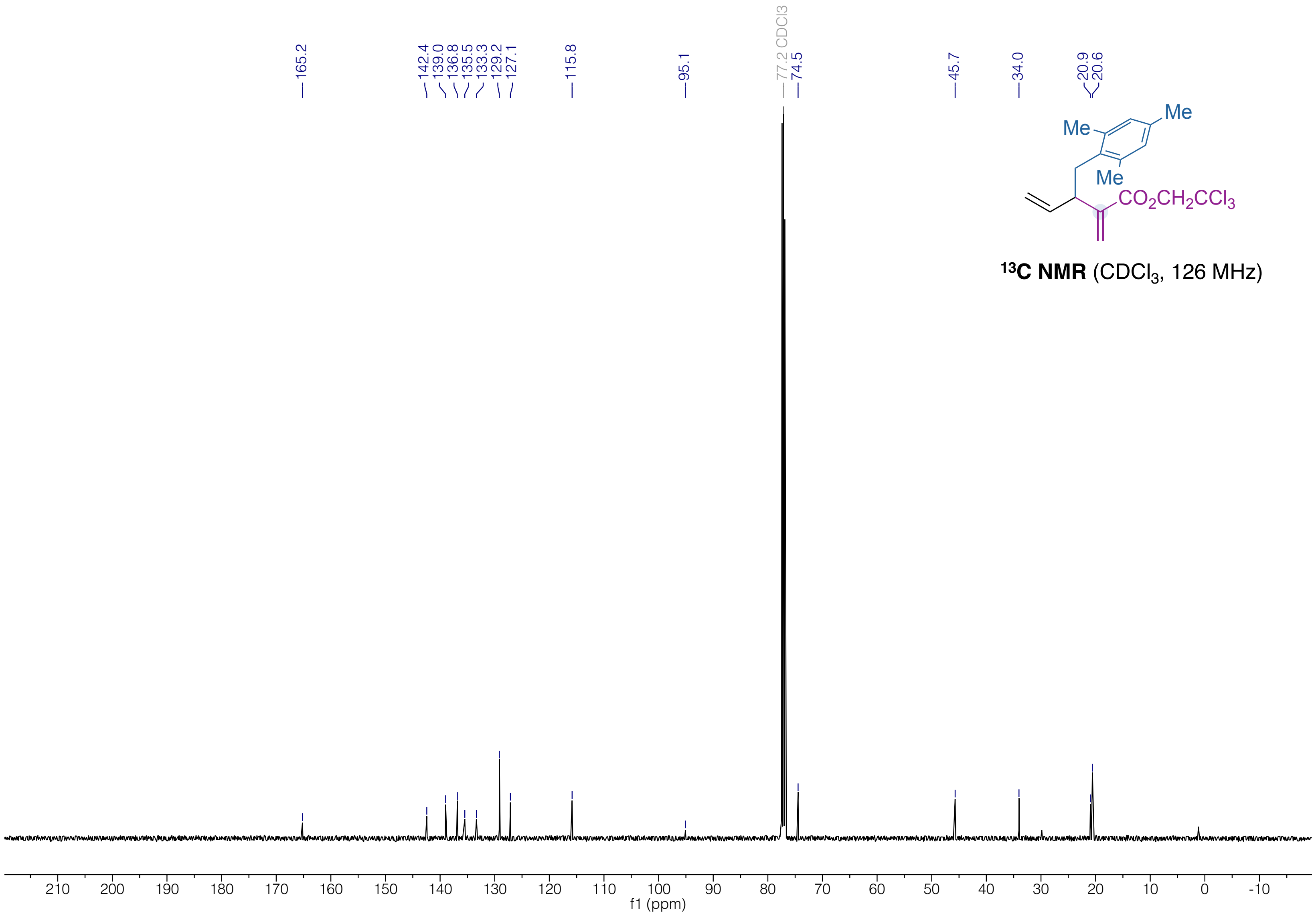

165.2
142.4
139.0
136.8
135.5
133.3
129.2
127.1
115.8
95.1
77.2 CDCl3
74.5
45.7
34.0
20.9
20.6
Me
Me
Me
CO2CH2CCl3
13C NMR (CDCl3, 126 MHz)
210 200 190 180 170 160 150 140 130 120 110 100 90 80 70 60 50 40 30 20 10 0 -10
f1 (ppm)

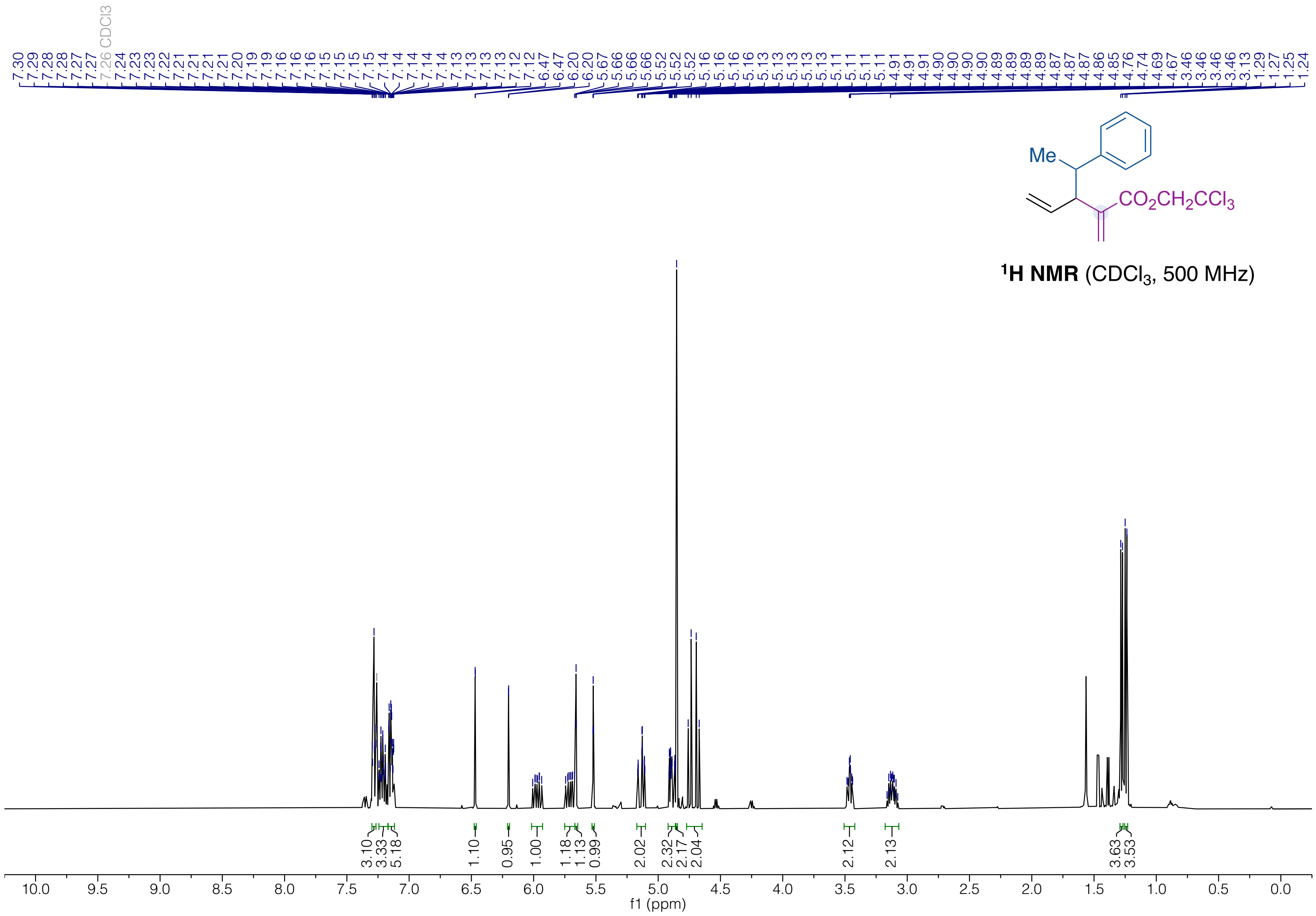

Me
$CO_2CH_2CCl_3$
^{1}H NMR ($CDCl_3$, 500 MHz)
7.30 7.29 7.28 7.28 7.27 7.27 7.26 CDCl3 7.24 7.23 7.23 7.22 7.21 7.21 7.21 7.20 7.19 7.19 7.16 7.16 7.15 7.15 7.15 7.14 7.14 7.14 7.14 7.13 7.13 7.13 7.13 7.12 6.47 6.47 6.20 6.20 5.67 5.66 5.66 5.52 5.52 5.52 5.16 5.16 5.16 5.13 5.13 5.13 5.13 5.11 5.11 5.11 4.91 4.91 4.91 4.90 4.90 4.90 4.89 4.89 4.89 4.87 4.87 4.86 4.85 4.76 4.74 4.69 4.67 3.46 3.46 3.46 3.13 1.29 1.27 1.25 1.24
3.10 3.33 5.18 1.10 0.95 1.00 1.18 1.13 0.99 2.02 2.32 2.17 2.04 2.12 2.13 3.63 3.53
10.0 9.5 9.0 8.5 8.0 7.5 7.0 6.5 6.0 5.5 5.0 4.5 4.0 3.5 3.0 2.5 2.0 1.5 1.0 0.5 0.0
f1 (ppm)

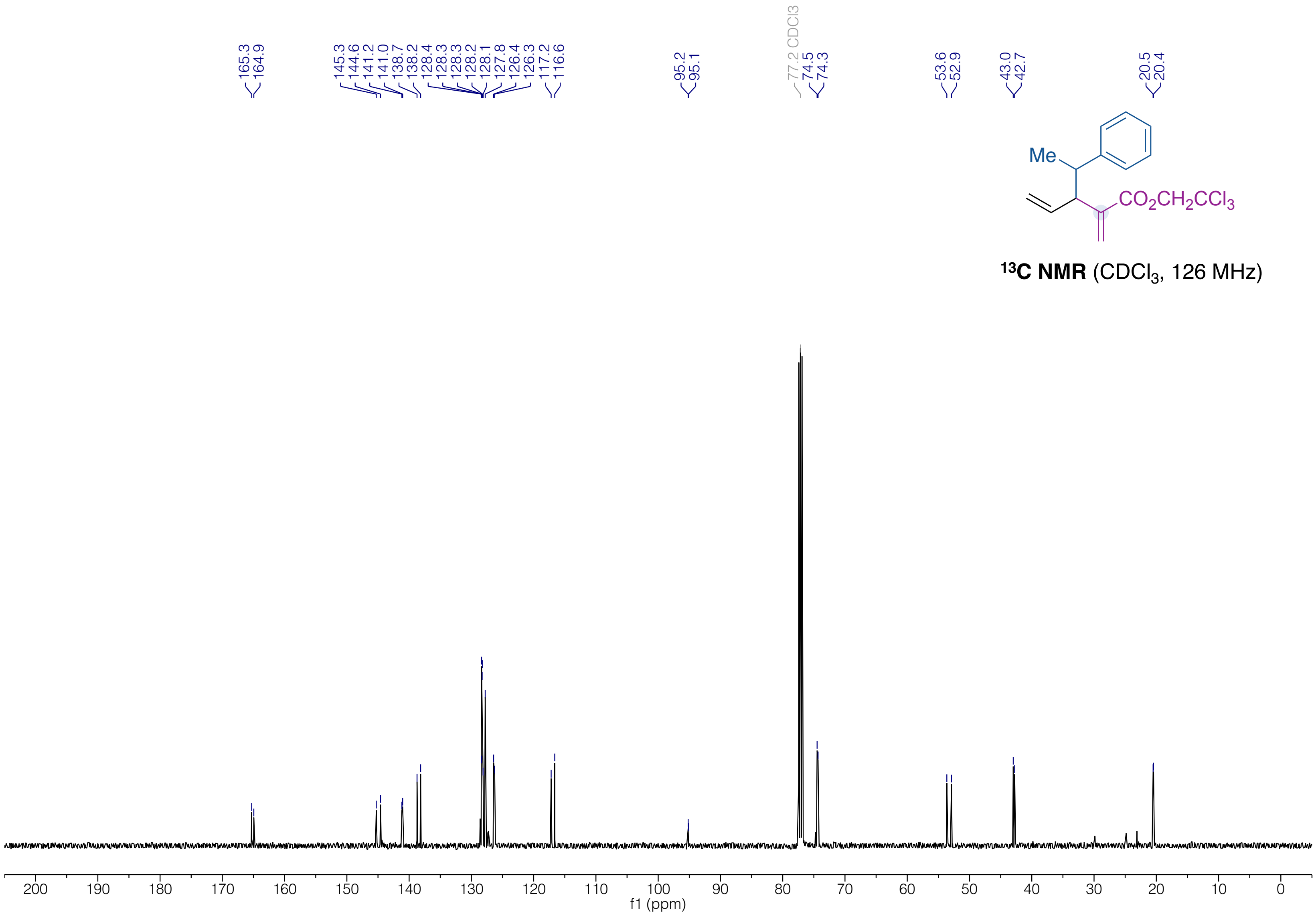

^{13}C NMR ($CDCl_3$, 126 MHz)

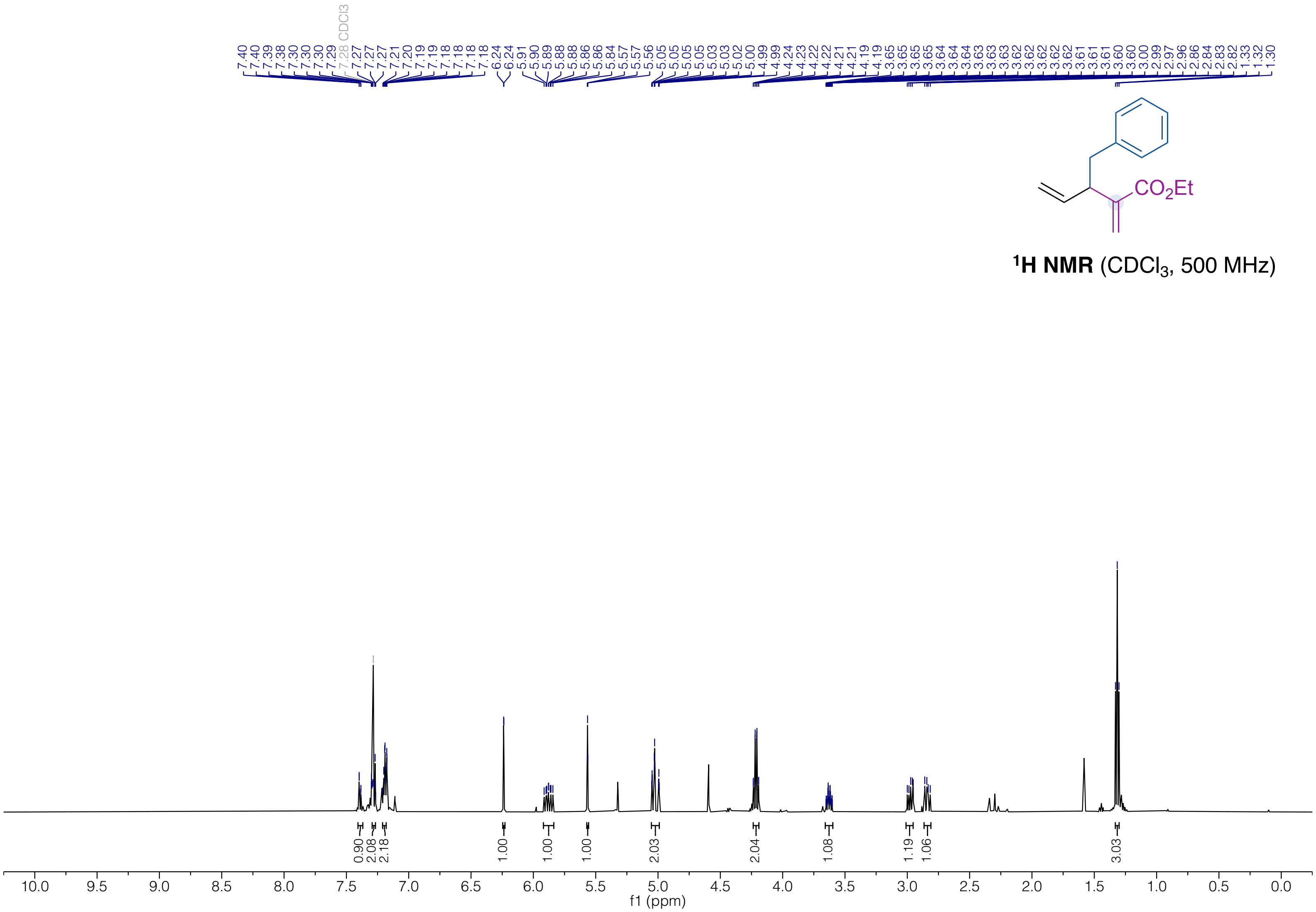

^{1}H NMR ($CDCl_3$, 500 MHz)

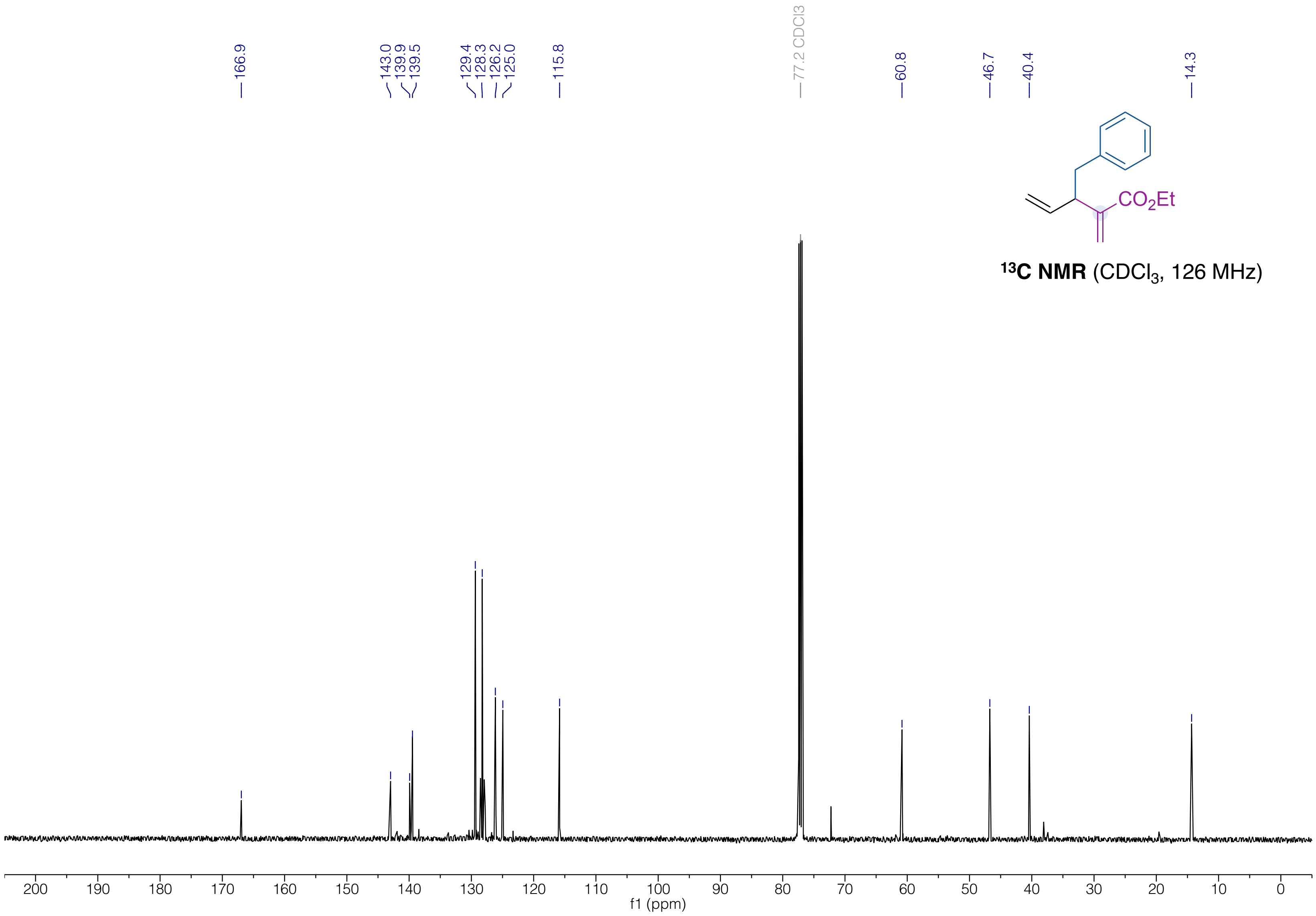
166.9
143.0
139.9
139.5
129.4
128.3
126.2
125.0
115.8
77.2 CDCl3
60.8
46.7
40.4
14.3
CO2Et
13C NMR (CDCl3, 126 MHz)
200
190
180
170
160
150
140
130
120
110
100
90
80
70
60
50
40
30
20
10
0
f1 (ppm)

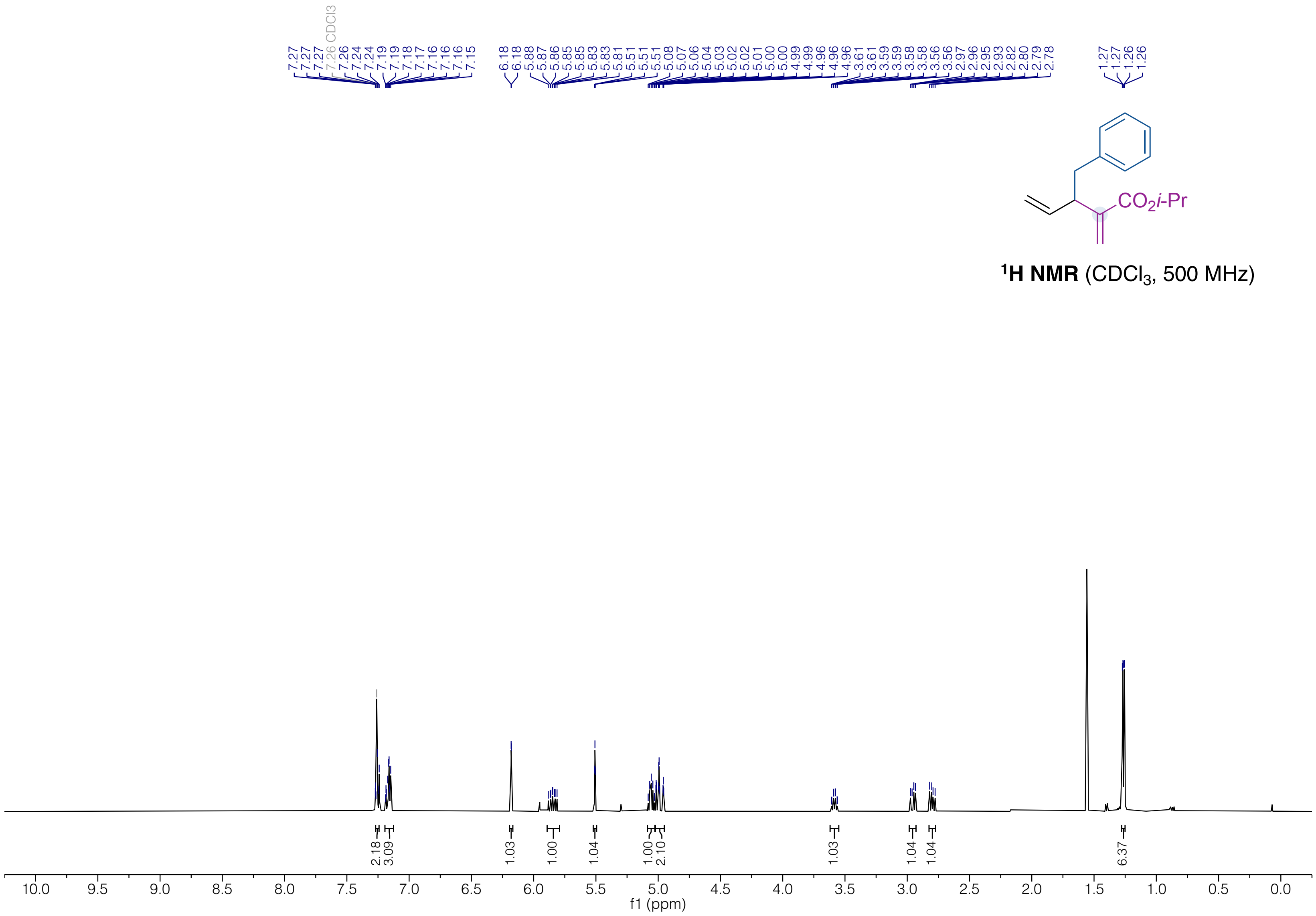

^{1}H NMR ($CDCl_3$, 500 MHz)

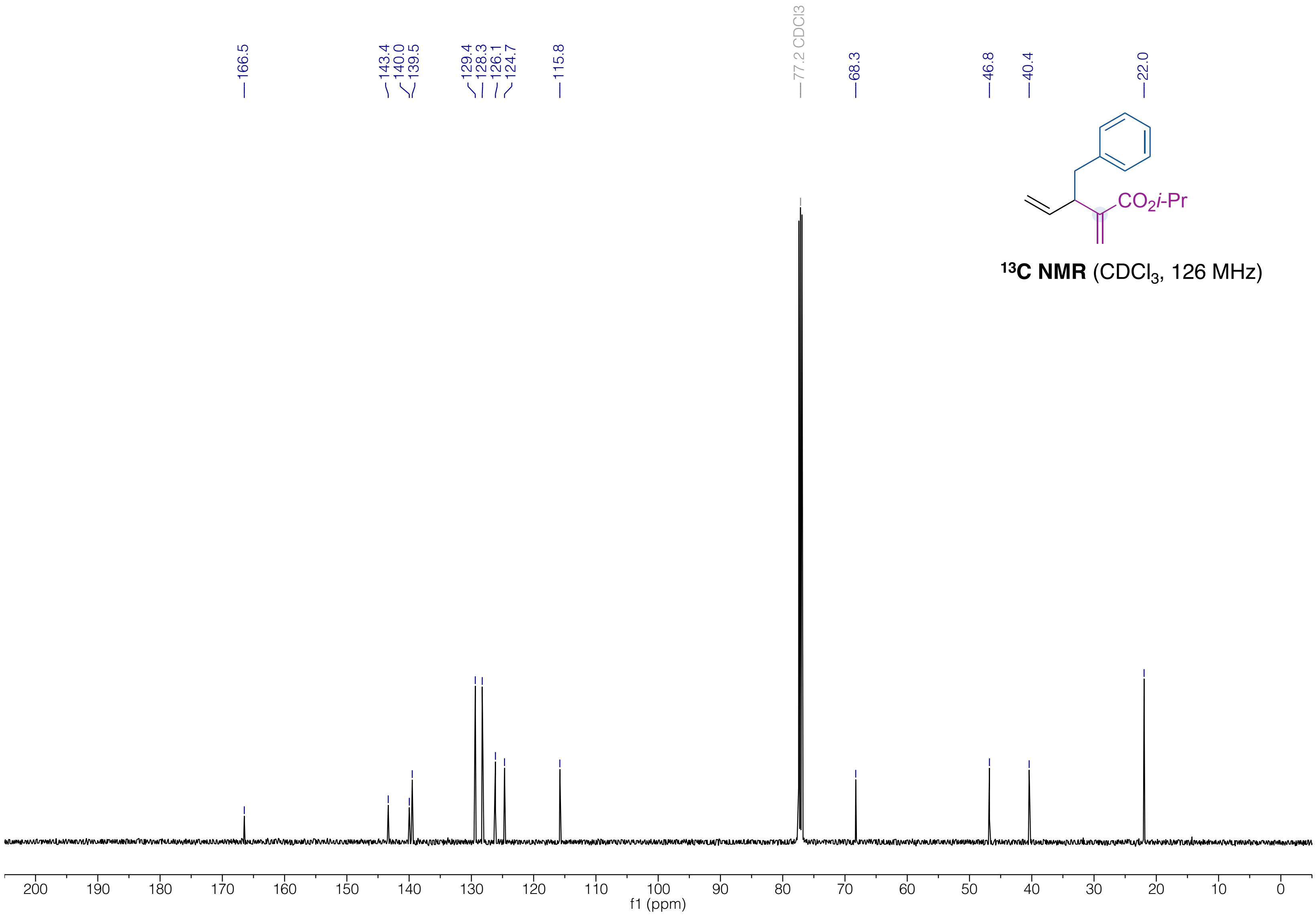
166.5
143.4
140.0
139.5
129.4
128.3
126.1
124.7
115.8
77.2 CDCl3
68.3
46.8
40.4
22.0
CO_2i-Pr
^{13}C NMR ($CDCl_3$, 126 MHz)
f1 (ppm)

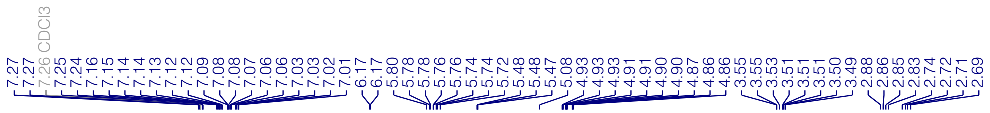

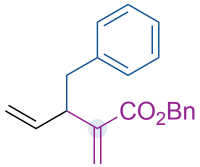

¹H NMR ($CDCl_3$, 400 MHz)

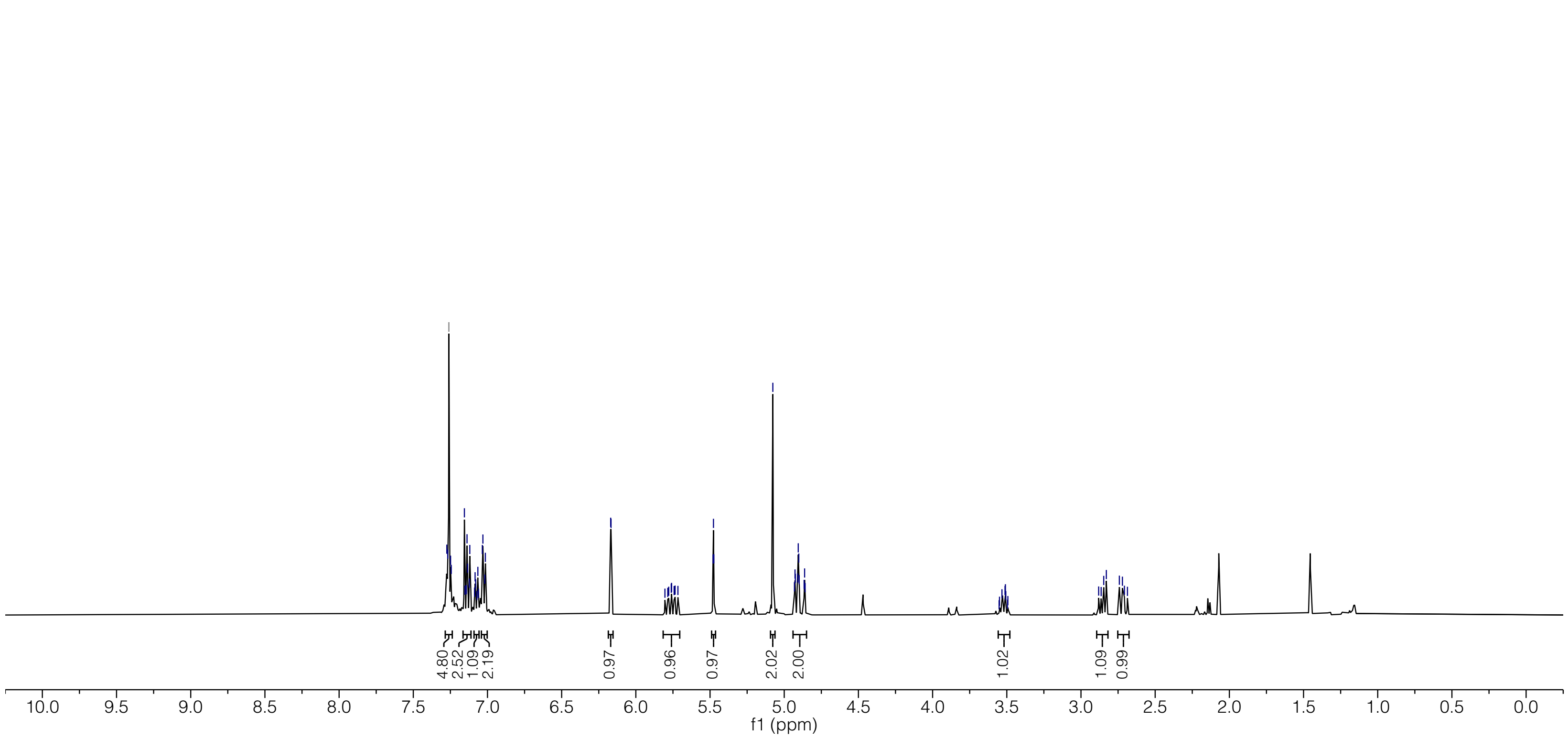

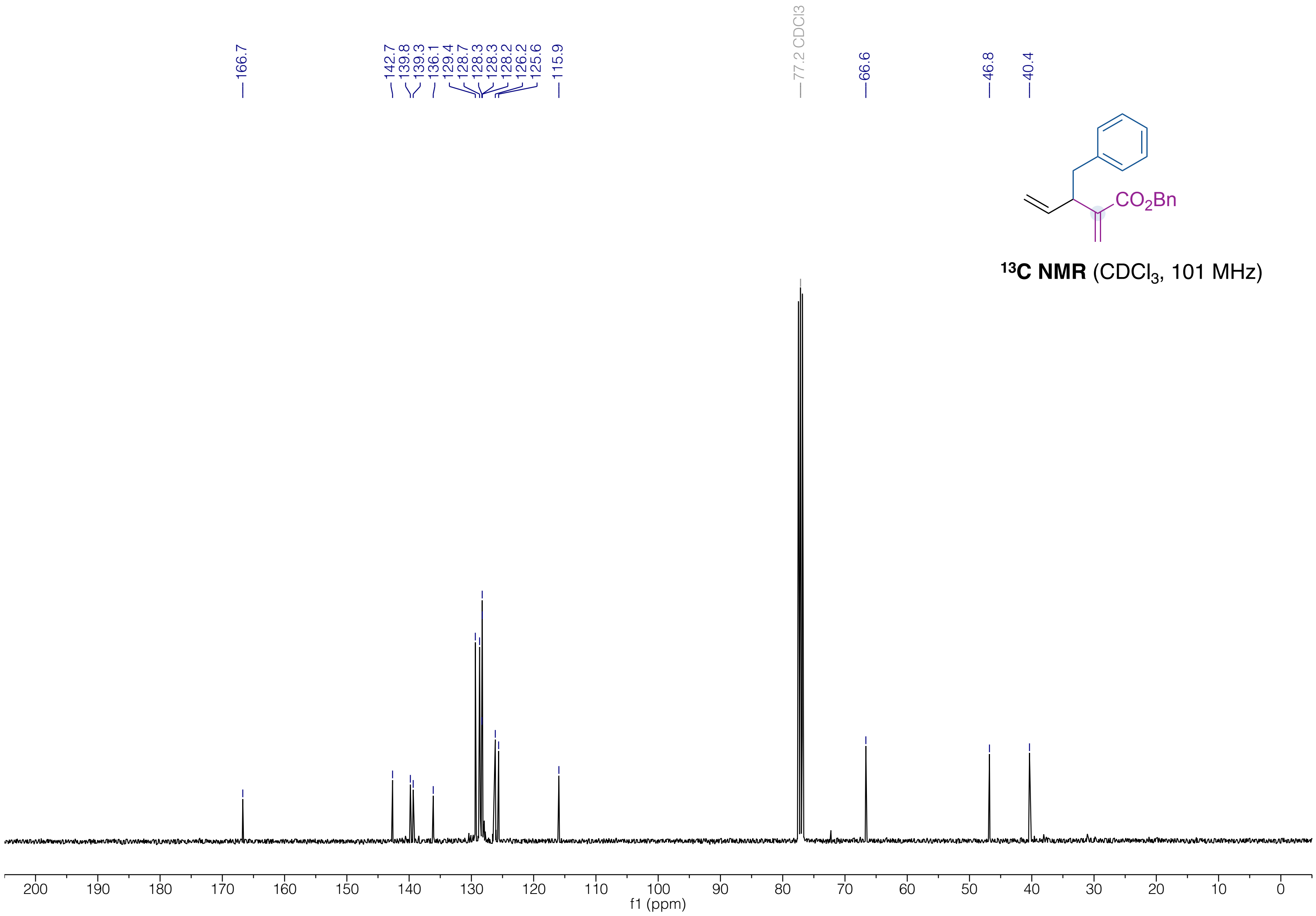
166.7
142.7
139.8
139.3
136.1
129.4
128.7
128.3
128.3
128.2
126.2
125.6
115.9
77.2 CDCl3
66.6
46.8
40.4
CO2Bn
13C NMR (CDCl3, 101 MHz)
200
190
180
170
160
150
140
130
120
110
100
90
80
70
60
50
40
30
20
10
0
f1 (ppm)

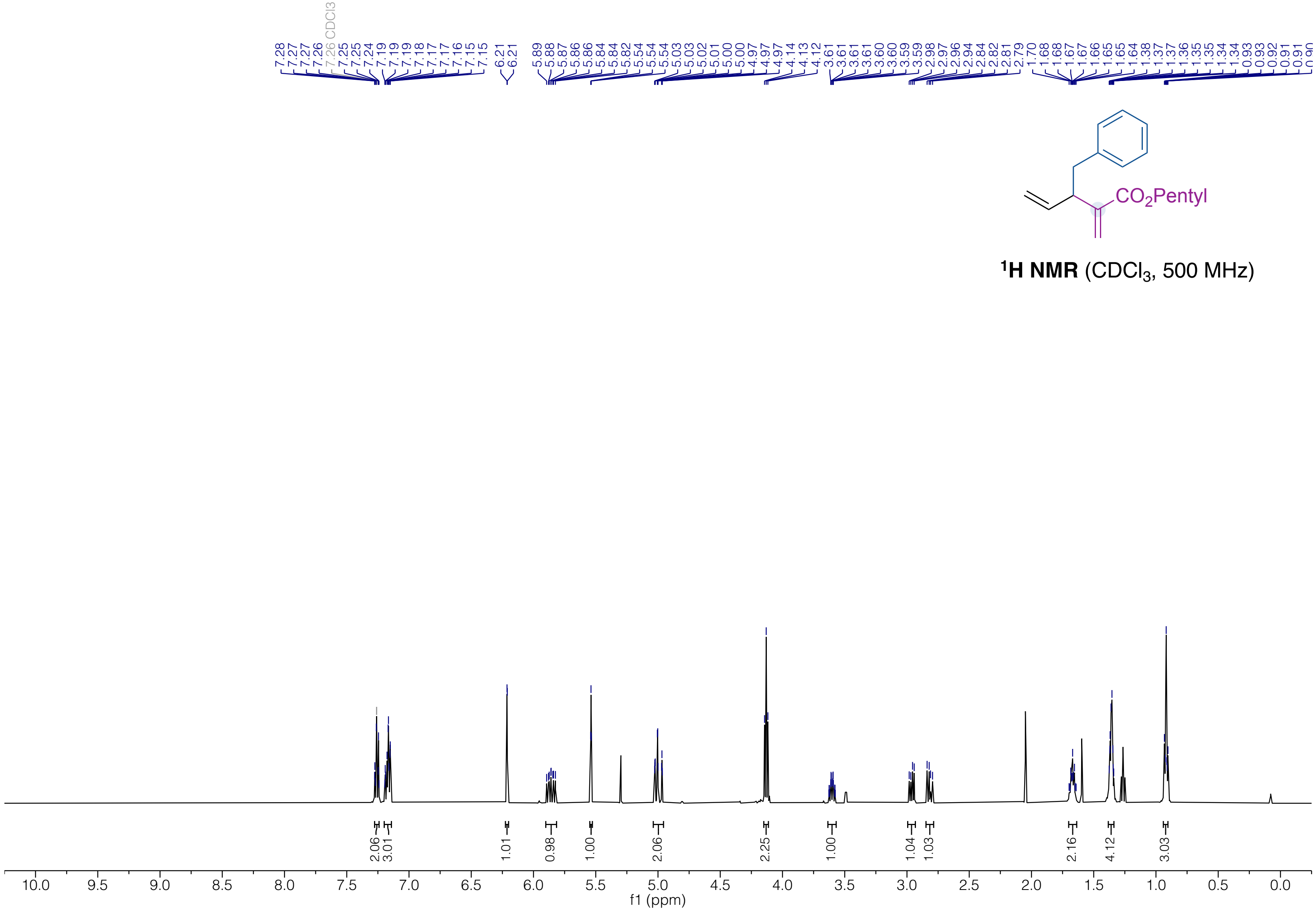
7.28 7.27 7.27 7.26 CDCl3 7.25 7.25 7.24 7.19 7.19 7.19 7.18 7.17 7.17 7.16 7.15 6.21 6.21 5.89 5.88 5.87 5.86 5.86 5.84 5.84 5.82 5.54 5.54 5.54 5.03 5.03 5.02 5.01 5.00 5.00 4.97 4.97 4.14 4.13 4.12 3.61 3.61 3.61 3.60 3.60 3.59 2.98 2.97 2.96 2.94 2.84 2.82 2.81 2.79 1.70 1.68 1.68 1.67 1.66 1.65 1.65 1.64 1.38 1.37 1.37 1.36 1.35 1.34 1.34 0.93 0.93 0.92 0.91 0.91
CO2Pentyl
1H NMR (CDCl3, 500 MHz)
2.06 3.01 1.01 0.98 1.00 2.06 2.25 1.00 1.04 1.03 2.16 4.12 3.03
10.0 9.5 9.0 8.5 8.0 7.5 7.0 6.5 6.0 5.5 5.0 4.5 4.0 3.5 3.0 2.5 2.0 1.5 1.0 0.5 0.0
f1 (ppm)

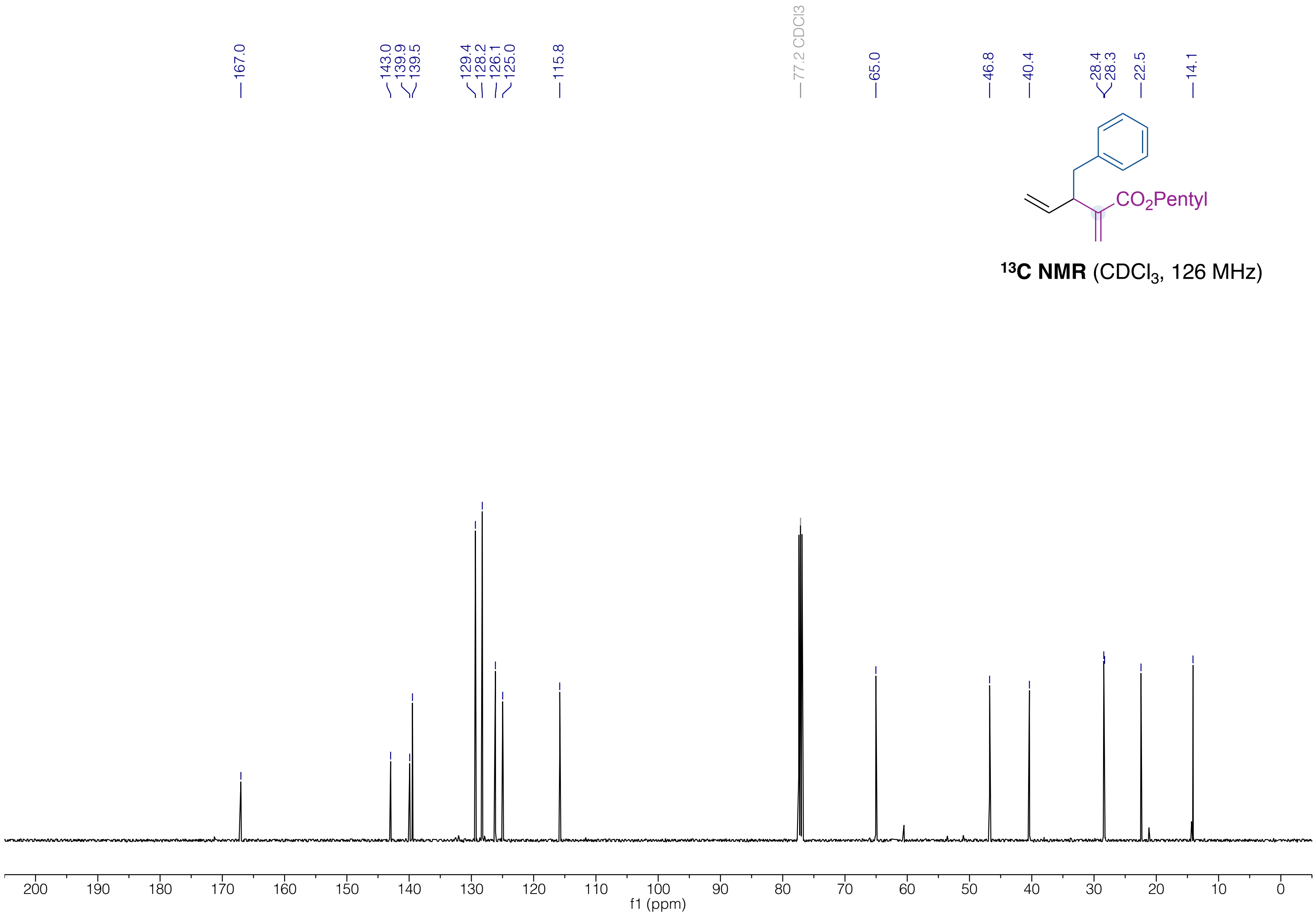
167.0
143.0
139.9
139.5
129.4
128.2
126.1
125.0
115.8
77.2 CDCl3
65.0
46.8
40.4
28.4
28.3
22.5
14.1
CO2Pentyl
13C NMR (CDCl3, 126 MHz)
f1 (ppm)

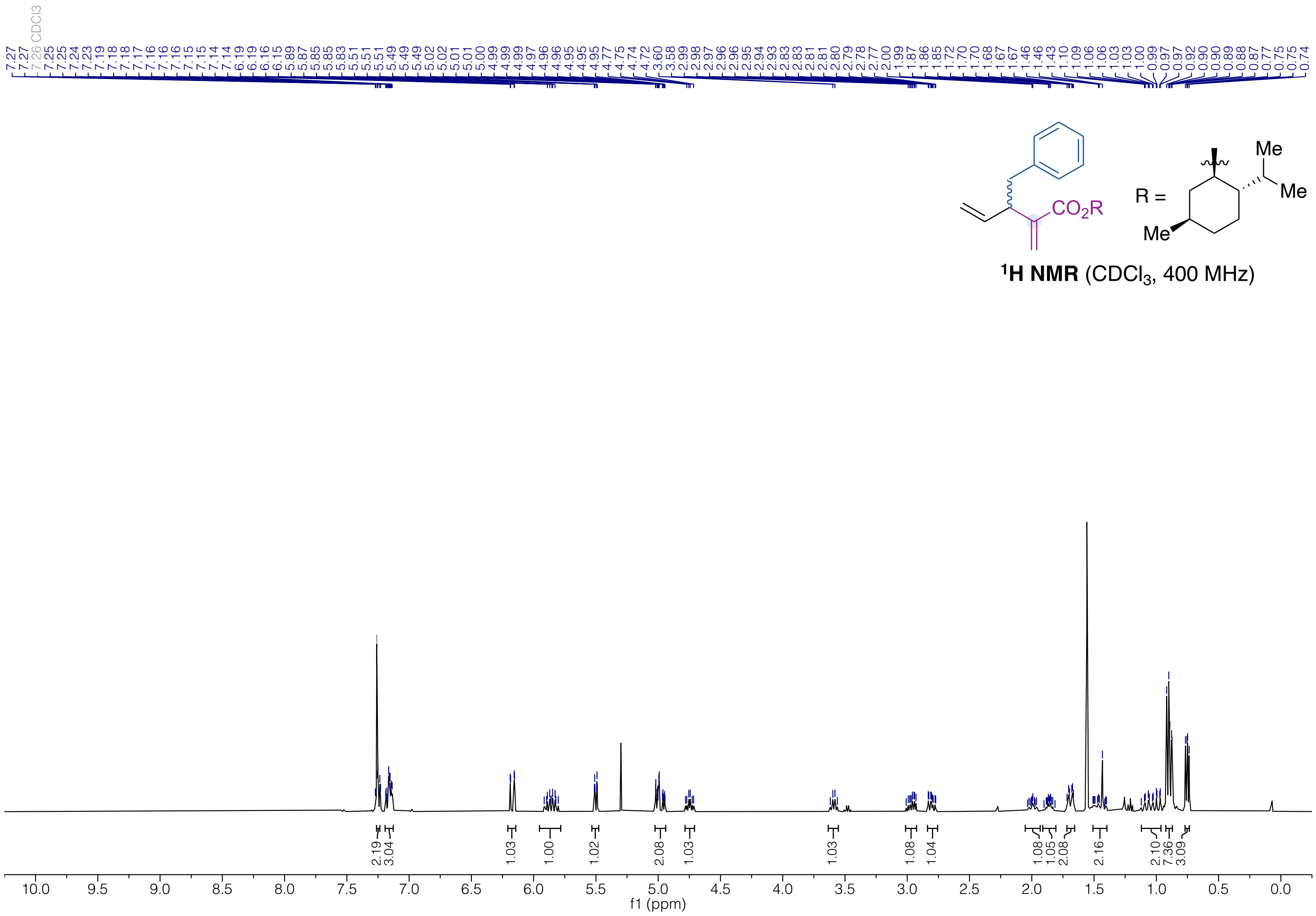

R =
Me
Me
Me
CO_2R
1H NMR ($CDCl_3$, 400 MHz)
f1 (ppm)

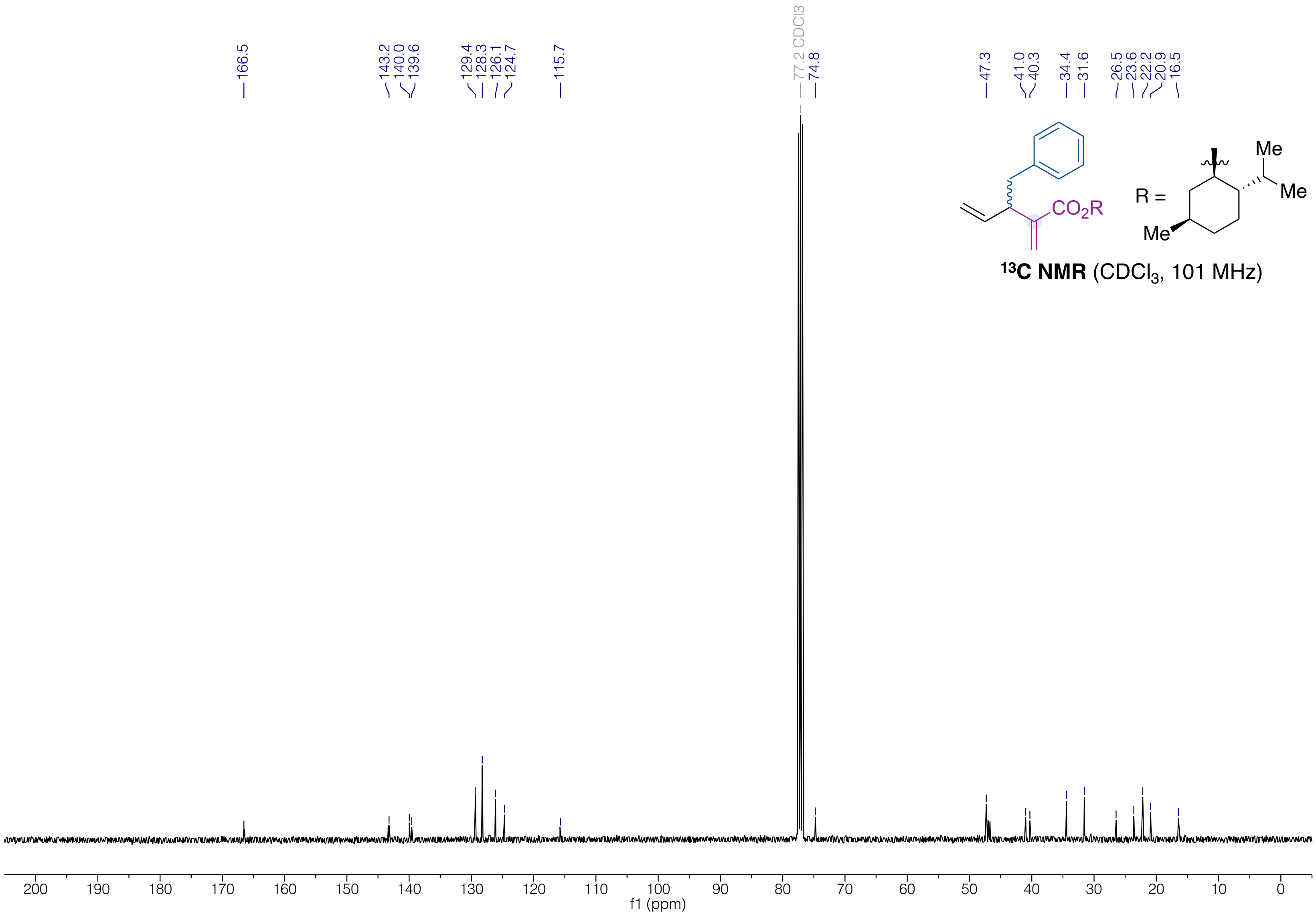
166.5
143.2
140.0
139.6
129.4
128.3
126.1
124.7
115.7
77.2 CDCl3
74.8
47.3
41.0
40.3
34.4
31.6
26.5
23.6
22.2
20.9
16.5
CO2R
R =
Me
Me
Me
13C NMR (CDCl3, 101 MHz)
200
190
180
170
160
150
140
130
120
110
100
90
80
70
60
50
40
30
20
10
0
f1 (ppm)

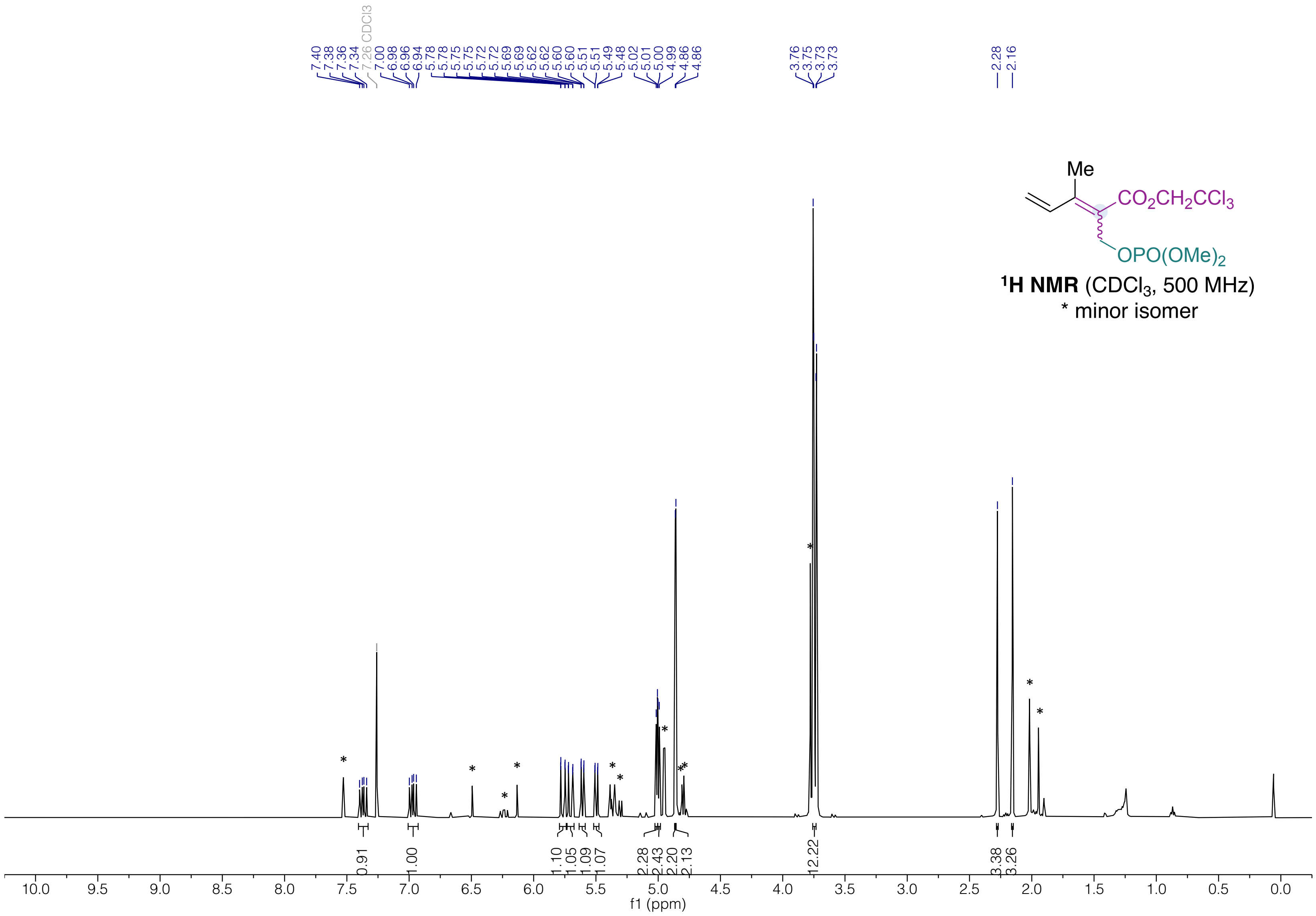

7.40 7.38 7.36 7.34 7.26 CDCl3 7.00 6.98 6.96 6.94 5.78 5.78 5.75 5.75 5.72 5.72 5.69 5.69 5.62 5.62 5.60 5.60 5.51 5.51 5.49 5.48 5.02 5.01 5.00 4.99 4.86 4.86
3.76 3.75 3.73 3.73
2.28 2.16
Me
CO2CH2CCl3
OPO(OMe)2
1H NMR (CDCl3, 500 MHz)
* minor isomer
0.91 1.00 1.10 1.05 1.09 1.07 2.28 2.43 2.20 2.13 12.22 3.38 3.26
10.0 9.5 9.0 8.5 8.0 7.5 7.0 6.5 6.0 5.5 5.0 4.5 4.0 3.5 3.0 2.5 2.0 1.5 1.0 0.5 0.0
f1 (ppm)

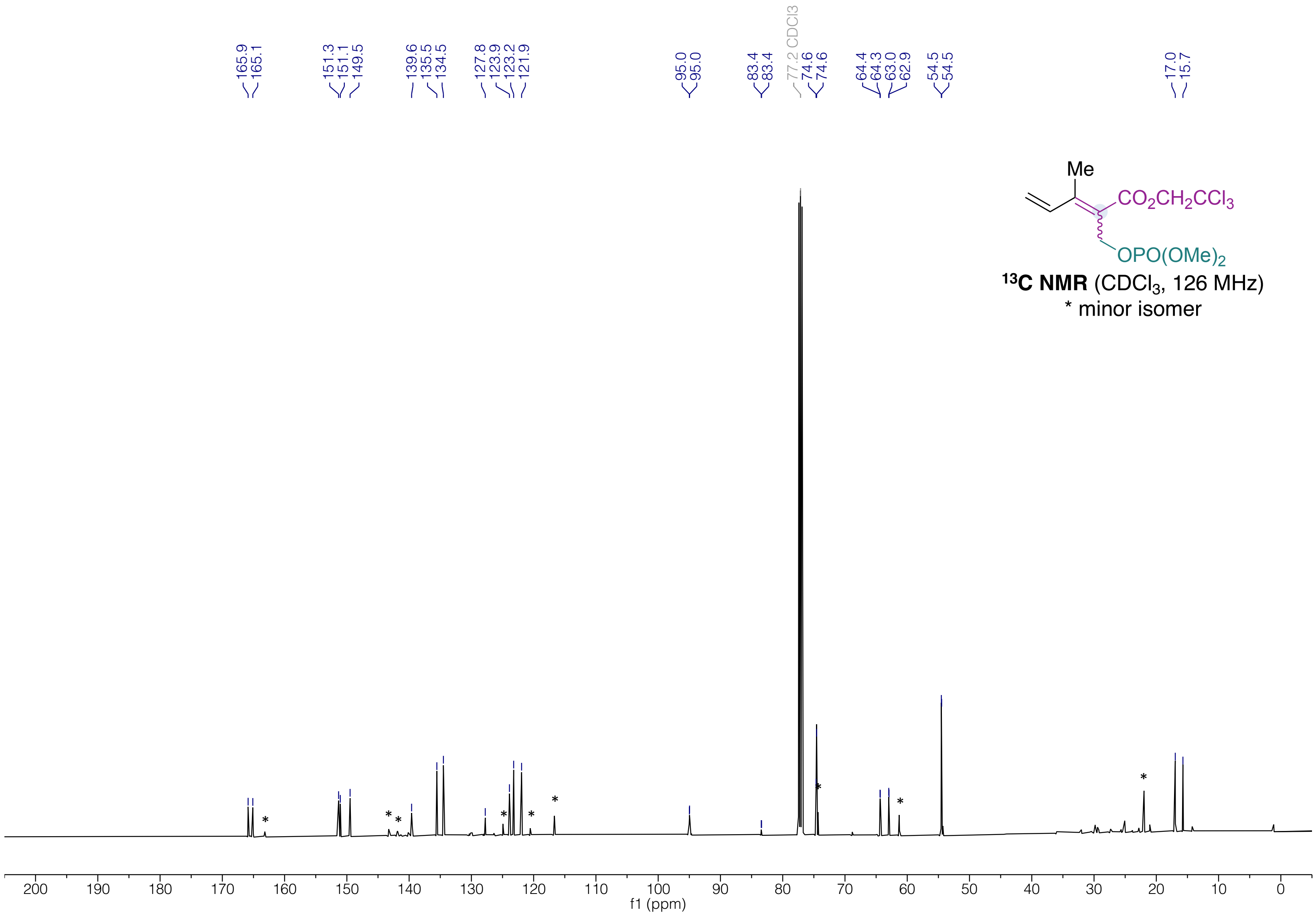
165.9
165.1
151.3
151.1
149.5
139.6
135.5
134.5
127.8
123.9
123.2
121.9
95.0
95.0
83.4
83.4
77.2 CDCl3
74.6
74.6
64.4
64.3
63.0
62.9
54.5
54.5
17.0
15.7
Me
CO2CH2CCl3
OPO(OMe)2
13C NMR (CDCl3, 126 MHz)
* minor isomer
200 190 180 170 160 150 140 130 120 110 100 90 80 70 60 50 40 30 20 10 0
f1 (ppm)

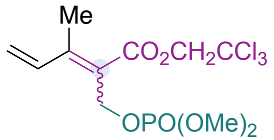

^{31}P NMR ($CDCl_3$, 202 MHz)
* minor isomer

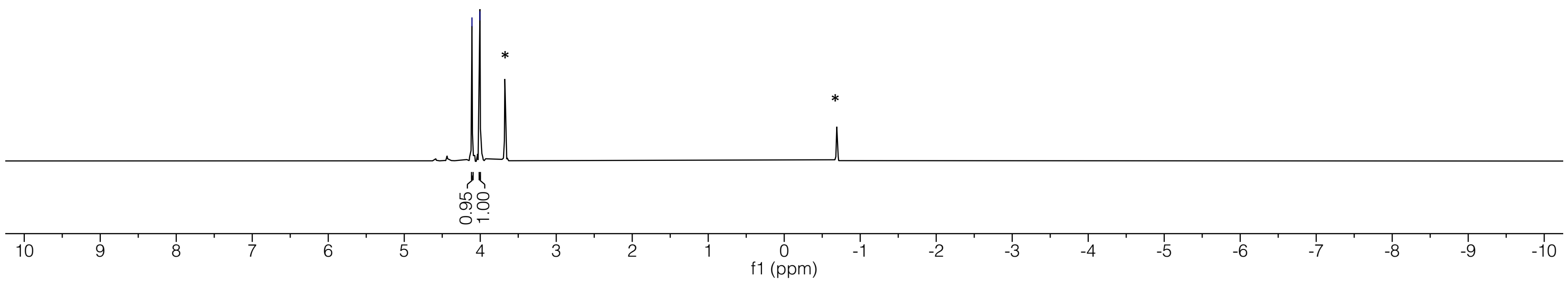

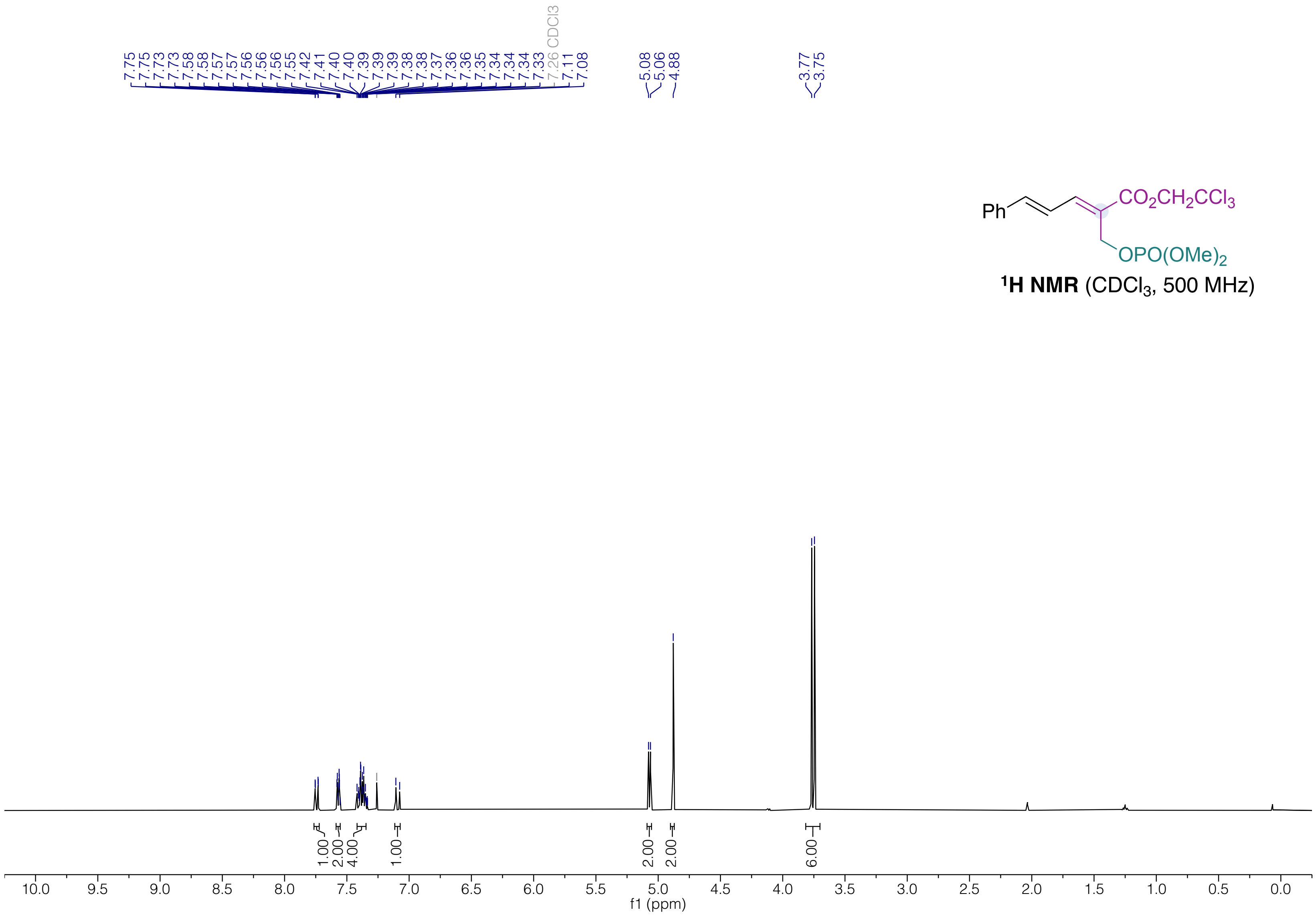
7.75
7.75
7.73
7.73
7.58
7.58
7.57
7.57
7.56
7.56
7.56
7.55
7.42
7.41
7.40
7.40
7.39
7.39
7.39
7.38
7.38
7.37
7.36
7.36
7.35
7.34
7.34
7.34
7.33
7.26 CDCl3
7.11
7.08
5.08
5.06
4.88
3.77
3.75
Ph
CO2CH2CCl3
OPO(OMe)2
1H NMR (CDCl3, 500 MHz)
1.00
2.00
4.00
1.00
2.00
2.00
6.00
10.0
9.5
9.0
8.5
8.0
7.5
7.0
6.5
6.0
5.5
5.0
4.5
4.0
3.5
3.0
2.5
2.0
1.5
1.0
0.5
0.0
f1 (ppm)

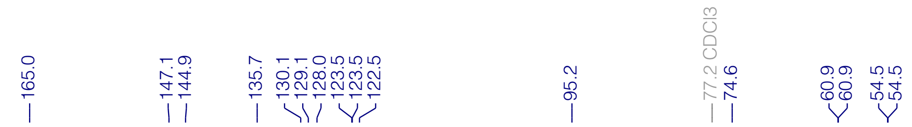

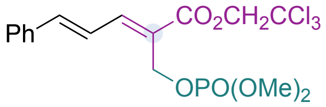

13C NMR ($CDCl_3$, 126 MHz)

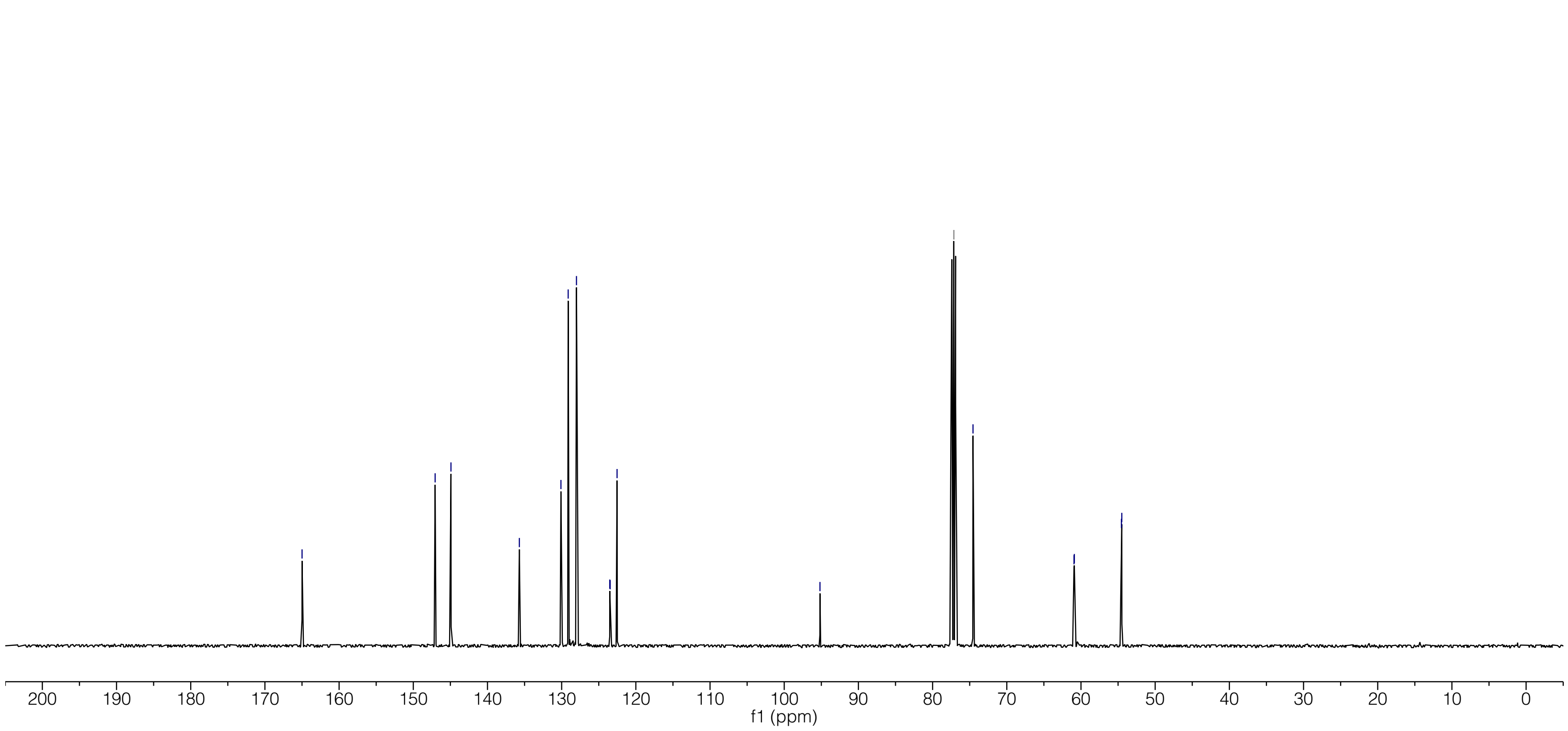

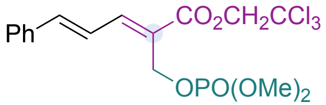

^{31}P NMR ($CDCl_3$, 202 MHz)

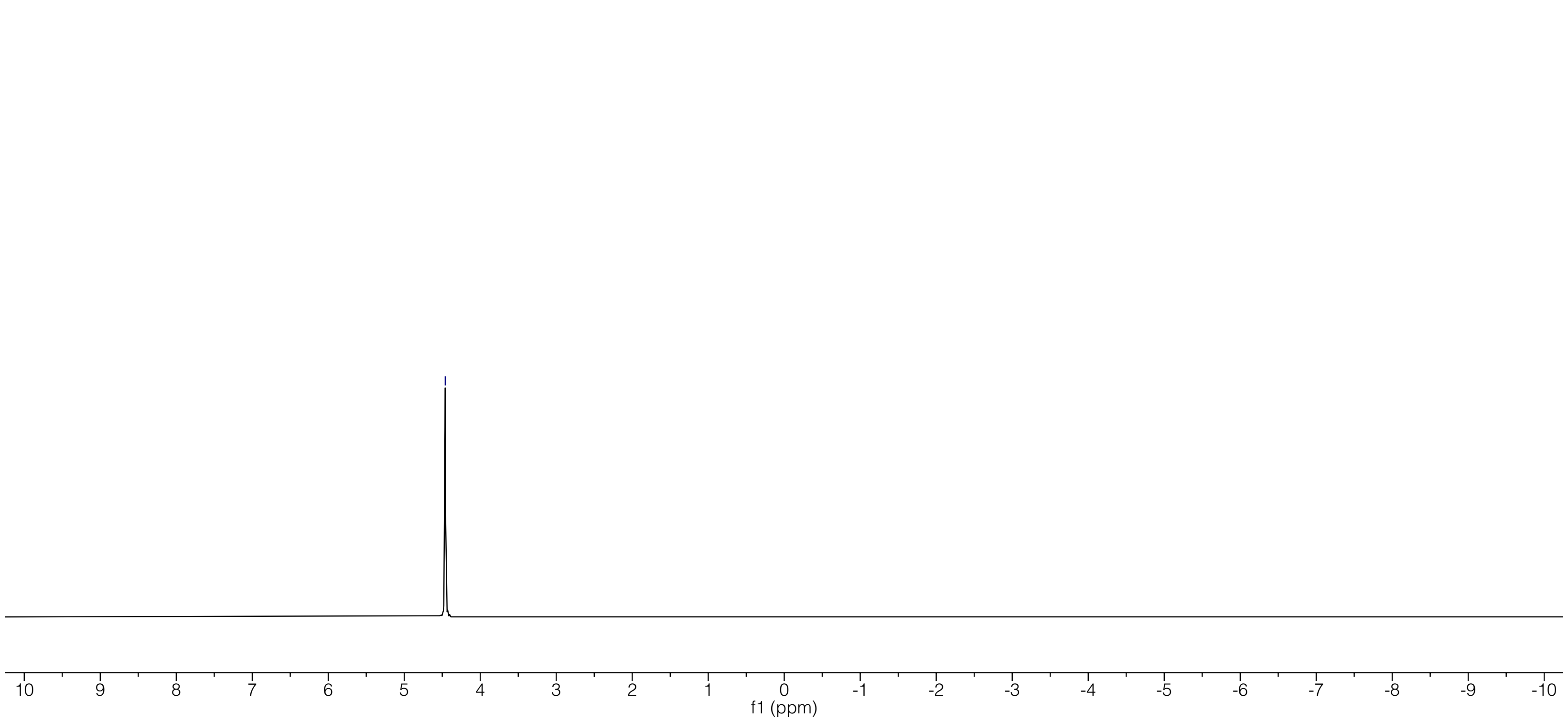

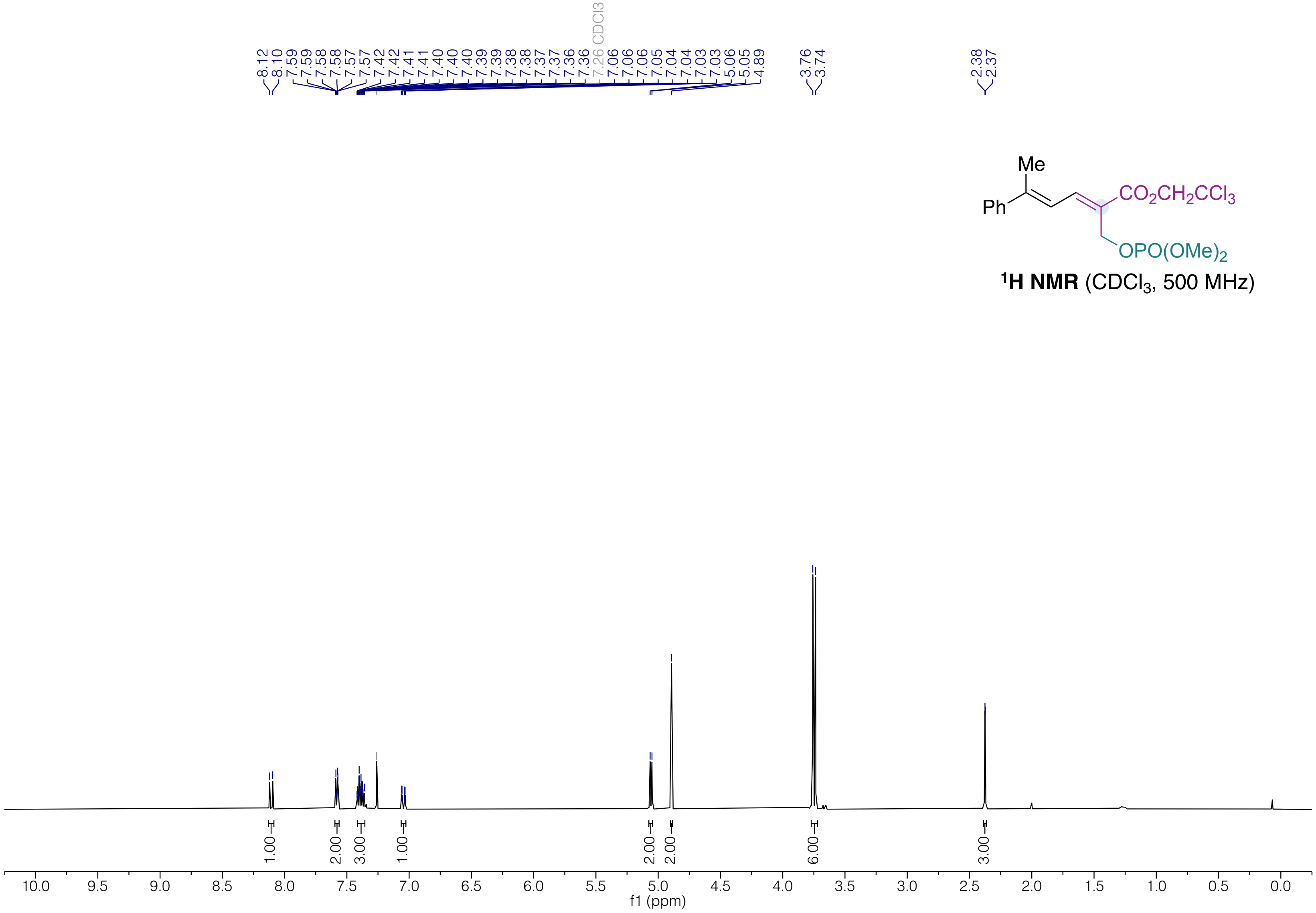

1H NMR ($CDCl_3$, 500 MHz)

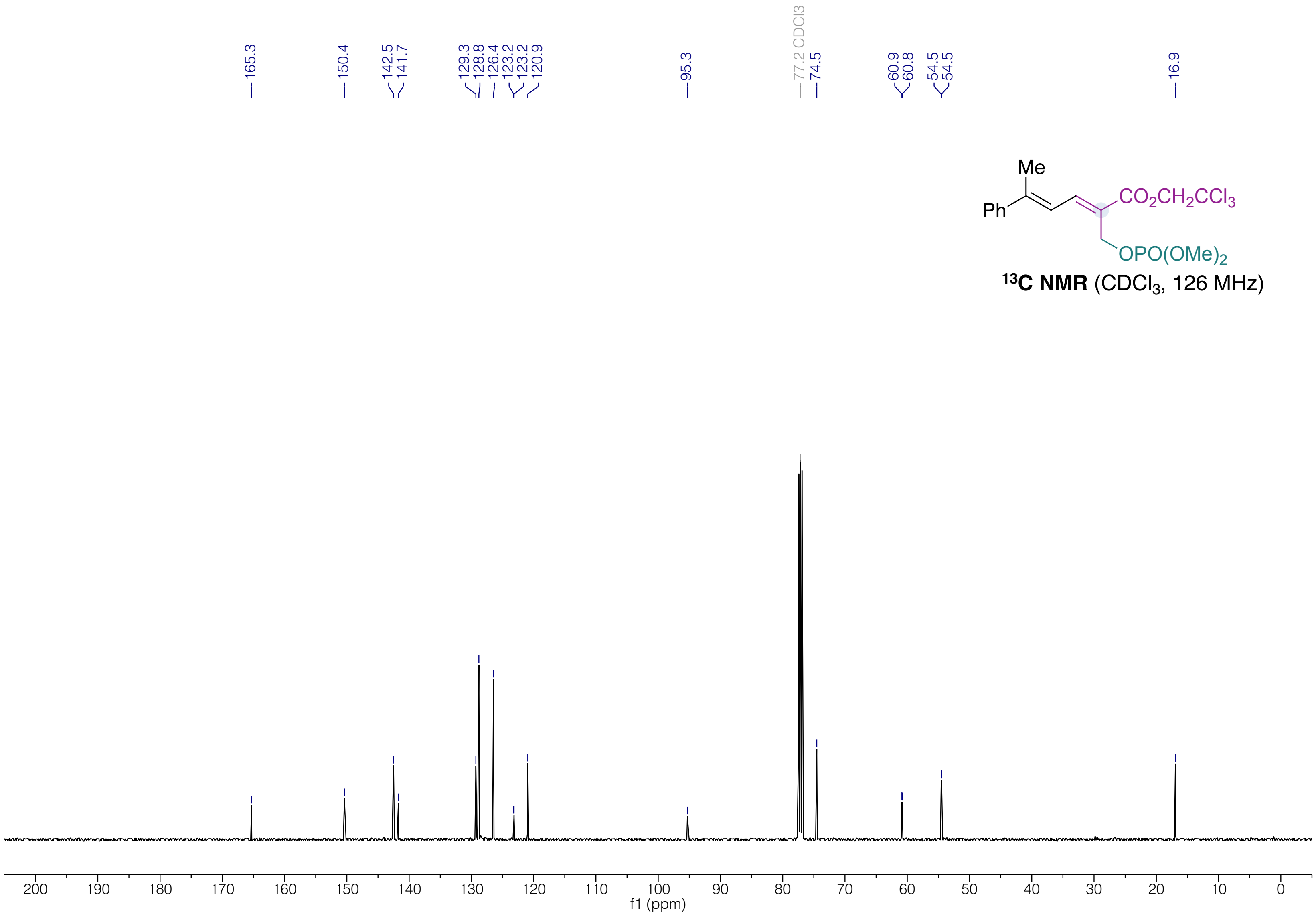
165.3
150.4
142.5
141.7
129.3
128.8
126.4
123.2
123.2
120.9
95.3
77.2 CDCl3
74.5
60.9
60.8
54.5
54.5
16.9
Me
Ph
CO2CH2CCl3
OPO(OMe)2
13C NMR (CDCl3, 126 MHz)
200 190 180 170 160 150 140 130 120 110 100 90 80 70 60 50 40 30 20 10 0
f1 (ppm)

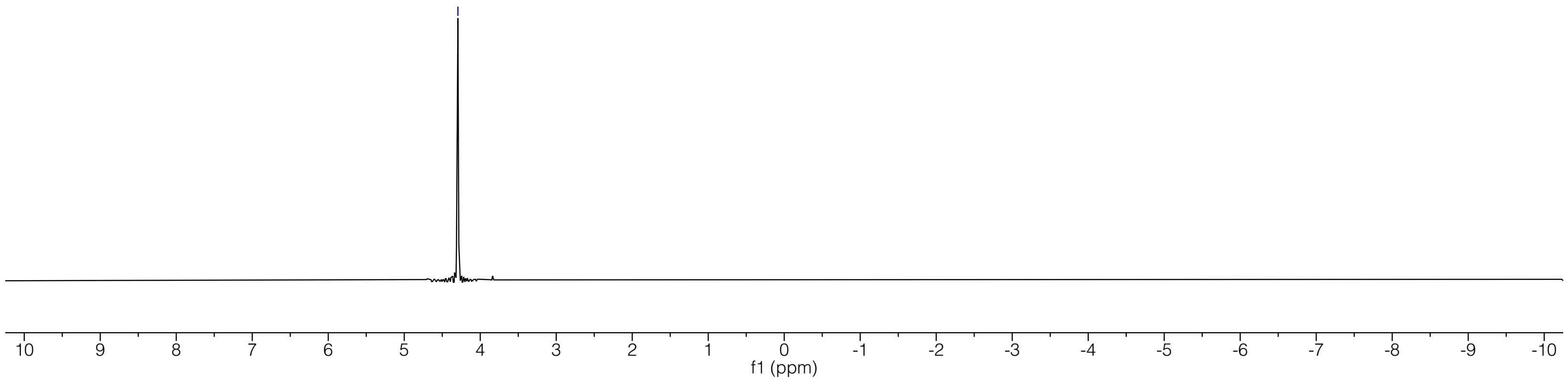

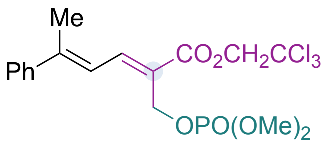

^{31}P NMR ($CDCl_3$, 202 MHz)

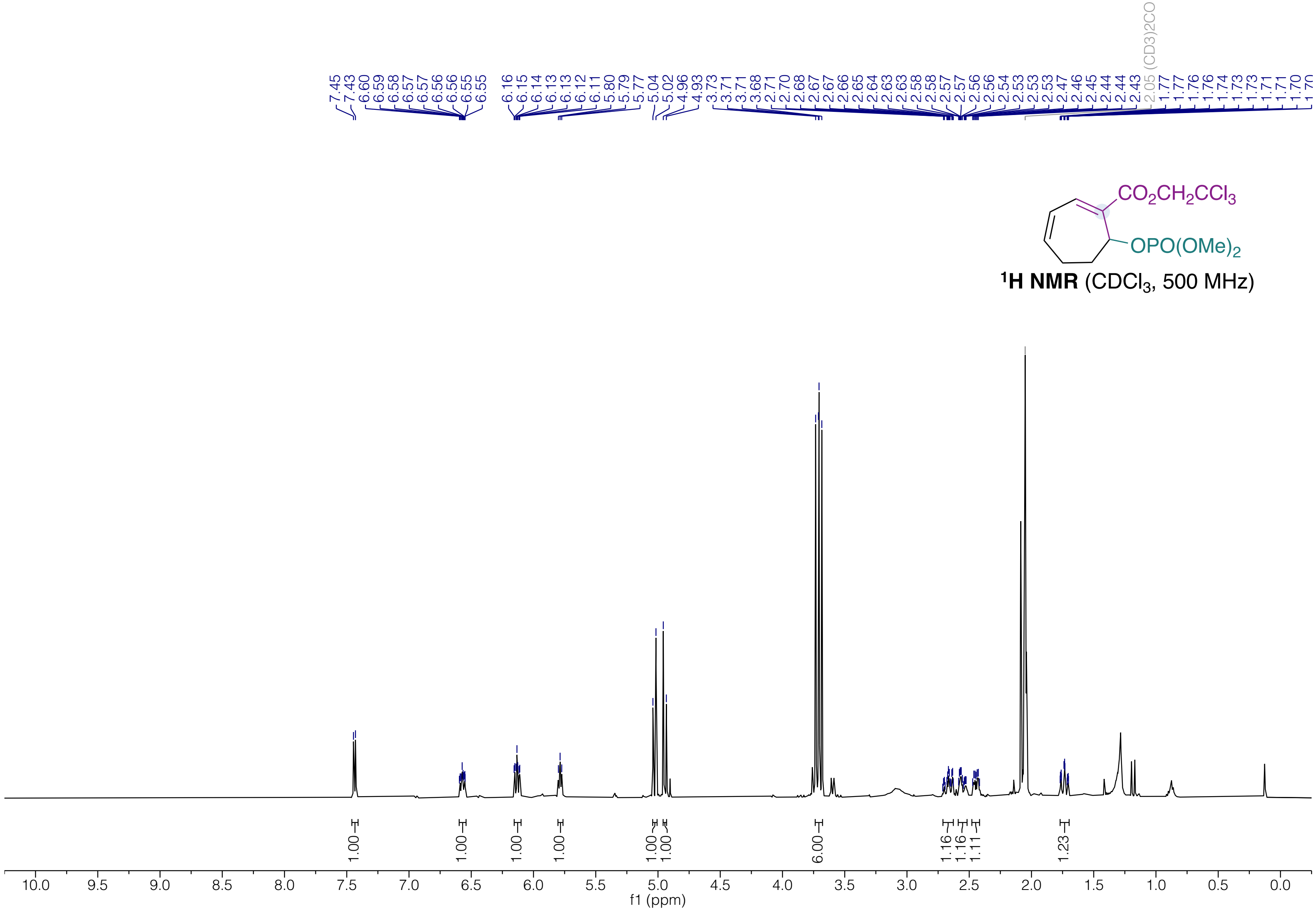
7.45
7.43
6.60
6.59
6.58
6.57
6.57
6.56
6.56
6.55
6.55
6.16
6.15
6.14
6.13
6.13
6.12
6.11
5.80
5.79
5.77
5.04
5.02
4.96
4.93
3.73
3.71
3.71
3.68
2.71
2.70
2.68
2.67
2.67
2.66
2.65
2.64
2.63
2.63
2.58
2.58
2.57
2.57
2.56
2.56
2.54
2.53
2.53
2.53
2.47
2.46
2.45
2.44
2.44
2.43
2.05 (CD3)2CO
1.77
1.77
1.76
1.76
1.74
1.73
1.73
1.71
1.71
1.70
1.70
CO2CH2CCl3
OPO(OMe)2
1H NMR (CDCl3, 500 MHz)
1.00
1.00
1.00
1.00
1.00
1.00
6.00
1.16
1.16
1.11
1.23
10.0
9.5
9.0
8.5
8.0
7.5
7.0
6.5
6.0
5.5
5.0
4.5
4.0
3.5
3.0
2.5
2.0
1.5
1.0
0.5
0.0
f1 (ppm)

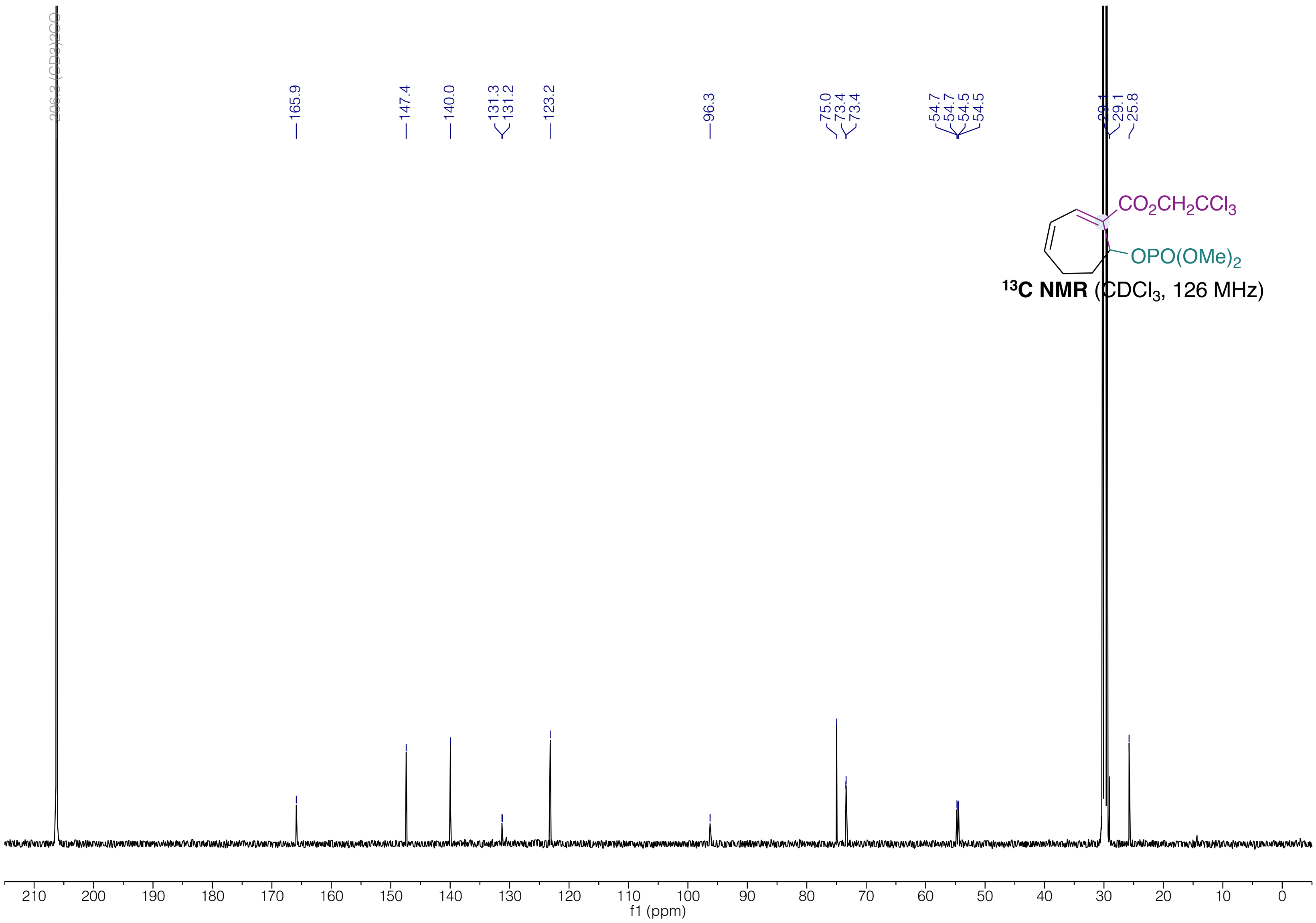
206.3 (CD3)2CO
165.9
147.4
140.0
131.3
131.2
123.2
96.3
75.0
73.4
73.4
54.7
54.7
54.5
54.5
29.1
29.1
25.8
CO2CH2CCl3
OPO(OMe)2
13C NMR (CDCl3, 126 MHz)
f1 (ppm)

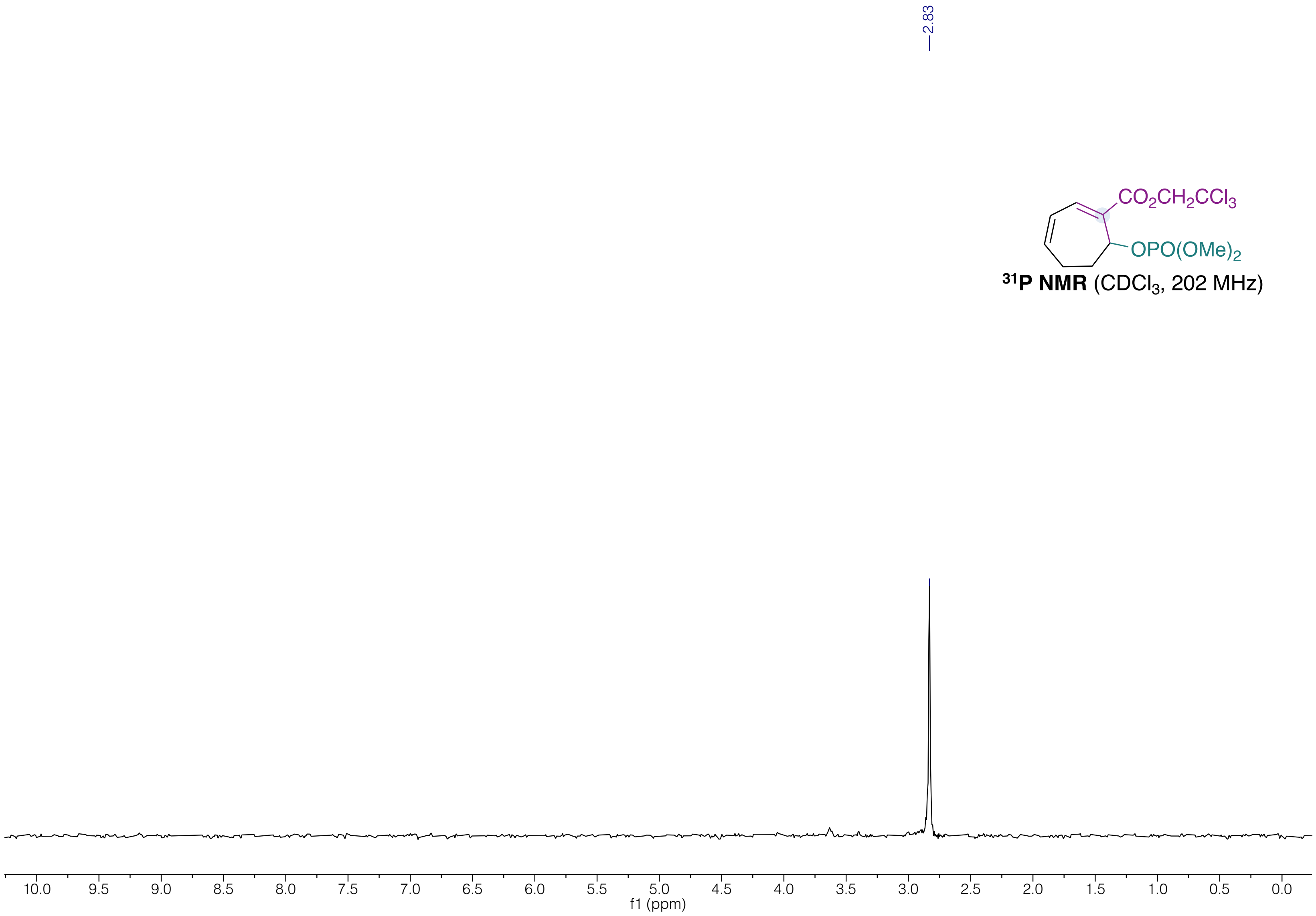

^{31}P NMR ($CDCl_3$, 202 MHz)

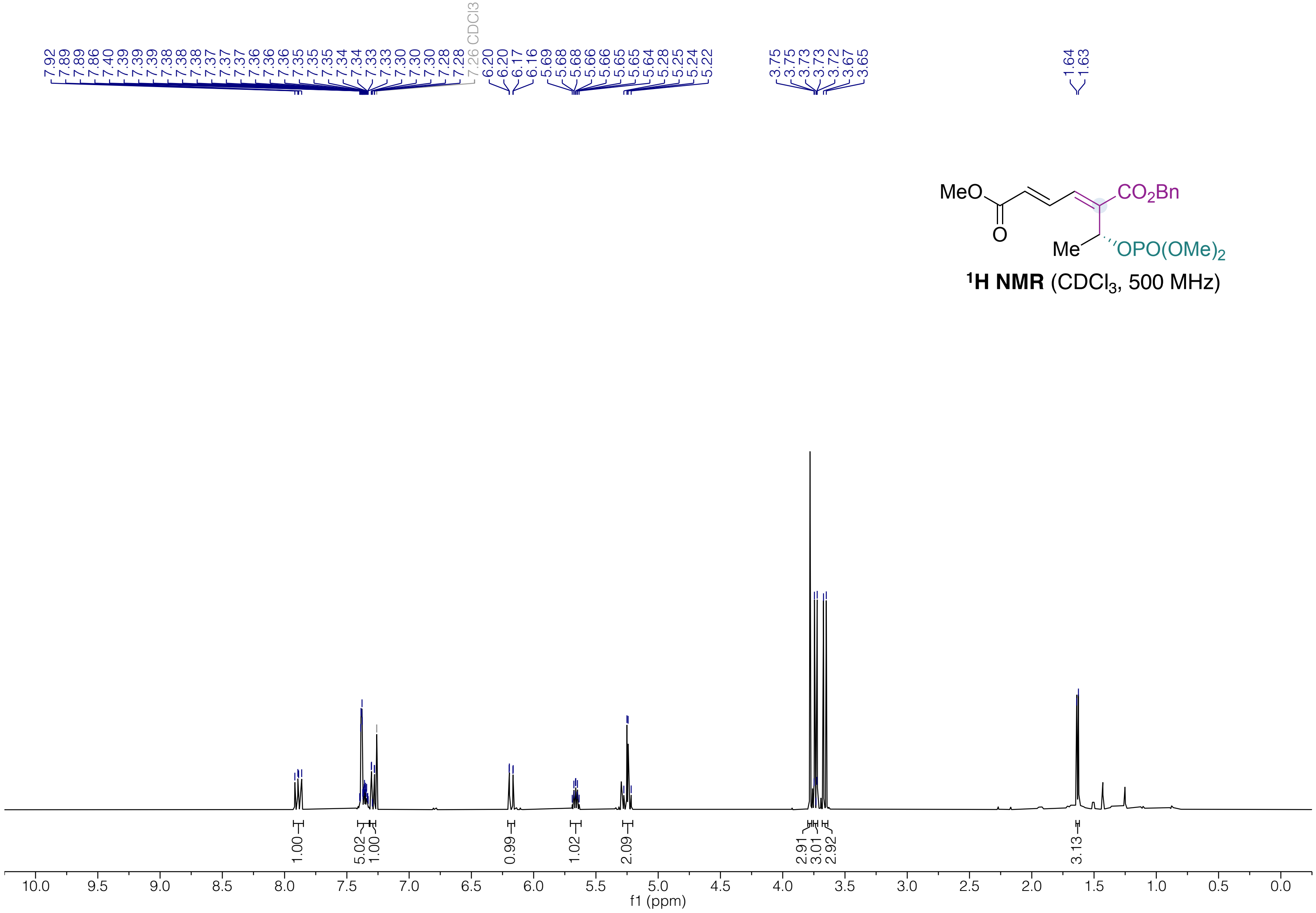

1H NMR ($CDCl_3$, 500 MHz)

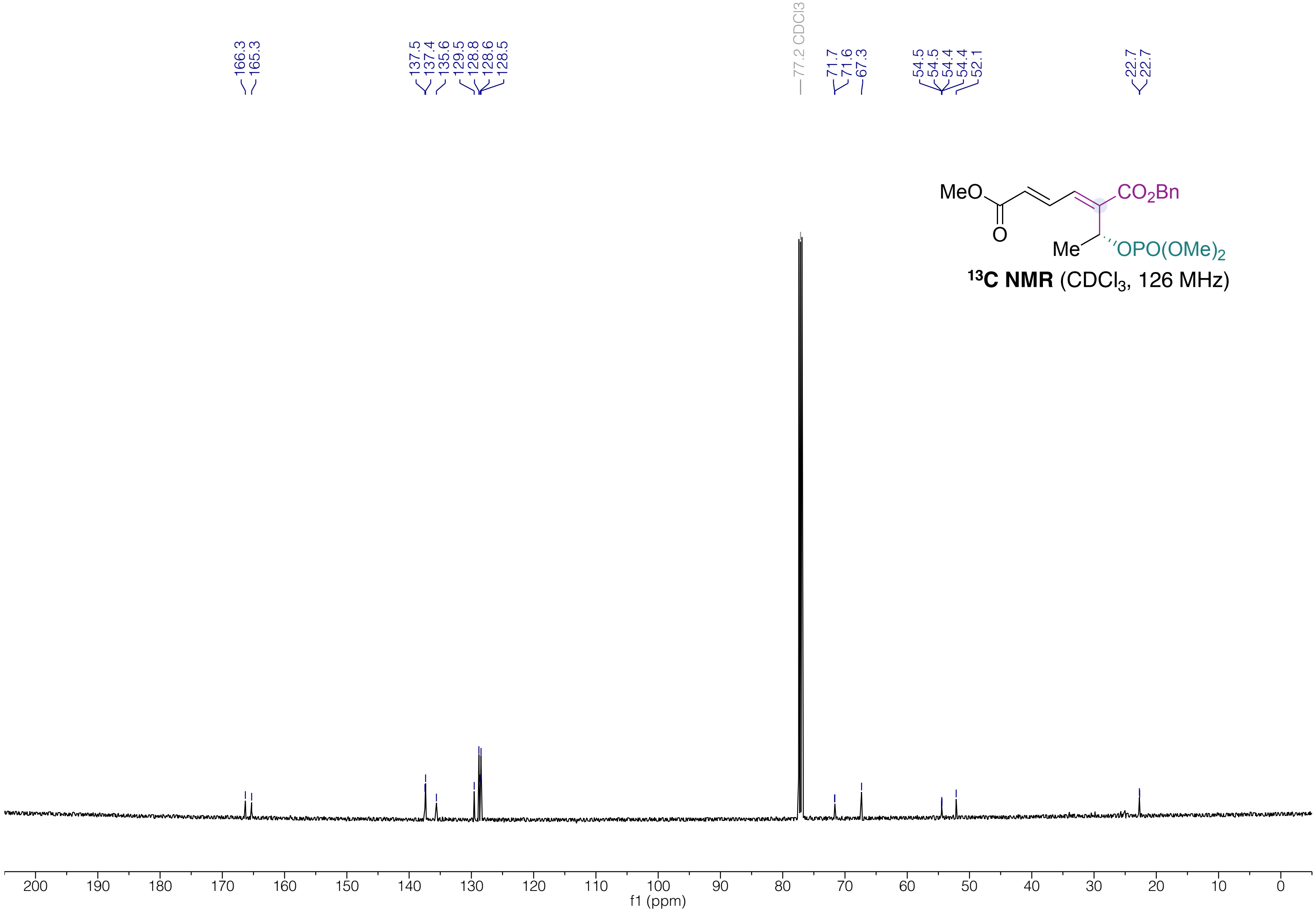

166.3
165.3
137.5
137.4
135.6
129.5
128.8
128.6
128.5
77.2 CDCl3
71.7
71.6
67.3
54.5
54.5
54.4
54.4
52.1
22.7
22.7
MeO
O
CO2Bn
Me
OPO(OMe)2
13C NMR (CDCl3, 126 MHz)
200
190
180
170
160
150
140
130
120
110
100
90
80
70
60
50
40
30
20
10
0
f1 (ppm)

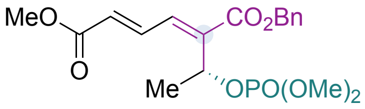

^{31}**P NMR** ($CDCl_3$, 202 MHz)

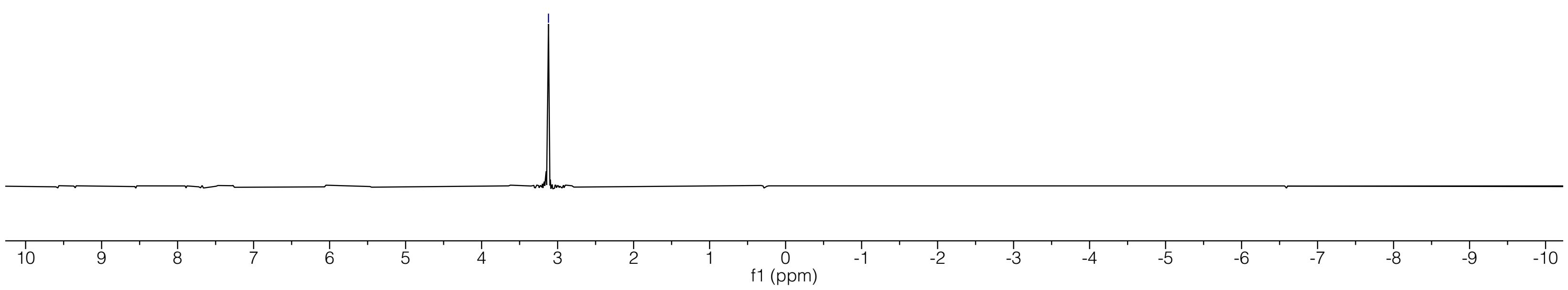

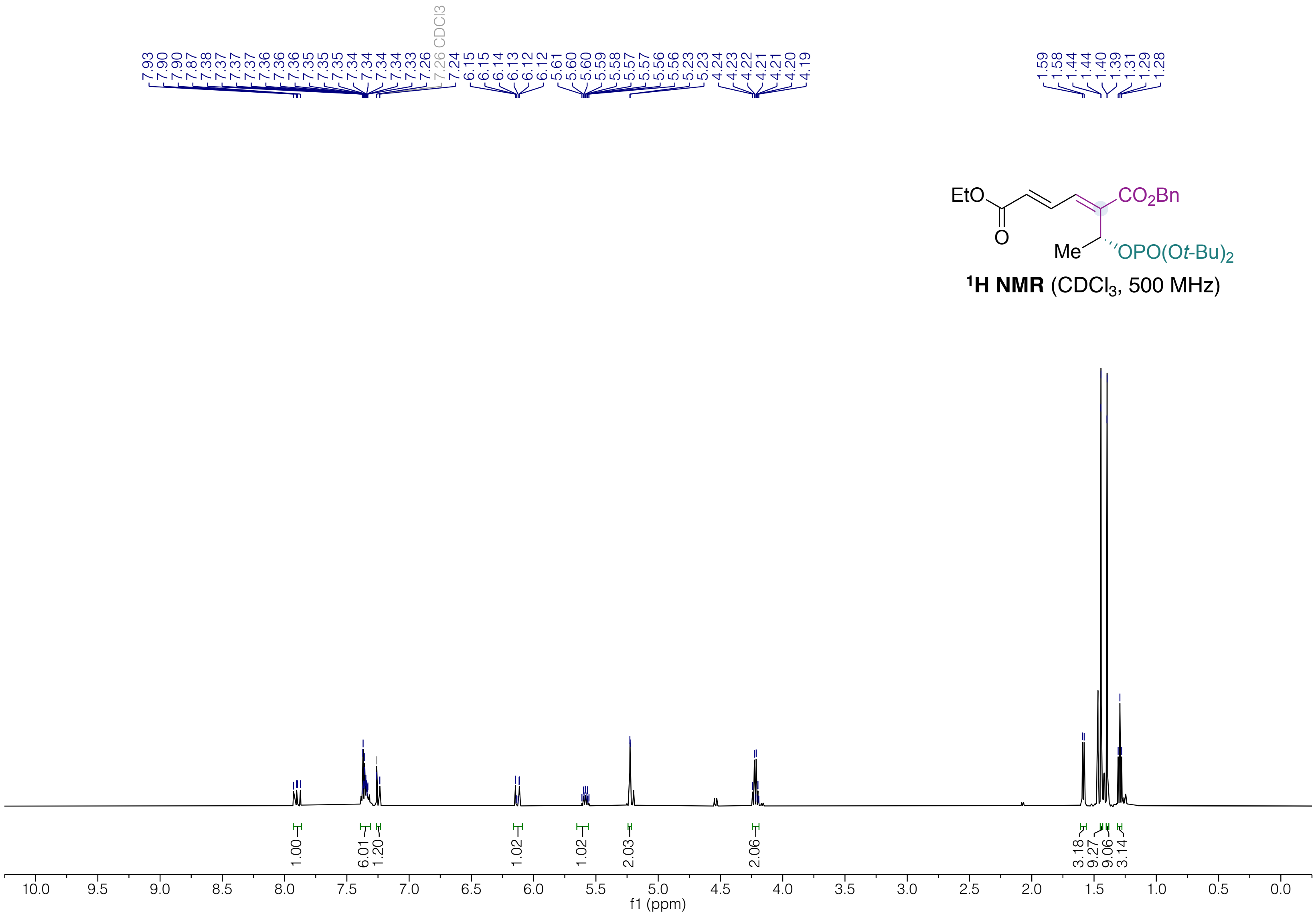
7.93 7.90 7.90 7.87 7.38 7.37 7.37 7.37 7.36 7.36 7.36 7.35 7.35 7.35 7.34 7.34 7.34 7.34 7.33 7.26 7.26 CDCl3 7.24 6.15 6.14 6.13 6.12 6.12 5.61 5.60 5.60 5.59 5.58 5.57 5.57 5.56 5.56 5.23 5.23 4.24 4.23 4.22 4.21 4.21 4.20 4.19
1.59 1.58 1.44 1.44 1.40 1.39 1.31 1.29 1.28
EtO
O
CO2Bn
Me
OPO(Ot-Bu)2
1H NMR (CDCl3, 500 MHz)
1.00 6.01 1.20 1.02 1.02 2.03 2.06 3.18 9.27 9.06 3.14
10.0 9.5 9.0 8.5 8.0 7.5 7.0 6.5 6.0 5.5 5.0 4.5 4.0 3.5 3.0 2.5 2.0 1.5 1.0 0.5 0.0
f1 (ppm)

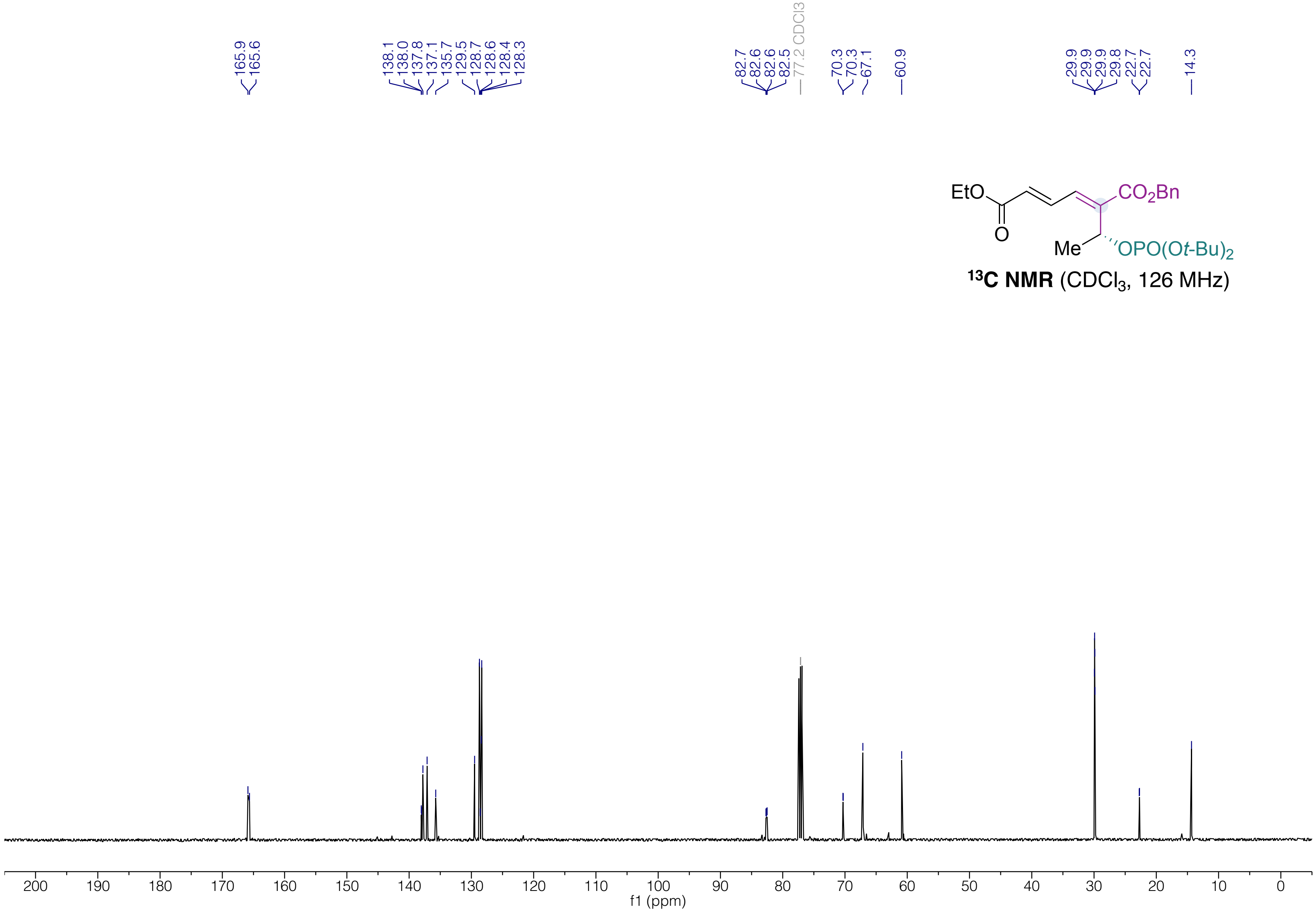

^{13}C NMR ($CDCl_3$, 126 MHz)

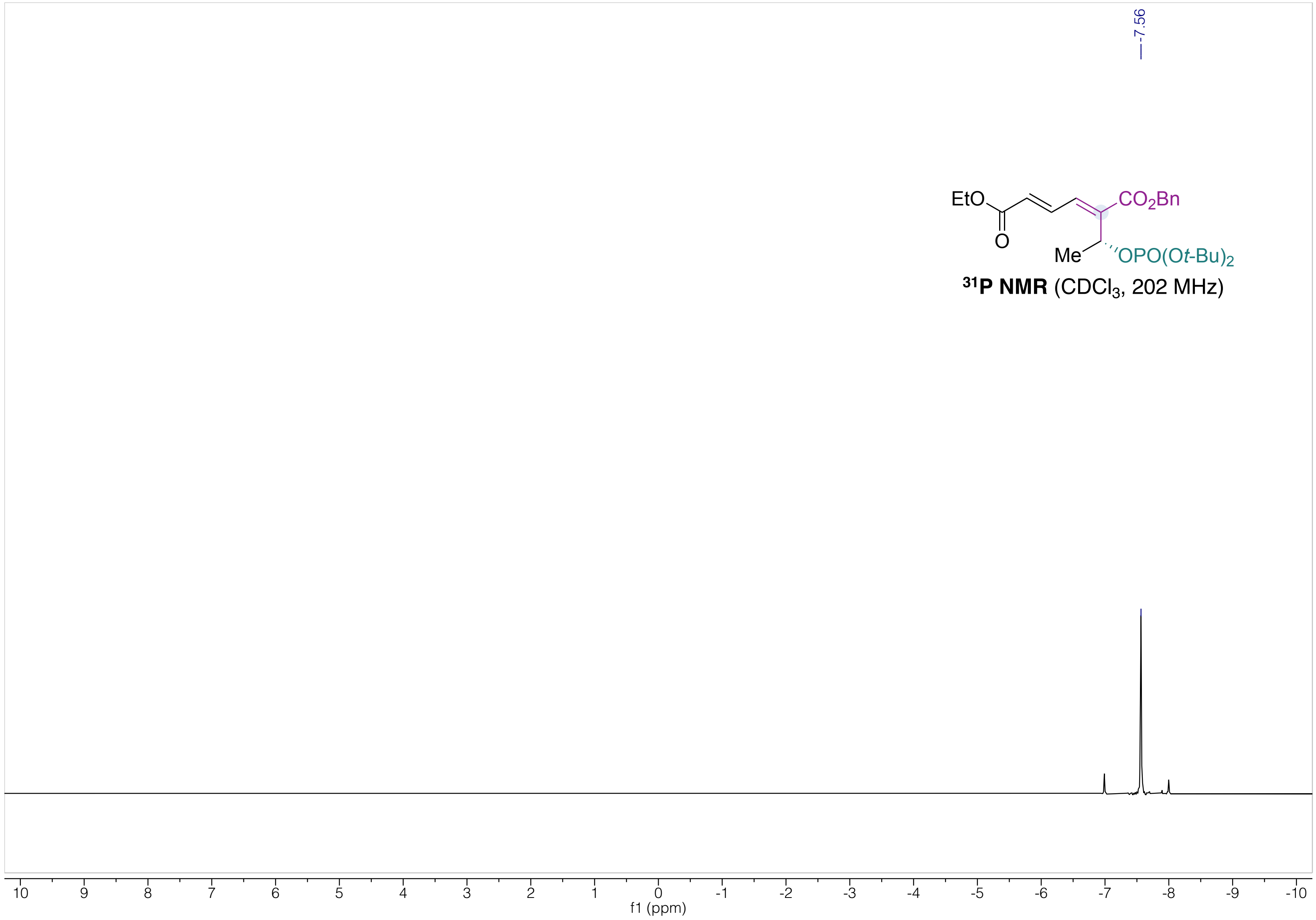

^{31}P NMR ($CDCl_3$, 202 MHz)

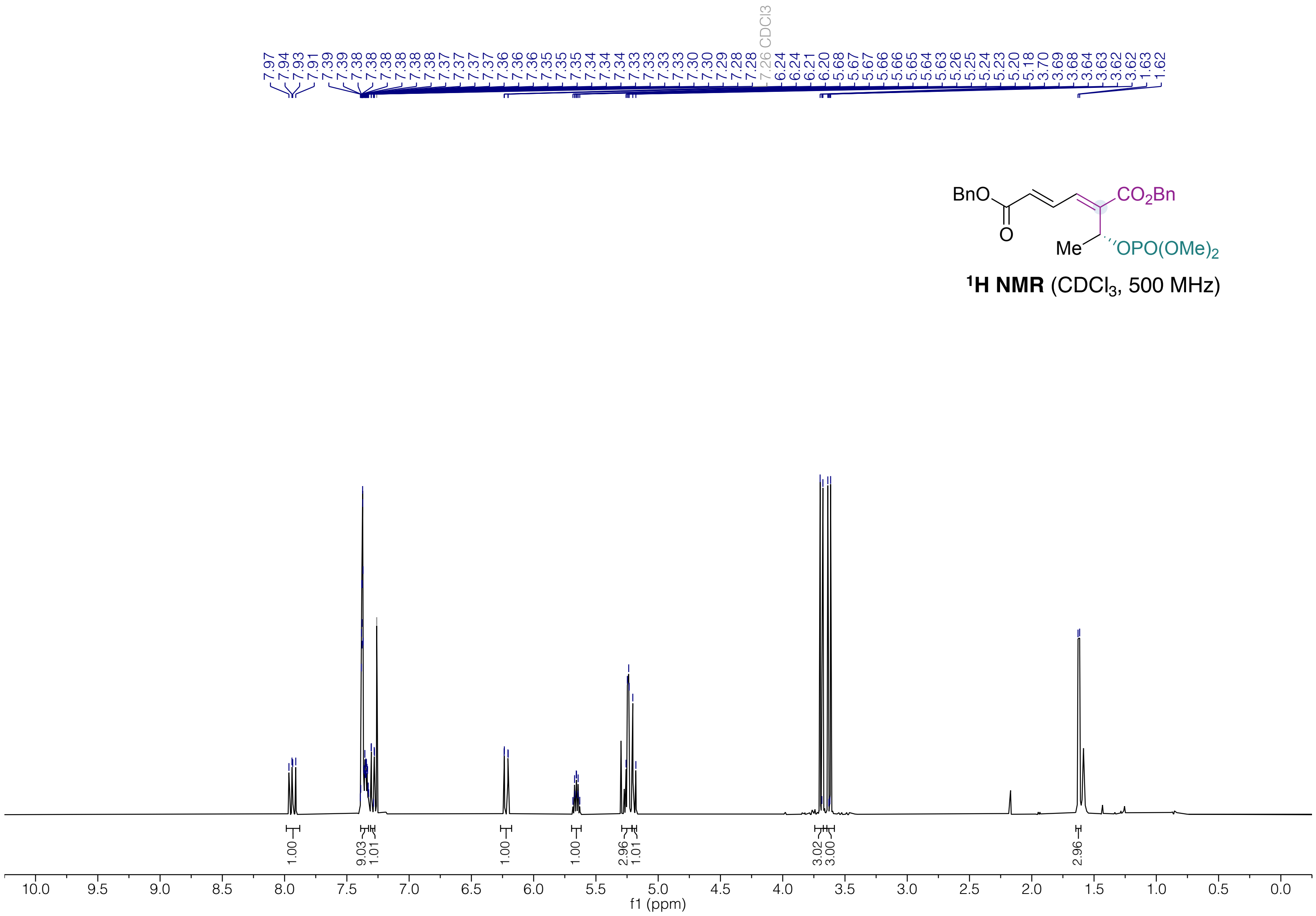
BnO
O
CO2Bn
Me
OPO(OMe)2
1H NMR (CDCl3, 500 MHz)
f1 (ppm)

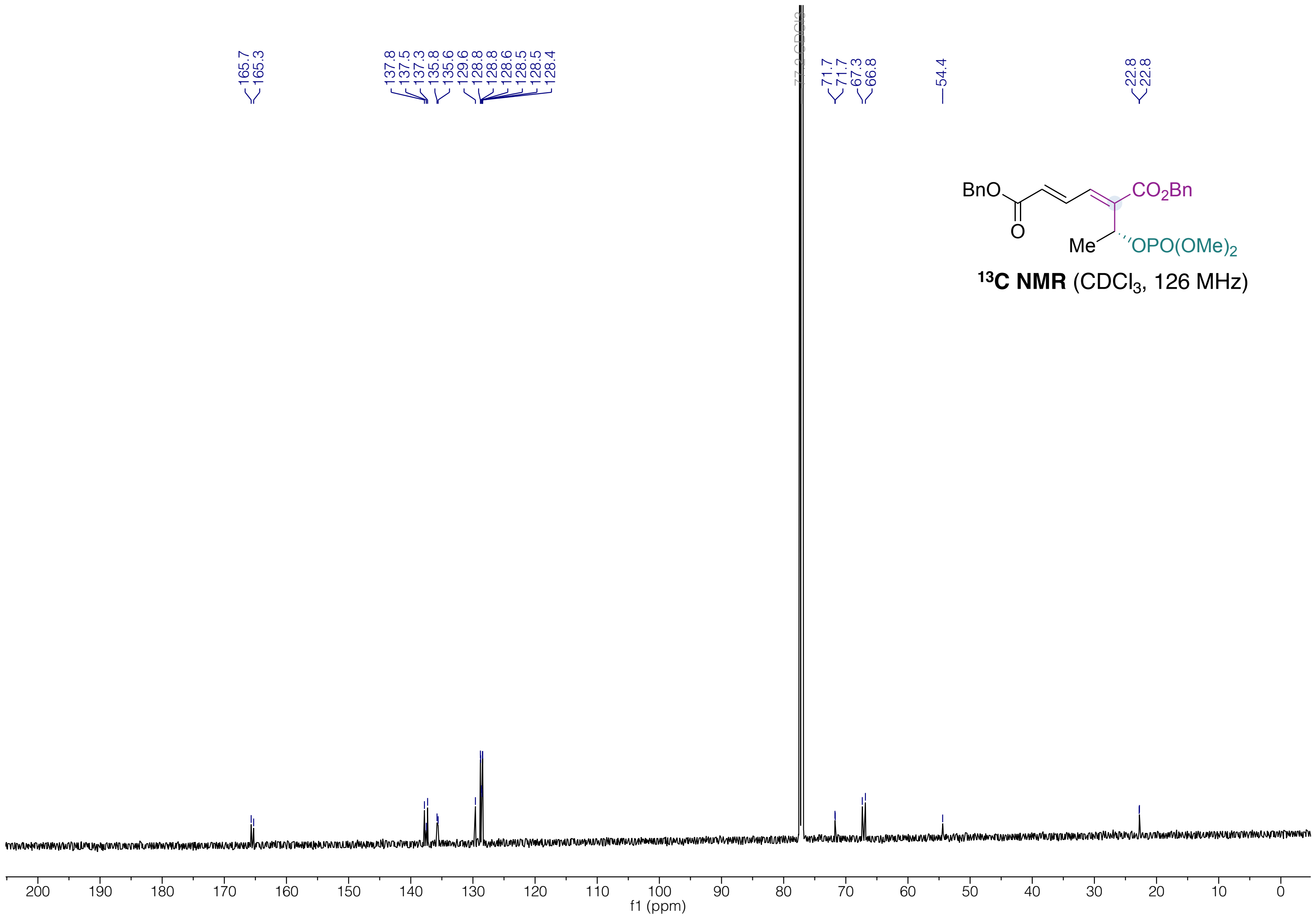
165.7
165.3
137.8
137.5
137.3
135.8
135.6
129.6
128.8
128.8
128.6
128.5
128.5
128.4
77.2 CDCl3
71.7
71.7
67.3
66.8
54.4
22.8
22.8
BnO
O
CO2Bn
Me
OPO(OMe)2
13C NMR (CDCl3, 126 MHz)
f1 (ppm)

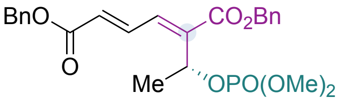

^{31}**P NMR** ($CDCl_3$, 202 MHz)

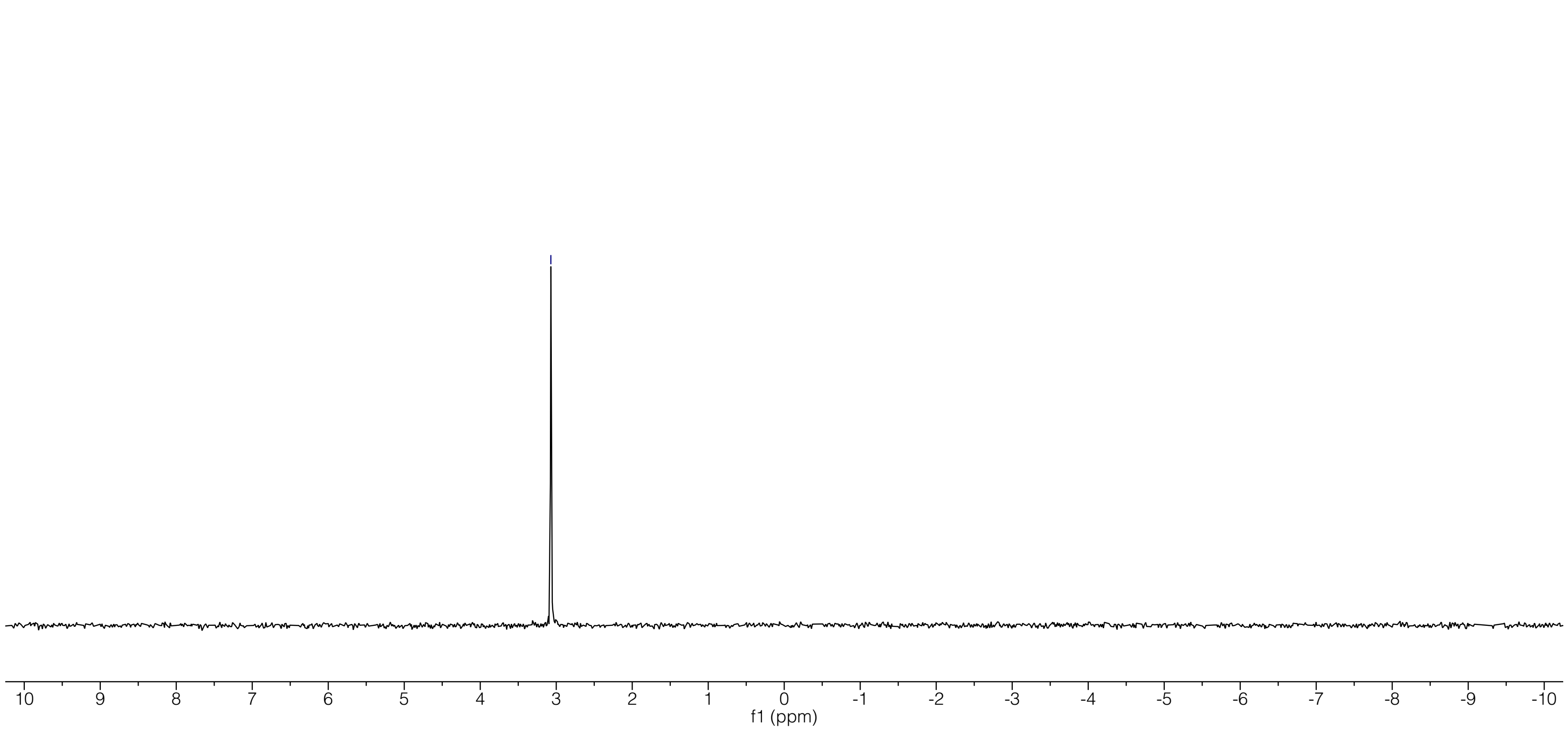

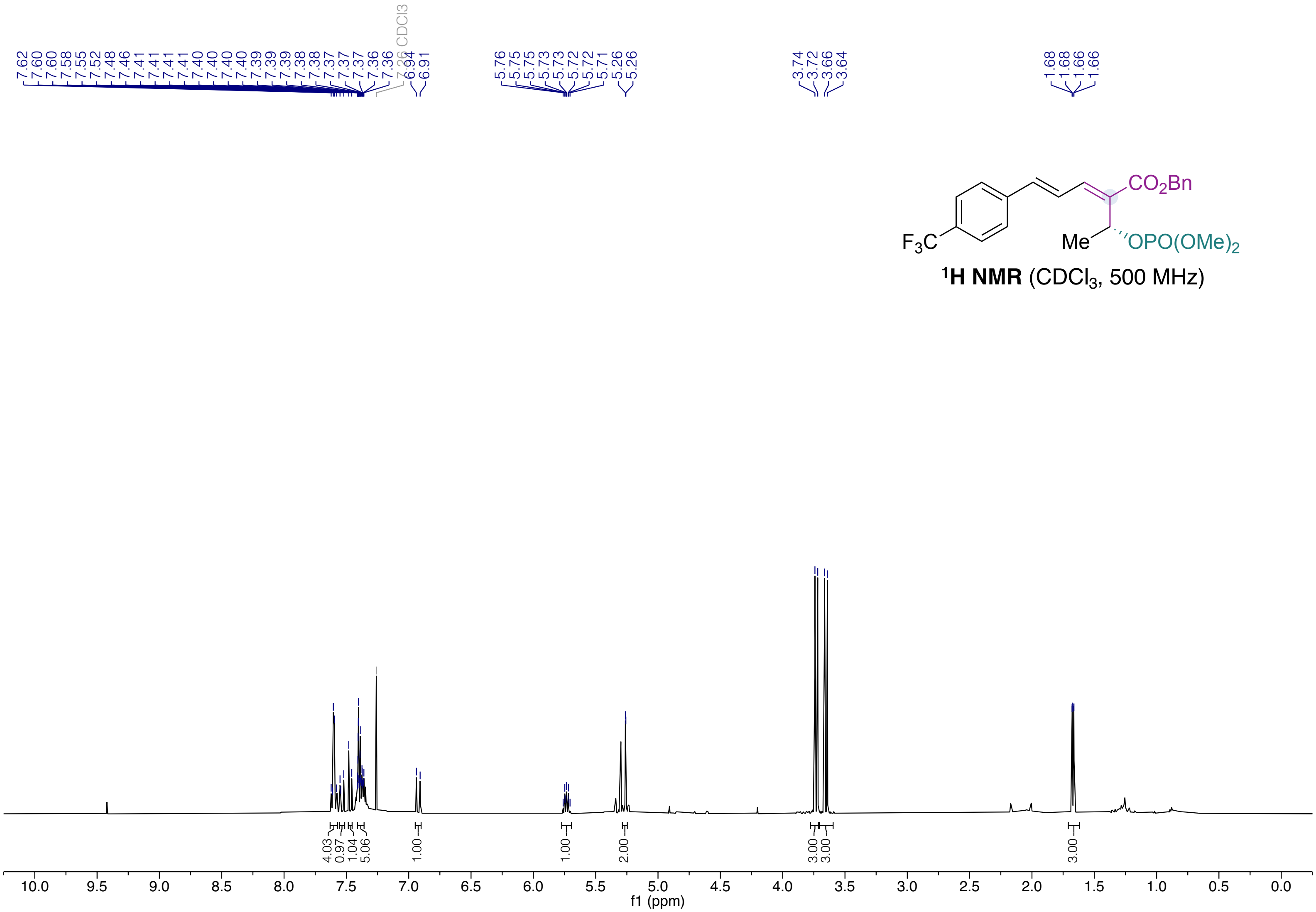

^{1}H NMR ($CDCl_3$, 500 MHz)

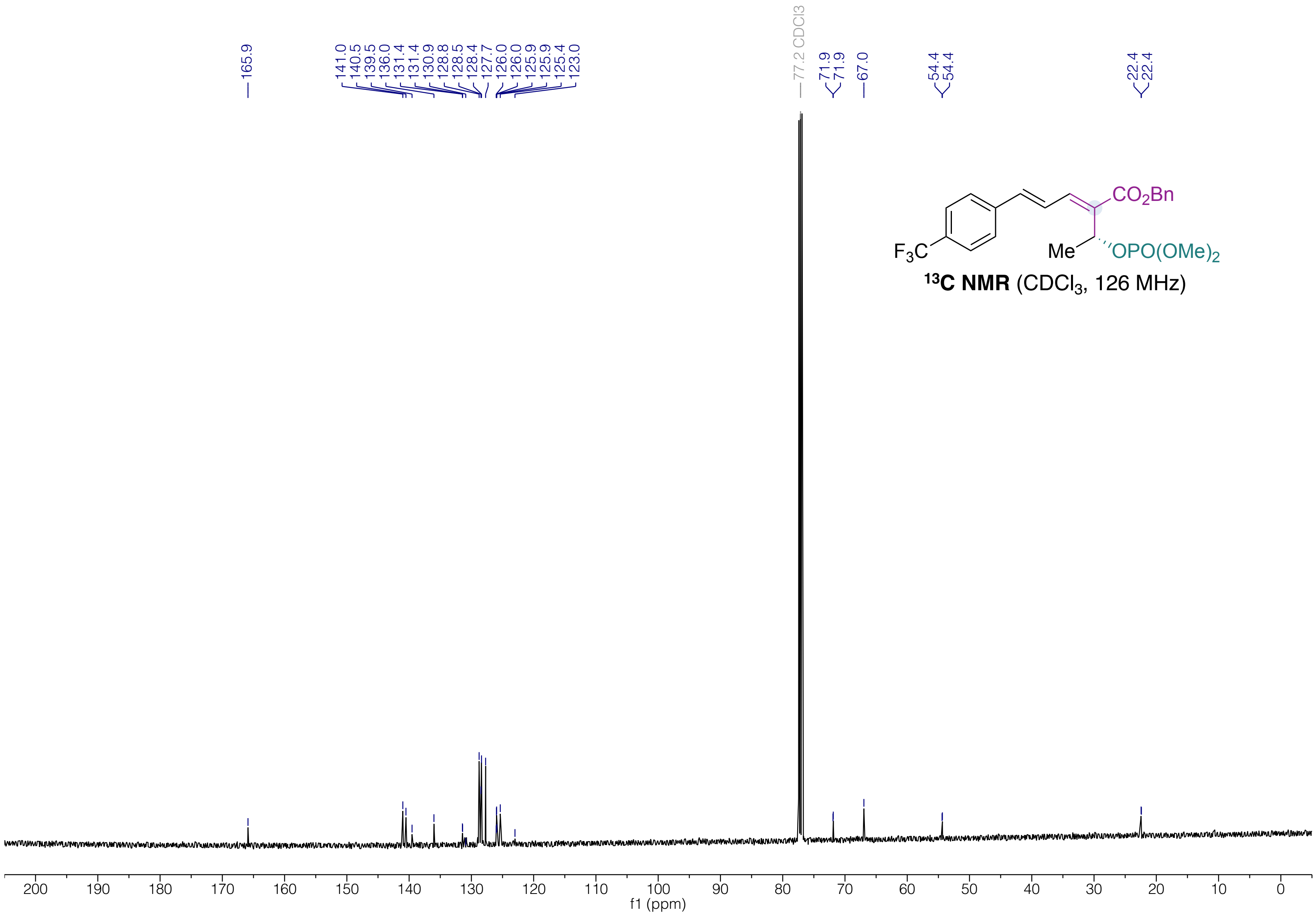
165.9
141.0
140.5
139.5
136.0
131.4
131.4
130.9
128.8
128.5
128.4
127.7
126.0
126.0
125.9
125.9
125.4
123.0
77.2 CDCl3
71.9
71.9
67.0
54.4
54.4
22.4
22.4
CO2Bn
F3C
Me
OPO(OMe)2
13C NMR (CDCl3, 126 MHz)
200 190 180 170 160 150 140 130 120 110 100 90 80 70 60 50 40 30 20 10 0
f1 (ppm)

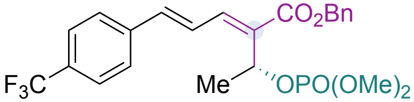

^{19}F NMR ($CDCl_3$, 471 MHz)

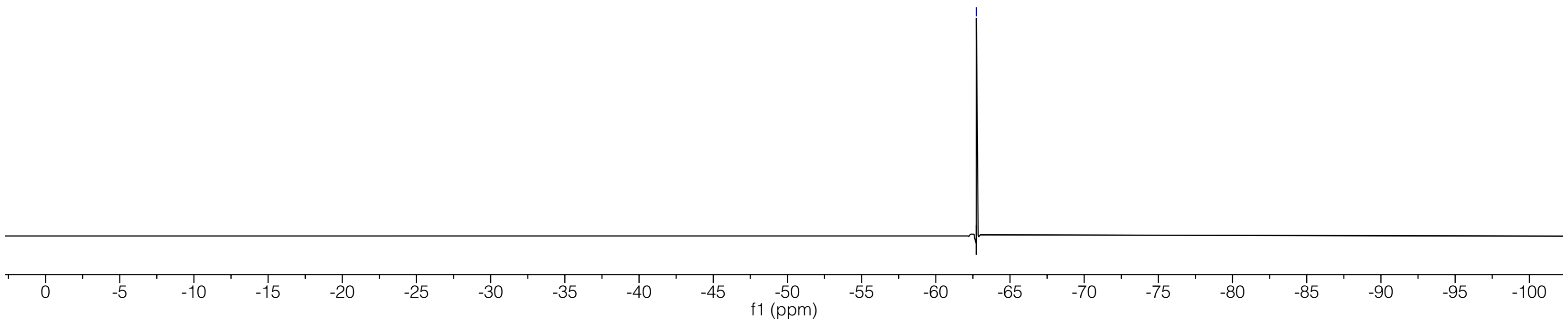

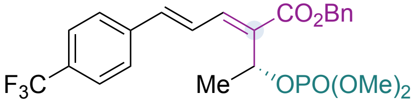

^{31}P NMR ($CDCl_3$, 202 MHz)

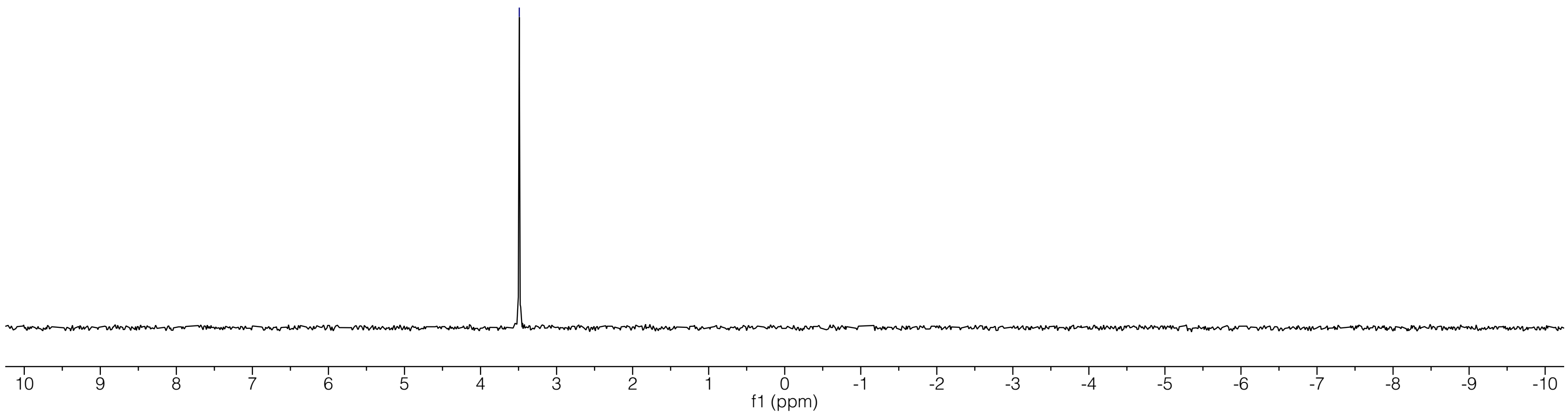

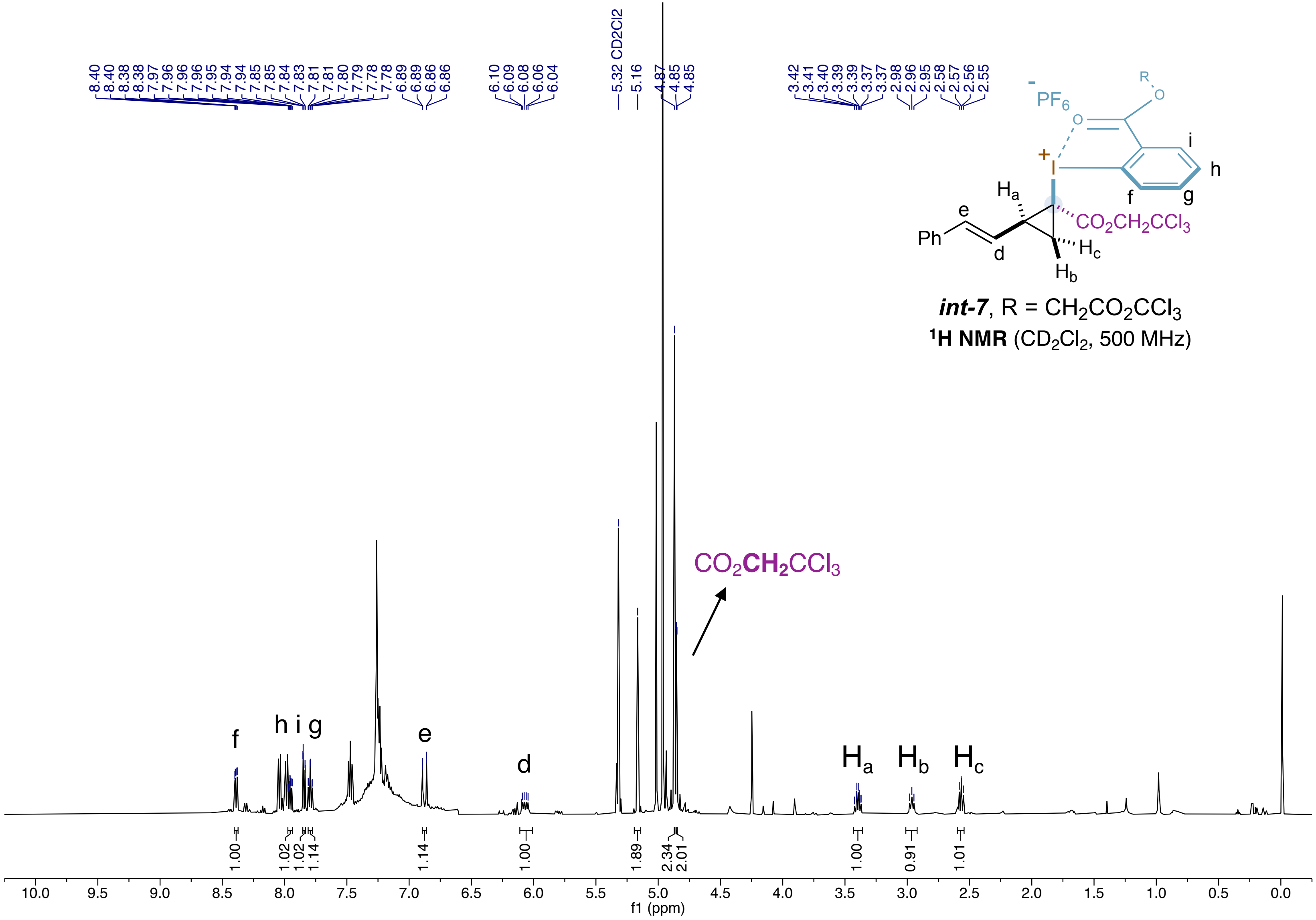

8.40 8.40 8.38 8.38 7.97 7.96 7.96 7.96 7.95 7.94 7.94 7.85 7.85 7.84 7.83 7.81 7.81 7.80 7.79 7.78 7.78 6.89 6.89 6.86 6.86
6.10 6.09 6.08 6.06 6.04
5.32 CD2Cl2
5.16
4.87 4.85 4.85
3.42 3.41 3.40 3.39 3.39 3.37 3.37 2.98 2.96 2.95 2.58 2.57 2.56 2.55
-PF6
R
O
O
+I
i
h
f
g
Ha
e
Ph
d
CO2CH2CCl3
Hc
Hb
int-7, R = CH2CO2CCl3
1H NMR (CD2Cl2, 500 MHz)
CO2CH2CCl3
f
h i g
e
d
Ha
Hb
Hc
1.00 1.02 1.02 1.14 1.14 1.00 1.89 2.34 2.01 1.00 0.91 1.01
10.0 9.5 9.0 8.5 8.0 7.5 7.0 6.5 6.0 5.5 5.0 4.5 4.0 3.5 3.0 2.5 2.0 1.5 1.0 0.5 0.0
f1 (ppm)

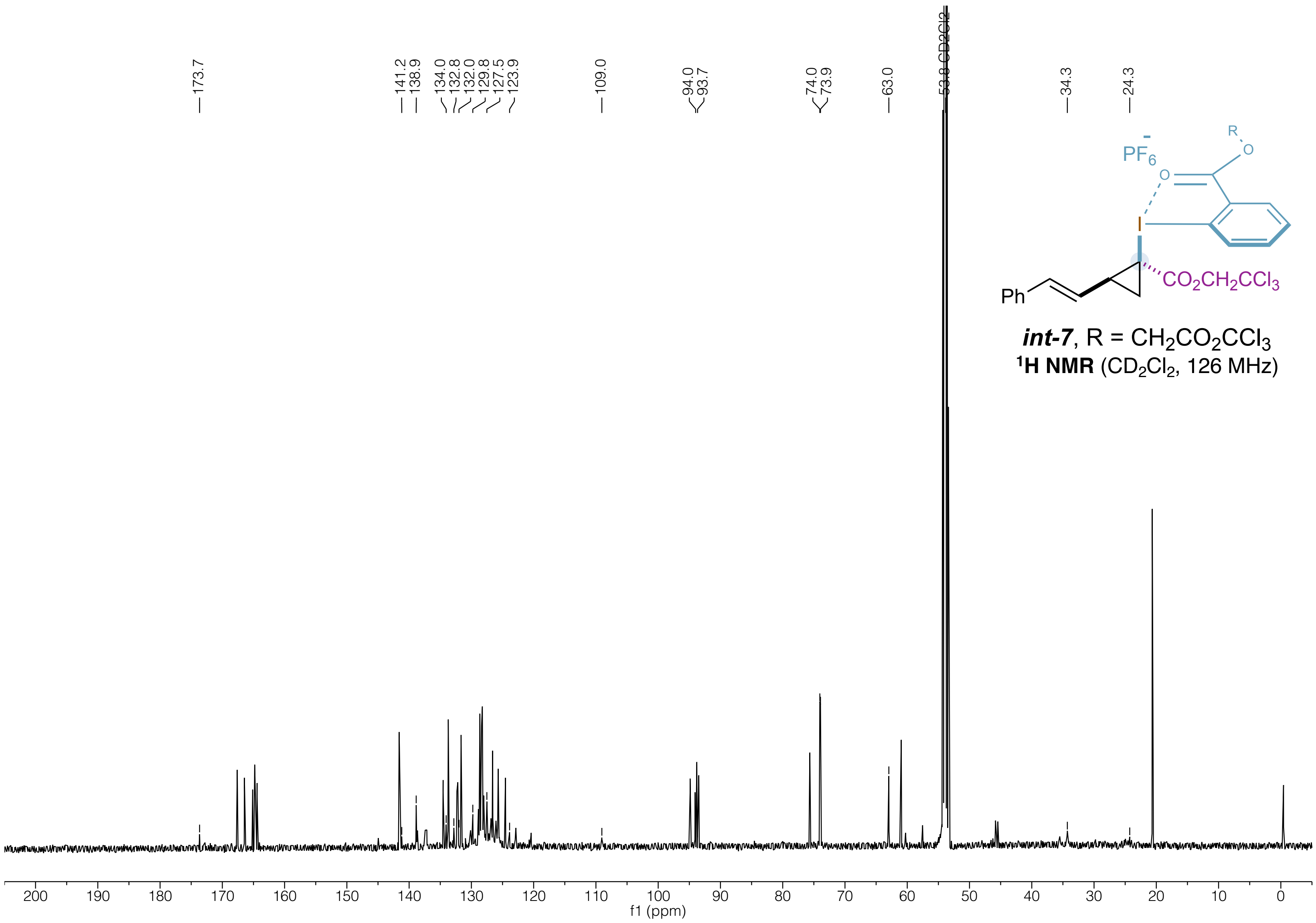

173.7
141.2
138.9
134.0
132.8
132.0
129.8
127.5
123.9
109.0
94.0
93.7
74.0
73.9
63.0
53.8 CD2Cl2
34.3
24.3
PF6
R
O
O
I
Ph
CO2CH2CCl3
int-7, R = CH2CO2CCl3
1H NMR (CD2Cl2, 126 MHz)
200 190 180 170 160 150 140 130 120 110 100 90 80 70 60 50 40 30 20 10 0
f1 (ppm)

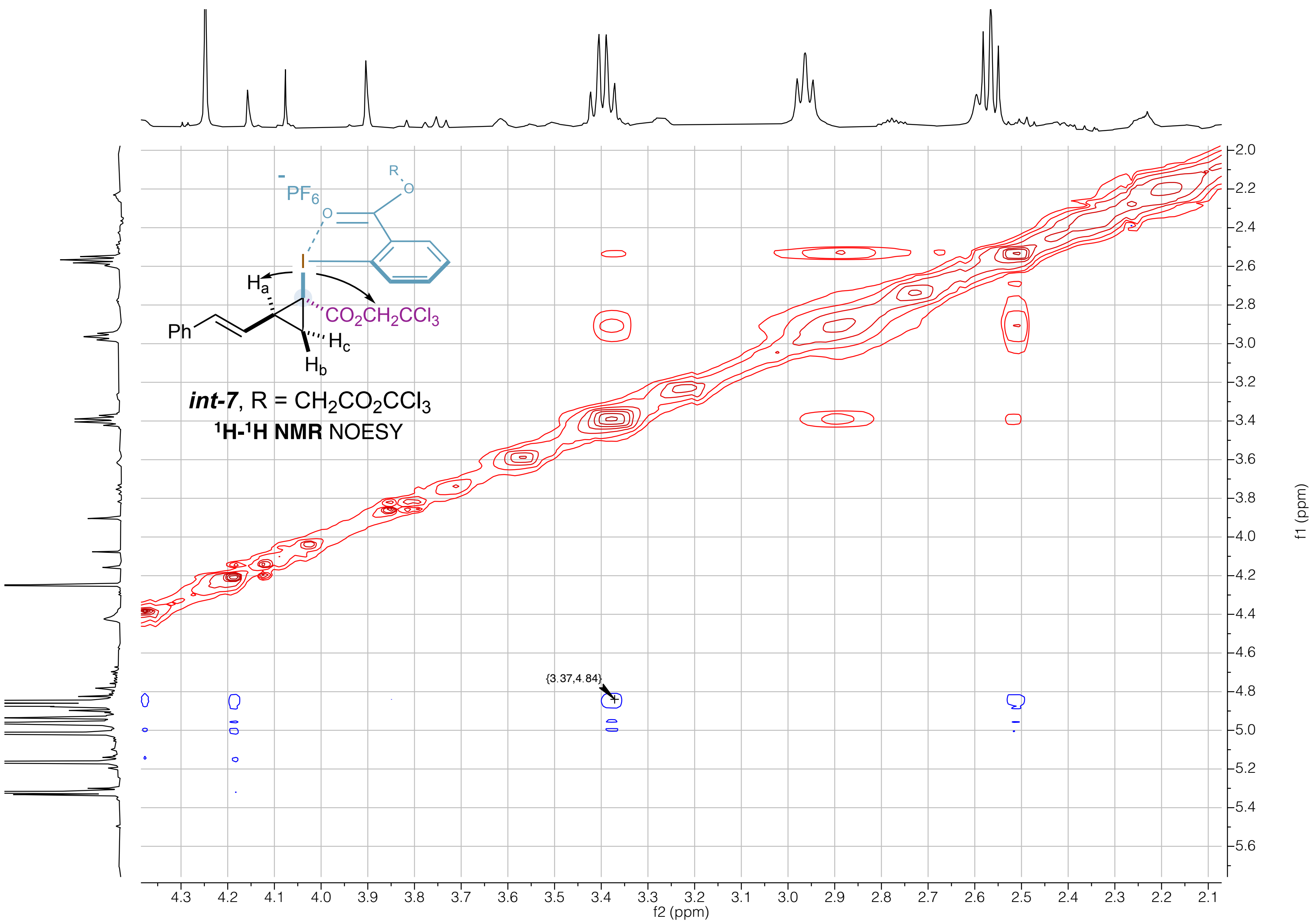
PF6
R
O
O
I
Ha
Ph
CO2CH2CCl3
Hc
Hb
int-7, R = CH2CO2CCl3
1H-1H NMR NOESY
{3.37,4.84}
f2 (ppm)
f1 (ppm)

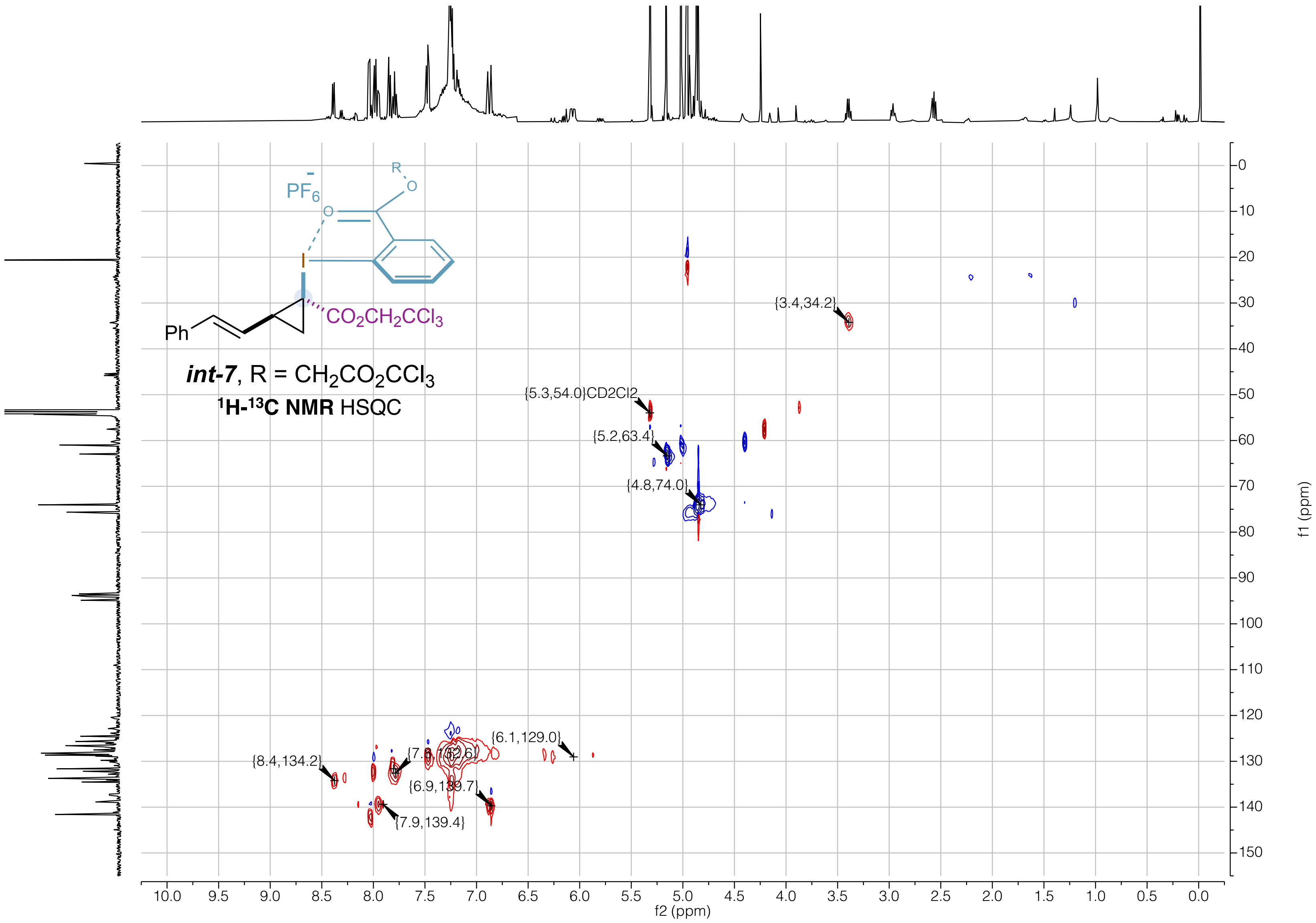

PF6
R
O
O
O
I
CO2CH2CCl3
Ph
int-7, R = CH2CO2CCl3
1H-13C NMR HSQC
{3.4,34.2}
{5.3,54.0}CD2Cl2
{5.2,63.4}
{4.8,74.0}
{6.1,129.0}
{8.4,134.2}
{6.9,139.7}
{7.9,139.4}
f2 (ppm)
f1 (ppm)

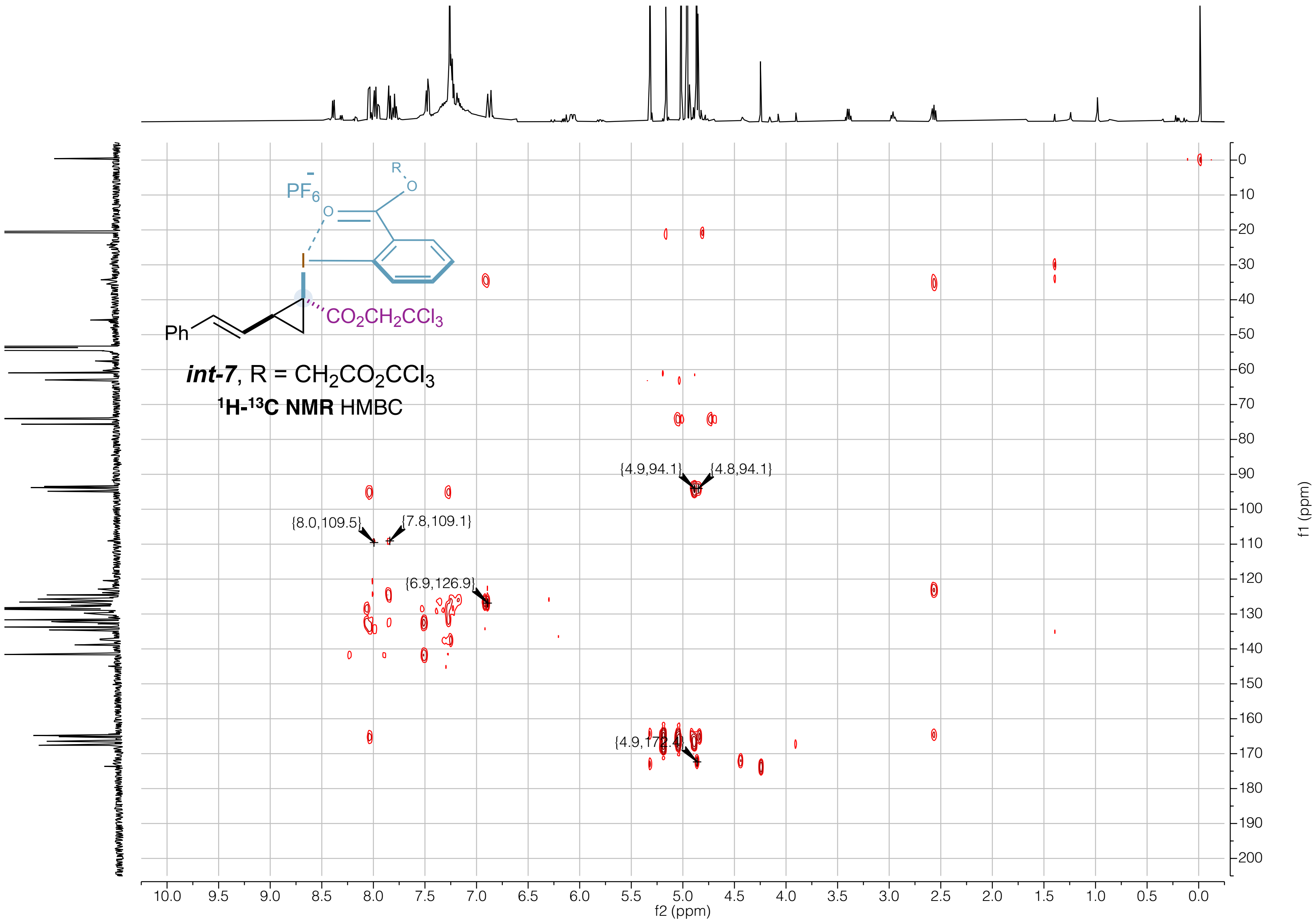

PF6
R
O
O
I
Ph
CO2CH2CCl3
int-7, R = CH2CO2CCl3
1H-13C NMR HMBC
{4.9,94.1}
{4.8,94.1}
{8.0,109.5}
{7.8,109.1}
{6.9,126.9}
f1 (ppm)
f2 (ppm)

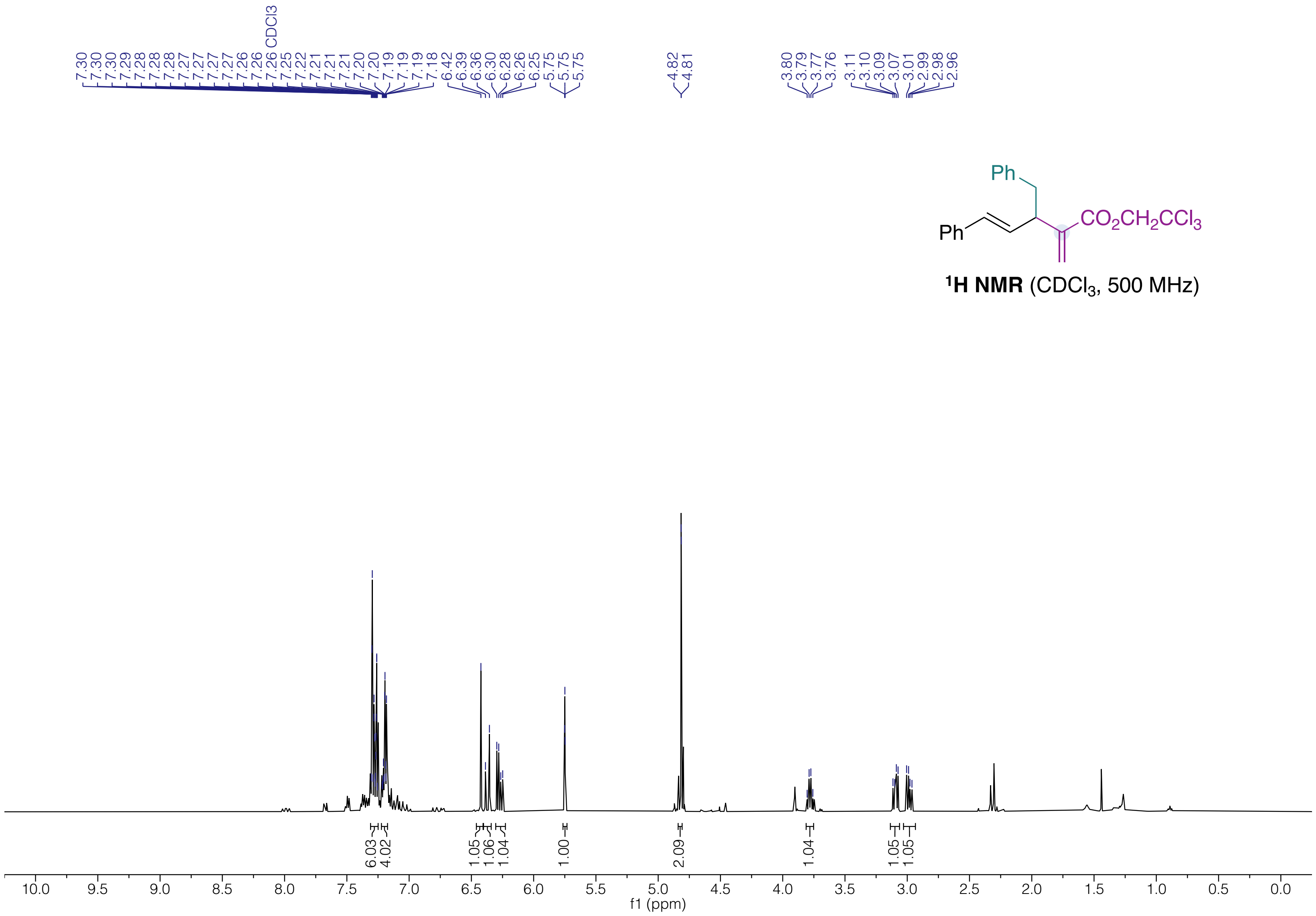
7.30
7.30
7.30
7.29
7.28
7.28
7.27
7.27
7.27
7.26
7.26 CDCl3
7.25
7.22
7.21
7.21
7.21
7.20
7.20
7.19
7.19
7.19
7.18
6.42
6.39
6.36
6.30
6.28
6.26
6.25
5.75
5.75
5.75
4.82
4.81
3.80
3.79
3.77
3.76
3.11
3.10
3.09
3.07
3.01
2.99
2.98
2.96
Ph
Ph
CO2CH2CCl3
1H NMR (CDCl3, 500 MHz)
6.03
4.02
1.05
1.06
1.04
1.00
2.09
1.04
1.05
1.05
10.0
9.5
9.0
8.5
8.0
7.5
7.0
6.5
6.0
5.5
5.0
4.5
4.0
3.5
3.0
2.5
2.0
1.5
1.0
0.5
0.0
f1 (ppm)

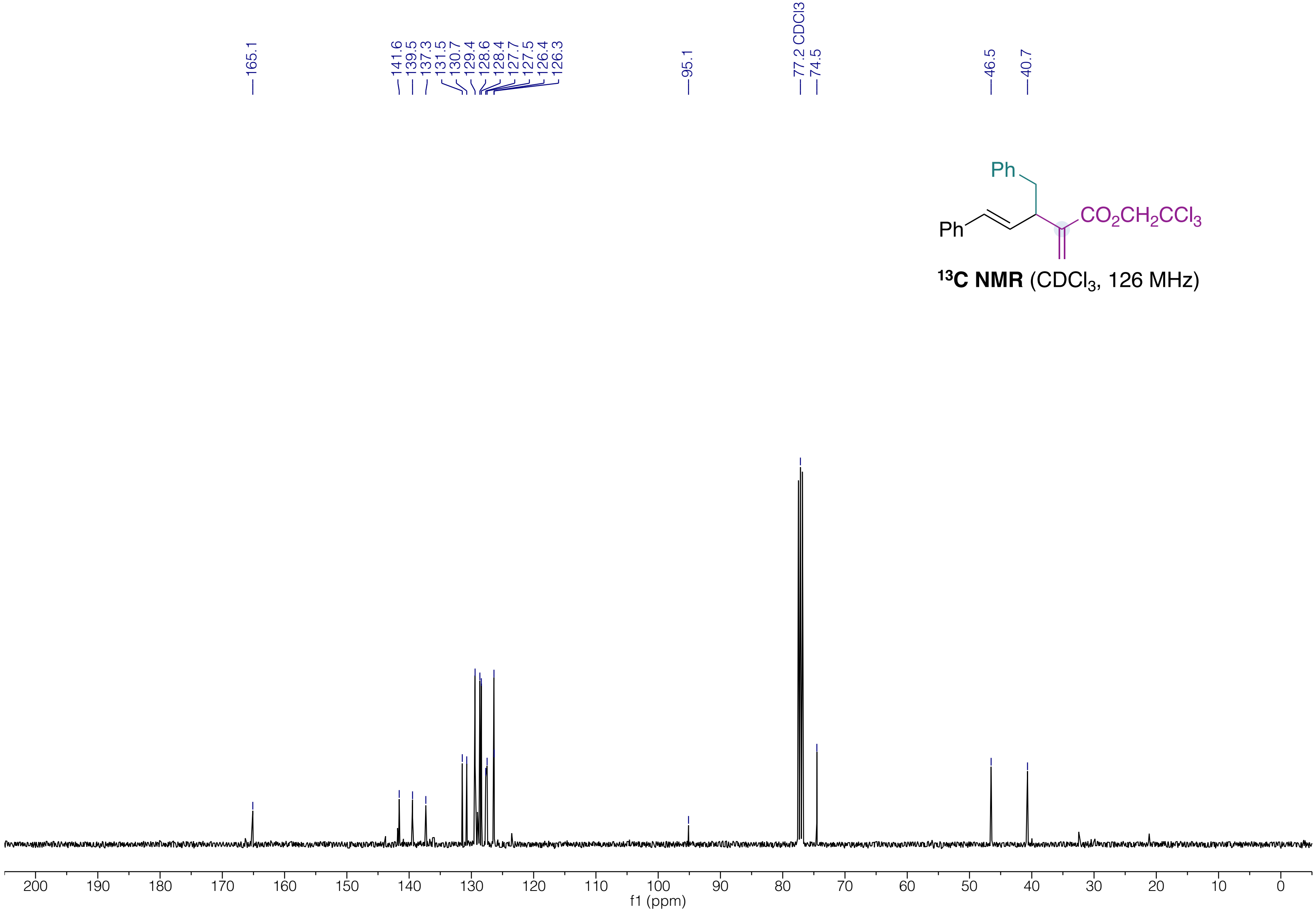

^{13}C NMR ($CDCl_3$, 126 MHz)
